# Iminopyridonato Iridium Complexes: *O*‑Functionalization via C‑X Bond Cleavage

**DOI:** 10.1021/acs.inorgchem.6c00285

**Published:** 2026-05-17

**Authors:** Ondřej Moždiak, Jiří Tydlitát, Zdeňka Růžičková, Andreas Steffen, Roman Jambor

**Affiliations:** † Department of General and Inorganic Chemistry, 48252 University of Pardubice, 532 10 Pardubice, Czech Republic; ‡ Institute of Organic Chemistry and Technology, University of Pardubice, 532 10 Pardubice, Czech Republic; § Department of Chemistry and Chemical Biology, 14311 TU Dortmund University, 442 27 Dortmund, Germany

## Abstract

A new series of 2-pyridonato-based Ir­(III) half-sandwich complexes [(η^5^-Cp*)­IrX­(2-{(2,6-*i*Pr_2_–C_6_H_3_)N = CH}–C_5_H_3_N–6-(O))] (**3**: X = Cl; **4**: X = I) were prepared via a thermally initiated demethylation of the corresponding ionic complexes [(η^5^ -Cp*)­IrX­(κ^2^-{2-[(2,6-*i*Pr_2_–C_6_H_3_)­N=CH]-6-(OMe)­C_5_H_3_N})]­(X) (**1**: X = Cl; **2**: X = I). These neutral complexes **3** and **4** were further studied as versatile precursors for the preparation of unprecedented polymetallic Ir–Zn and Ir–Zn–Ru complexes. Remarkably, complexes **3** and **4** could activate C­(sp^3^)-X bonds, leading to the isolation of several O-substituted ionic products. Alongside this *O*-functionalization, substitution of the Ir–Cl bond by Ir–X (X = Br or I) was also observed during this reaction and both processes were investigated in detail to provide mechanistic insight.

## Graphical abstract

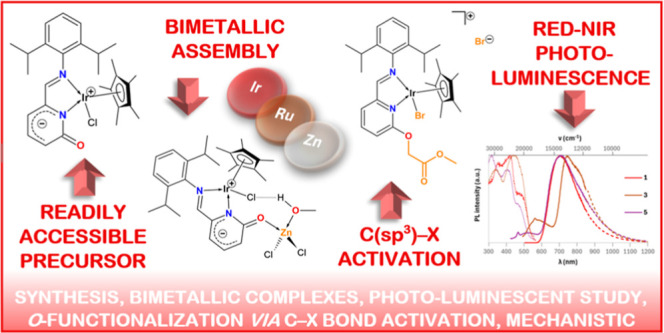

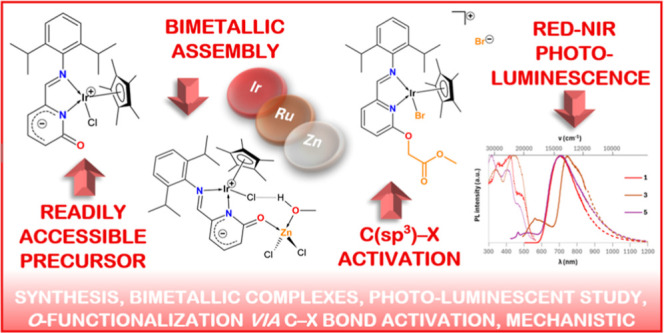

## Introduction

Pyridonato ligands have emerged as versatile scaffolds in coordination chemistry due to their ambidentate character.^1^ Apart from the use as a spectator ligand supporting metal-centered reactivity, complexes based on 2-pyridonato ligand may be involved in the bond breaking and bond forming reactions. Such synergetic bond activation of substrates is described in modern literature as metal–ligand cooperation (MLC).^1,2^ Their ability to participate in proton transfer, hydrogen bonding, and reversible aromatization/dearomatization processes has made pyridonate complexes particularly attractive for catalytic transformations^3–9^ and organic substrate activations.^6–9^ In 2015, *Szymczak* reported observation of E–H bond splitting mediated by Ru­(II) 2-pyridonato complex [{2-(C_5_H_3_NOH)–C_8_H_4_N_3_-2-(C_5_H_3_NO)}­(PPh_3_)_2_Ru], which was capable to react with H_2_ and HBPin.^6^ These systems have been primarily employed for the activation of various E–H bonds (Figure 1) so far.^1^ Three main activation pathways have been identified depending on the polarity of the E–H bond (Figure 1). For substrates bearing acidic protons E­(δ^–^)–H­(δ^+^), interaction with 2-pyridonato complexes results in the formation of a pyridinol fragment with a new O–H bond, accompanied by the generation of a new TM–E bond or TM^+^ cation compensated by E^–^ anion.^7,8^ Substrates with opposite polarity of the E­(δ^+^)–H­(δ^–^) bond (e.g., boranes, silanes) react with these complexes providing O-substituted pyridinole-based ligands bearing new O–E bonds accompanied by the formation of a new TM–H hydride species.^6^ Finally, reactions with H_2_, as an example of the E–H substrate with a nonpolar bond, occur as heterolytic cleavage reactions yielding TM-H fragment together with either ligand with new O–H bond or N–H bond.^8,9^ In our previous work, we reported the synthesis of Ru­(II) 2-pyridonato complexes [(η^6^-*p*-cymene)­RuX­(2-{(2,6-*i*Pr_2_–C_6_H_3_)­N=CH}–C_5_H_3_N–6-(O))] (X = Cl or I).^10^

**Figure 1:**
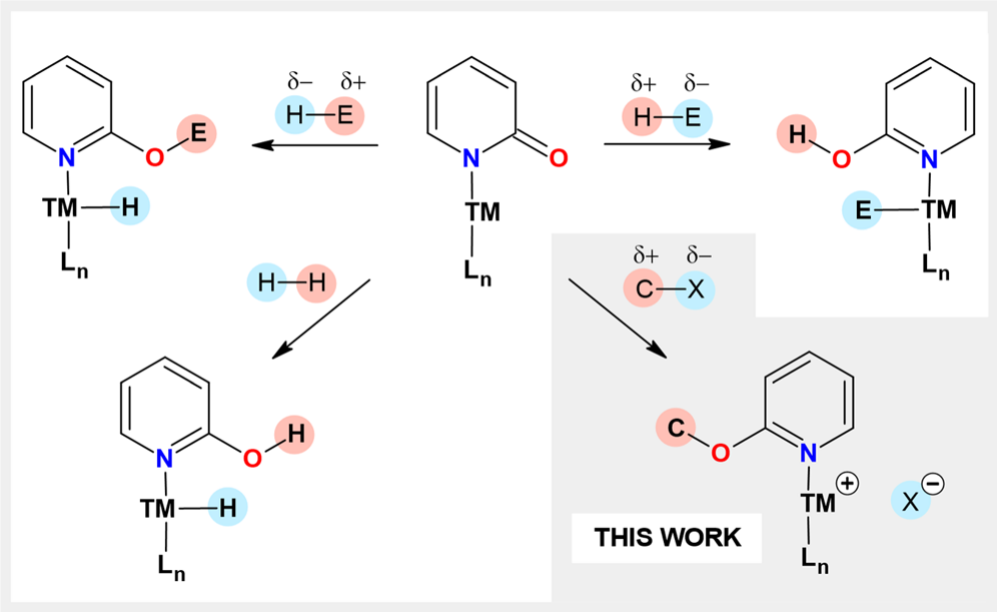
Known MLC activations of E–H substrates mediated by TM with 2-pyridonato ligands.

In this study, new series of 2-pyridonato-based Ir­(III) half-sandwich complexes [(η^5^ -Cp*)­IrX­(2-{(2,6-*i*Pr_2_–C_6_H_3_)N = CH}–C_5_H_3_N–6-(O))] (**3**: X = Cl; **4**: X = I) were prepared via a thermally initiated demethylation of the corresponding ionic complexes [(η^5^ -Cp*)­IrX­(κ2-{2-[(2,6-*i*Pr_2_–C_6_H_3_)N = CH]-6-(OMe)­C_5_H_3_N})]­(X) (**1**: X = Cl; **2**: X = I) bearing the same ligand **L**. To prove electron-rich character of the C=O group in the 2-pyridonato-Ir­(III) complexes **3** and **4**, they were further studied as versatile precursors for the preparation of Ir–Zn and Ir–Zn–Ru complexes. Finally, the presence of an electron-rich C=O bond in complex **3** prompted us to study C–X bond activations, as an unexplored type of the reactivity for TM 2-pyridonato complexes (Figure 1). Previously reported C–X bond activations involve both main-group^11,12^ and transition-metal systems^13,14^ based on frustrated Lewis pairs.

## Results and Discussion

### Synthesis and Characterization of Ir­(III) 2-Pyridonato Complex

As stated, similar to Ru­(II)-assisted MeI elimination, we applied the same procedure to ionic Ir­(III) complexes [(η^5^ -Cp*)­IrX­(κ2-L)]­(X) (**1**: X = Cl; **2**: X = I) bearing the same ligand **L** to see whether the TM-assisted MeX elimination can be generalized for the synthesis of TM 2-pyridonato complexes with a free, electron-rich C=O group. Therefore, both solid compounds **1** and **2** were heated for 2 h at 120 °C in air (Scheme 1) affording dark orange powders of [(η^5^ -Cp*)­IrX­(2-{(2,6-*i*Pr_2_–C_6_H_3_)N = CH}–C_5_H_3_N–6-(O))] (**3**: X = Cl; **4**: X = I) in quantitative yield. Complexes **1**–**4** were characterized using NMR, FT-IR, and UV/vis spectroscopy (Figures S1–S8 and S42–S45 and S56), while **3** and **4** were additionally studied by SC-XRD.

**Scheme 1:**
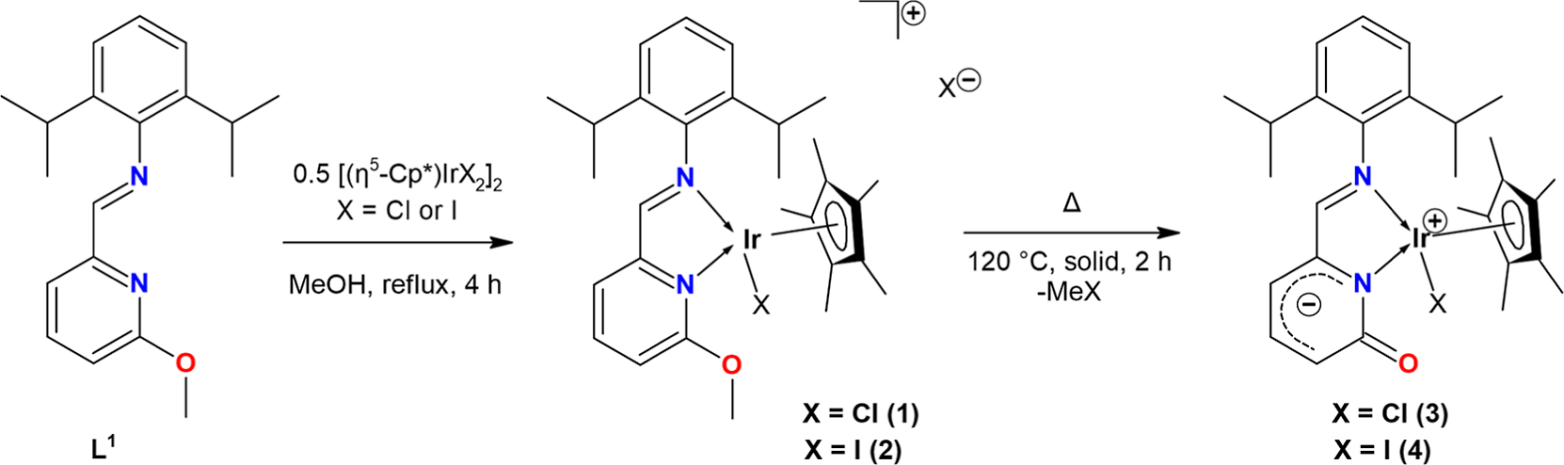
Preparation of Ionic Ir­(III) Complexes **1** and **2** and Their Thermal Demethylation to Neutral Ir­(III) Complexes **3** and **4**

The ^1^H NMR spectroscopy showed the signal of the C*H*=N proton resonating at δ 9.18 (**1**) or 8.87 (**2**) ppm, respectively, for ionic **1** and **2**. This value is shifted upfield to δ 8.33 (**3**) and 8.28 (**4**) ppm for neutral **3** and **4**. In the ^1^H NMR spectra of **3** and **4**, the OMe signal is absent, confirming MeI elimination from the starting **1** and **2**. The formation of the Ir­(III) 2-pyridonato complexes **3** and **4** with the C=O group is demonstrated by new signals at δ 167.7 ppm (**3**) or 168.3 ppm (**4**) in the ^13^C­{^1^H} NMR spectra of **3** and **4**. This was also supported by FT-IR spectroscopy, which showed strong absorptions at ν_(C=O)_ = 1556 cm^–1^ for **3** and 1548 cm^–1^ for **4**.

Single crystals of **3** and **4** were grown in toluene (**3**) and DCM/hexane 2:1 (**4**) and are depicted in Figure 2 (for crystallographic details, see Table S1). The molecular structures of **3** and **4** proved the formation of neutral Ir­(III) complexes featuring an *N*-bound 2-pyridonato ligand. This is demonstrated by Ir1–N1 (2.106(2) A for **3**, 2.086(4) A for **4**) and C1–O1 (1.243(4) A for **3**, 1.]­8(6) for **4**) bond distances, that are close to a C=O double bond (Σ_dcov_ C=O = 1.24 Å)^15^ and are similar to those found in related 2-pyridonato complexes.^7–10,16,17^

**Figure 2:**
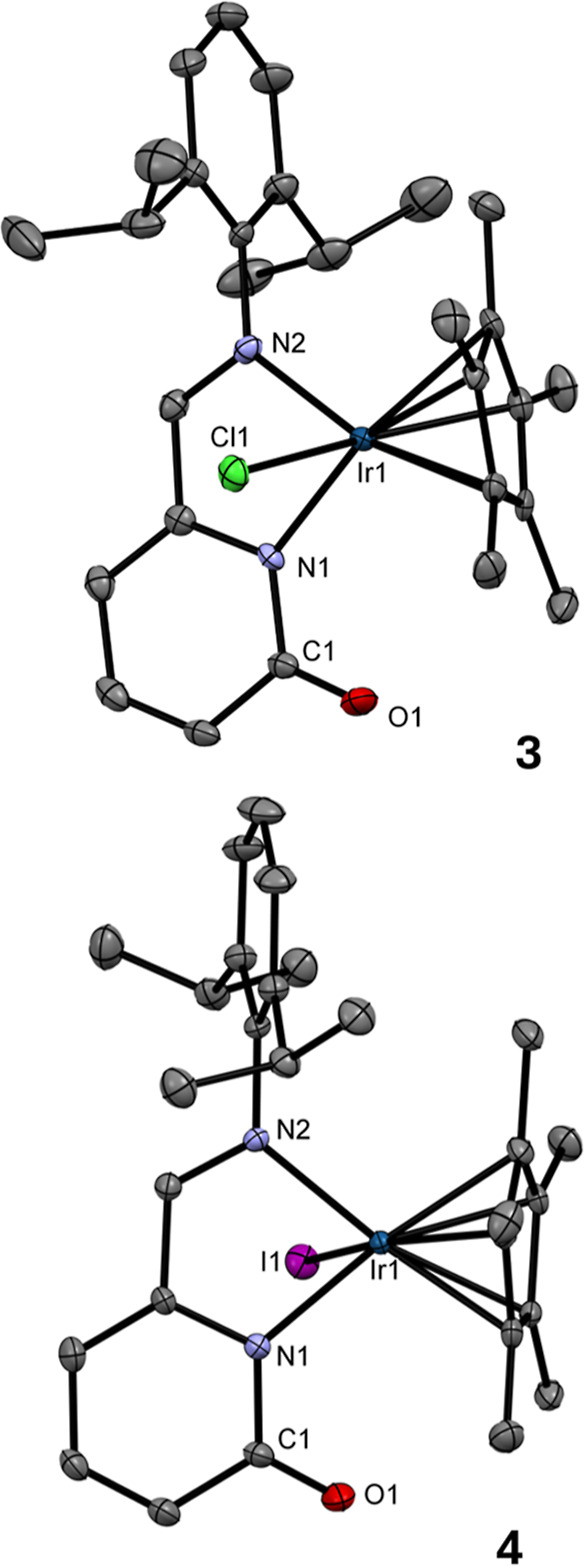
ORTEPs of **3** and **4**. Non-H atoms are represented by 50% probability ellipsoids. H atoms and molecule of C_7_H_8_ (**3**) are omitted for clarity. For **3**: bond lengths (Å): Ir1–N1 2.106(2); Ir1–N2 2.125(3); Ir1–Cl1 2.4324(8); C1–O1 1.243(4). Bond angles (°): N1–Ir1–N2 76.8(1); N1–Ir1–Cl1 80.53(7); N2–Ir1–Cl1 90.54(7). For **4**: bond lengths (Å): Ir1–N1 2.086(4); Ir1–N2 2.122(4); Ir1–I1 2.7083(4); C1–O1 1.238(6). Bond angles (°): N1–Ir1–N2 77.1(1); N1–Ir1–I1 81.6(1); N2–Ir1–I1 91.5(1).

To prove electron-rich character of the C=O bond in the 2-pyridonato-based complexes **3** and **4**, complex **3** was tested as a potential O-donor ligand. Therefore, the stoichiometric reaction of **3** with ZnCl_2_ in MeOH was run yielding new complex [(η^5^ -Cp*)­IrCl­(2-{(2,6-*i*Pr_2_–C_6_H_3_)­N=CH}–C_5_H_3_N–6-(O→)]­ZnCl_2_(MeOH) (**5**), the product of C = O→Zn coordination (Scheme 2). Notably, variation of the reaction stoichiometry did not affect the composition of the isolated product. Complex **5** was characterized by NMR, FT-IR, and UV/vis spectroscopies (Figures S9, S10, S46, and S57) and by SC-XRD analysis.

**Scheme 2:**
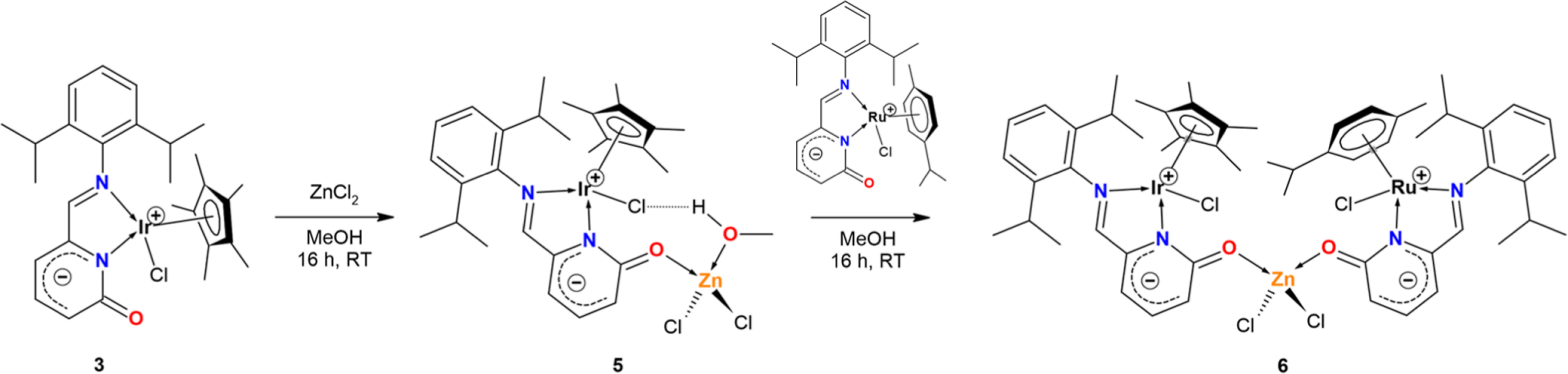
Preparation of Polymetallic Complexes **5** and **6**

In the ^1^H NMR spectrum of **5**, the signal of the C*H* = N group at δ 8.40 ppm is shifted downfield relative to starting neutral complex **3** (δ 8.33) yet still shifted upfield compared to the ionic precursor **1** (δ 9.18). Single crystals of **5** were grown in MeOH at RT, and their molecular structures are depicted in Figure 3 (for crystallographic details, see Table S1). The molecular structure of **5** proved the coordination of **3** to ZnCl_2_ via C=O → Zn coordination. Consequently, the C1–O1 bond distance (1.270(4) Å) in **5** is elongated compared to free **3** (1.243(4) Å). The tetrahedral arrangement of the Zn1 atom is completed by two Cl atoms and O2 atom of MeOH. The isolation of **5** suggests that the coordination ability of **3** differs from 2-pyridonato Ru­(II) analogue [(η^6^-*p*-cymene)­RuCl­(2-{(2,6-*i*Pr_2_–C_6_H_3_)­N=CH}–C_5_H_3_N–6-(O))] since its reaction with ZnCl_2_ yielded the symmetric Ru–Zn complex [(η^6^-*p*-cymene)­RuCl­(2-{(2,6-*i*Pr_2_–C_6_H_3_)­N=CH}–C_5_H_3_N–6-(O))]_2_(ZnCl_2_).^10^ As the Zn atom in **5** is coordinated by one solvent MeOH molecule, we tested additional stoichiometric reaction of **5** with [(η^6^-*p*-cymene)­RuCl­(2-{(2,6-*i*Pr_2_–C_6_H_3_)­N=CH}–C_5_H_3_N–6-(O))], as additional Ru-based LB. This reaction yielded heterotrimetallic complex [(η^5^ -Cp*)­IrCl­(2-{(2,6-*i*Pr_2_–C_6_H_3_)­N=CH}–C_5_H_3_N–6-(O→))]­[(η^6^-*p*-cymene)­RuCl­(2-{(2,6-*i*Pr_2_–C_6_H_3_)N = CH}–C_5_H_3_N–6-(O→))]­(μ-ZnCl_2_) (**6**) (Scheme 2), characterized by NMR, FT-IR, and UV/vis spectroscopies (Figures S11, S12, S47, and S57) and by SC-XRD analysis.

**Figure 3:**
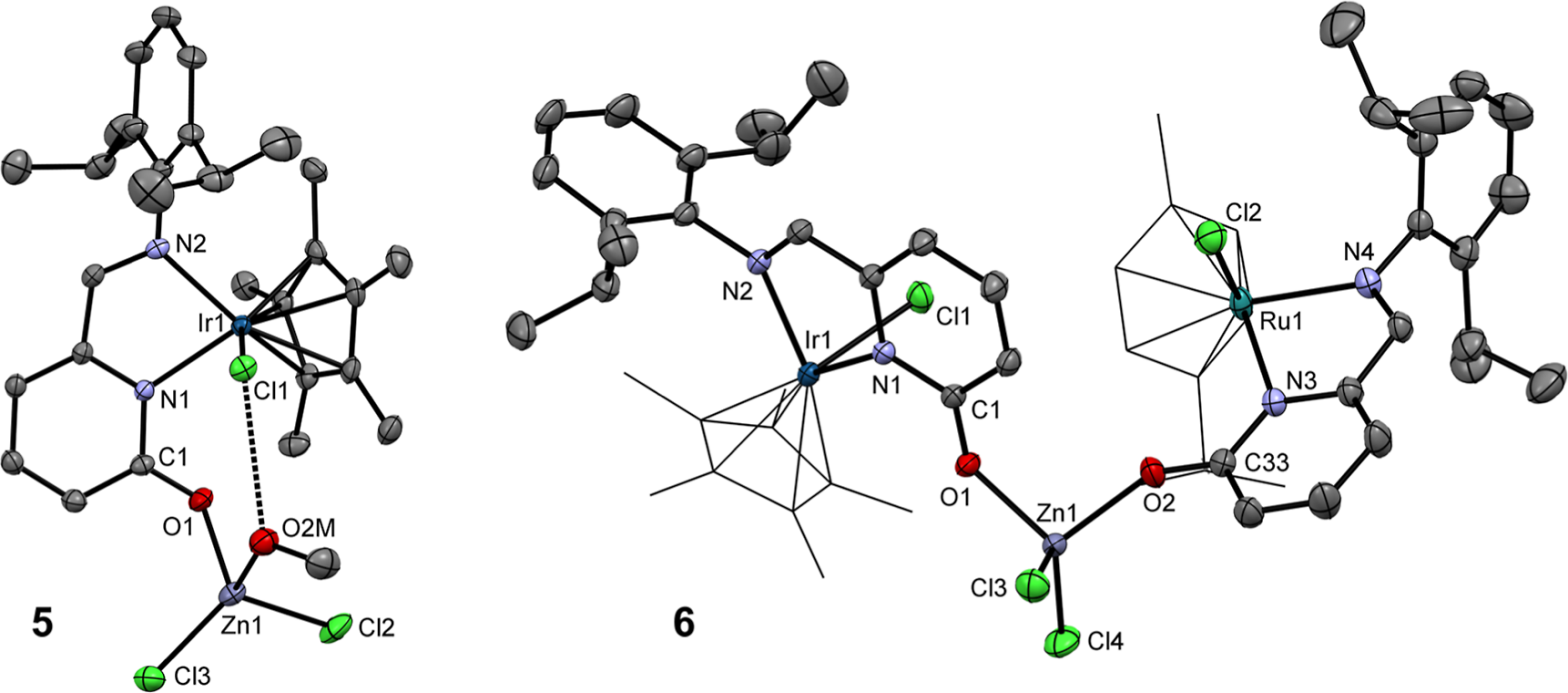
ORTEPs of **5** and **6**. Non-H atoms are represented by 50% probability ellipsoids. H atoms and noncoordinated MeOH molecule in **5**·**MeOH** are omitted for clarity. For **5**: bond lengths (Å): Ir1–N1 2.128(3); Ir1–N2 2.121(2); Cl1–Ir1 2.4436(9); C1–O1 1.270(4); Zn1–O1 1.956(2); Zn1–O2M 2.064(3); Zn1–Cl2 2.219(1); Zn1–Cl3 2.200(1); Cl1–H2M 2.23(4). Bond angles (°): N1–Ir1–N2 76.4(1); O1–Zn1–O2M 89.9(1); Cl2–Zn1–Cl3 118.19(4). For **6**: bond lengths (Å): Ir1–N1 2.118(2); Ir1–N2 2.132(2); Ru1–N3 2.117(2); Ru1–N4 2.101(2); Cl1–Ir1 2.4157(6); Cl2–Ru1 2.4039(8); C1–O1 1.273(3); C33–O2 1.273(3); Zn1–O1 1.982(2); Zn1–O2 1.977(2); Zn1–Cl3 2.2325(8); Zn1–Cl4 2.2309(9). Bond angles (°): N1–Ir1–N2 76.62(7); N3–Ru1–N4 76.43(8); O1–Zn1–O2 98.37(8); Cl3–Zn1–Cl4 118.83(3).

In the ^1^H NMR spectrum of **6**, two signals of the C*H*=N groups (δ 8.44 ppm for Ir and 7.81 ppm for the Ru moiety) suggest presence of two nonequivalent 2-pyridonato ligands. The chemical shifts fall within the range defined by neutral complex **3** (δ 8.33) and ionic **1** (δ 9.18).

The FT-IR spectroscopy of **5** and **6** revealed a shift of the stretching bands to the 1487–1490 cm^–1^ region relative to the free **3** due the weakening of the C=O bond due to the C=O → Zn coordination.

Single crystals of **6** were grown in MeOH at RT and is depicted in Figure 3 (for crystallographic details, see Table S1). The molecular structure of **6** proved the coordination of two nonequivalent C=O groups to ZnCl_2_. Consequently, both C1–O1 and C33–O2 bond distances (1.273(3) Å) in **6** are elongated compared to **3** (1.243(4) Å). Therefore, the **5** can be used as a precursor for the synthesis of heterotrimetallic complexes.

In addition, while cyclometalated Ir­(III) complexes are well-known a class of luminescent compounds with diverse applications (particularly in biological imaging, photodynamic therapy, and sensing)^18–22^ their half-sandwich analogues have been rarely explored in this field.^23–25^ The UV/vis absorption spectrum of [Ir­(Cp*)­Cl­(2-{(Dipp)­N=CH}–C_5_H_3_N–6-(O))] (**3**) in DCM displays a broad lowest energy absorption band between 380 and 550 nm with λ_max_ = 450 nm (ε = 10,000 M^–1^ cm^–1^) and a weaker shoulder at ca. 490 nm (ε = 6000 M^–1^ cm^–1^) (Figure 4), which correspond to the intense dark orange color of the solution. The spectral appearance and medium intensity suggest that those bands are charge transfer (CT) in nature, and indeed our TD-DFT calculations (see Table S2) reveal that the low-energy S_0_ → S_1_/S_2_ transitions originate from IrCl → iminopyridonate metal-to-ligand CT and Cp* → iminopyridonate ligand-to-ligand CT (MLCT/LLCT), facilitated by the redox-active nature of both the ligand and the Ir­(III) center.^26–29^ These transitions are further mixed with intraligand CT (ILCT), involving the electron-rich C=O group as a donor of electron density. Noteworthy, the different Ir­(d)/Cl­(p) orbital combinations as part of HOMO and HOMO–1 give rise to very similar CT states, but for symmetry and orbital overlap reasons slightly different energies and oscillator strengths *f* are observed (see also Supporting Information). A slightly less intense band between 300 and 380 nm with λ_max_ = 345 nm (ε_max_ = 6,500 M^–1^ cm^–1^) is due to several overlapping transitions of pyridonate/Ir → Cp* and IrCl/CO → py CT type, while higher energy transitions in the UV region can be tentatively assigned to ligand-centered absorptions.

**Figure 4:**
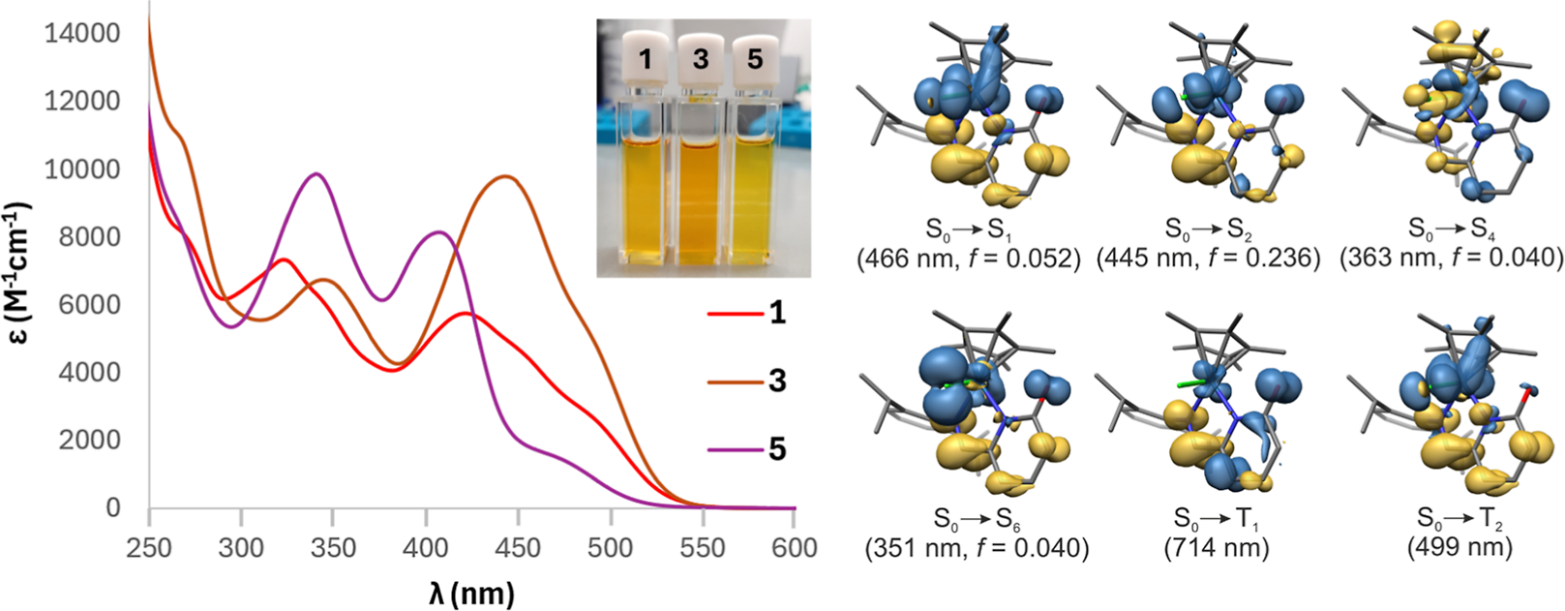
Left: absorption spectra of **1**, **3**, and **5** in DCM, and photograph of the corresponding solutions (concentration 100 μmol/L). Right: TD-DFT calculated (D3BJ-PBE0, ZORA, def2-SVP) electron density differences for selected vertical allowed S_0_ → S_n_ transitions of **1** relevant for interpretation of the experimental absorption spectra, and depiction of the T_1_ and T_2_ states at the ground-state geometry (loss of electron density colored in blue, gain colored in yellow; for further details, see Supporting Information).

Using complex **3** as a well-analyzed benchmark, the electronic structure effects on the absorption spectra of cationic [(Cp*)­IrCl­(κ^2^-L)]^+^ (**1**) and the ZnCl_2_ adduct **5** can be understood. While the energetic positions of the lowest energy absorption band of **1** shift only marginally due to the cationic charge, the absorption coefficients are significantly reduced because of the OMe moiety at the pyridine ring, diminishing the orbital contribution of the oxygen and thus reducing the *f*-enhancing contribution of the ILCT character. It is possible that rotation around the C­(py)–OMe bond leads to different conformations in solution, of which the orbital overlap of the oxygen lone pair with the π* system of the ligand changes in dependence of the rotational angle. Consequently, a higher number of excited states with different absorption coefficients should be observed as shoulders, as shown in Figure 4. In contrast, coordination of Lewis acidic ZnCl_2_ to the carbonyl moiety in **5** has a severe influence on the absorption spectrum as also displayed by its yellow color due to the resulting stabilization of the O­(*n*) electrons. A broad CT band of very low ε of ca. 2,000 M^–1^ cm^–1^ between 450 and 520 nm is observed presumably due to IrCl → iminopyridonate (MX)­LCT, similar to the T_2_ state (Figure 4). An intense band with λ_max_ = 410 nm (ε_max_ = 8,000 M^–1^ cm^–1^) can be assigned to (MX)­LCT/ILCT states as described for the original S_1_/S_2_ states of complex **3** but hypsochromically shifted due to the Zn^2+^ coordination. Interestingly, the presence of O atom in any form at the *o*-position of the pyridine ring induces a significant red-shift of the low-energy absorption bands compared to the unsubstituted analogue [(η^5^-Cp*)­IrCl­{κ^2^-{2-[(2,6-*i*Pr_2_–C_6_H_3_)­N=CH]–C_5_H_3_N}­(PF_6_).^30^ The significant role of the C–O group in electronic transitions motivated us to explore the light-induced demethylation of ionic complex **1** as an alternative to the thermally initiated pathway. Complex **1** was irradiated with a 467 nm lamp either in DCM solution or in the solid state for 12 h. Unfortunately, neither compound **3** nor any other product was detected by ^1^H NMR spectrum (Figure S99, for detailed photophysical studies of **1**, **3**, and **5**, see Figures S76–S98 in Supporting Information).

### C­(sp^3^)–X Bond Activation Mediated by Complex **3**

The presence of an electron-rich C=O bond in complex **3** prompted us to explore C–X bond activation mediated by the 2-pyridone complex **3**. Several organic substrates RCH_2_X (R is H, C_5_H_10_I, Ph, CO*t*Bu, CN, COOMe, and X is I or Br) were tested. Complex **3** was added into neat organic substrates, and resulting solutions/suspensions were stirred for 16 h. The reaction mixture was washed by hexane or Et_2_O to remove an excess of the organic substrate, and resulting solids were characterized as complexes [(η^5^ -Cp*)­IrX­(2-{(2,6-*i*Pr_2_–C_6_H_3_)­N=CH}–C_5_H_3_N–6-(OCH_2_R)]­(X) (**2**: R = H, X = I; **7**: R = C_5_H_10_I, X = I; **8**: R = Ph, X = Br; **9**: R = CO*t*Bu, X = Br; **10**: R = CN, X = Br; **11**: R = COOMe, X = Br) (see Figure 5). If the reactions were done in the stoichiometric manner in DCM solution, low yields were obtained only. Notably, complexes **3** and **4** are indefinitely stable in chlorinated solvents (DCM and CHCl_3_/CDCl_3_), even under elevated temperatures. Furthermore, other types of substrates were tested under identical neat conditions. Unfortunately, the reaction of **3** with *i*PrI (as an example of secondary halide), CH_2_I_2_, CH_2_ICl (α-dihalides), PhI (aromatic halide), or ClCH_2_COCl (lighter alkyl halides) did not afford the *O*-alkylated products.

**Figure 5:**
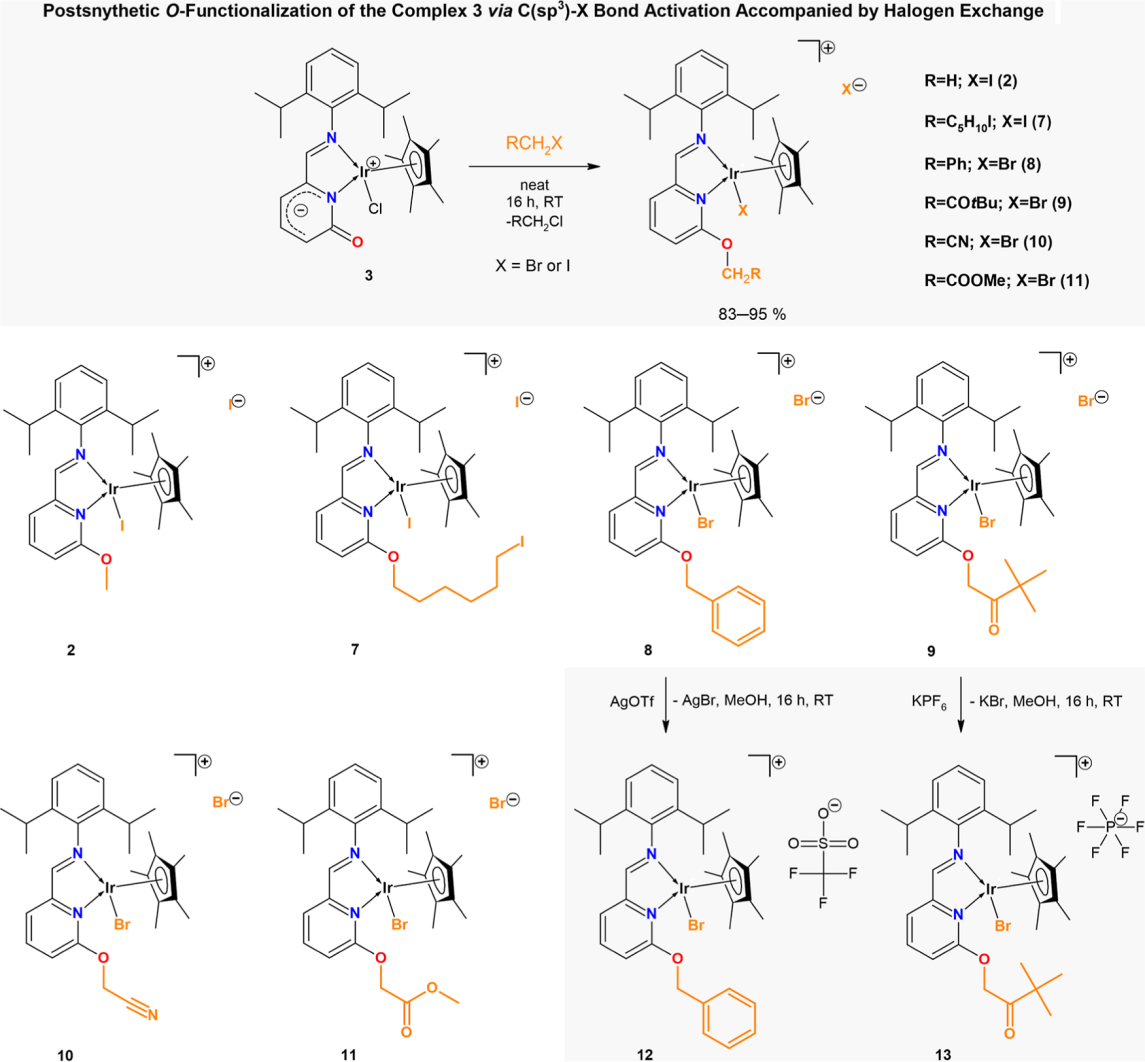
Portfolio of the O-substituted Ir­(III) complexes prepared via C­(sp^3^)–X bond activation of substituted alkyl halides with **3** under neat conditions.

New complexes, products of RCH_2_–X bond activation, were characterized using NMR, FT-IR, and UV/vis spectroscopies and MS-spectrometry (Figures S13–S23, S32–S41, S48–S54, and S58).

In the ^1^H NMR spectra, C*H*=N signals of **7**–**11** were observed in the region typical for ionic Ir­(III) complexes **1**–**2**, ranging from δ 8.83 to δ 9.16 ppm (Figure 6).

**Figure 6:**
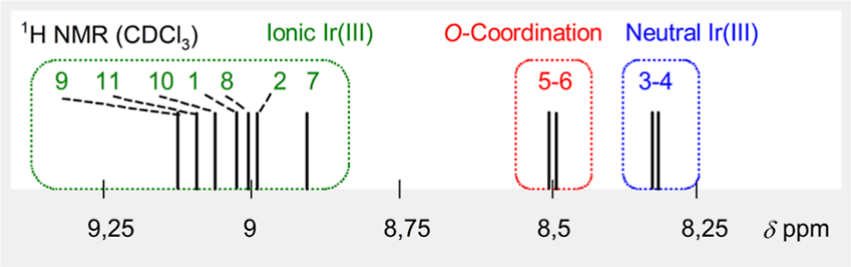
Stick ^1^H NMR diagram showing the C*H*=N chemical shift as a powerful tool for classifying complexes **1**–**11** into each structural class.

Most importantly, new AB or AX spin systems of the newly formed OC*H*_2_R groups were observed in the ^1^H NMR spectra of **7**–**11** (range of δ 4.49 to δ 6.00 ppm). These signals were absent in the ^1^H NMR spectrum of **3**, and they are also shifted downfield as compared to the singlet resonances of the parent RC*H*_2_X substrates. The ^13^C­{^1^H} NMR spectra of **7**–**11** absent the signal at δ 167.7 ppm, typical for free C=O group in **3**. Similarly, FT-IR spectra of **7**–**11** missed the strong absorptions of the free C=O group in **3**. In addition, FT-IR spectra of **9** and **11** revealed characteristic absorptions for the carbonyl group at ν_(CO)_ = 1720 cm^–1^ and for ester group at ν_(CO)_ = 1750 cm^–1^ due to formation of new OC*H*_2_R moieties. Therefore, the NMR and IR data suggest that complex **3** is suitable for the activation of RC*H*_2_X (X = Br or I) substrates. These ionic compounds were also characterized by the HR MALDI MS (Figures S32–S41). Most importantly, the mass values of molecular ions in the positive-ion part correspond to the [M]^+^ cations with [Ir–I]^+^ or [Ir–Br]^+^ bonds instead of original [Ir–Cl]^+^. This suggests that, in addition to the C–X bond activation, a substitution reaction of the Ir–Cl bond also occurs. The latter is well-known and has already been reported in the literature.^31,32^

This substitution of the Ir–Cl bond in **3** was further confirmed experimentally. As suggested, the reaction of **3** with MeI provided ionic compound **2**, that was independently prepared by the reaction of ligand **L** with [(η^5^ -Cp*)­IrI_2_]_2_ (see Scheme 1). The reaction of **3** with 4 eq. of BrCH_2_CO*t*Bu was also monitored by the ^1^H NMR spectroscopy, and the reaction mixture showed the signal at δ 4.22 ppm of the starting BrC*H*_2_ *C*O*t*Bu together with a new signal at δ 4.42 ppm of ClC*H*_2_CO*t*Bu formed due to the substitution reaction (Figures S66 and S69). All O-substituted compounds **2** and **7**–**11** were also characterized by the UV/vis spectroscopy. Interestingly, the absorption spectra of ionic products **2** and **7**–**11** showed marginal influence of the O-substituent on absorption maxima (see Figure S58).

Since the attempts to obtain single crystals of **2**, **7**–**11** were unsuccessful, additional substitutions reactions of Br counteranions in **8** and **9** were performed. Reactions of **8** and **9** with AgOTf or KPF_6_ were performed and provided new compounds **12** and **13**, that were characterized by NMR and FT-IR spectroscopies (Figures S24–S29, S50, and S52) and by SC-XRD. All the data correlate well with those found in the starting ionic compounds **8** and **9**, therefore the most importantly, molecular structures of **12** and **13** clearly showed alkyl halides activation mediated by Ir­(III) 2-pyridonato complex **3**. Single crystals of **12** and **13** were grown in THF and are depicted in Figure 7 (for crystallographic details, see Table S1).

**Figure 7:**
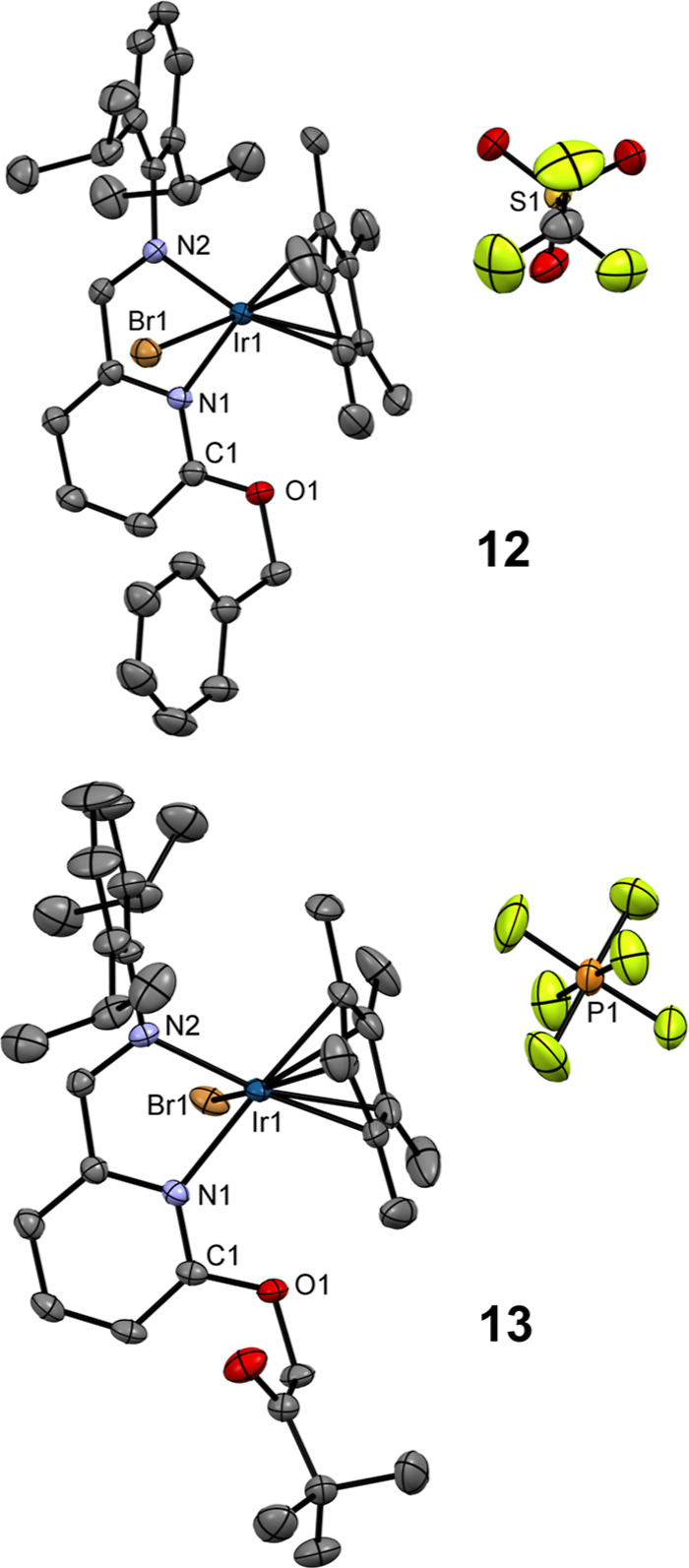
ORTEPs of **12** and **13**. Non-H atoms are represented by 50% probability ellipsoids. H atoms and THF molecules are omitted for clarity. For **12**: bond lengths (Å): Ir1–N1 2.129(2); Ir1–N2 2.113(2); Ir1–Br1 2.5280(6); C1–O1 1.335(4). Bond angles (°): N1–Ir1–N2 76.06(9); N1–Ir1–Br1 80.09(7); N2–Ir1–Br1 89.42(7); N1–C5–O1 113.7(3). For **13**: bond lengths (Å): Ir1–N1 2.138(1); Ir1–N2 2.112(2); Ir1–Br1 2.5465(5); C1–O1 1.338(2). Bond angles (°): N1–Ir1–N2 76.65(6); N1–Ir1–Br1 79.88(4); N2–Ir1–Br1 89.10(4); N1–C1–O1 114.3(1).

The molecular structures revealed that the substitution of the Ir–Cl bond took place, and new Ir1–Br1 bonds with distance of 2.5280(6) Å (**12**) and 2.5465(5) Å (**13**) are formed. In addition, new O1–C bonds with distance of 1.451(4) Å (**12**) and 1.441(3) (**13**) are present, as the result of activation of organic substrates PhCH_2_Br (**12**) and *t*BuCOCH_2_Br (**13**). This process is accompanied by the disruption of the 2-pyridonato ligand arrangement, which is manifested by the significant elongation of the C1–O1 bond from 1.243(4) Å in **3** to 1.335(4) Å (**12**) and 1.338(2) Å (**13**).

As the reaction of **3** with RC*H*_2_X alkyl halides proceeds as both an *O*-functionalization and substitution reaction, we also run additional kinetic studies of two model reactions (Figure 8).

**Figure 8:**
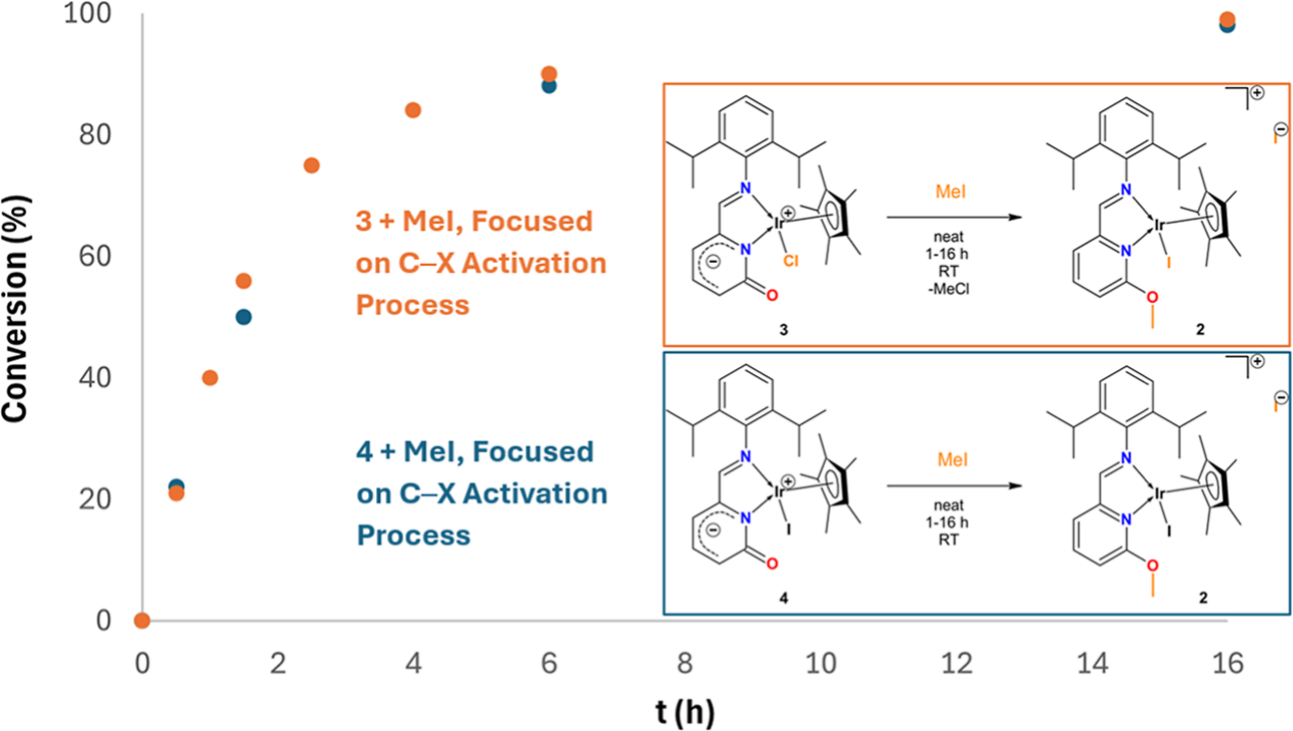
Reactions of MeI with **3** (orange) and **4** (blue), where the side reaction of the halide substitution is suppressed. Monitored by the C*H*=N imine proton shift in ^1^H NMR.

The reaction of neutral complex **4** with MeI (conditions as described above) was monitored by ^1^H NMR spectrum (Figure S68). Since the neutral complex **4** contains both free C=O group and an Ir–I bond, this reaction was expected to proceed predominantly via C–X bond activation of MeI to yield ionic complex **2**, suppressing the halide substitution. The conversion of neutral compound **4** to ionic **2** was monitored by the ^1^H NMR spectrum, by the integration ratio of the C*H*=N imine groups of the reaction mixture. According to ^1^H NMR data, this activation of MeI was completed within 16 h (Figure 8). Second, the reaction of neutral **3** with MeI (conditions as described above) was also monitored by ^1^H NMR (Figure S67). This reaction also provided ionic compound **2**, therefore, the halide substitution reaction of the Ir–Cl bond is involved. The conversion of neutral compound **3** to ionic **2** was monitored by the ^1^H NMR spectrum, by the integration ratio of the C*H*=N imine groups of the reaction mixture. According to the ^1^H NMR spectroscopy, this reaction was also completed within 16 h yielding complex **2** as the final product. Thus, both reactions revealed similar kinetical trend indicating that halide substitution does not affect the O-functionalization pathway (Figure 8).

The reaction of MeI with **3** was also monitored by UV/vis analysis since the UV/vis spectra of **2**–**4** are mutually different (Figure 9). Therefore, absorption spectrum of the reaction mixture of **3** with MeI was recorded after 1 h, simultaneously with the ^1^H NMR experiment, where the presence of ionic **2** and neutral moieties **3** or **4** (indistinguishable by ^1^H) was detected in a 40:60 ratio. The experimental data (black solid curve in Figure 9) closely matched the simulated absorption spectrum of **2** and **4** (red dots curve in Figure 9) in a 40:60 ratio, detected by the ^1^H NMR experiment (see Figures S67 and S70). This finding suggests the exclusive presence of Ir–I species, e.g., ionic complex **2** and neutral compound **4**, the products of the substitution reaction. These findings thus indicate that substitution of the Ir–Cl bond in **3** proceeds more rapidly than C–X bond activation.

**Figure 9:**
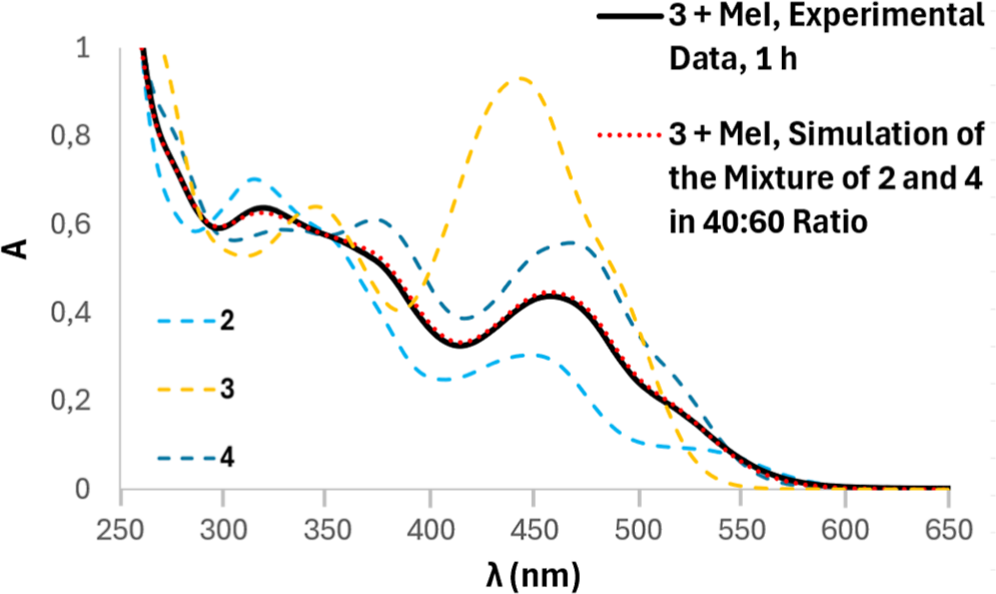
Absorption spectra of the reaction mixture of **3** with MeI (black solid) recorded after 1 h. A simulated absorption spectrum (red dots) of the mixture of **2** and **4** in the 40:60 ratio (detected by ^1^H NMR) obtained from the experimental data of **2** and **4**: *A*_simulated_ = 0.4·A­(**2**) + 0.6·A­(**4**) closely matches the experimental spectrum of the mixture and confirms the absence of the starting compound **3** after 1 h. The UV/vis spectra of isolated pure compounds **2** (light blue), **3** (yellow), **4** (dark blue).

To gain deeper insight into the *O*-alkylation mechanism, we focused on the preparation of a halide-free complex. The treatment of **3** with AgOTf afforded a mixture of unseparated compounds, but the substitution reaction of **3** with K­[Ag­(CN)_2_] provided halide-free complex [(η^5^ -Cp*)­Ir­(CN)­(2-{(2,6-*i*Pr_2_–C_6_H_3_)­N=CH}–C_5_H_3_N–6-(O))] (**14**). Yellow complex **14** was characterized by the help of NMR, FT-IR, and UV/vis spectroscopies (Figures S30, S31, S55, and S59).

The ^1^H and ^13^C­{^1^H} NMR spectra of **14** are comparable to **3** or **4**. The ^1^H NMR spectroscopy showed the signal of the C*H*=N proton resonating at δ 8.28 similarly to starting **3**. The presence of the Ir­(III) 2-pyridonato complex **14** with C=O group is demonstrated by the signal at δ 168.7 ppm in the ^13^C­{^1^H} NMR spectra of **14**. The presence of the Ir–CN group was confirmed by the characteristic signal at δ 116.0 ppm in the ^13^C­{^1^H} NMR spectrum, as well as by a strong vibrational band with a maximum at 2121 cm^–1^ in the FT-IR spectrum of **14**.

Finally, the reaction of **14** with MeI afforded a mixture of starting **14** and MeI, together with signals suggesting the formation of new ionic O-substituted compound [(η^5^ -Cp*)­Ir­(CN)­(κ^2^-{2-[(2,6-*i*Pr_2_–C_6_H_3_)­N=CH]-6-(OMe)­C_5_H_3_N})]­(I) (**15**) (Scheme 3). In the ^1^H NMR spectrum (Figure S71), two signals of the C*H*=N groups were found at δ 8.24 ppm and at δ 8.93 ppm. While the former signal corresponds to starting **14**, downfield shifted signal (δ 8.93) falls to the range typical for ionic complexes (Figure 6). Moreover, the successful methylation is evidenced by the signal at δ 4.34 ppm, the value typical for the OMe group in the ^1^H NMR spectrum. The successful O-substitution and the formation of new O*Me* bond was confirmed by the ^13^C­{^1^H} NMR spectrum (Figure S72), where the signal of the O*Me* group at δ 59.2 ppm was observed. Importantly, the presence of two signals of the Ir–*CN* groups at δ 113.2 and δ 115.8 ppm (**14**) indicates that the Ir–*CN* bond remains preserved during the reaction (Figure S72). These observations suggest that MeI is cleaved by the C=O group via an SN2-type reaction, in which the O atom is methylated while iodide is released as the counterion compensating the formed cation. In contrast, a similar reaction of *N*-methyl-2-pyridone with MeI under same conditions did not provide any *O*-alkylation product and only starting *N*-methyl-2-pyridone was detected in ^1^H NMR spectrum (Figure S73). This suggests an important role of the Ir center and proves that these *O*-alkylations are metal–ligand cooperation processes. For completeness, the thermally induced reverse dealkylation of two ionic compounds (**10** vs **13**) with different counteranions (Br vs PF_6_^–^) was also investigated. However, compound **10** containing the Br^–^ counteranion afforded a new neutral compound [(η^5^ -Cp*)­IrBr­(2-{(2,6-*i*Pr_2_–C_6_H_3_)­N=CH}–C_5_H_3_N–6-(O))] upon heating in the solid state (Figure S74) along with the elimination of CNCH_2_Br, compound **13** remains stable and does not undergo the elimination reaction (Figure S75). This observation indicates that while the halide counteranions serve as the source for the alkyl halides, the Ir-halide unit does not interact with the C–O-alkyl group on the ligand during the reverse elimination.

**Scheme 3:**
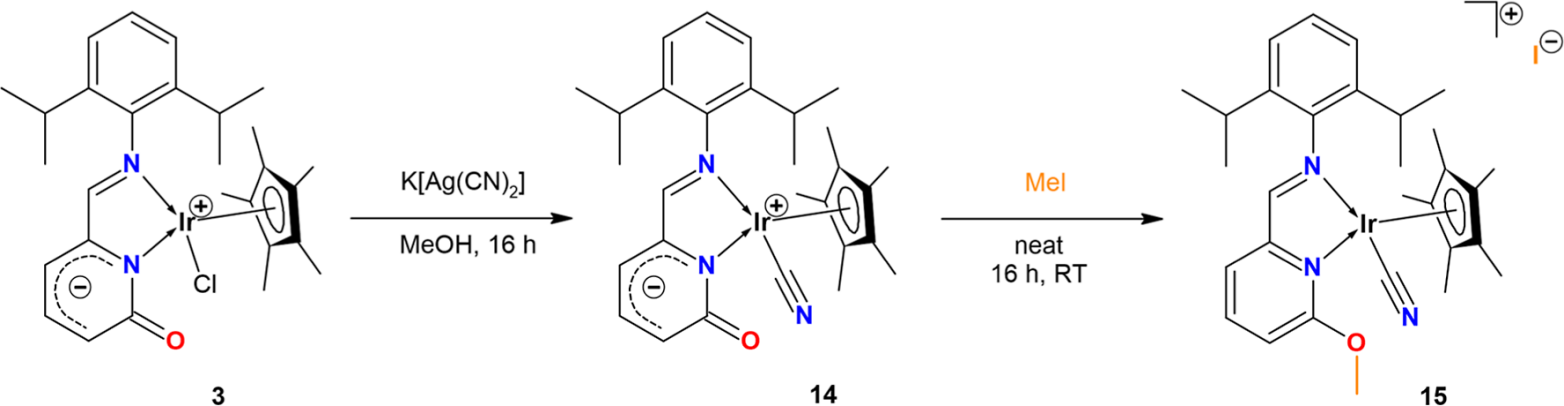
Preparation of Halide-Free Complex **14** as a Precursor for *O*-Alkylation and Dealkylation Studies

## Conclusion

In this work, we have introduced an efficient methodology for the preparation of a new series of 2-pyridonato-based Ir­(III) half-sandwich complexes [(η^5^ -Cp*)­IrX­(2-{(2,6-*i*Pr_2_–C_6_H_3_)N = CH}–C_5_H_3_N–6-(O))] (**3**: X = Cl; **4**: X = I). These complexes were prepared via thermally initiated demethylation of the corresponding ionic complexes [(η^5^ -Cp*)­IrX­(κ^2^-{2-[(2,6-*i*Pr_2_–C_6_H_3_)­N=CH]-6-(OMe)­C_5_H_3_N})]­(X) (**1**: X = Cl; **2**: X = I) in the solid state in quantitative yields and high purity. Neutral complexes **3** and **4** were further studied as versatile precursors for the preparation of unprecedented heterobimetallic Ir–Zn complex **5** stabilized by the additional MeOH ligand forming an intramolecular hydrogen bond as well as heterotrimetallic Ir–Zn–Ru complex **6**. Remarkably, complexes **3** and **4** were capable of C­(sp^3^)–X bond activations, leading to the isolation of several O-substituted ionic products. This approach introduced various functional groups such as iodide, aryl, ketone, nitrile, or ester into structure of ionic complexes **7**–**13**. Alongside this activation, substitution of the Ir–Cl bond by Ir–X (X = Br or I) was also observed during this reaction and both processes were investigated in detail by NMR and UV/vis study to provide mechanistic insight. The halide-free complex [(η^5^ -Cp*)­Ir­(CN)­(2-{(2,6-*i*Pr_2_–C_6_H_3_)­N=CH}–C_5_H_3_N–6-(O))] (**14**) was also prepared and treated with MeI. This reaction also provided new ionic product containing new O*Me* and suggests that the reactions of 2-pyridonato-based Ir­(III) half-sandwich complexes with alkyl halides proceed as the SN2 reactions. However, since the free *N*-methyl-2-pyridone does not react with MeI under the same condition, these *O*-alkylations are metal–ligand cooperation processes.

## Experimental Section

The preparations of all the complexes mentioned were carried out in an inert atmosphere of argon (air products, 99.999%) using the Schlenk flask technique using septa and cannulas. Solvents were dried by standard methods and distilled prior to use. The deuterated solvents were purchased from GenChem and dried over 4 Å molecular sieves. No uncommon hazards are noted.

### Physical Measurement

^1^H, ^13^C­{^1^H}, ^19^F­{^1^H}, and ^31^P­{^1^H} NMR spectra were recorded on a Bruker Avance 400 and 500 MHz NMR spectrometer at 298 K. The ^1^H and ^13^C­{^1^H} NMR spectra were referenced internally to residual proton-solvent and solvent resonances, respectively, and are reported relative to Me_4_Si (δ = 0 ppm). FT-IR spectroscopy was measured on a Nicolet iS50 FT-IR spectrometer (Thermo Scientific) in the range 400–4000 cm^–1^ in the solid phase using the ATR (diamond crystal) technique. The melting or decomposition temperature of complexes was determined on a Stuart MP3 thermometer in a glass capillary. Elemental analyses were performed on a LECO–CHNS-932 analyzer. High-resolution MALDI MS spectra were measured with a MALDI mass spectrometer LTQ Orbitrap XL (Thermo Fisher Scientific, Bremen, Germany) equipped with nitrogen UV laser (337 nm, 60 Hz). The LTQ Orbitrap instrument was operated in the positive-ion mode over a normal mass range (*m*/*z* 50–2000) with resolution 100 000 at *m*/*z* = 400. The survey crystal positioning system (survey CPS) was set for the random choice of shot position by automatic crystal recognition. *Trans*-2-[3-(4-*tert*Butylphenyl)-2-methyl-2-propenylidene]­malononitrile (DCTB) was used as a matrix. Mass spectra were averaged over the whole MS record for all measured samples. UV/vis spectra were recorded on an UV-1600PC spectrophotometer in the quartz cuvette in the range 1100–200 nm. Excitation and emission spectra were recorded at right angles to the excitation source on an Edinburgh Instrument FLS1000, equipped with a 450 W xenon arc lamp, double monochromators for the excitation and emission pathways, and a red-sensitive photomultiplier (PMT-980) as the detector. The luminescence lifetimes were measured using either a 450 nm VPL with a repetition rate 100 kHz (depending on the time range) or a multichannel scaling (MCS). Quantum yields were recorded using a quantaurus-QY absolute PL quantum yield spectrometer C11347 by Hamamatsu.

### Chemical and Reagents

6-Methoxy-pyridine-2-carboxaldehyde, 2,6-diisopropylaniline, [(η^5^ -Cp*)­IrCl_2_]_2_, [(η^5^ -Cp*)­IrI_2_]_2,_ ZnCl_2_, MeI, I­(CH_2_)_6_I, PhCH_2_Br, *t*BuCOCH_2_Br, NCCH_2_Br, MeCO_2_CH_2_Br, K­[Ag­(CN)_2_], *N*-methyl-2-pyridone, AgOTf, and KPF_6_ procured from Sigma-Aldrich (USA) were used as received. Ligand **L** and complex [(η^6^ *-p-cymene*)­RuCl­(2-{(2,6-*i*Pr_2_–C_6_H_3_)N = CH}–C_5_H_3_N–6-(O))] were prepared according to the literature procedure.^10,33^

### Synthesis of **1**

**L** (350 mg, 1.2 mmol) was dissolved in MeOH (5 mL), and [(η^5^ -Cp*)­IrCl_2_]_2_ (470 mg, 0.59 mmol) was added. The Ir precursor dissolved in few minutes, and the red solution was heated to 110 °C and refluxed under an inert argon atmosphere for 4 h. Then, the solvent was evaporated under reduced pressure, the residue was washed with hexane, THF, and hexane again and dried. Orange fine powder was characterized as **1**. **Yield**: 200 mg (95%). Anal. Calcd for **C**_**29**_ **H**_**39**_ **IrCl**_**2**_ **N**_**2**_ **O** (**M**_**w**_ = **694.76**) C, 50.1; H, 5.7. Found: C, 50.3; H, 5.9. **Mp**: thermally unstable. ^**1**^ **H NMR** (CDCl_3_, 500.13 MHz): δ (ppm) 0.87 (d, 3H, CH_3_(dipp), ^3^ *J*(^1^H, ^1^H) = 6.4 Hz), 1.20 (d, 3H, CH_3_(dipp), ^3^ *J*(^1^H, ^1^H) = 6.8 Hz), 1.24 (d, 3H, CH_3_(dipp), ^3^ *J*(^1^H, ^1^H) = 6.8 Hz), 1.32 (d, 3H, CH_3_(dipp), ^3^ *J*(^1^H, ^1^H) = 6.8 Hz), 1.46 (s, 15H, CH_3_–Cp*), 2.42 (sept, 1H, CH­(dipp), ^3^ *J*(^1^H, ^1^H) = 6.8 Hz), 3.71 (sept, 1H, CH­(dipp), ^3^ *J*(^1^H, ^1^H) = 6.8 Hz), 4.32 (s, 3H, CH_3_O), 7.25 (d, 1H, Ar–H, ^3^ *J*(^1^H, ^1^H) = 7.8 Hz), 7.29 (d, 1H, Ar–H, ^3^ *J*(^1^H, ^1^H) = 7.8 Hz), 7.34 (t, 1H, Ar–H, ^3^ *J*(^1^H, ^1^H) = 7.8 Hz), 7.83 (d, 1H, Ar–H, ^3^ *J*(^1^H, ^1^H) = 8.3 Hz), 8.25–8.29 (m, 2H, Ar–H), 9.18 (s, 1H, CH = N). ^**13**^ **C­{** ^**1**^ **H} NMR** (CDCl_3_, 125.613): δ (ppm) 9.63 (CH_3_–Cp*), 21.6; 24.1; 26.2 (CH_3_(dipp)), 27.6; 28.0 (CH­(dipp)), 28.1 (CH_3_(dipp)), 58.8 (CH_3_O), 91.24 (C-Cp*), 113.9; 124.4; 124.6; 124.7; 129.7 (Ar–CH), 141.5; 143.0; 144.2 (Ar–C), 144.7 (Ar–CH), 152.7; 164.6 (Ar–C), 173.9 (CH = N). **FT-IR** (ATR): ν 1617 (m, ν C=N) cm^–1^.

### Synthesis of **2**

**L** (250 mg, 0.84 mmol) was dissolved in MeOH (5 mL), and [(η^5^ -Cp*)­IrI_2_]_2_ (490 mg, 0.42 mmol) was added. The Ir precursor did not dissolve completely during the reaction. The red-brown suspension and solution were heated to 110 °C and refluxed under an inert argon atmosphere for 6 h. Then, the solution was filtered from the unreacted Ir precursor, solvent was evaporated under reduced pressure, the residue was washed with hexane, THF, and hexane again. Red-brown powdery material was characterized as **2**. **Yield**: 200 mg (95%). Anal. Calcd for **C**_**29**_ **H**_**39**_ **IrI**_**2**_ **N**_**2**_ **O** (**M**_**w**_ = **877.67**); C, 39.7; H, 4.5. Found: C, 39.9; H, 4.6. **Mp**: thermally unstable. ^**1**^ **H NMR** (CDCl_3_, 500.13 MHz): δ (ppm) 0.82 (d, 3H, CH_3_(dipp), ^3^ *J*(^1^H, ^1^H) = 6.6 Hz), 1.18 (d, 3H, CH_3_(dipp), ^3^ *J*(^1^H, ^1^H) = 6.5 Hz), 1.22 (d, 3H, CH_3_(dipp), ^3^ *J*(^1^H, ^1^H) = 6.7 Hz), 1.31 (d, 3H, CH_3_(dipp), ^3^ *J*(^1^H, ^1^H) = 6.6 Hz), 1.52 (s, 15H, CH_3_–Cp*), 2.41 (sept, 1H, CH­(dipp), ^3^ *J*(^1^H, ^1^H) = 6.7 Hz), 3.93 (sept, 1H, CH­(dipp), ^3^ *J*(^1^H, ^1^H) = 6.7 Hz), 4.31 (s, 3H, CH_3_O), 7.23 (d, 1H, Ar–H, ^3^ *J*(^1^H, ^1^H) = 7.8 Hz), 7.28–7.35 (m, 2H, Ar–H), 7.65 (d, 1H, Ar–H, ^3^ *J*(^1^H, ^1^H) = 8.7 Hz), 8.09 (d, 1H, Ar–H, ^3^ *J*(^1^H, ^1^H) = 7.8 Hz), 8.17 (t, 1H, Ar–H, ^3^ *J*(^1^H, ^1^H) = 7.8 Hz), 8.87 (s, 1H, CH=N). ^**13**^ **C­{** ^**1**^ **H} NMR** (CDCl_3_, 125.613): δ (ppm) 10.8 (CH_3_–Cp*), 21.1; 24.3; 26.0 (CH_3_(dipp)), 27.9; 28.4 (CH­(dipp)), 29.5 (CH_3_(dipp)), 59.0 (CH_3_O), 91.8 (C-Cp*), 112.9; 124.3; 124.4; 124.7; 129.7 (Ar–CH), 141.5; 142.7 (Ar–C), 143.4 (Ar–CH), 144.8; 152.7; 165.6 (Ar–C), 172.2 (CH = N). **FT-IR** (ATR): ν 1615 (m, ν C=N) cm^–1^. **HRMS** (MALDI): *m*/*z* calcd for C_29_H_39_^193^IrIN_2_O, 751.17308 [M]^+^; found, 751.17197; calcd for C_29_H_39_^193^IrN_2_O: 624.26861 [M – I]^+^; found, 624.26858; *m*/*z* calcd for I: [I]^−^ 126.90502; found, 126.90985.

### Synthesis of **3**

Complex **1** (400 mg) was heated in a solid state in a flask open to air for 2 h at 120 °C to give a brick red fine powder characterized as **3**. **Yield**: 371 mg (100%). Anal. Calcd for **C**_**28**_ **H**_**36**_ **IrClN**_**2**_ **O** (**M**_**w**_ = **644.28**) C, 52.2; H, 5.6. Found: C, 52.1; H, 5.6. **Mp**: 260 °C with decomp. ^**1**^ **H NMR** (CDCl_3_, 500.13 MHz): δ (ppm) 0.90 (d, 3H, CH_3_(dipp), ^3^ *J*(^1^H, ^1^H) = 6.7 Hz), 1.12 (d, 3H, CH_3_(dipp), ^3^ *J*(^1^H, ^1^H) = 6.7 Hz), 1.24 (d, 3H, CH_3_(dipp), ^3^ *J*(^1^H, ^1^H) = 6.7 Hz), 1.29 (d, 3H, CH_3_(dipp), ^3^ *J*(^1^H, ^1^H) = 6.7 Hz), 1.46 (s, 15H, CH_3_–Cp*), 2.58 (sept, 1H, CH­(dipp), ^3^ *J*(^1^H, ^1^H) = 6.8 Hz), 3.99 (sept, 1H, CH­(dipp), ^3^ *J*(^1^H, ^1^H) = 6.7 Hz), 6.55–6.58 (m, 2H, Ar–H), 7.17–7.26 (m, 4H, Ar–H), 8.33 (s, 1H, CH=N). ^**13**^ **C­{** ^**1**^ **H} NMR** (CDCl_3_, 125.613): δ (ppm) 9.7 (CH_3_–Cp*), 21.6; 24.1; 26.3 (CH_3_(dipp)), 27.4; 27.7 (CH­(dipp)), 27.9 (CH_3_(dipp)), 89.2 (C-Cp*), 115.2; 124.0; 124.1; 124.8; 128.4; 135.5 (Ar–CH), 141.7; 143.9; 145.5; 152.2 (Ar–C), 167.7 (Ar–C=O), 174.5 (CH=N). **FT-IR** (ATR): ν 1556 (s, ν (C=O); ν 1620 (m, ν C=N) cm^–1^.

### Synthesis of **4**

Complex **2** (250 mg) was heated in a solid state in a flask open to air for 2 h at 120 °C to give a brown-red powdery material characterized as **4**. **Yield**: 210 mg (100%). Anal. Calcd for **C**_**28**_ **H**_**36**_ **IrIN**_**2**_ **O** (**M**_**w**_ = **735.73**) C, 45.7; H, 4.9. Found: C, 45.9; H, 4.8. **Mp**: 258 °C. ^**1**^ **H NMR** (CDCl_3_, 500.13 MHz): δ (ppm) 0.86 (d, 3H, CH_3_(dipp), ^3^ *J*(^1^H, ^1^H) = 6.6 Hz), 1.12 (d, 3H, CH_3_(dipp), ^3^ *J*(^1^H, ^1^H) = 6.6 Hz), 1.23 (d, 3H, CH_3_(dipp), ^3^ *J*(^1^H, ^1^H) = 6.7 Hz), 1.31 (d, 3H, CH_3_(dipp), ^3^ *J*(^1^H, ^1^H) = 6.6 Hz), 1.58 (s, 15H, CH_3_–Cp*), 2.54 (sept, 1H, CH­(dipp), ^3^ *J*(^1^H, ^1^H) = 6.7 Hz), 4.19 (sept, 1H, CH­(dipp), ^3^ *J*(^1^H, ^1^H) = 6.6 Hz), 6.51 (d, 1H, Ar–H, ^3^ *J*(^1^H, ^1^H) = 8.7 Hz), 6.67 (d, 1H, Ar–H, ^3^ *J*(^1^H, ^1^H) = 6.5 Hz), 7.21–7.27 (m, 4H, Ar–H), 8.28 (s, 1H, CH = N). ^**13**^ **C­{** ^**1**^ **H} NMR** (CDCl_3_, 125.613): δ (ppm) 10.6 (CH_3_–Cp*), 21.1; 24.2; 26.1 (CH_3_(dipp)), 27.6; 28.0 (CH­(dipp)), 29.2 (CH_3_(dipp)), 89.6 (C-Cp*), 115.9; 123.2; 124.1; 124.2; 128.5; 135.5 (Ar–CH), 141.6; 143.6; 145.5; 152.5 (Ar–C), 168.3 (Ar–C=O), 173.9 (CH=N). **FT-IR** (ATR): ν 1548 (s, ν (C=O); ν 1618 (m, ν C=N) cm^–1^.

### Synthesis of **5**

MeOH solution (2 mL) of ZnCl_2_ (32 mg, 0.23 mmol) was added to the MeOH solution (3 mL) of **3** (150 mg; 0.23 mmol) at the RT, and the orange mixture was stirred for 16 h. Then, the solvent was evaporated under reduced pressure and yellow powder was washed with hexane 3 times and dried. Yellow powdery material was characterized as **5**. **Yield**: 180 mg (96%). Anal. Calcd for **C**_**29**_ **H**_**40**_ **IrZnCl**_**3**_ **N**_**2**_ **O**_**2**_ (**M**_**w**_ = **812.60**) C, 42.9; H, 5.0. Found: C, 42.6; H, 4.8. **Mp**: 230 °C. ^**1**^ **H NMR** (CDCl_3_, 500.13 MHz): δ (ppm) 0.86 (br s, 3H, CH_3_(dipp)), 1.18 (br s, 3H, CH_3_(dipp)), 1.27–1.32 (br s, 6H, CH_3_(dipp)), 1.45 (s, 15H, CH_3_–Cp*), 2.52 (br s, 1H, CH­(dipp)), 3.11 (br s, 1H, CH_3_O*H* → Zn), 3.45 (s, 3H, C*H*_3_OH → Zn), 3.80 (br s, 1H, CH­(dipp)), 7.00 (br s, 2H, Ar–H), 7.26 (br s, 2H, Ar–H), 7.31 (t, 1H, Ar–H, ^3^ *J*(^1^H, ^1^H) = 7.5 Hz), 7.52 (t, 1H, Ar–H, ^3^ *J*(^1^H, ^1^H) = 8.0 Hz), 7.58 (br s, 1H, Ar–H), 8.46 (s, 1H, CH=N). ^**13**^ **C­{** ^**1**^ **H} NMR** (CDCl_3_, 125.613): δ (ppm) 10.0 (CH_3_–Cp*), 21.9; 24.8; 26.2 (CH_3_(dipp)), 27.9 (CH­(dipp) + CH_3_(dipp)), 51.5 (CH_3_OH → Zn), 90.6 (C-Cp*), 119.6; 124.3; 124.5; 124.8; 129.4 (Ar–CH), 138.9; 142.2; 143.7 (Ar–C), 145.3 (Ar–CH), 151.2; 169.9 (Ar–C), 174.6 (CH=N). **FT-IR** (ATR): ν 1490 (s, ν (C=O); ν 1617 (m, ν C=N) cm^–1^.

### Synthesis of **6**

MeOH solution (2 mL) of ZnCl_2_ (21 mg, 0.16 mmol) was added to the MeOH solution (3 mL) of **3** (100 mg; 0.16 mmol) and **[(η** ^**6**^ *-p-cymene* **)­RuCl­(2-{(2,6-** *i* **Pr**_**2**_ **-C**_**6**_ **H**_**3**_ **)­N**=**CH}-C**_**5**_ **H**_**3**_ **N-6-(O))]** (86 mg, 0.16) at the RT, and the orange mixture was stirred for 16 h. Then, the solvent was concentrated to the half. Orange crystals grown at RT were than decanted, washed with hexane, and dried under reduced pressure. Orange crystalline material was characterized as **6**. **Yield**: 148 mg (71%). Anal. Calcd for **C**_**56**_ **H**_**71**_ **RuIrZnCl**_**4**_ **N**_**4**_ **O**_**2**_ (**M**_**w**_ = **1332.68**) C, 50.5; H, 5.4. Found: C, 50.6; H, 5.4. **Mp**: 284 °C. ^**1**^ **H NMR** (CDCl_3_, 500.13 MHz): δ (ppm) 0.83–0.87 (m, 6H, CH_3_(dipp)), 1.09–1.11 (m, 9H, CH_3_(dipp)), 1.17 (d, 3H, CH_3_(dipp), ^3^ *J*(^1^H, ^1^H) = 6.6 Hz), 1.22–1.26 (m, 6H, CH_3_(dipp)), 1.31 (d, 3H, CH_3_(dipp), ^3^ *J*(^1^H, ^1^H) = 6.5 Hz), 1.38 (d, 3H, CH_3_(dipp), ^3^ *J*(^1^H, ^1^H) = 6.5 Hz), 1.51 (s, 15H, CH_3_–Cp*­{Ir}), 2.14 (s, 3H, CH_3_(cym)­{Ru}), 2.52 (sept, 1H, CH­(dipp)­{Ir}, ^3^ *J*(^1^H, ^1^H) = 6.6 Hz), 2.71 (sept, 1H, CH­(dipp)­{Ru}, ^3^ *J*(^1^H, ^1^H) = 6.6 Hz), 2.88 (sept, 1H, CH­(dipp)­{Ru}, ^3^ *J*(^1^H, ^1^H) = 6.6 Hz), 3.90 (br s, 1H, CH­(dipp)), 3.97 (sept, 1H, CH­(dipp), ^3^ *J*(^1^H, ^1^H) = 6.6 Hz), 4.93 (br s, 1H, Ar­(cym)-H­{Ru}), 5.35 (br s, 1H, Ar­(cym)-H­{Ru}), 6.46 (vbs, 1H, Ar­(cym)-H­{Ru}), 6.90 (br s, 1H, Ar–H), 6.76 (br s, 1H, Ar–H), 7.20–7.30 (m, 5H, Ar–H), 7.33 (t, 1H, Ar–H, ^3^ *J*(^1^H, ^1^H) = 7.7 Hz), 7.39 (br s, 1H, Ar–H), 7.46 (br s, 3H, Ar–H), 7.58 (br s, 1H, Ar–H), 7.81 (s, 1H, CH=N­{Ru}), 8.44 (s, 1H, CH=N­{Ir}). ^**13**^ **C­{** ^**1**^ **H} NMR** (CDCl_3_, 125.613): δ (ppm) 9.9 (CH_3_–Cp*­{Ir}), 19.0 (CH_3_(cym)­{Ru}), 21.5; 21.9; 22.5; 22.9; 23.9; 24.0; 26.0; 26.1; 27.1; 27.2 (CH_3_(*i*Pr)), 27.5; 27.6; 27.7 (CH­(dipp)), 31.5 (CH­(cym)­{Ru}), 81.3; 85.3; 87.4 (Ar­(cym)-C_2,3,5,6_{Ru}), 90.6 (C-Cp*­{Ir}), 100.0; 107.2 (Ar­(cym)-C_1,4_{Ru}),118.1; 122.4; 123.8; 124.0; 124.1; 124.4; 128.5; 138.2 (Ar–CH), 140.2 (Ar–C), 141.7 (Ar–CH), 143.3; 145.2; 148.5; 151.3; 169.5; 171.7 (Ar–C), 171.7 (CH=N­{Ru}) 174.2 (CH=N­{Ir}). **FT-IR** (ATR): ν 1488 (s, ν (C = O); ν 1616 (m, ν C=N) cm^–1^.

### General Procedure for the O-Functionalization

Complex **3** (cca. 150 mg) was dissolved in 1 mL of MeI (**2**), I­(CH_2_)_6_I (**7**) PhCH_2_Br (**8**), *t*BuCOCH_2_Br (**9**), NCCH_2_Br (**10**), or MeCO_2_CH_2_Br (**11**). The solution or suspension was stirred for 16 h at RT. Then, ionic complexes were precipitated with hexane (**2**, **7**, **8**, **9**) or with Et_2_O (**10**, **11**) and washed 3 times with that solvent and dried under the reduced pressure to give brown-red (**2**, **7**) or orange (**8–11**) powdery materials in yields 80–95%. The hexane/Et_2_O solution of RX (except of MeI due to low boiling point) can be evaporated, and RX can be reused for the next reaction. Complex **14** (cca. 150 mg) was dissolved in 1 mL of MeI, and the solution was stirred for 16 h at RT and hexane was added then to precipitate the Ir-complexes. The orange residue was dried and characterized by ^1^H and ^13^C­{^1^H} NMR spectrum (see Figures S71 and S72). *N*-Methyl-2-pyridone (cca. 100 mg) was dissolved in 1 mL of MeI, and the solution was stirred for 16 h at RT. The solution was vacuum-dried to evaporated excess of MeI and the residue characterized by ^1^H NMR spectrum (Figure S73).

#### 7: Yield

210 mg (83% from 152 mg of **3**). Anal. Calcd for **C**_**34**_ **H**_**48**_ **IrI**_**3**_ **N**_**2**_ **O** (**M**_**w**_ = **1073.70**) C, 38.0; H, 4.5. Found: C, 38.2; H, 4.6. **Mp**: thermally unstable. ^**1**^ **H NMR** (CDCl_3_, 500.13 MHz): δ (ppm) 0.82 (d, 3H, CH_3_(dipp), ^3^ *J*(^1^H, ^1^H) = 6.5 Hz), 1.19 (d, 3H, CH_3_(dipp), ^3^ *J*(^1^H, ^1^H) = 6.5 Hz), 1.23 (d, 3H, CH_3_(dipp), ^3^ *J*(^1^H, ^1^H) = 6.7 Hz), 1.32 (d, 3H, CH_3_(dipp), ^3^ *J*(^1^H, ^1^H) = 6.5 Hz), 1.40–1.48 (m, 4H, CH_2_), 1.53 (s, 15H, CH_3_–Cp*), 1.77–1.97 (m, 4H, CH_2_), 2.42 (sept, 1H, CH­(dipp), ^3^ *J*(^1^H, ^1^H) = 6.5 Hz), 3.11–3.16 (m, 2H, CH_2_), 3.96 (sept, 1H, CH­(dipp), ^3^ *J*(^1^H, ^1^H) = 6.7 Hz), 4.46–4.51 (m, 1H, CH_2_), 4.87–4.92 (m, 1H, CH_2_), 7.23–7.35 (m, 3H, Ar–H), 7.68 (d, 1H, Ar–H, ^3^ *J*(^1^H, ^1^H) = 8.6 Hz), 7.97 (d, 1H, Ar–H, ^3^ *J*(^1^H, ^1^H) = 7.3 Hz), 8.18 (t, 1H, Ar–H, ^3^ *J*(^1^H, ^1^H) = 8.0 Hz), 8.83 (s, 1H, CH = N). ^**13**^ **C­{** ^**1**^ **H} NMR** (CDCl_3_, 125.613): δ (ppm) 7.6 (CH_2_), 11.1 (CH_3_–Cp*), 21.5; 24.7 (CH_3_(dipp)); 25.1 (CH_2_), 26.3 (CH_3_(dipp)), 28.2 (CH­(dipp)), 28.3 (CH_2_), 28.7 (CH_3_(dipp)), 29.8 (CH­(dipp)), 30.4; 33.5 (CH_2_), 72.1 (OCH_2_), 91.9 (C-Cp*), 114.2; 124.5; 124.7; 125.1; 130.0 (Ar–CH), 142.0; 143.2 (Ar–C), 143.4 (Ar–CH), 145.2; 153.0; 165.3 (Ar–C), 172.7 (CH=N). **FT-IR** (ATR): ν 1614 (m, ν C=N) cm^–1^. **HRMS** (MALDI): *m*/*z* calcd for C_34_H_48_^193^IrI_2_N_2_O: 947.14798 [M]^+^; found, 947.14637; calcd for C_34_H_48_^193^IrIN_2_O, 820.24351­[M – I]^+^; found, 820.24272.

#### 8: Yield

167 mg (87% from 144 mg of **3**). Anal. Calcd for **C**_**35**_ **H**_**43**_ **IrBr**_**2**_ **N**_**2**_ **O** (**M**_**w**_ = **859.77**) C, 48.9; H, 5.0. Found: C, 49.1; H, 5.1. **Mp**: thermally unstable. ^**1**^ **H NMR** (CDCl_3_, 400.13 MHz): δ (ppm) 0.81 (d, 3H, CH_3_(dipp), ^3^ *J*(^1^H, ^1^H) = 6.5 Hz), 1.17 (d, 3H, CH_3_(dipp), ^3^ *J*(^1^H, ^1^H) = 6.5 Hz), 1.21 (d, 3H, CH_3_(dipp), ^3^ *J*(^1^H, ^1^H) = 6.5 Hz), 1.29 (d, 3H, CH_3_(dipp), ^3^ *J*(^1^H, ^1^H) = 6.5 Hz), 1.38 (s, 15H, CH_3_–Cp*), 2.40 (sept, 1H, CH­(dipp), ^3^ *J*(^1^H, ^1^H) = 6.5 Hz), 3.83 (sept, 1H, CH­(dipp), ^3^ *J*(^1^H, ^1^H) = 6.5 Hz), 5.69 (AM system, 1H, CH_2_), 6.00 (AM system, 1H, CH_2_), 7.21–7.33 (m, 6H, Ar–H), 7.49 (d, 1H, Ar–H, ^3^ *J*(^1^H, ^1^H) = 8.2 Hz), 7.55 (d, 2H, Ar–H, ^3^ *J*(^1^H, ^1^H) = 7.2 Hz), 7.95 (t, 1H, Ar–H, ^3^ *J*(^1^H, ^1^H) = 8.2 Hz), 8.04 (d, 1H, Ar–H, ^3^ *J*(^1^H, ^1^H) = 7.0 Hz), 8.95 (s, 1H, CH=N). ^**13**^ **C­{** ^**1**^ **H} NMR** (CDCl_3_, 100.613): δ (ppm) 10.1 (CH_3_–Cp*), 21.6; 24.5; 26.2 (CH_3_(dipp)), 28.1 (CH­(dipp)), 28.3 (CH_3_(dipp)), 28.4 (CH­(dipp)), 73.0 (OCH_2_), 91.6 (C-Cp*), 115.5; 124.5; 124.7; 124.9; 127.6; 129.0; 129.2; 129.8; 133.7 (Ar–CH), 141.8; 143.1 (Ar–CH), 143.2 (Ar–CH), 145.0; 152.6; 164.4 (Ar–C), 173.4 (CH=N). **FT-IR** (ATR): ν 1616 (w, ν C=N) cm^–1^. **HRMS** (MALDI): *m*/*z* calcd for C_35_H_43_^193^Ir^79^BrN_2_O, 779.21825 [M]^+^; found, 779.21570; calcd for C_35_H_42_^193^IrN_2_O, 699.29209 [M-HBr]^+^; found, 699.29225.

#### **9**: Yield

194 mg (94% from 153 mg of **3**). Anal. Calcd for **C**_**34**_ **H**_**47**_ **IrBr**_**2**_ **N**_**2**_ **O**_**2**_ (**M**_**w**_ = **867.79**) C, 47.1; H, 5.5. Found: C, 47.3; H, 5.6. **Mp**: thermally unstable. ^**1**^ **H NMR** (CDCl_3_, 500.13 MHz): δ (ppm) 0.88 (d, 3H, CH_3_(dipp), ^3^ *J*(^1^H, ^1^H) = 6.5 Hz), 1.21 (d, 3H, CH_3_(dipp), ^3^ *J*(^1^H, ^1^H) = 6.5 Hz), 1.26 (d, 3H, CH_3_(dipp), ^3^ *J*(^1^H, ^1^H) = 7.0 Hz), 1.32 (s, 9H, *t*Bu), 1.34 (d, 3H, CH_3_(dipp), ^3^ *J*(^1^H, ^1^H) = 7.0 Hz), 1.43 (s, 15H, CH_3_–Cp*), 2.41 (sept, 1H, CH­(dipp), ^3^ *J*(^1^H, ^1^H) = 6.5 Hz), 3.83 (sept, 1H, CH­(dipp), ^3^ *J*(^1^H, ^1^H) = 7.0 Hz), 5.77 (AB system, 2H, CH_2_), 7.26–7.38 (m, 3H, Ar–H), 7.42 (d, 1H, Ar–H, ^3^ *J*(^1^H, ^1^H) = 8.5 Hz), 8.07 (t, 1H, Ar–H, ^3^ *J*(^1^H, ^1^H) = 8.0 Hz), 8.27 (d, 1H, Ar–H, ^3^ *J*(^1^H, ^1^H) = 7.2 Hz), 9.16 (s, 1H, CH = N). ^**13**^ **C­{** ^**1**^ **H} NMR** (CDCl_3_, 125.613): δ (ppm) 9.9 (CH_3_–Cp*), 21.4; 24.1; 26.1 (CH_3_(dipp)), 26.3 (C­(*C*H_3_)_3_), 27.9 (CH­(dipp)), 28.1 (CH_3_(dipp)), 28.2 (CH­(dipp)), 43.3 (*C*(CH_3_)_3_), 71.6 (OCH_2_), 91.4 (C-Cp*), 114.5; 124.3; 124.6; 125.0; 129.6; (Ar–CH), 141.8; 142.8 (Ar–C), 143.1 (Ar–CH), 144.7; 152.5; 164.3 (Ar–C), 173.5 (CH=N), 208.6 (C=O). **FT-IR** (ATR): ν 1616 (m, ν C=N), ν 1720 (m, ν (C=O) cm^–1^. **HRMS** (MALDI): *m*/*z* calcd for C_34_H_47_^193^Ir^79^BrN_2_O_2_, 787.24447 [M]^+^; found, 787.24245; calcd for C_34_H_47_^193^IrN_2_O_2_, 708.32613 [M – Br]^+^; found, 708.32631.

#### **10**: Yield

176 mg (93% from 150 mg of **3**). Anal. Calcd for **C**_**30**_ **H**_**38**_ **IrBr**_**2**_ **N**_**3**_ **O** (**M**_**w**_ = **808.68**) C, 44.6; H, 4.7. Found: C, 44.4; H, 4.7. **Mp**: thermally unstable. ^**1**^ **H NMR** (CDCl_3_, 500.13 MHz): δ (ppm) 0.84 (d, 3H, CH_3_(dipp), ^3^ *J*(^1^H, ^1^H) = 6.5 Hz), 1.19 (d, 3H, CH_3_(dipp), ^3^ *J*(^1^H, ^1^H) = 7.0 Hz), 1.22 (d, 3H, CH_3_(dipp), ^3^ *J*(^1^H, ^1^H) = 7.0 Hz), 1.31 (d, 3H, CH_3_(dipp), ^3^ *J*(^1^H, ^1^H) = 6.5 Hz), 1.46 (s, 15H, CH_3_–Cp*), 2.38 (sept, 1H, CH­(dipp), ^3^ *J*(^1^H, ^1^H) = 6.5 Hz), 3.78 (sept, 1H, CH­(dipp), ^3^ *J*(^1^H, ^1^H) = 7.0 Hz), 5.95 (s, 2H, CH_2_), 7.23–7.36 (m, 3H, Ar–H), 8.11 (d, 1H, Ar–H, ^3^ *J*(^1^H, ^1^H) = 8.3 Hz), 8.16 (t, 1H, Ar–H, ^3^ *J*(^1^H, ^1^H) = 8.0 Hz), 8.22 (d, 1H, Ar–H, ^3^ *J*(^1^H, ^1^H) = 7.2 Hz), 9.09 (s, 1H, CH = N). ^**13**^ **C­{** ^**1**^ **H} NMR** (CDCl_3_, 125.613): δ (ppm) 10.1 (CH_3_–Cp*), 22.7; 24.3; 26.1 (CH_3_(dipp)), 28.0 (CH­(dipp)), 28.1 (CH_3_(dipp)), 28.3 (CH­(dipp)), 57.3 (OCH_2_), 91.8 (C-Cp*), 114.0 (CN), 114.9; 124.4; 124.5; 124.8; 125.5; 129.9 (Ar–CH), 141.6; 143.0 (Ar–C), 143.9 (Ar–CH), 144.8; 152.9; 162.8 (Ar–C), 173.0 (CH = N). **FT-IR** (ATR): ν 1616 (w, ν C = N). **HRMS** (MALDI): *m*/*z* calcd for C_30_H_38_^193^Ir^79^BrN_3_O: 728.18220 [M]^+^; found, 728.18006; calcd for C_30_H_38_^193^IrN_3_O: 649.26386 [M – Br]^+^; found, 649.26442.

#### **11**: Yield

194 mg (95% from 155 mg of **3**). Anal. Calcd for **C**_**31**_ **H**_**41**_ **IrBr**_**2**_ **N**_**2**_ **O**_**3**_ (**M**_**w**_ = **841.71**) C, 44.2; H, 4.9. Found: C, 44.3; H, 4.9. **Mp**: thermally unstable. ^**1**^ **H NMR** (CDCl_3_, 500.13 MHz): δ (ppm) 0.82 (d, 3H, CH_3_(dipp), ^3^ *J*(^1^H, ^1^H) = 6.5 Hz), 1.18–1.21 (m, 6H, CH_3_(dipp)), 1.29 (d, 3H, CH_3_(dipp), ^3^ *J*(^1^H, ^1^H) = 6.5 Hz), 1.40 (s, 15H, CH_3_–Cp*), 2.38 (sept, 1H, CH­(dipp), ^3^ *J*(^1^H, ^1^H) = 6.5 Hz), 3.80 (br s, 4H, OCH_3_ + CH­(dipp)), 5.30 (AB system, 2H, CH_2_), 7.21–7.31 (m, 3H, Ar–H), 7.59 (d, 1H, Ar–H, ^3^ *J*(^1^H, ^1^H) = 8.4 Hz), 8.16 (t, 1H, Ar–H, ^3^ *J*(^1^H, ^1^H) = 8.4 Hz), 8.30 (d, 1H, Ar–H, ^3^ *J*(^1^H, ^1^H) = 7.0 Hz), 9.13 (s, 1H, CH = N). ^**13**^ **C­{** ^**1**^ **H} NMR** (CDCl_3_, 125.613): δ (ppm) 10.0 (CH_3_–Cp*), 21.5; 24.2; 26.1 (CH_3_(dipp)), 28.0 (CH­(dipp)), 28.2 (CH_3_(dipp)), 28.3 (CH­(dipp)), 53.2 (CO_2_ *C*H_3_), 67.2 (OCH_2_), 91.5 (C-Cp*), 114.2; 124.4; 124.7; 125.4; 129.7 (Ar–CH), 141.5; 143.0 (Ar–C), 143.7 (Ar–CH), 144.8; 152.8; 163.9 (Ar–C), 167.5 (*C*O_2_Me), 173.4 (CH = N). **FT-IR** (ATR): ν 1615 (m, ν C=N), ν 1750 (m, ν (C=O) cm^–1^. **HRMS** (MALDI): *m*/*z* calcd for C_31_H_41_^193^Ir^79^BrN_2_O_3_: 761.19243 [M]^+^; found, 761.18978; calcd for C_31_H_41_^193^IrN_2_O_3_: 682.27409 [M-Br]^+^; found, 682.27397.

### Synthesis of **12**

Compound **8** (150 mg; 0.17 mmol) was dissolved in 3 mL of MeOH. AgOTf (45 mg, 0.17 mmol) in 2 mL of MeOH was dissolved in 2 mL of MeOH. Both solutions were mixed, and the precipitate of AgCl appeared immediately. The mixture was stirred in the dark for 16 h. Then, the orange solution was filtered, solvent was evaporated, and the orange solid was dissolved in 1 mL of hot THF. Red crystals grown up at 5 °C were decanted, dissolved in CH_2_Cl_2_, and precipitated with hexane. Red solid material was washed with hexane 3 times, dried under reduced pressure, and characterized as **12**. **Yield**: 138 mg (85%). Anal. Calcd for **C**_**36**_ **H**_**43**_ **IrF**_**3**_ **BrN**_**2**_ **O**_**4**_ **S** (**M**_**w**_ = **928.93**) C, 46.6; H, 4.7. Found: C, 46.5; H, 4.7. **Mp**: 148 °C. ^**1**^ **H NMR** (CDCl_3_, 500.13 MHz): δ (ppm) 0.84 (d, 3H, CH_3_(dipp), ^3^ *J*(^1^H, ^1^H) = 6.5 Hz), 1.17 (d, 3H, CH_3_(dipp), ^3^ *J*(^1^H, ^1^H) = 6.5 Hz), 1.26 (d, 3H, CH_3_(dipp), ^3^ *J*(^1^H, ^1^H) = 6.5 Hz), 1.33 (d, 3H, CH_3_(dipp), ^3^ *J*(^1^H, ^1^H) = 6.5 Hz), 1.42 (s, 15H, CH_3_–Cp*), 2.53 (sept, 1H, CH­(dipp), ^3^ *J*(^1^H, ^1^H) = 6.5 Hz), 3.89 (sept, 1H, CH­(dipp), ^3^ *J*(^1^H, ^1^H) = 6.5 Hz), 5.70 (AB system, 2H, CH_2_), 7.24–7.34 (m, 7H, Ar–H), 7.52 (d, 2H, Ar–H, ^3^ *J*(^1^H, ^1^H) = 7.0 Hz), 7.70 (d, 1H, Ar–H, ^3^ *J*(^1^H, ^1^H) = 7.0 Hz), 7.88 (t, 1H, Ar–H, ^3^ *J*(^1^H, ^1^H) = 7.5 Hz), 8.76 (s, 1H, CH = N). ^**13**^ **C­{** ^**1**^ **H} NMR** (CDCl_3_, 125.613): δ (ppm) 9.6 (CH_3_–Cp*), 21.3; 24.2; 25.6 (CH_3_(dipp)), 27.7 (CH­(dipp)), 27.9 (CH_3_(dipp)), 28.2 (CH­(dipp)), 72.4 (OCH_2_), 91.5 (C-Cp*), 114.8; 123.6; 124.2; 124.8; 128.8; 129.1; 129.6; 133.4 (Ar–CH), 141.9; (Ar–C), 142.5 (Ar–CH), 142.9; 144.8; 152.3; 164.3 (Ar–C), 172.8 (CH=N). **FT-IR** (ATR): ν 1617 (m, ν C=N) cm^–1^.

### Synthesis of **13**

Compound **13** was prepared analogously to **12** with the use of KPF_6_. **Yield**: 141 mg (88% from 150 mg of **9**). Anal. Calcd for **C**_**34**_ **H**_**47**_ **IrF**_**6**_ **BrN**_**2**_ **O**_**2**_ **P** (**M**_**w**_ = **932.85**) C, 43.8; H, 5.1. Found: C, 43.6; H, 5.0. **Mp**: 178 °C. ^**1**^ **H NMR** (CDCl_3_, 500.13 MHz): δ (ppm) 0.81–0.85 (m, 6H, CH_3_(dipp)), 1.15 (d, 3H, CH_3_(dipp), ^3^ *J*(^1^H, ^1^H) = 6.7 Hz), 1.25 (s, 9H, *t*Bu), 1.33 (d, 3H, CH_3_(dipp), ^3^ *J*(^1^H, ^1^H) = 6.7 Hz), 1.39 (s, 15H, CH_3_–Cp*), 2.42 (sept, 1H, CH­(dipp), ^3^ *J*(^1^H, ^1^H) = 6.7 Hz), 3.81 (sept, 1H, CH­(dipp), ^3^ *J*(^1^H, ^1^H) = 6.7 Hz), 5.31 (AM system, 1H, CH_2_), 5.48 (AM system, 1H, CH_2_), 6.92 (d, 1H, Ar–H, ^3^ *J*(^1^H, ^1^H) = 8.9 Hz), 7.25–7.37 (m, 3H, Ar–H),, 7.78 (d, 1H, Ar–H, ^3^ *J*(^1^H, ^1^H) = 7.0 Hz), 7.97 (t, 1H, Ar–H, ^3^ *J*(^1^H, ^1^H) = 8.0 Hz), 8.79 (s, 1H, CH=N). ^**13**^ **C­{** ^**1**^ **H} NMR** (CDCl_3_, 125.613): δ (ppm) 9.8 (CH_3_–Cp*), 21.5; 24.1; 26.0 (CH_3_(dipp)), 26.1 (C­(*C*H_3_)_3_), 27.9 (CH­(dipp)), 28.0 (CH_3_(dipp)), 28.3 (CH­(dipp)), 43.4 (*C*(CH_3_)_3_), 70.4 (OCH_2_), 91.7 (C-Cp*), 113.5; 123.9; 124.4; 124.7; 129.8; (Ar–CH), 141.6 (Ar–C), 143.0 (Ar–CH), 144.8; 152.5; 164.4 (Ar–C), 172.9 (CH=N), 208.1 (C=O). ^**31**^ **P­{** ^**1**^ **H} NMR** (CDCl_3_, 202.457): δ (ppm) −145.3 (sept). **FT-IR** (ATR): ν 834 (ν_asym_ P–F); ν 1619 (w, ν C=N), ν 1717 (w, ν (C=O) cm^–1^.

### Synthesis of **14**

A solution of K­[Ag­(CN)_2_] (85 mg, 0.23 mmol) in MeOH (2 mL) was added to a solution of **3** (150 mg, 0.23 mmol). The color of the solution changed from dark orange to yellow, accompanied by the formation of a white precipitate. The mixture was stirred at RT for 16 h in the absence of light. The solvent was then evaporated, and the yellow residue was extracted with DCM to remove AgCl and KCN. The solvent was evaporated again, and the resulting solid was washed and reprecipitated with hexane, then dried under reduced pressure. The bright yellow powder was characterized as **14**. **Yield**: 142 mg (96%). Anal. Calcd for **C**_**29**_ **H**_**36**_ **IrN**_**3**_ **O** (**M**_**w**_ = **634.84**) C, 54.9; H, 5.7. Found: C, 55.0; H, 5.7. **Mp**: 236 °C. ^**1**^ **H NMR** (CDCl_3_, 500.13 MHz): δ (ppm) 1.04 (d, 3H, CH_3_(dipp), ^3^ *J*(^1^H, ^1^H) = 6.9 Hz), 1.17 (d, 3H, CH_3_(dipp), ^3^ *J*(^1^H, ^1^H) = 7.0 Hz), 1.36 (d, 6H, CH_3_(dipp), ^3^ *J*(^1^H, ^1^H) = 7.0 Hz), 1.68 (s, 15H, CH_3_–Cp*), 2.56 (sept, 1H, CH­(dipp), ^3^ *J*(^1^H, ^1^H) = 7.0 Hz), 3.98 (sept, 1H, CH­(dipp), ^3^ *J*(^1^H, ^1^H) = 7.0 Hz), 6.50 (d, 1H, Ar–H, ^3^ *J*(^1^H, ^1^H) = 8.8 Hz), 6.67 (d, 1H, Ar–H, ^3^ *J*(^1^H, ^1^H) = 6.5 Hz), 7.24–7.33 (m, 4H, Ar–H), 8.28 (s, 1H, CH=N). ^**13**^ **C­{** ^**1**^ **H} NMR** (CDCl_3_, 125.613): δ (ppm) 10.2 (CH_3_–Cp*), 22.2; 24.5; 26.0 (CH_3_(dipp)), 27.8; 28.1 (CH­(dipp)), 28.2 (CH_3_(dipp)), 93.3 (C-Cp*), 116.0 (Ir–CN); 116.6; 124.0; 124.4; 124.8; 128.9; 135.7 (Ar–CH), 141.5; 143.5; 145.2; 152.4 (Ar–C), 168.7 (Ar–C = O), 173.9 (CH=N). **FT-IR** (ATR): ν 1554 (s, ν C=O), 1618 (w, ν C=N), 2121 (m, ν C≡N) cm^–1^.

### Reverse Elimination of the Alkyl Halides

Complexes **10** and **13** (250 mg) were heated in a solid state in a flask open to air for 2 h at 120 °C to give a powdery material. The ^1^H NMR spectra suggested elimination of CNCH_2_Br and formation of a new product [(η^5^ -Cp*)­IrBr­(2-{(2,6-*i*Pr_2_–C_6_H_3_)N = CH}–C_5_H_3_N–6-(O))] in the case of **10**, while complex **13** is thermally stable (Figures S74 and S75).

### Crystallography

Single crystals of **4** and **6** were grown from CH_2_Cl_2_/hexane (2:1; **4**), MeOH (**6**) by slow evaporation at room temperature and characterized as **4** and **11**. Single crystals of **3**, **5**, **12**, and **13** suitable for X-ray diffraction analysis were grown in toluene (**3**), MeOH (**5**), CH_2_Cl_2_ (**7**), and THF (**12**, **13**), and at 5 °C and characterized as **3•**C_7_H_8_, **5**·MeOH, **12**·2THF and **13**·THF (Figures S60–S65). Diffraction data for **3**·C7H8, **4**, **5**·MeOH, **6**, **12**·2THF, and **13**·THF were collected using a Bruker Venture D8 diffractometer at 150 K with graphite-monochromated Mo–Kα (0.7107 Å) radiation. The frames were integrated with the Bruker SAINT software package using a narrow frame algorithm. Data were corrected for absorption effects using the Multi-Scan method (SADABS). Obtained data were treated by XT-version 2014/5 and SHELXL-2019/1 software implemented in APEX4 v2021.10–0 (Bruker AXS) system.^34^ All non-hydrogen atoms were refined using anisotropic displacement parameters. There are residual electron maxima within the unit cell originated from the disordered solvent in the structure of **6** and **13**, PLATON/SQUEEZE was used to correct the data for the presence of disordered solvent.^35^ Crystallographic data for the structural analyses have been deposited with the Cambridge Crystallographic Data Centre, CCDC nos. 2519843–2519848.

Copies of this information may be obtained free of charge from The Director, CCDC, 12 Union Road, Cambridge CB2 1EY, UK (fax: +44–1223–336033; e-mail: deposit@ccdc.cam.ac.uk; or Web site: http://www.ccdc.cam.ac.uk).

## Supplementary Material



## Notes

NMR and IR spectra for prepared compounds; crystallographic parameters; photophysical properties of **1**–**11** and **14**; photoluminescence of **1**, **3**, and **5**; and discussion of DFT calculation (PDF)
