# Supplementary material for: Iminopyridonato Iridium Complexes: O‑Functionalization via C‑X Bond Cleavage
Source: Inorg Chem. 2026 May 17;65(21):11653–64. doi: 10.1021/acs.inorgchem.6c00285 (PMC13231413; doi:10.1021/acs.inorgchem.6c00285)
Supplement: Supplementary file 1 [file ic6c00285_si_001.pdf]

SI for: Iminopyridonato Iridium Complexes: *O*-Functionalization *via* C-X Bond Cleavage

Ondřej Moždiak,[a] Jiří Tydlitát,[b] Zdeňka Růžičková,[a] Andreas Steffen[c] and Roman Jambor*[a]*

[a] Ondřej Moždiak, Dr. Zdeňka Růžičková, Prof. Dr. Roman Jambor

Department of General and Inorganic Chemistry, University of Pardubice

532 10 Pardubice, Czech Republic

E-mail: roman.jambor@upce.cz

[b] Dr. Jiří Tydlitát

Institute of Organic Chemistry and Technology, University of Pardubice

532 10 Pardubice, Czech Republic

E-mail: andreas.steffen@tu-dortmund.de

[c] Prof. Dr. Andreas Steffen

Department of Chemistry and Chemical Biology, TU Dortmund University

442 27 Dortmund, Germany

E-mail: andreas.steffen@tu-dortmund.de

Characterization of the Complexes: Spectroscopic data (NMR, MS, FT-IR, UV/VIS) S6
Characterization of the Complexes: Crystallographic data S51
TD-DFT study of 3 .... S69
Photophysical Properties of 1, 3 and 5 in the DCM solution S74
Photophysical Properties of 1, 3 and 5 in the solid state S86
References S98

Figure S1. ^{1}H NMR ($CDCl_3$, 500.13 MHz, 300 K) of **1**.......... S7
Figure S2. $^{13}C\{^{1}H\}$ NMR ($CDCl_3$, 125.76 MHz, 300 K) of **1**.......... S8
Figure S3. ^{1}H NMR ($CDCl_3$, 500.13 MHz, 300 K) of **2**.......... S9
Figure S4. $^{13}C\{^{1}H\}$ NMR ($CDCl_3$ 125.78 MHz, 300 K) of **2**.......... S10
Figure S5. ^{1}H NMR ($CDCl_3$, 500.13 MHz, 300 K) of **3**.......... S11
Figure S6. $^{13}C\{^{1}H\}$ NMR ($CDCl_3$, 125.76 MHz, 300 K) of **3**.......... S12
Figure S7. ^{1}H NMR ($CDCl_3$, 500.13 MHz, 300 K) of **4**.......... S13
Figure S8. $^{13}C\{^{1}H\}$ NMR ($CDCl_3$, 125.78 MHz, 300 K) of **4**.......... S14
Figure S9. ^{1}H NMR ($CDCl_3$, 500.13 MHz, 300 K) of **5**.......... S15
Figure S10. $^{13}C\{^{1}H\}$ NMR ($CDCl_3$, 125.78 MHz, 300 K) of **5**.......... S16
Figure S11. ^{1}H NMR ($CDCl_3$, 500.13 MHz, 300 K) of **6**.......... S17
Figure S12. $^{13}C\{^{1}H\}$ NMR ($CDCl_3$, 125.78 MHz, 300 K) of **6**.......... S18
Figure S13. ^{1}H NMR ($CDCl_3$, 500.13 MHz, 300 K) of **7**.......... S19
Figure S14. ^{1}H-^{1}H COSY NMR ($CDCl_3$, 500.13 MHz, 300 K) of **7**.......... S20
Figure S15. $^{13}C\{^{1}H\}$ NMR ($CDCl_3$, 100.61 MHz, 300 K) of **7**.......... S21
Figure S16. ^{1}H NMR ($CDCl_3$, 400.13 MHz, 300 K) of **8**.......... S22
Figure S17. $^{13}C\{^{1}H\}$ NMR ($CDCl_3$, 100.61 MHz, 300 K) of **8**.......... S23
Figure S18. ^{1}H NMR ($CDCl_3$, 500.13 MHz, 300 K) of **9**.......... S24

Figure S19. $^{13}C\{^{1}H\}$ NMR ($CDCl_3$, 125.78 MHz, 300 K) of **9**. S25
Figure S20. ^{1}H NMR ($CDCl_3$, 500.13 MHz, 300 K) of **10**. S26
Figure S21. $^{13}C\{^{1}H\}$ NMR ($CDCl_3$, 125.76 MHz, 300 K) of **10**. S27
Figure S22. ^{1}H NMR ($CDCl_3$, 500.13 MHz, 300 K) of **11**. S28
Figure S23. $^{13}C\{^{1}H\}$ NMR ($CDCl_3$, 125.76 MHz, 300 K) of **11**. S29
Figure S24. ^{1}H NMR ($CDCl_3$, 500.13 MHz, 300 K) of **12**. S30
Figure S25. $^{13}C\{^{1}H\}$ NMR ($CDCl_3$, 125.78 MHz, 300 K) of **12**. S31
Figure S26. $^{19}F\{^{1}H\}$ NMR ($CDCl_3$, 376.50 MHz, 300 K) of **12**. S32
Figure S27. ^{1}H NMR ($CDCl_3$, 500.13 MHz, 300 K) of **13**. S33
Figure S28. $^{13}C\{^{1}H\}$ NMR ($CDCl_3$, 125.78 MHz, 300 K) of **13**. S34
Figure S29. $^{31}P\{^{1}H\}$ NMR ($CDCl_3$, 202.46 MHz, 300 K) of **13**. S35
Figure S30. ^{1}H NMR ($CDCl_3$, 400.13 MHz, 300 K) of **14**. S36
Figure S31. $^{13}C\{^{1}H\}$ NMR ($CDCl_3$, 100.61 MHz, 300 K) of **14**. S37
Figure S32. MS spectrum of **7**, $[M]^+$, top: experimental, bottom: simulated. S38
Figure S33. MS spectrum of **7**, $[M-I]^+$, top: experimental, bottom: simulated. S38
Figure S34. MS spectrum of **8**, $[M]^+$, top: experimental, bottom: simulated. S39
Figure S35. MS spectrum of **8**, $[M-HBr]^+$, top: experimental, bottom: simulated. S39
Figure S36. MS spectrum of **9**, $[M]^+$, top: experimental, bottom: simulated. S40
Figure S37. MS spectrum of **9,** $[M-Br]^+$, top: experimental, bottom: simulated. S40
Figure S38. MS spectrum of **10**, $[M]^+$, top: experimental, bottom: simulated. S41
Figure S39. MS spectrum of **10**, $[M-Br]^+$, top: experimental, bottom: simulated. S41
Figure S40. MS spectrum of **11**, $[M]^+$, top: experimental, bottom: simulated. S42
Figure S41. MS spectrum of **11**, $[M-Br]^+$, top: experimental, bottom: simulated. S42
Figure S42. FT-IR (ATR) of **1**. Powder. S43
Figure S43. FT-IR (ATR) of **2**. Powder. S43
Figure S44. FT-IR (ATR) of **3**. Powder. S43
Figure S45. FT-IR (ATR) of **4**. Powder. S44
Figure S46. FT-IR (ATR) of **5**. Powder. S44
Figure S47. FT-IR (ATR) of **6**. Powder. S44
Figure S48. FT-IR (ATR) of **7**. Powder. S45
Figure S49. FT-IR (ATR) of **8**. Powder. S45

Figure S50. FT-IR (ATR) of **12**. Powder. S45
Figure S51. FT-IR (ATR) of **9**. Powder. S46
Figure S52. FT-IR (ATR) of **13**. Powder. S46
Figure S53. FT-IR (ATR) of **10**. Powder. S47
Figure S54. FT-IR (ATR) of **11**. Powder. S47
Figure S55. FT-IR (ATR) of **14**. Powder. S47
Figure S56. Absorption spectra of ionic **1—2** and neutral **3—4** Ir(III) complexes. S48
Figure S57. Absorption spectra of bimetallic complexes **5** and **6** compared with the starting Ir(III) complex **3**....... S49
Figure S58. Absorption spectra of ionic complexes **2**, **7—11**. S49
Figure S59. Absorption spectra of neutral complex **14** compared with analogues **3** and **4**....... S50
Figure S60. ORTEP of **3·C_7H_8**....... S51
Figure S61. ORTEP of **4**. S52
Figure S62. ORTEP of **5·MeOH**....... S53
Figure S63. ORTEP of **6**. S54
Figure S64. ORTEP of **12·2THF** S55
Figure S65. ORTEP of **13·THF** S56
Table S1. Crystallographic data of 3·C7H8, 4, 5·MeOH, 6, 12· 2THF, 13·1.5THF....... S57

Figure S66. ^{1}H NMR ($CDCl_3$, 500.13 MHz, 300 K) of the reaction mixture of **3** with a minimal amount of $BrCH_2COtBu$ under neat conditions after 16 h. . S59
Figure S67. ^{1}H NMR ($CDCl_3$, 500.13 MHz, 300 K) of **3** + MeI kinetic study. S60
Figure S68. ^{1}H NMR ($CDCl_3$, 500.13 MHz, 300 K) of **4** + MeI kinetic study. S62
Figure S69. ^{1}H NMR ($CDCl_3$, 400.13 MHz, 300 K) of the reaction mixture of **4** and $BrCH_2COtBu$ under similar conditions as for **3** and $BrCH_2COtBu$. Neat, 16 h, RT....... S63
Figure S70. Left: Absorption spectra of the reaction mixture of **3** with MeI (black solid) recorded after 1 h.. .... S63
Figure S71. ^{1}H NMR ($CDCl_3$, 500.13 MHz, 300 K) of the reaction mixture of **14** with MeI and **15**....... S64
Figure S72. $^{13}C\{^1H\}$ NMR ($CDCl_3$, 125.78 MHz, 300 K) of the reaction mixture of **14** with MeI and **15**....... S65
Figure S73. ^{1}H NMR ($CDCl_3$, 500.13 MHz, 300 K) of N-methylpyridone and its reaction with MeI under neat conditions, 16 h, RT....... S66
Figure S74. ^{1}H NMR ($CDCl_3$, 400.13 MHz, 300 K) of **10** before heating (red spectrum) and after heating in the solid phase (blue spectrum) with supposed major product. S67
Figure S75. ^{1}H NMR ($CDCl_3$, 500.13 MHz, 300 K) of **13·THF** before heating (red spectrum) and after heating (blue spectrum) in the solid phase. S68
Table S2. TD-DFT calculated vertical electronic transitions from the DFT optimized ground state S0 of 3....... S69

Table S3. Photophysical data of 1, 3 and 5 in DCM solution and in the solid state at 297 K....S73

Figure S76. Excitation (yellow) and emission (blue) spectra of **1**, DCM solution, 293 K, λ_{ex} = 490 nm. S74
Figure S77. Emission spectrum of **1**, DCM solution, 293 K, λ_{ex} = 490 nm, NIR detector....S75
Figure S78. Experimental time-resolved luminescence decay (yellow) with IRF (blue) of **1**, DCM solution, 293 K. Numerical biexponential reconvulation fit (purple). S76
Figure S79. Excitation (blue) and emission (yellow) spectra of **3**, DCM solution, 293 K, λ_{ex} = 480 nm. S77
Figure S80. Emission spectra of **3**, DCM solution, 293 K, λ_{ex} = 480 nm (blue), λ_{ex} = 420 nm (purple) and λ_{ex} = 400 nm (yellow). S78
Figure S81. Emission spectrum of **3**, DCM solution, 293 K, λ_{ex} = 480 nm, NIR detector....S79
Figure S82. Experimental time-resolved luminescence decay (yellow) with IRF (blue) of **3**, DCM solution, 293 K. Numerical biexponential reconvulation fit (purple). S80
Figure S83. Excitation (blue) and emission (yellow) spectra of **5**, DCM solution, 293 K, λ_{ex} = 465 nm. S81
Figure S84. Emission spectrum of **5**, DCM solution, 293 K, λ_{ex} = 465 nm, NIR detector....S82
Figure S85. Experimental time-resolved luminescence decay (yellow) with IRF (blue) of **3**, DCM solution, 293 K. Numerical biexponential reconvulation fit (purple). S83
Figure S86. Correlation of absorption (solid lines) and excitation (dotted lines) spectra of **1**, **3** and **5** in DCM. S84
Figure S87. Lifetime comparison of **1**, **3** and **5** in DCM solution together with IRF. S85
Figure S88. Emission spectrum of **1**, powder, 293 K, λ_{ex} = 490 nm. S86
Figure S89. Emission spectrum of **1**, powder, 293 K, λ_{ex} = 490 nm, NIR detector....S87
Figure S90. Experimental time-resolved luminescence decay (yellow) with IRF (blue) of **1**, powder, 293 K. Numerical triexponential reconvulation fit (purple). S88
Figure S91. Emission spectrum of **3**, powder, 293 K, λ_{ex} = 480 nm. S89
Figure S92. Emission spectrum of **3**, powder, 293 K, λ_{ex} = 420 nm. S90
Figure S93. Emission spectrum of **3**, powder, 293 K, λ_{ex} = 470 nm, NIR detector....S91
Figure S94. Experimental time-resolved luminescence decay (yellow) with IRF (blue) of **3**, powder, 293 K. Numerical biexponential reconvulation fit (purple). S92
Figure S95. Emission spectrum of **5**, powder, 293 K, λ_{ex} = 465 nm. S93
Figure S96. Emission spectrum of **5**, powder, 293 K, λ_{ex} = 470 nm, NIR detector....S94
Figure S97. Experimental time-resolved luminescence decay (yellow) with IRF (blue) of **5**, powder, 293 K. Numerical triexponential reconvulation fit (purple). S95
Figure S98. Lifetime comparison of **1**, **3** and **5** in powder together with IRF. S96
Figure S99. Blue Lamp (467 nm) for irradiation of **1** in DCM or powder. 12 h, RT. S97

Characterization of the Complexes: Spectroscopic data (NMR, MS, FT-IR, UV/VIS)

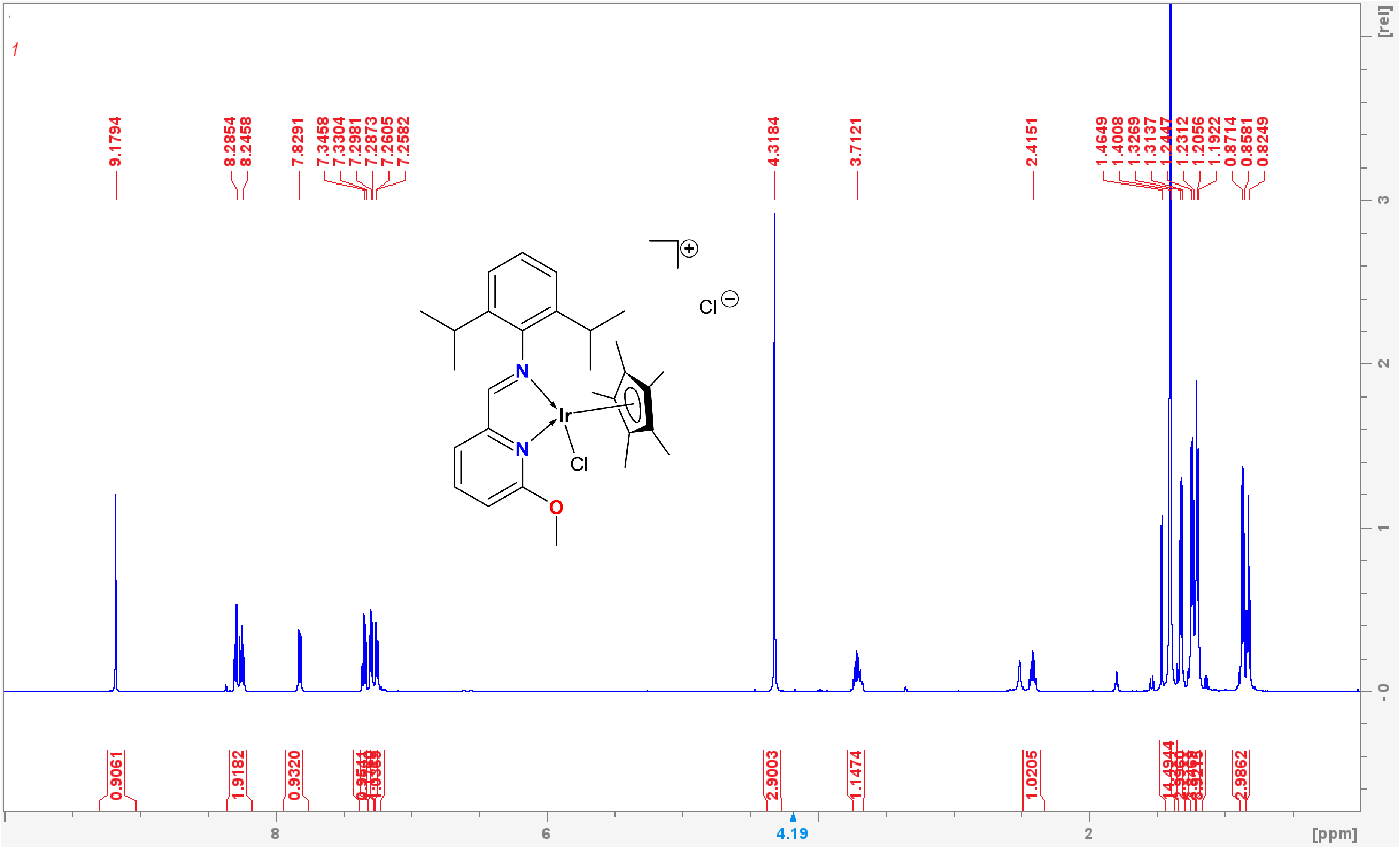

Figure S1. ^{1}H NMR ($CDCl_3$, 500.13 MHz, 300 K) of **1**.

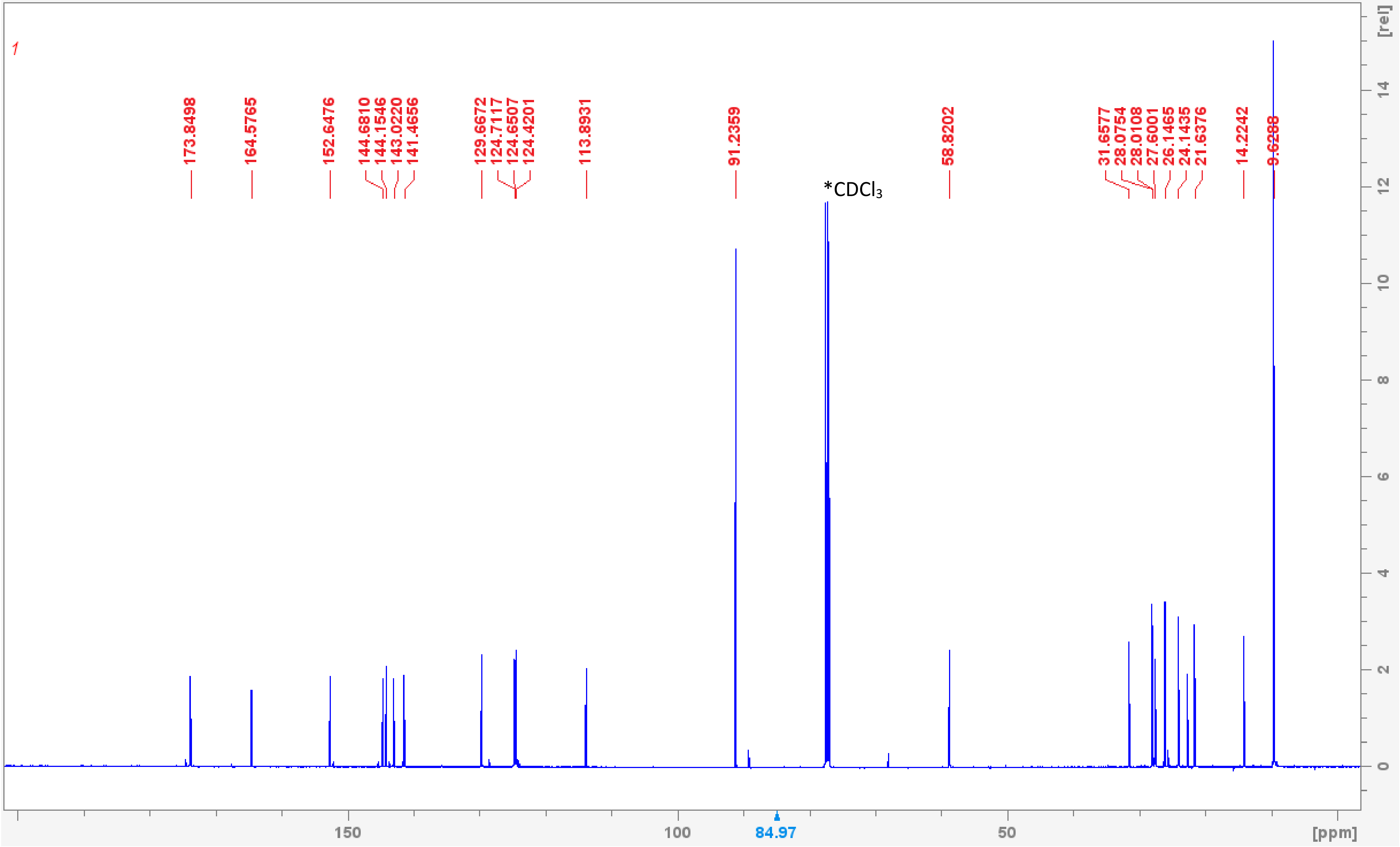

Figure S2. $^{13}C\{^{1}H\}$ NMR ($CDCl_3$, 125.76 MHz, 300 K) of **1**.

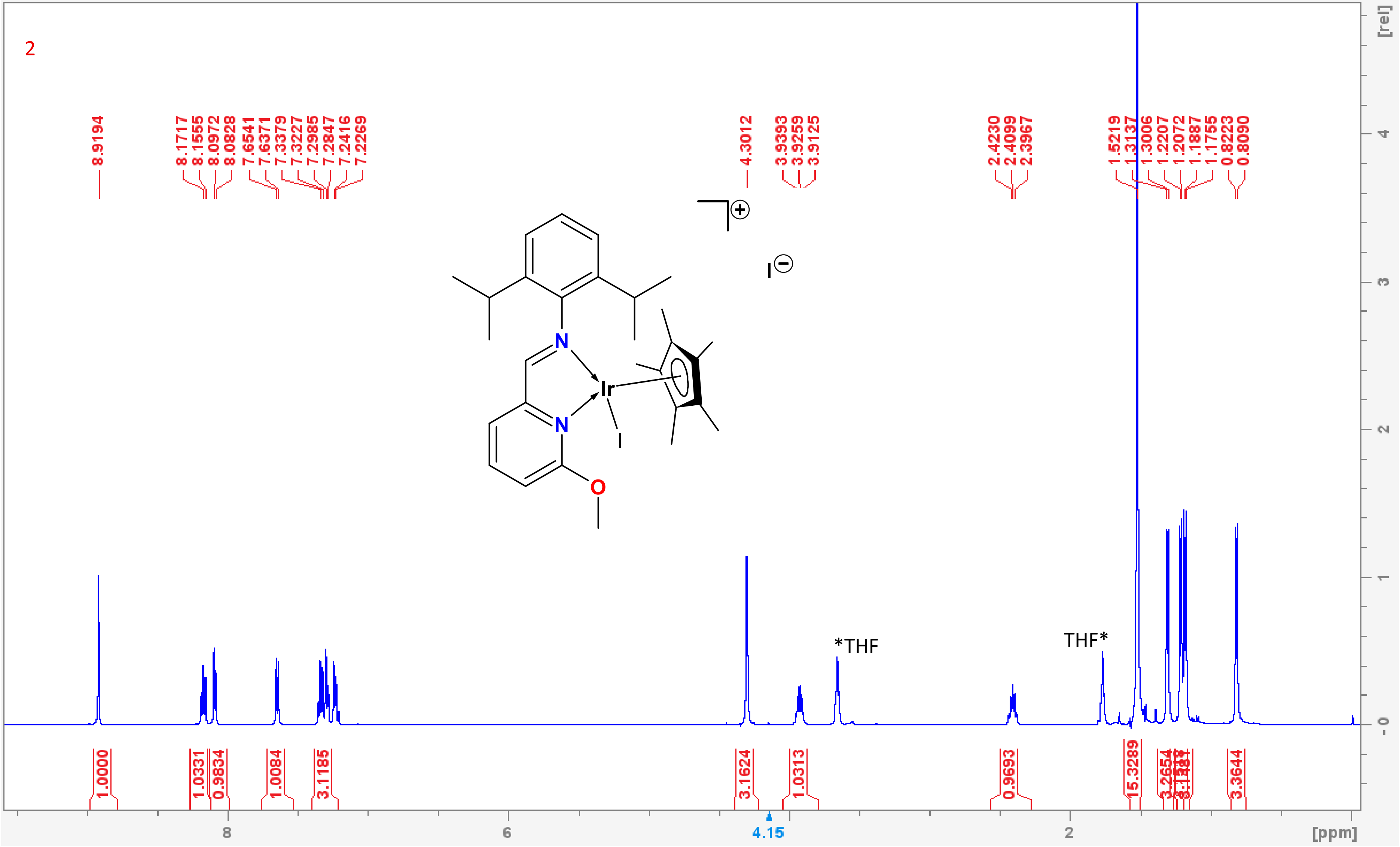

Figure S3. ^{1}H NMR ($CDCl_3$, 500.13 MHz, 300 K) of **2.**

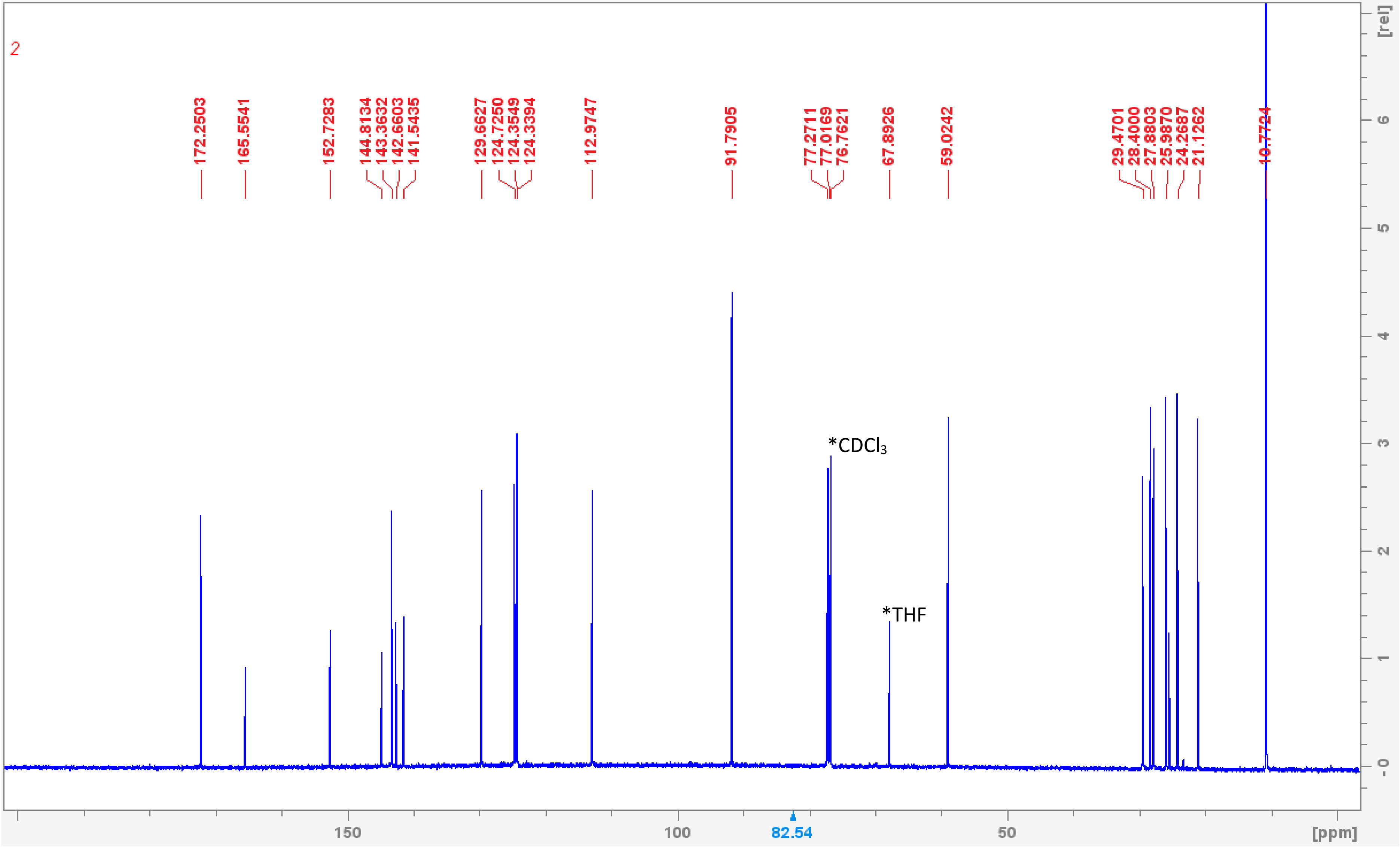

Figure S4. ${}^{13}C\{{}^{1}H\}$ NMR ($CDCl_3$ 125.78 MHz, 300 K) of **2**.

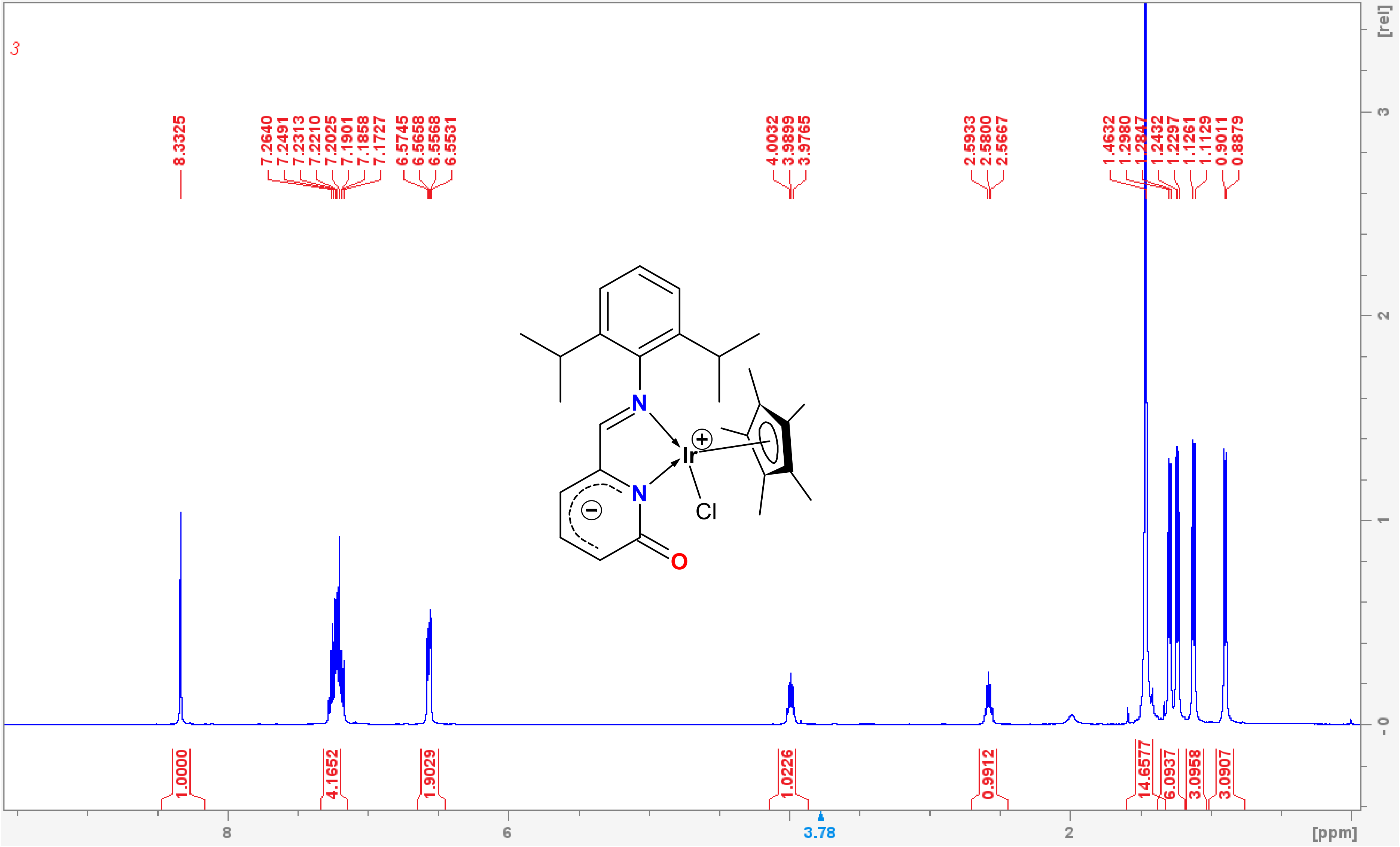

Figure S5. ^{1}H NMR ($CDCl_3$, 500.13 MHz, 300 K) of **3.**

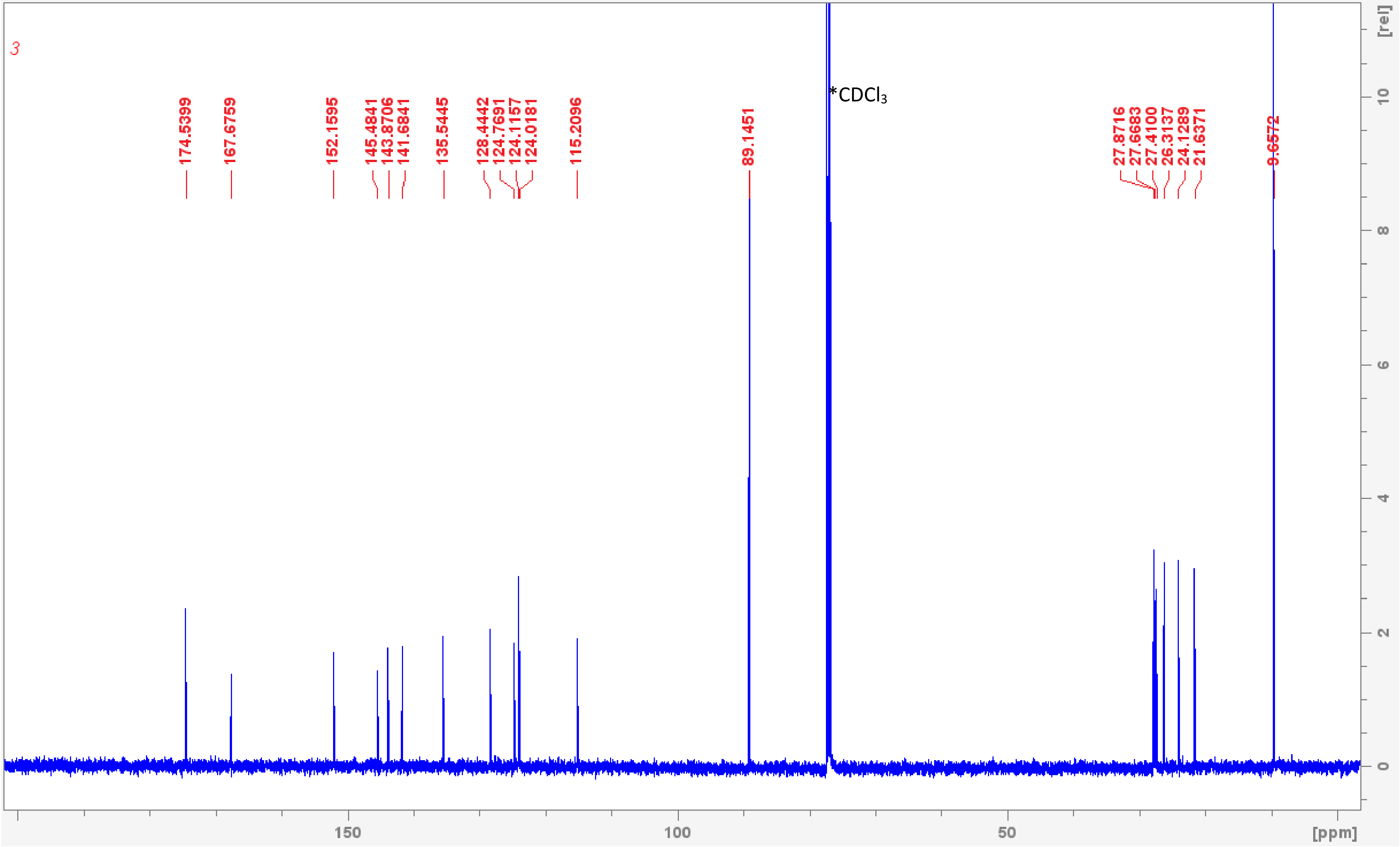

Figure S6. $^{13}C\{^{1}H\}$ NMR ($CDCl_3$, 125.76 MHz, 300 K) of **3**.

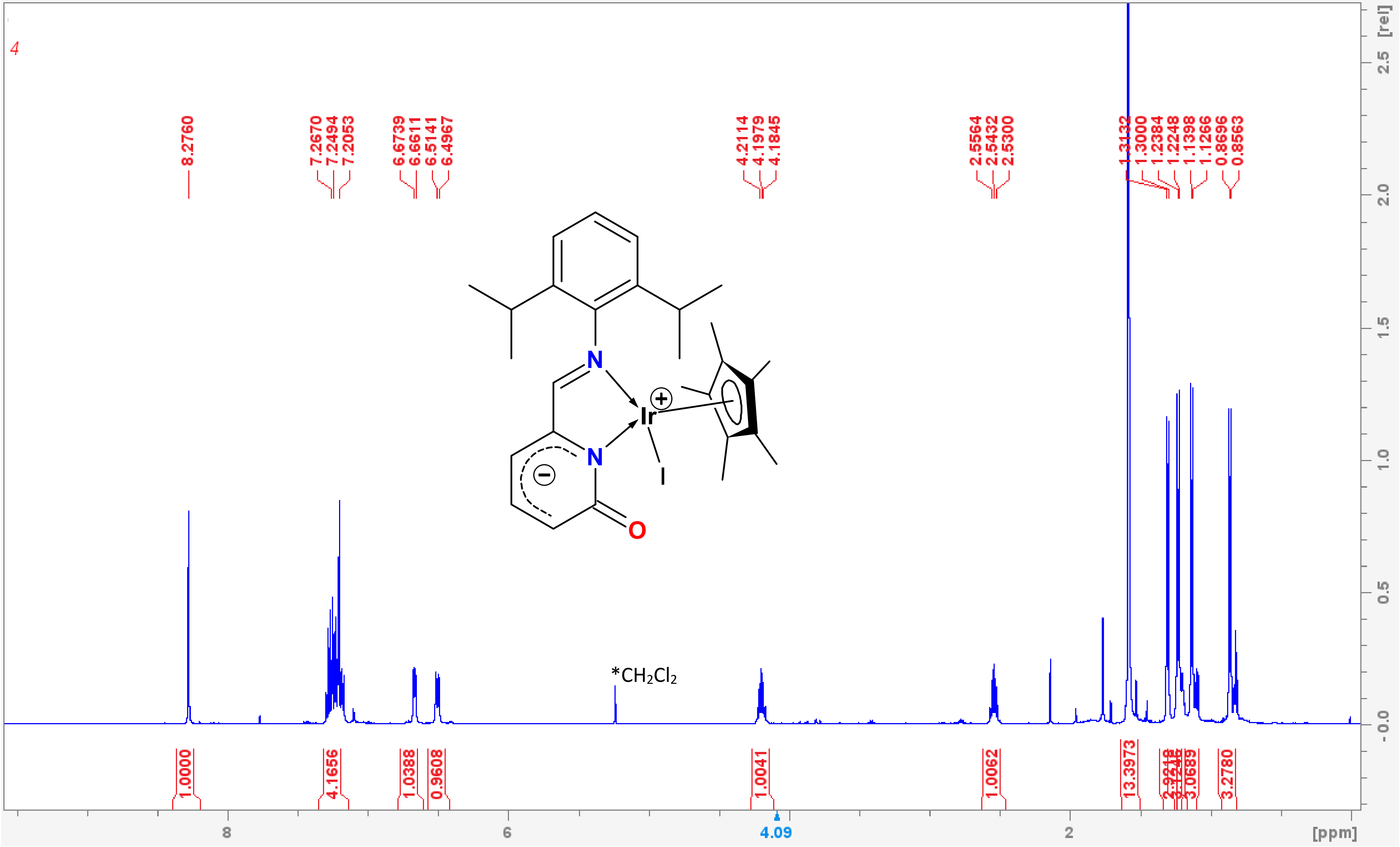

Figure S7. ^{1}H NMR ($CDCl_3$, 500.13 MHz, 300 K) of **4.**

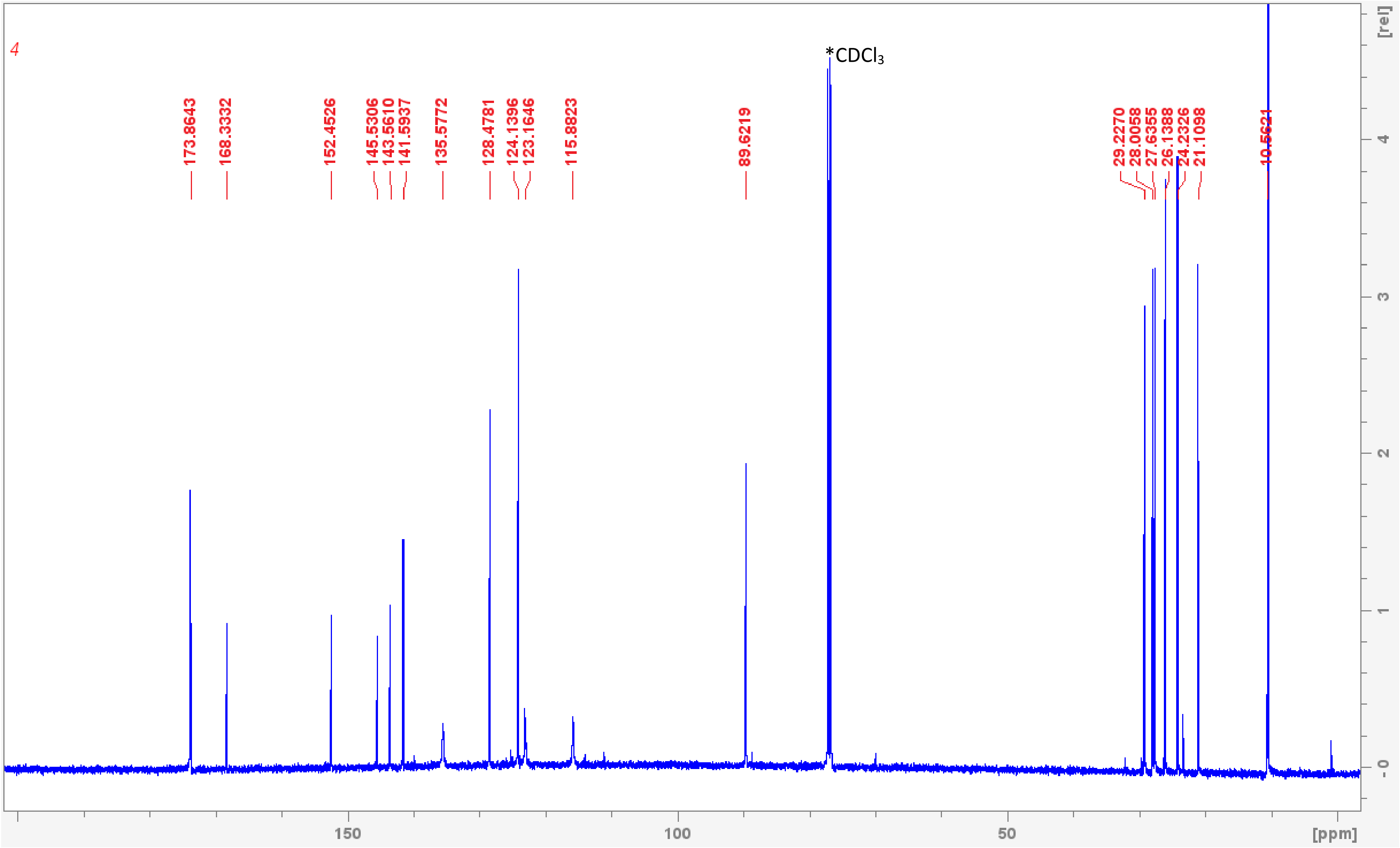

Figure S8. $^{13}C\{^1H\}$ NMR ($CDCl_3$, 125.78 MHz, 300 K) of **4.**

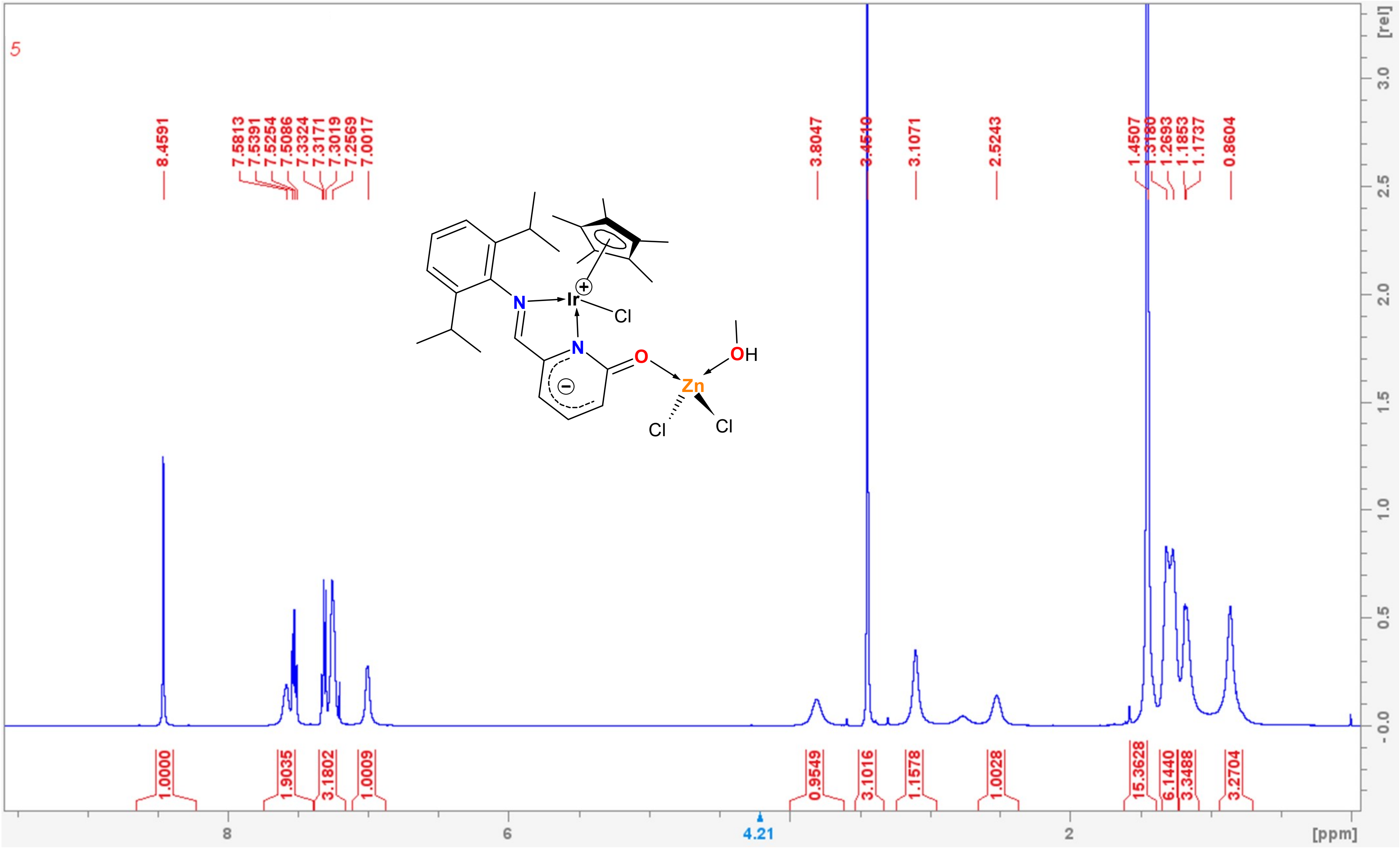

Figure S9. ^{1}H NMR ($CDCl_3$, 500.13 MHz, 300 K) of **5.**

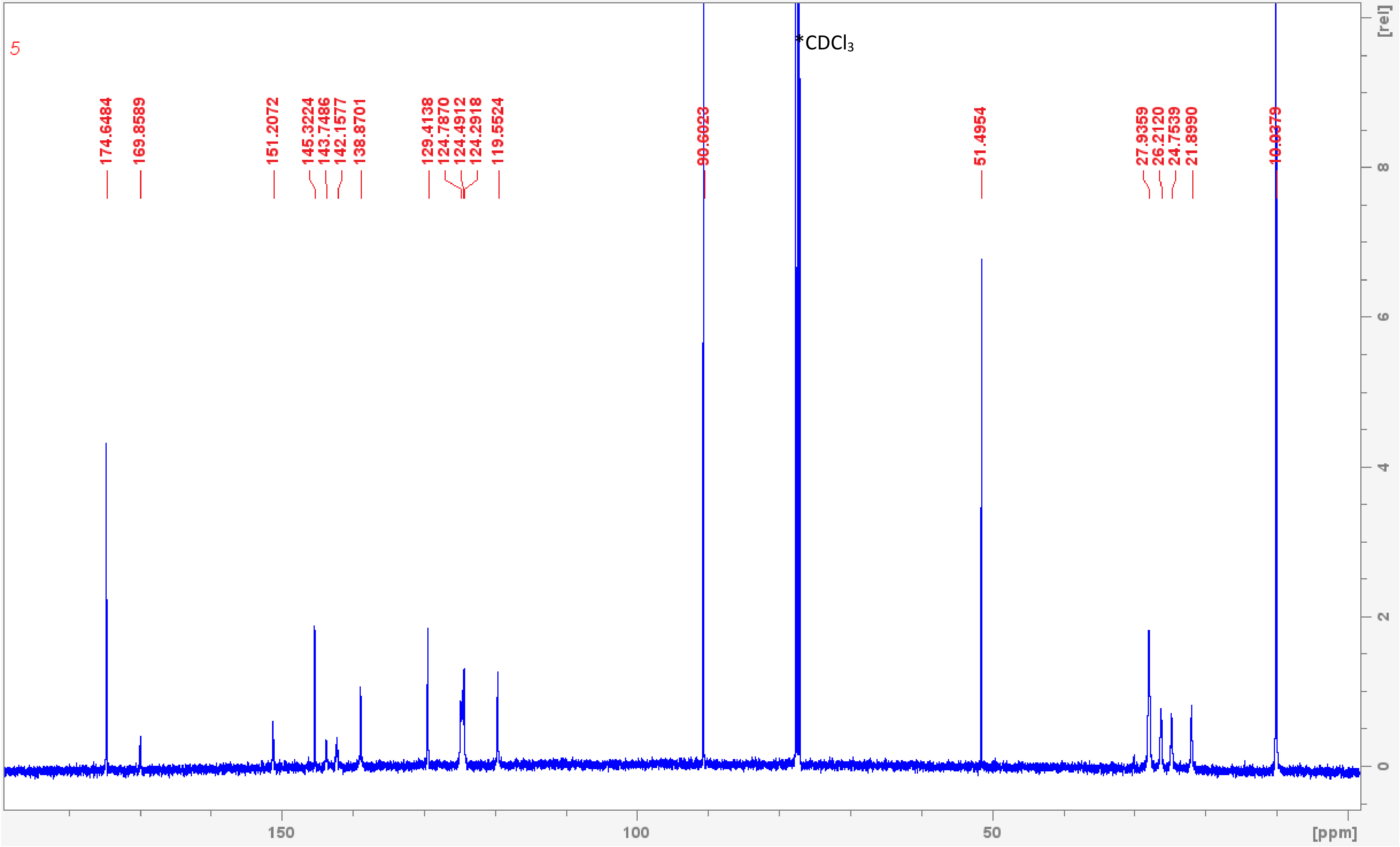

Figure S10. $^{13}C\{^1H\}$ NMR ($CDCl_3$, 125.78 MHz, 300 K) of **5.**

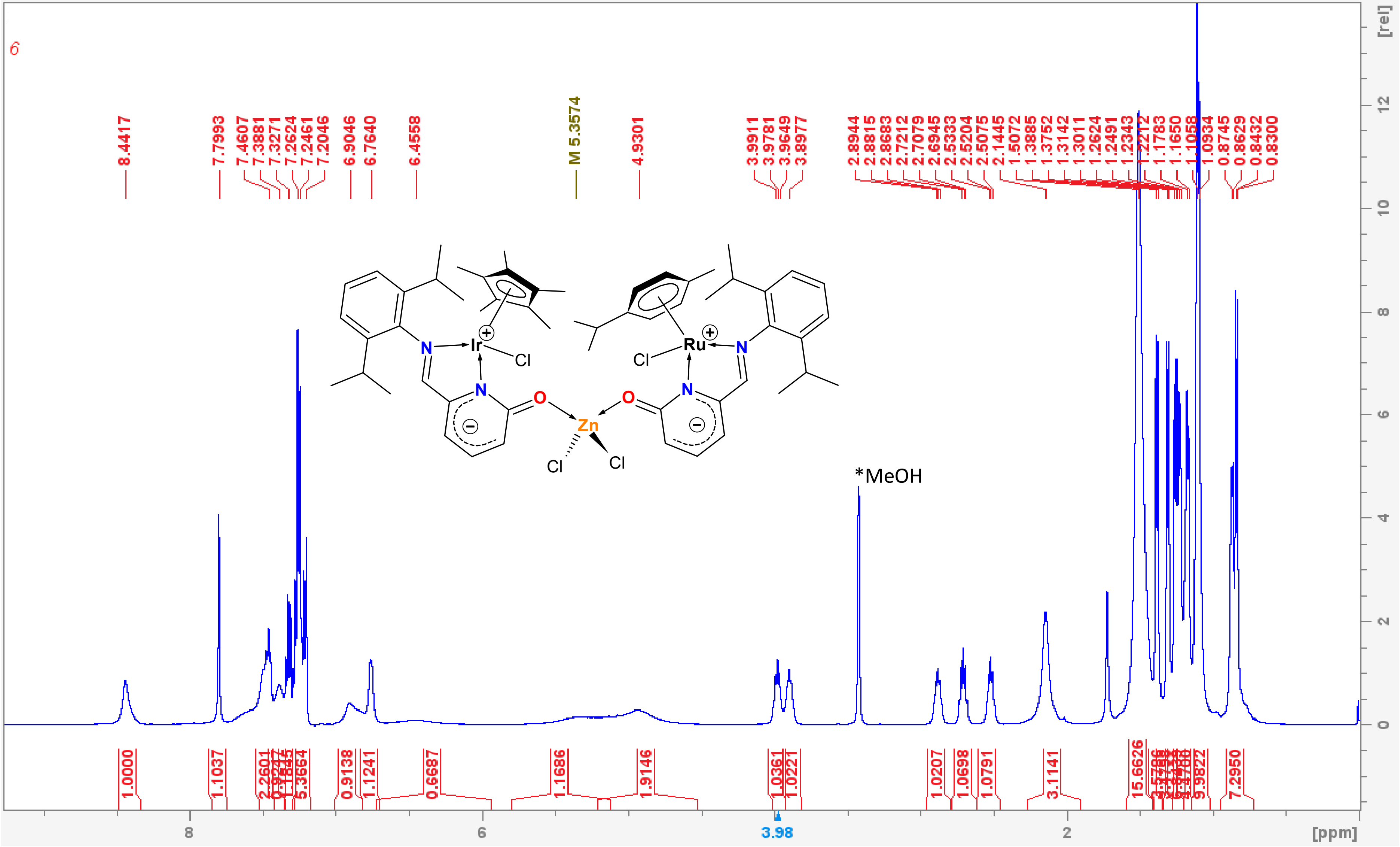

Figure S11. ^{1}H NMR ($CDCl_3$, 500.13 MHz, 300 K) of **6.**

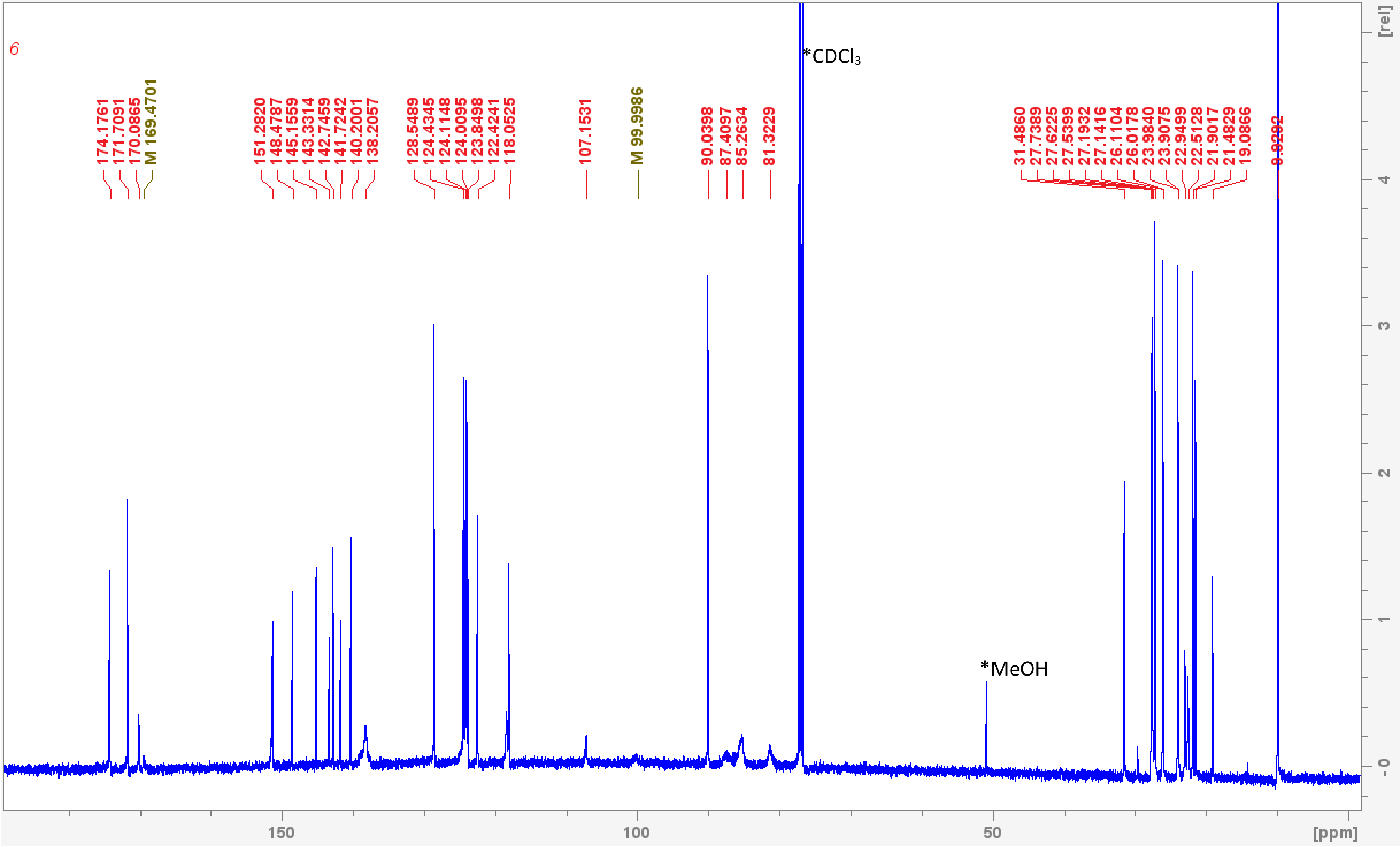

Figure S12. $^{13}C\{^{1}H\}$ NMR ($CDCl_3$, 125.78 MHz, 300 K) of **6.**

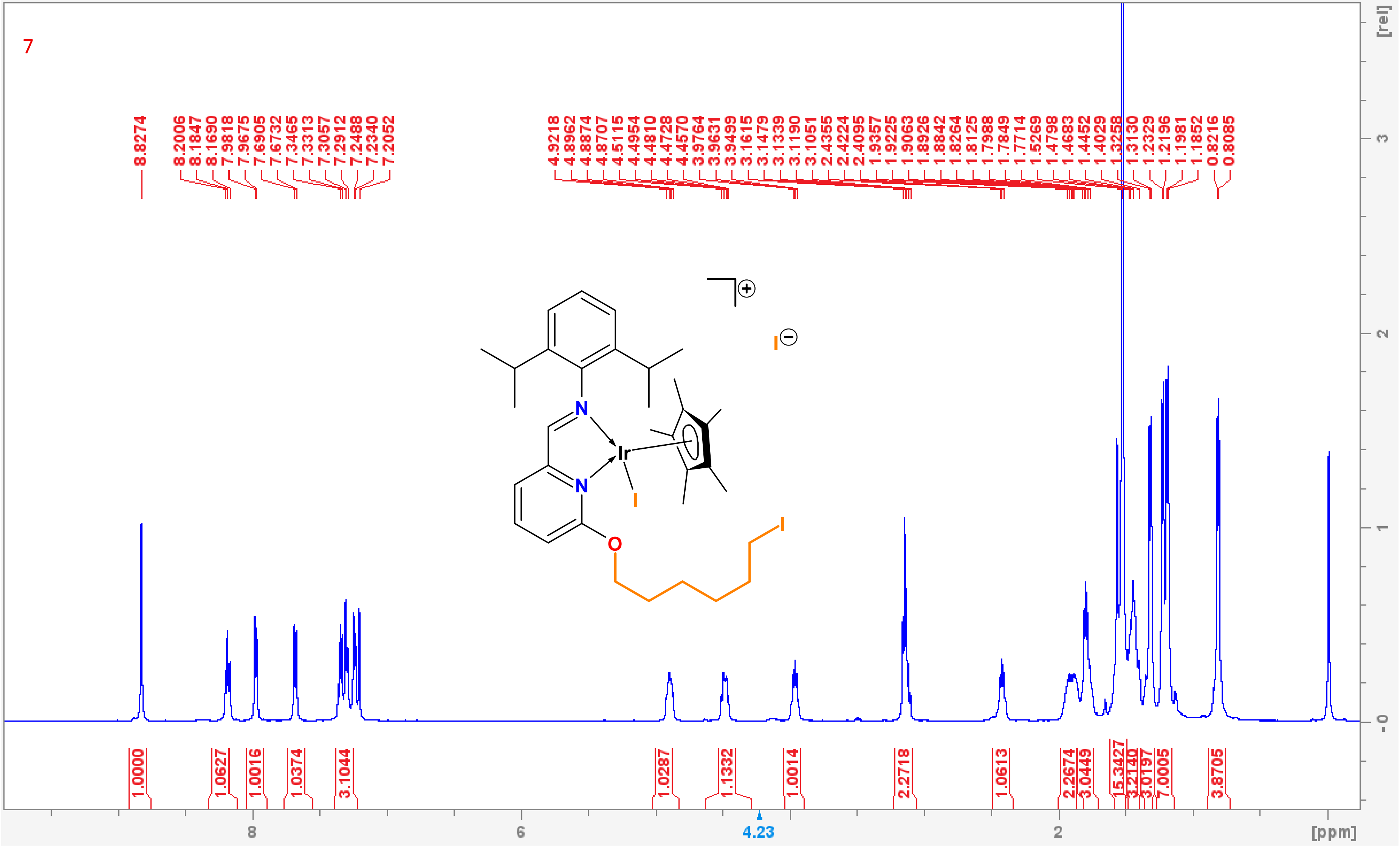

Figure S13. ^{1}H NMR ($CDCl_3$, 500.13 MHz, 300 K) of **7.**

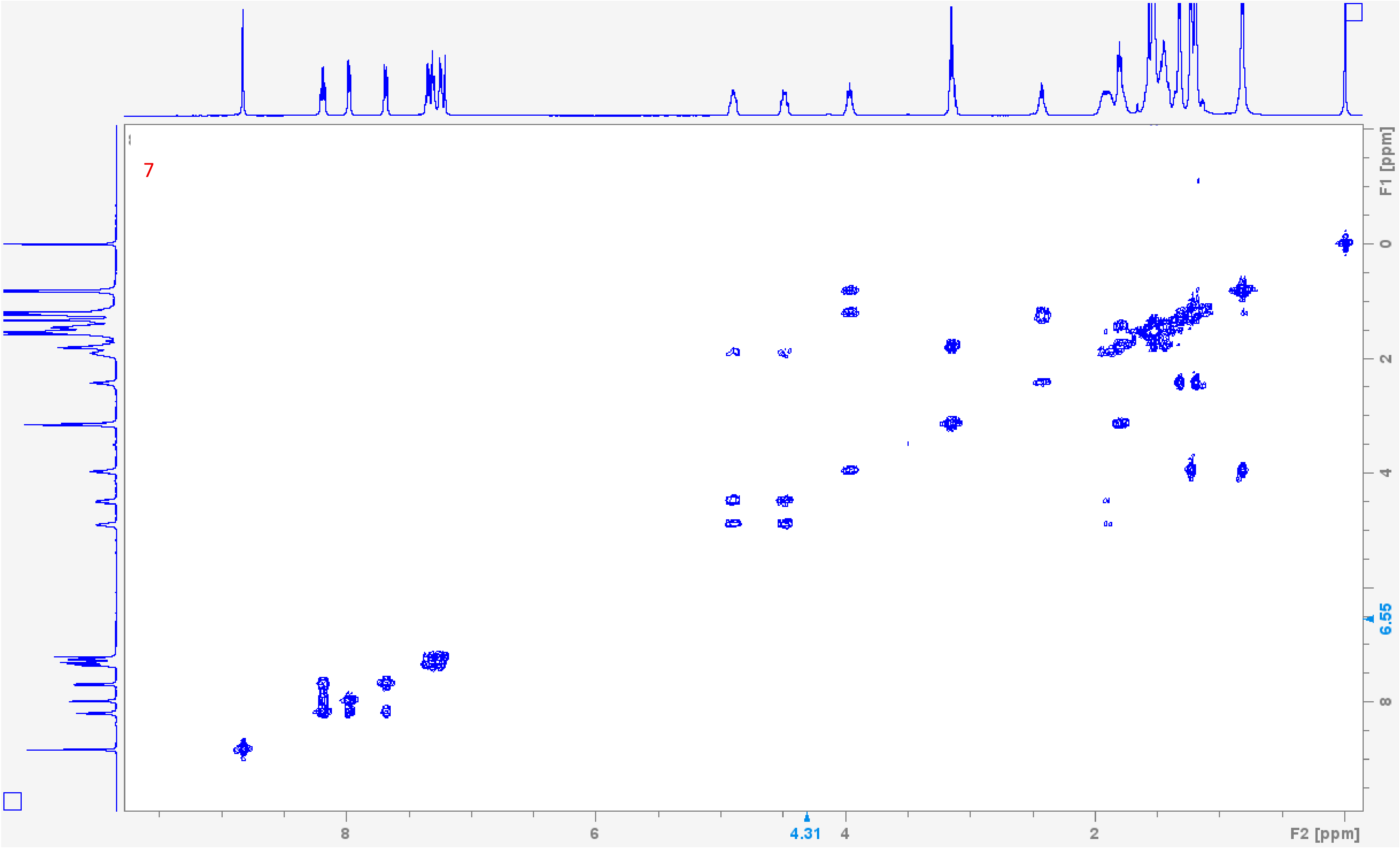

Figure S14. ^{1}H-^{1}H COSY NMR ($CDCl_3$, 500.13 MHz, 300 K) of **7.**

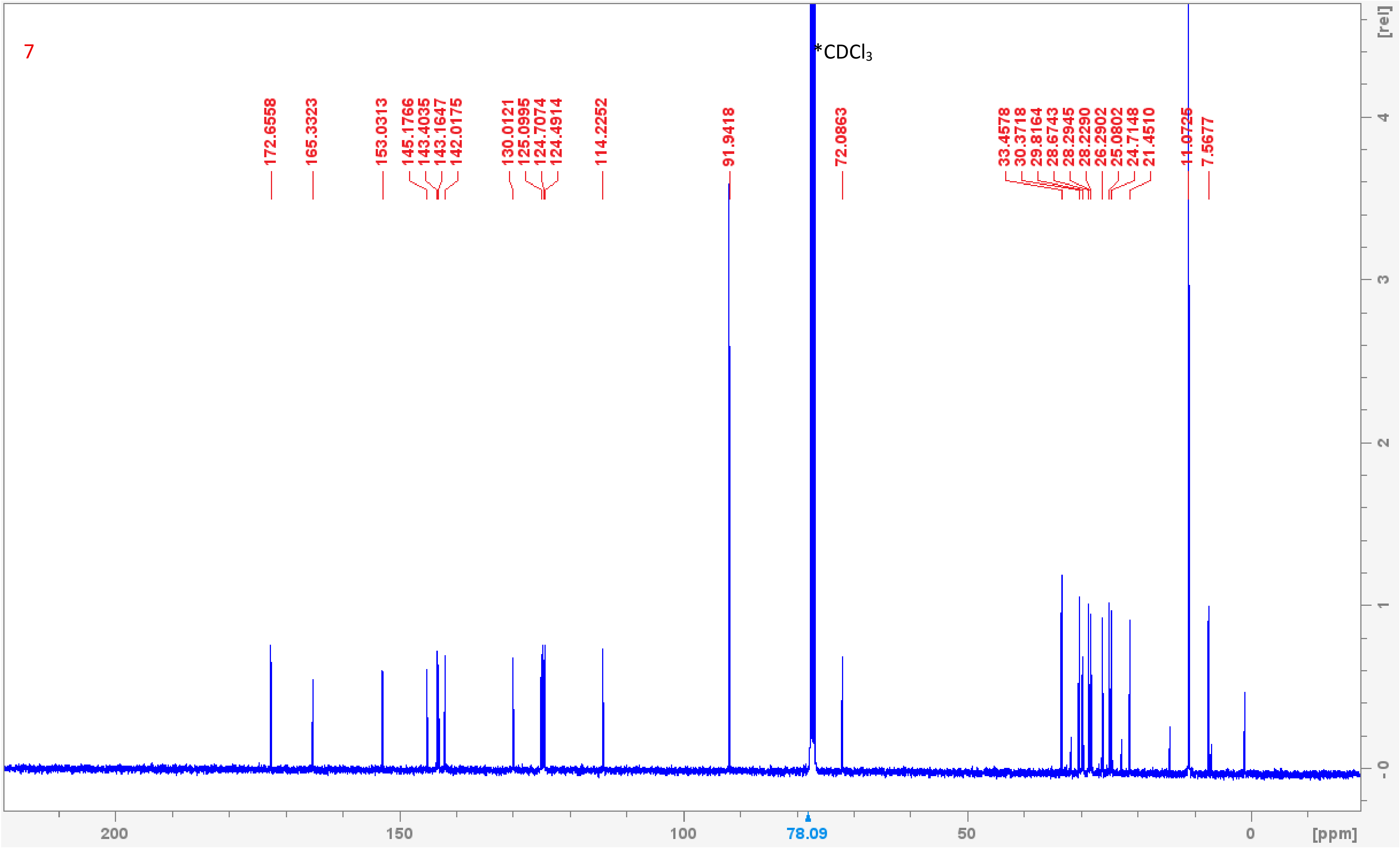

Figure S15. $^{13}C\{^{1}H\}$ NMR ($CDCl_3$, 100.61 MHz, 300 K) of **7.**

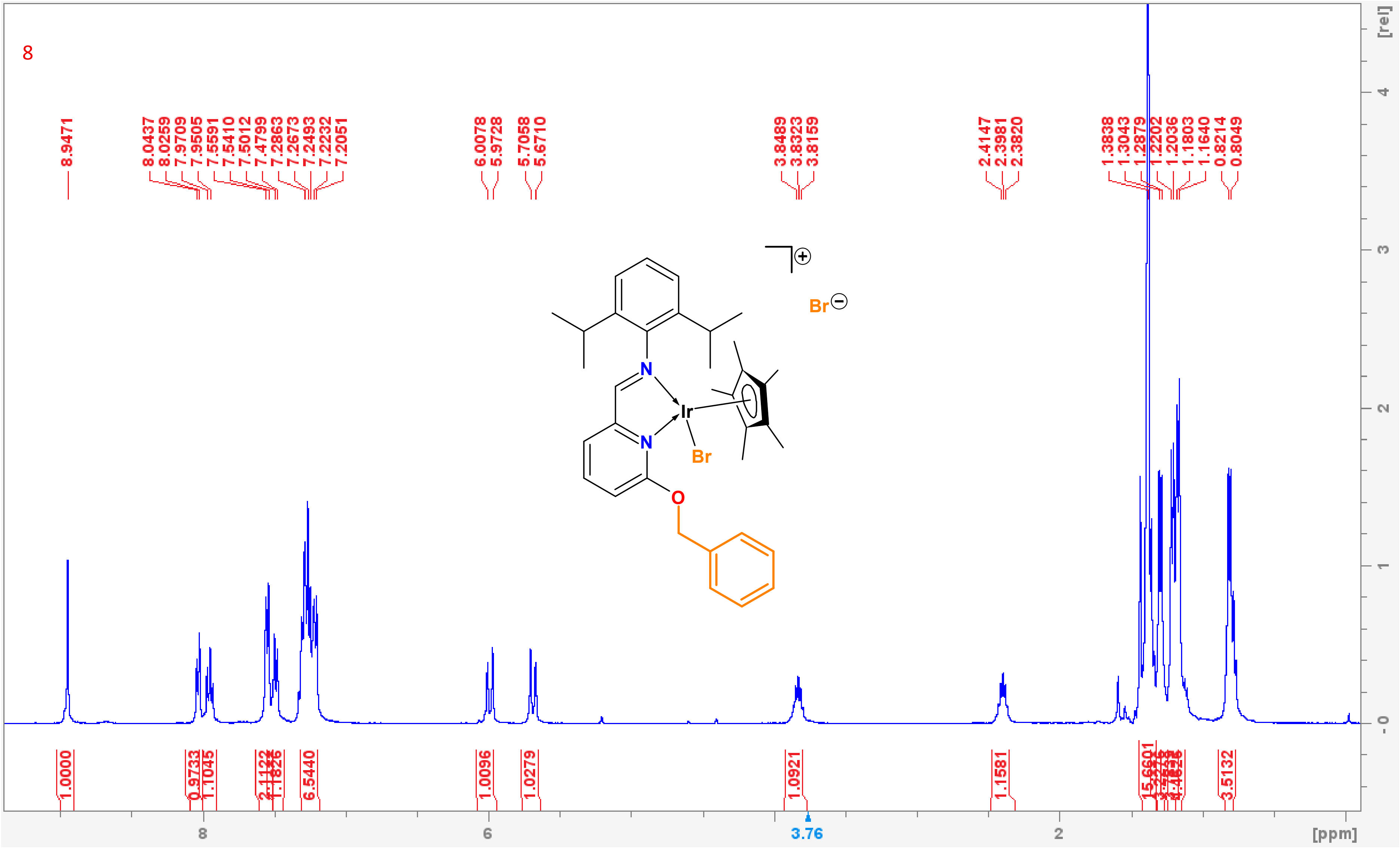

Figure S16. ^{1}H NMR ($CDCl_3$, 400.13 MHz, 300 K) of **8.**

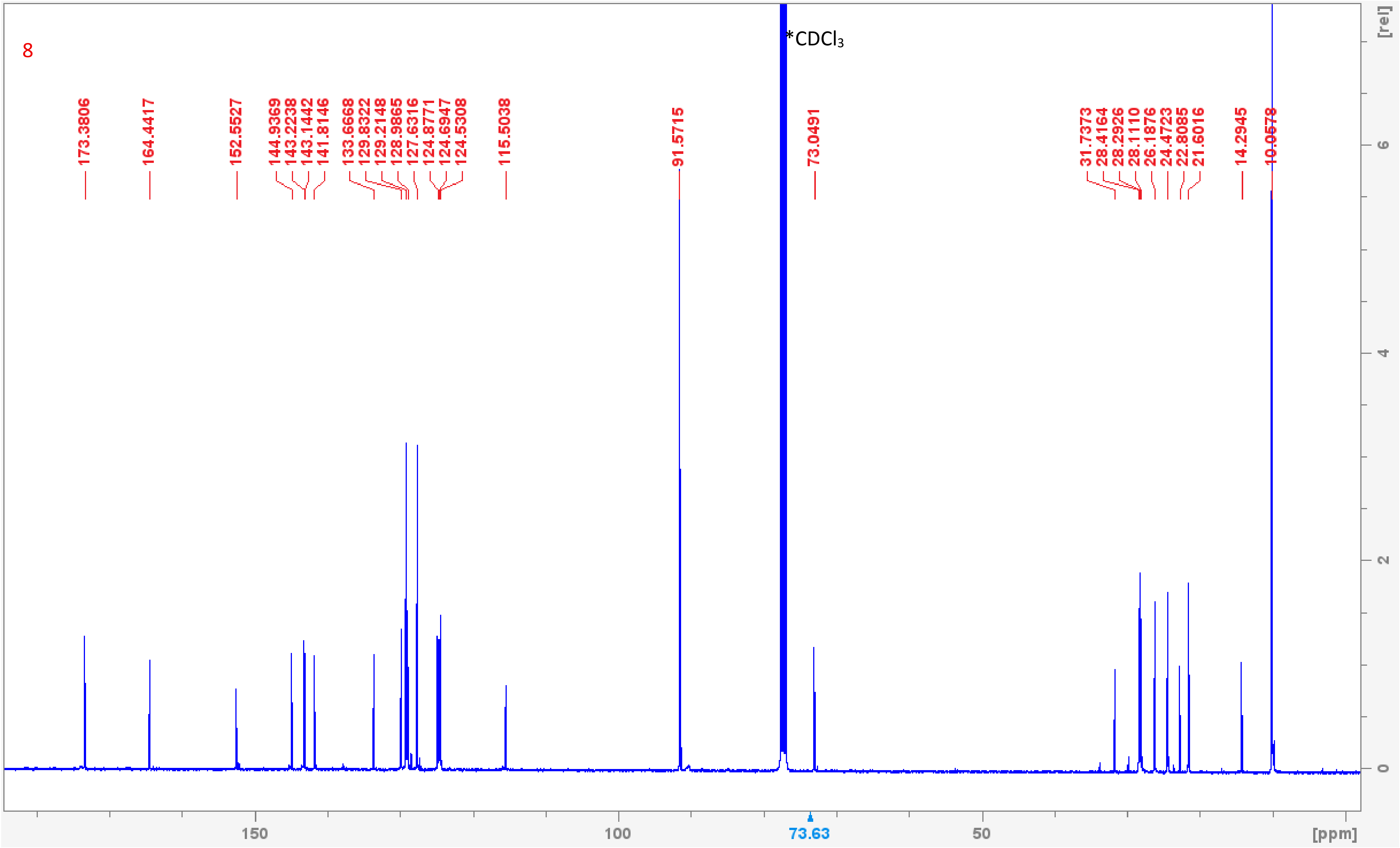

Figure S17. ${}^{13}C\{{}^{1}H\}$ NMR ($CDCl_3$, 100.61 MHz, 300 K) of **8**.

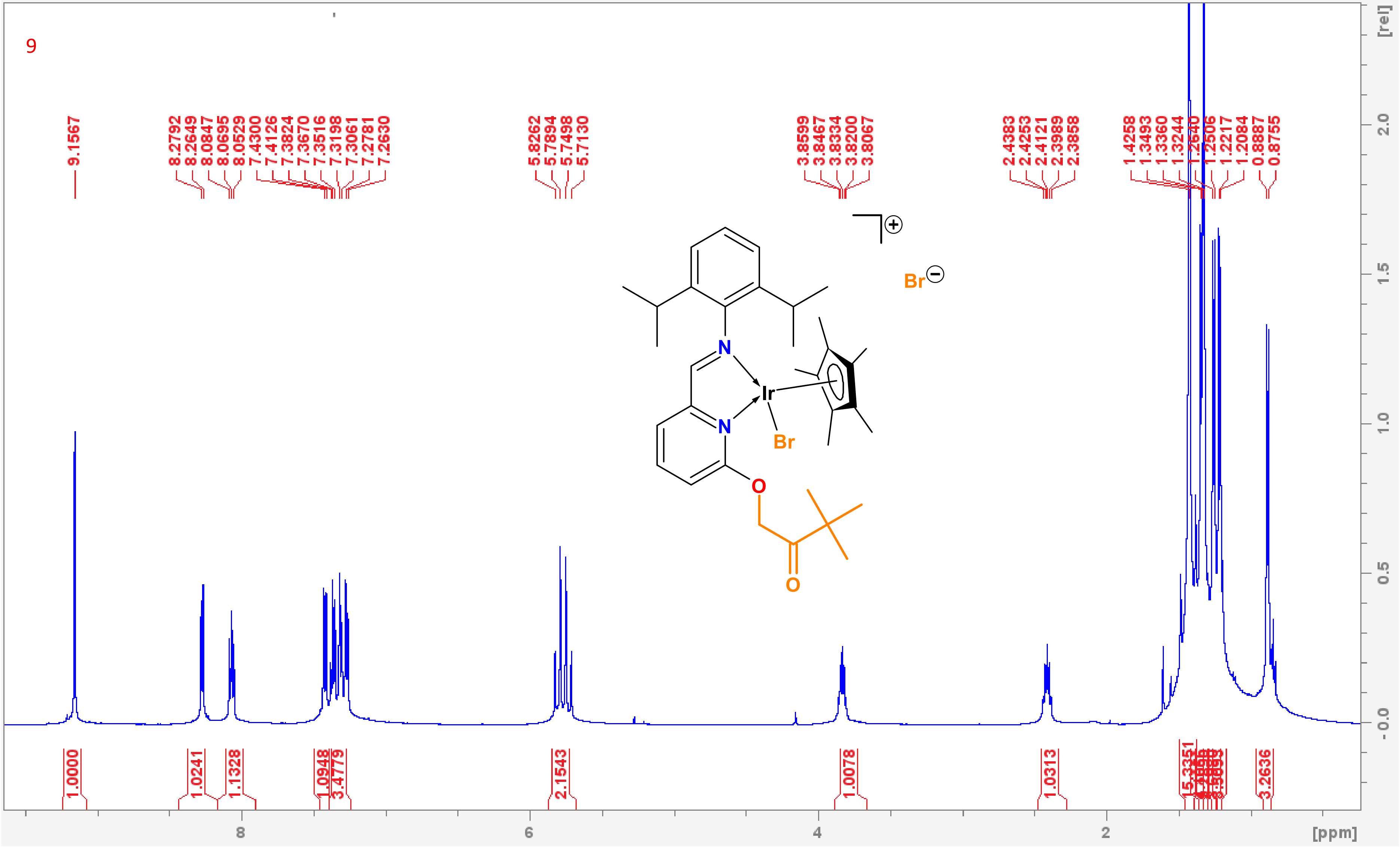

Figure S18. ^{1}H NMR ($CDCl_3$, 500.13 MHz, 300 K) of **9**.

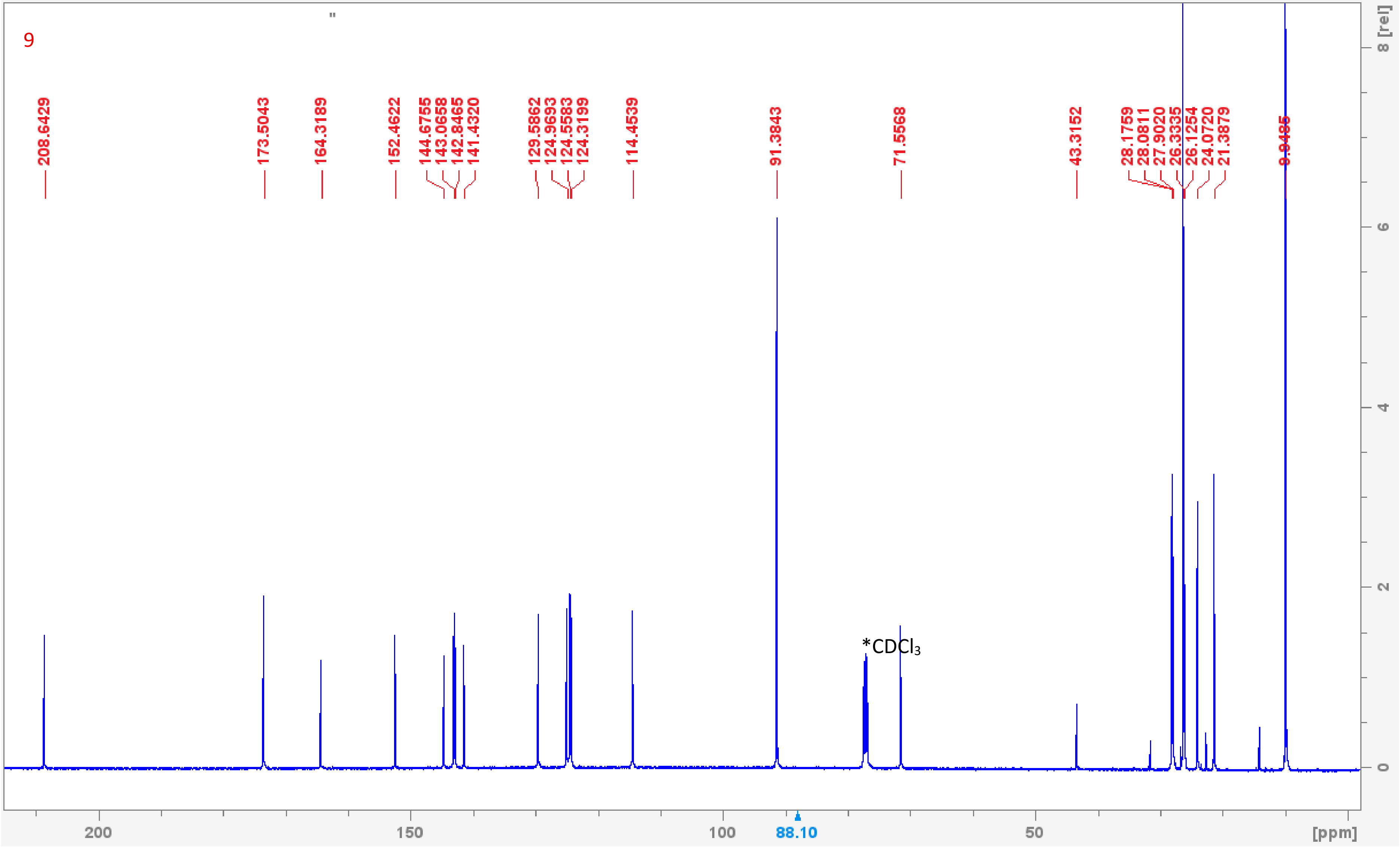

Figure S19. ${}^{13}C\{{}^{1}H\}$ NMR ($CDCl_3$, 125.78 MHz, 300 K) of **9**.

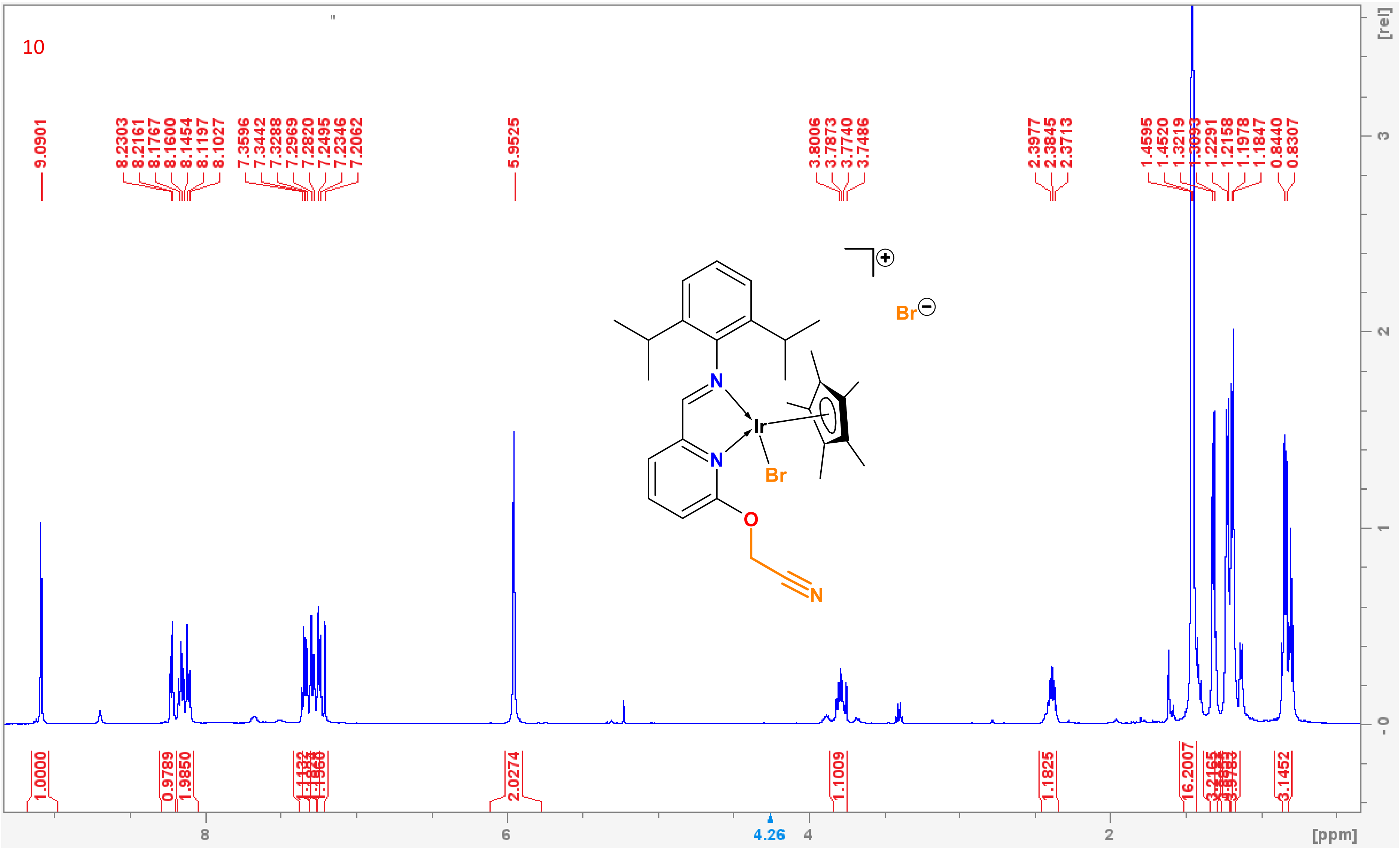

Figure S20. ^{1}H NMR ($CDCl_3$, 500.13 MHz, 300 K) of **10**.

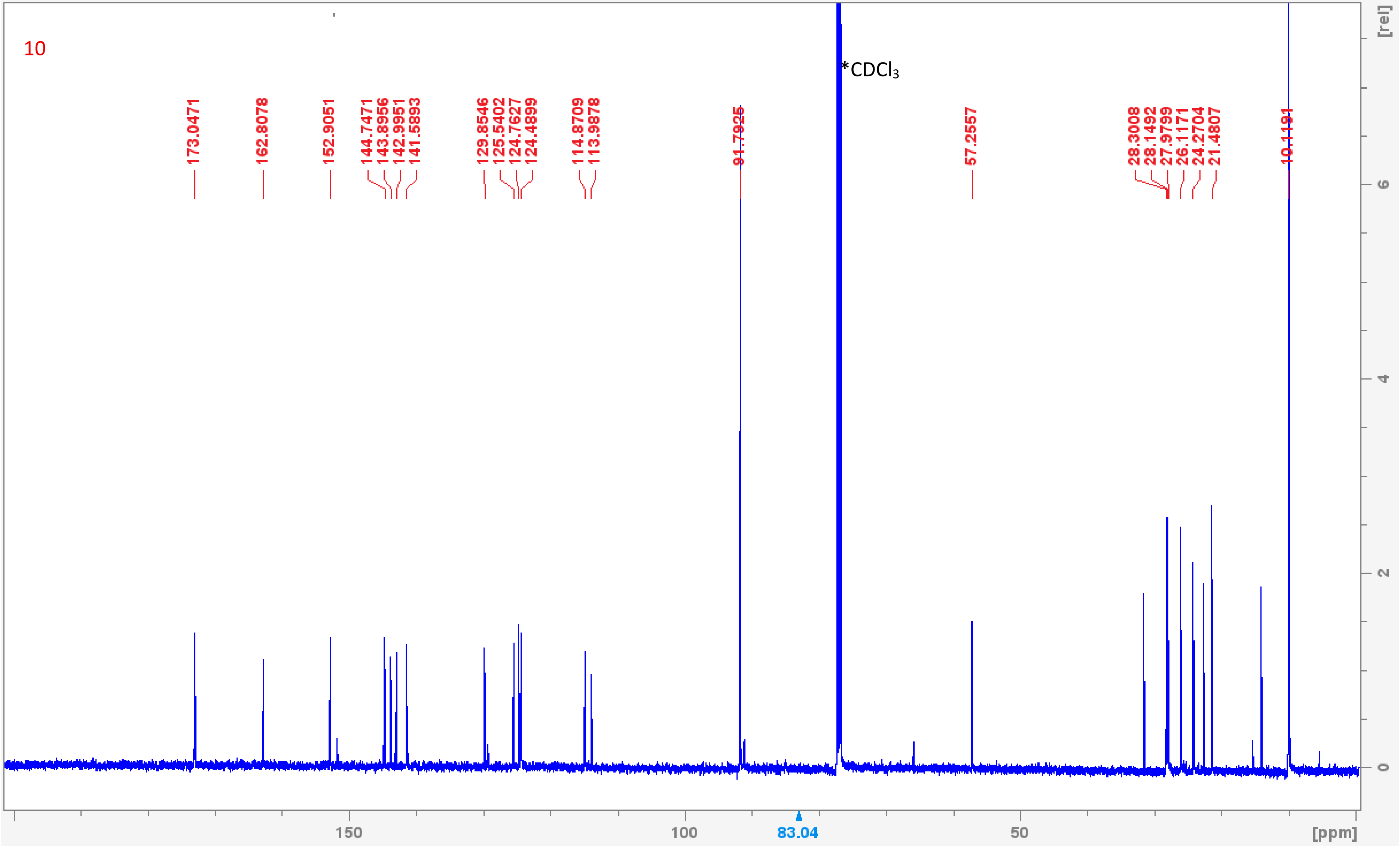

Figure S21. $^{13}C\{^{1}H\}$ NMR ($CDCl_3$, 125.76 MHz, 300 K) of **10**.

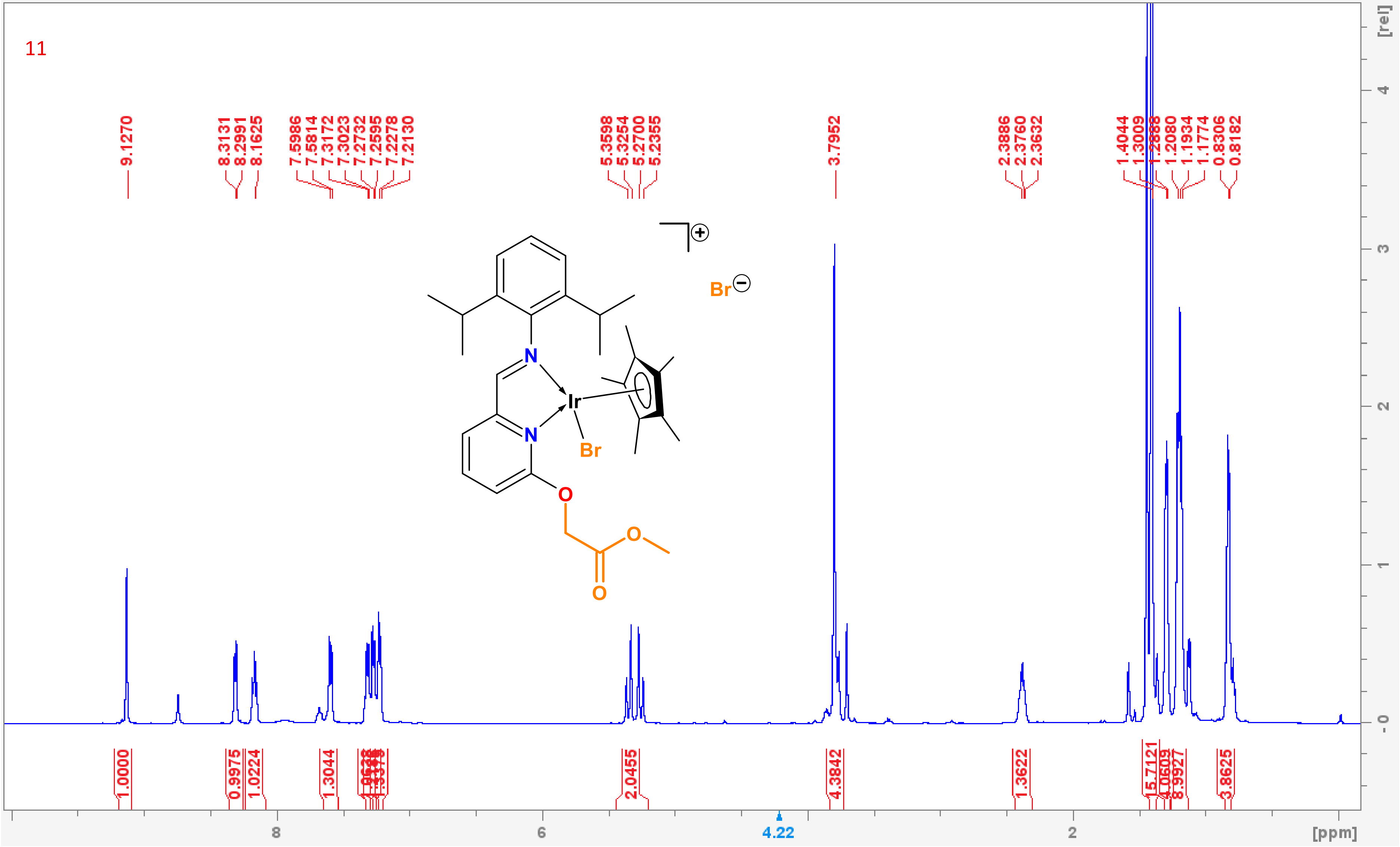

Figure S22. ^{1}H NMR ($CDCl_3$, 500.13 MHz, 300 K) of **11**.

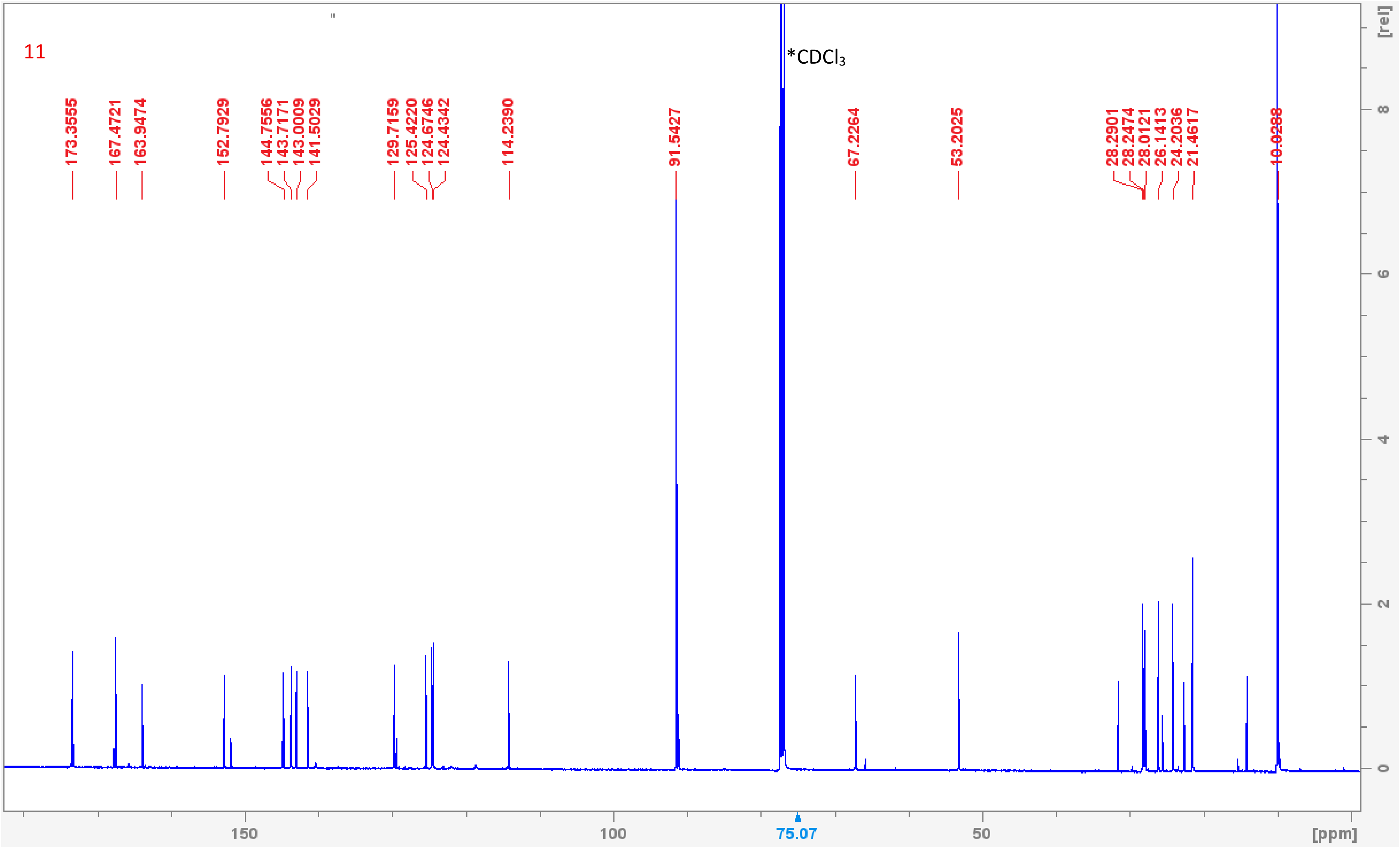

Figure S23. $^{13}C\{^{1}H\}$ NMR ($CDCl_3$, 125.76 MHz, 300 K) of **11**.

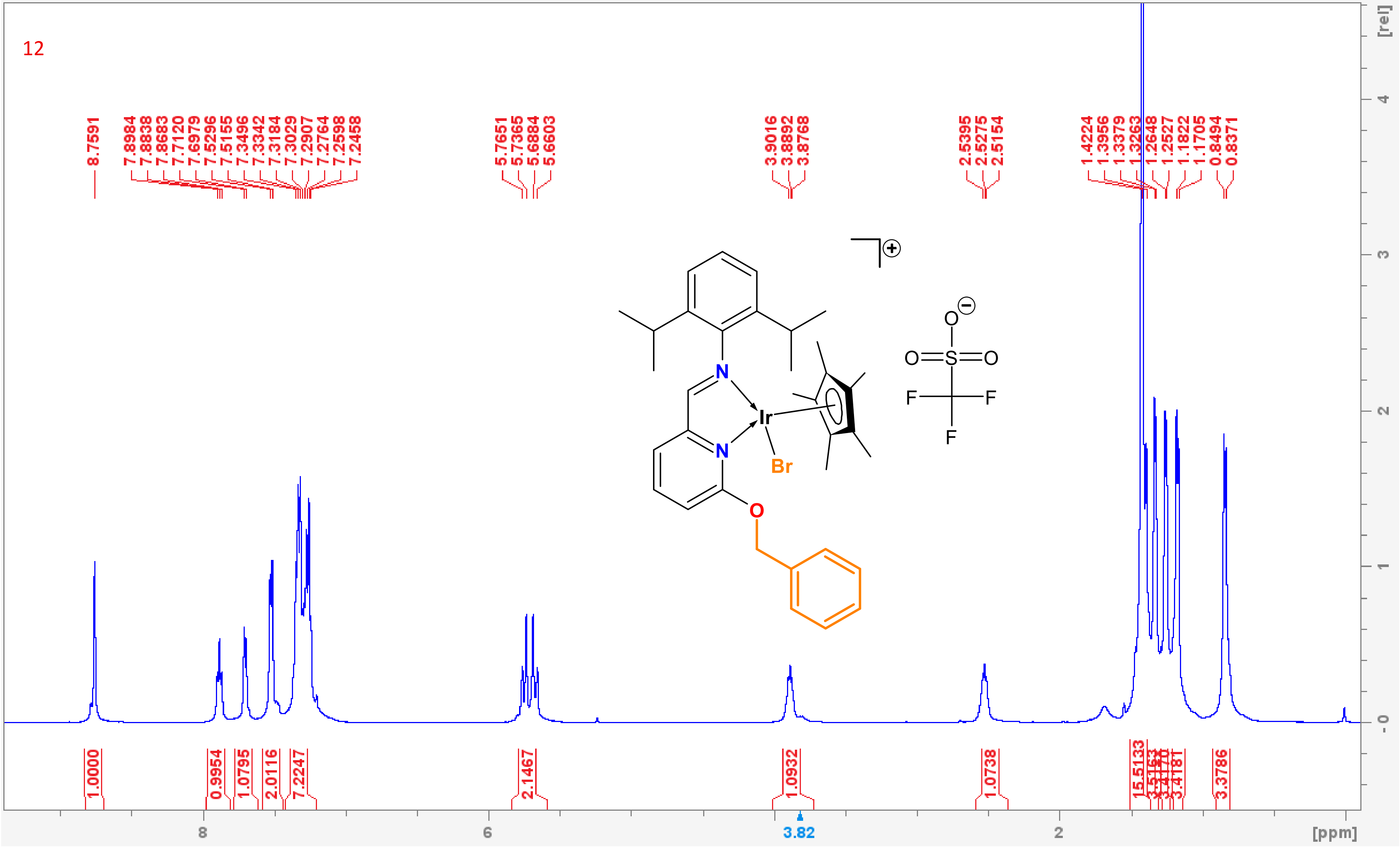

Figure S24. ^{1}H NMR ($CDCl_3$, 500.13 MHz, 300 K) of **12**.

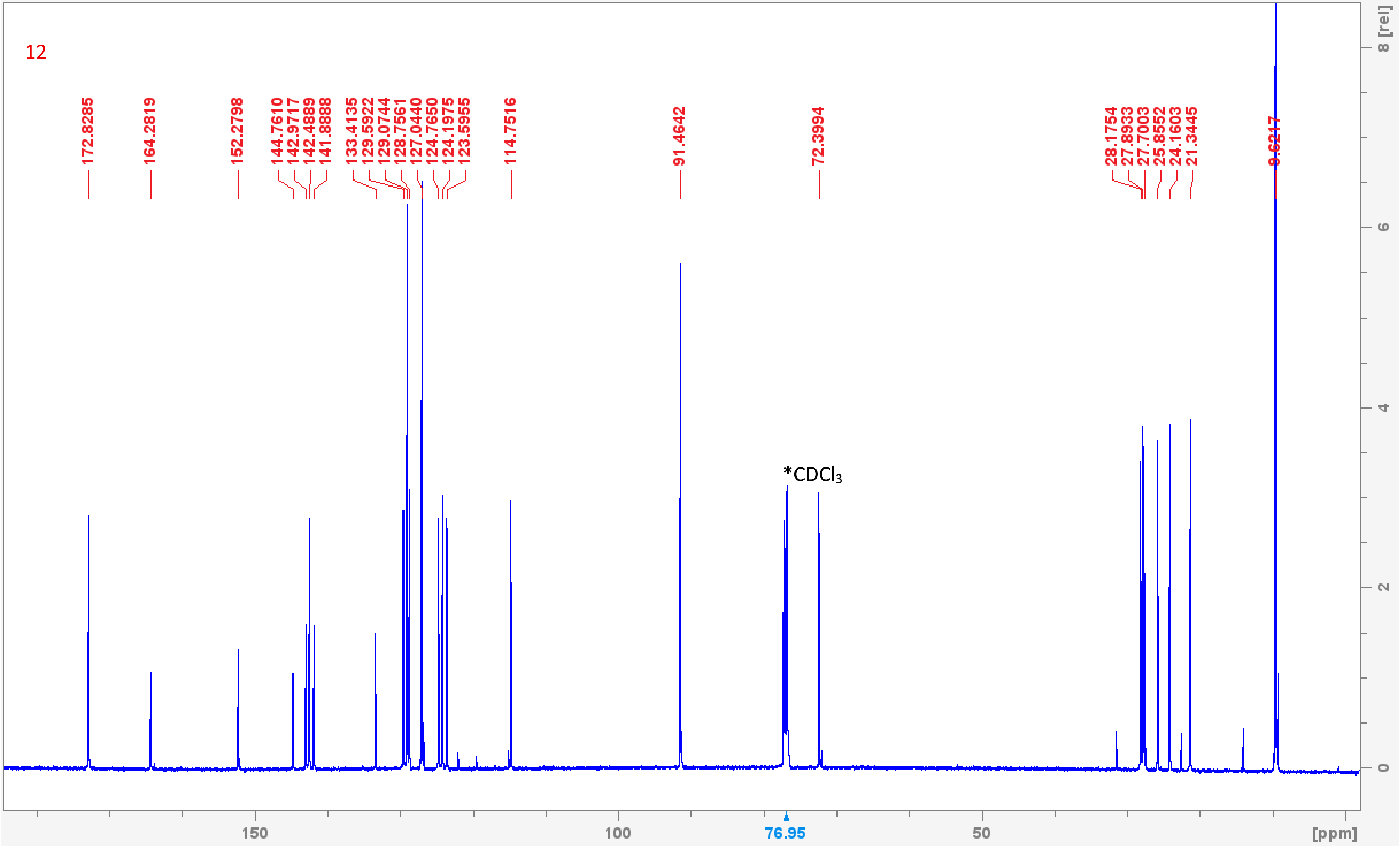

Figure S25. $^{13}C\{^1H\}$ NMR ($CDCl_3$, 125.78 MHz, 300 K) of **12**.

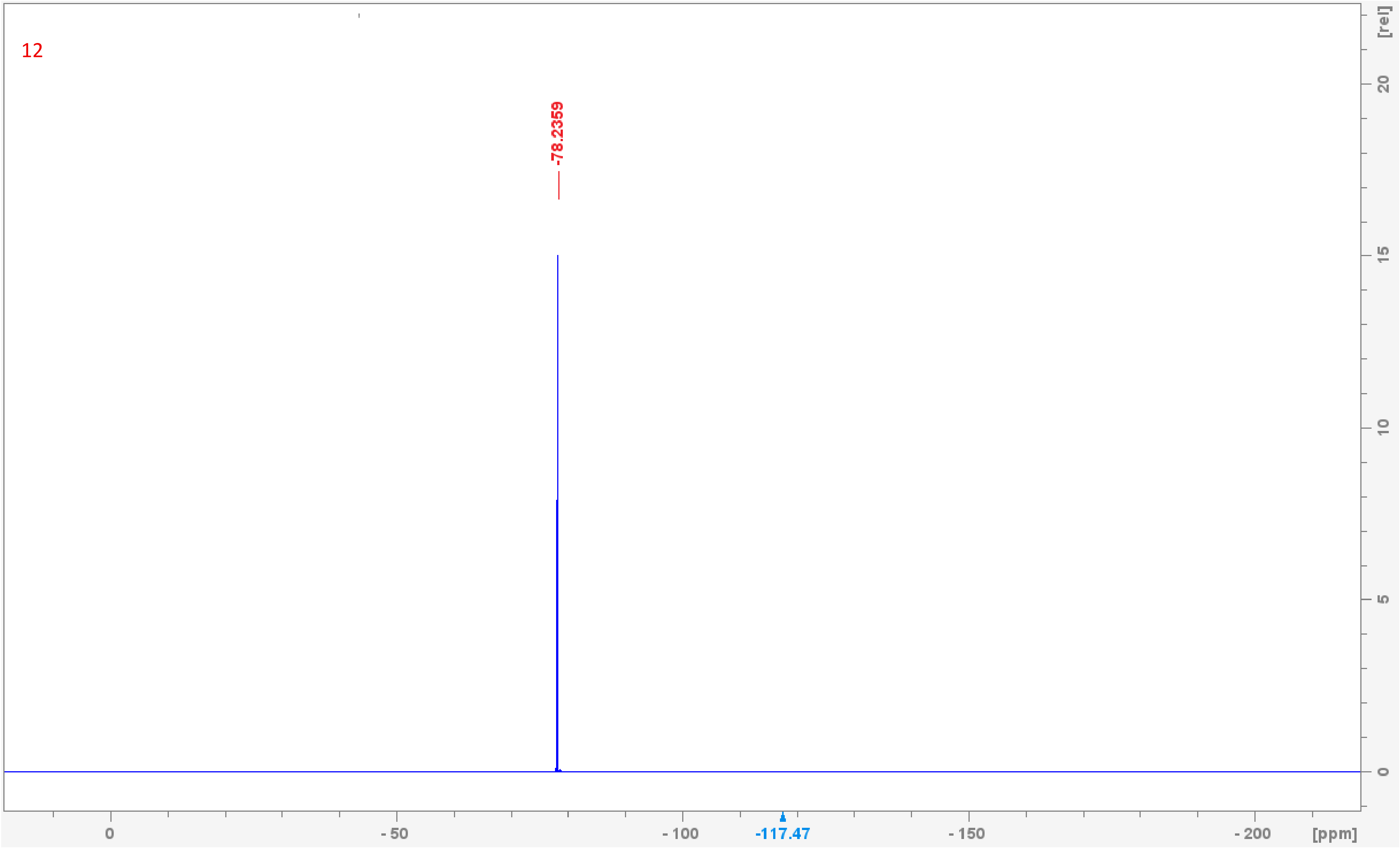

Figure S26. $^{19}F\{^{1}H\}$ NMR ($CDCl_3$, 376.50 MHz, 300 K) of **12**.

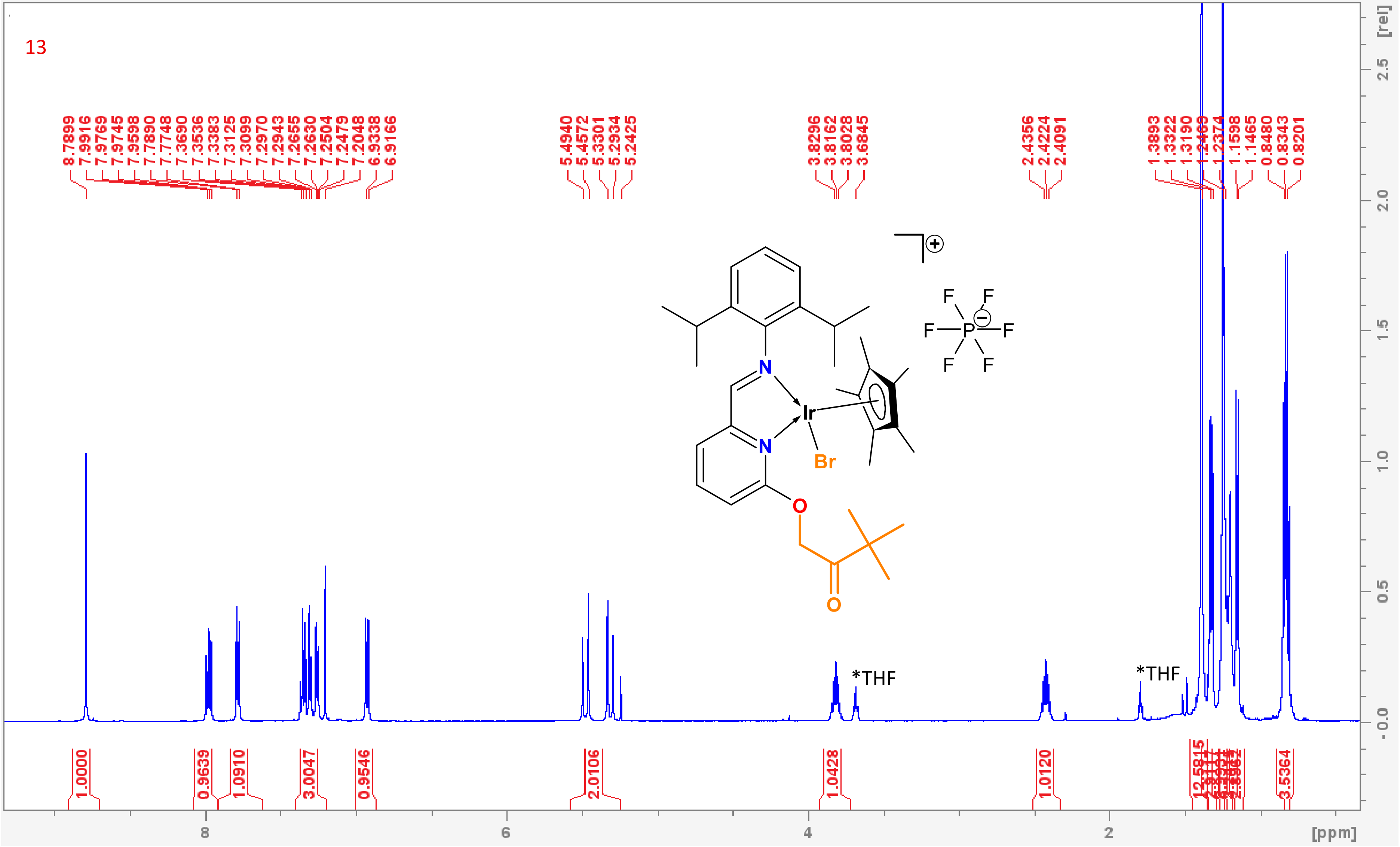

Figure S27. ^{1}H NMR ($CDCl_3$, 500.13 MHz, 300 K) of **13**.

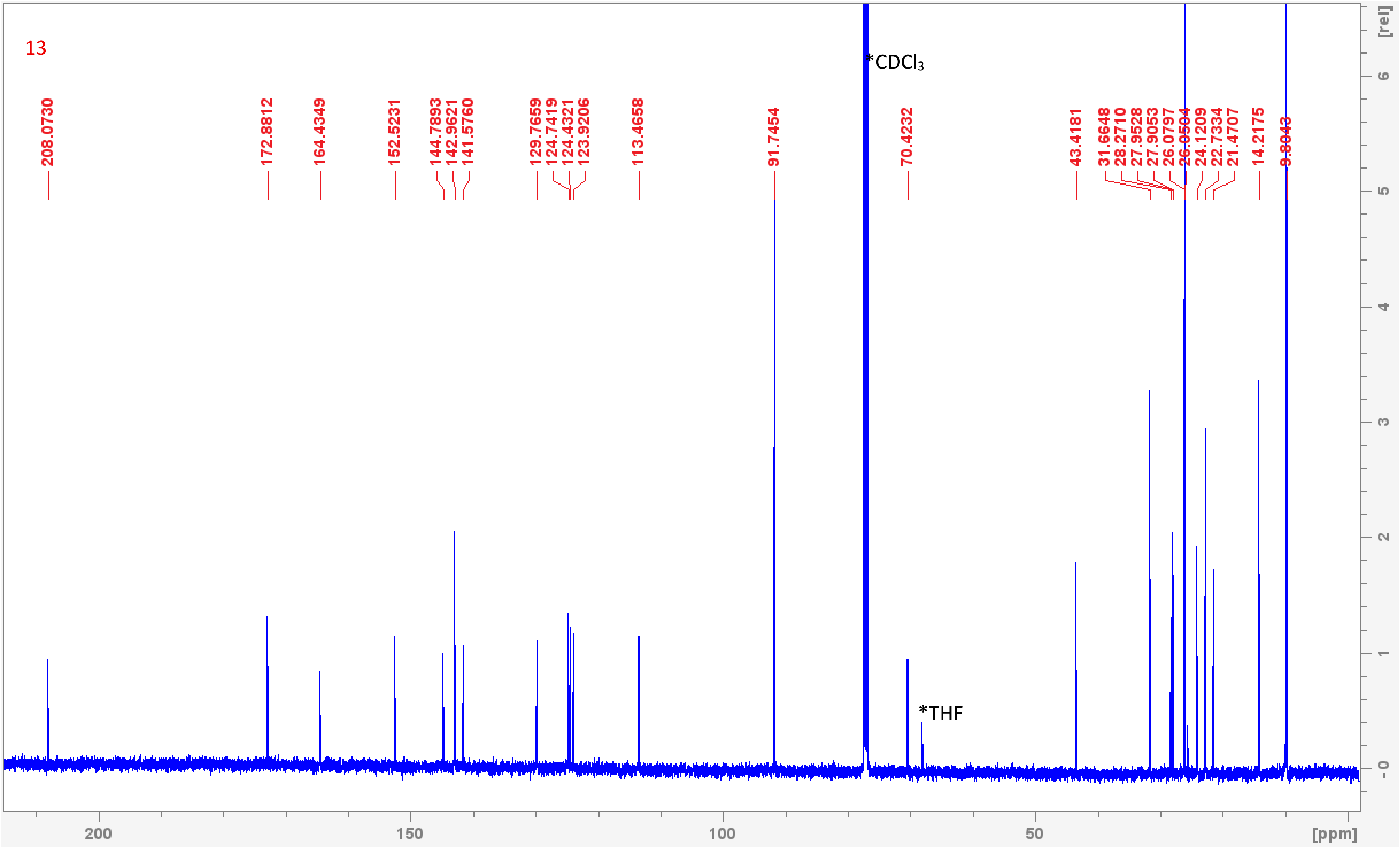

Figure S28. $^{13}C\{^1H\}$ NMR ($CDCl_3$, 125.78 MHz, 300 K) of **13**.

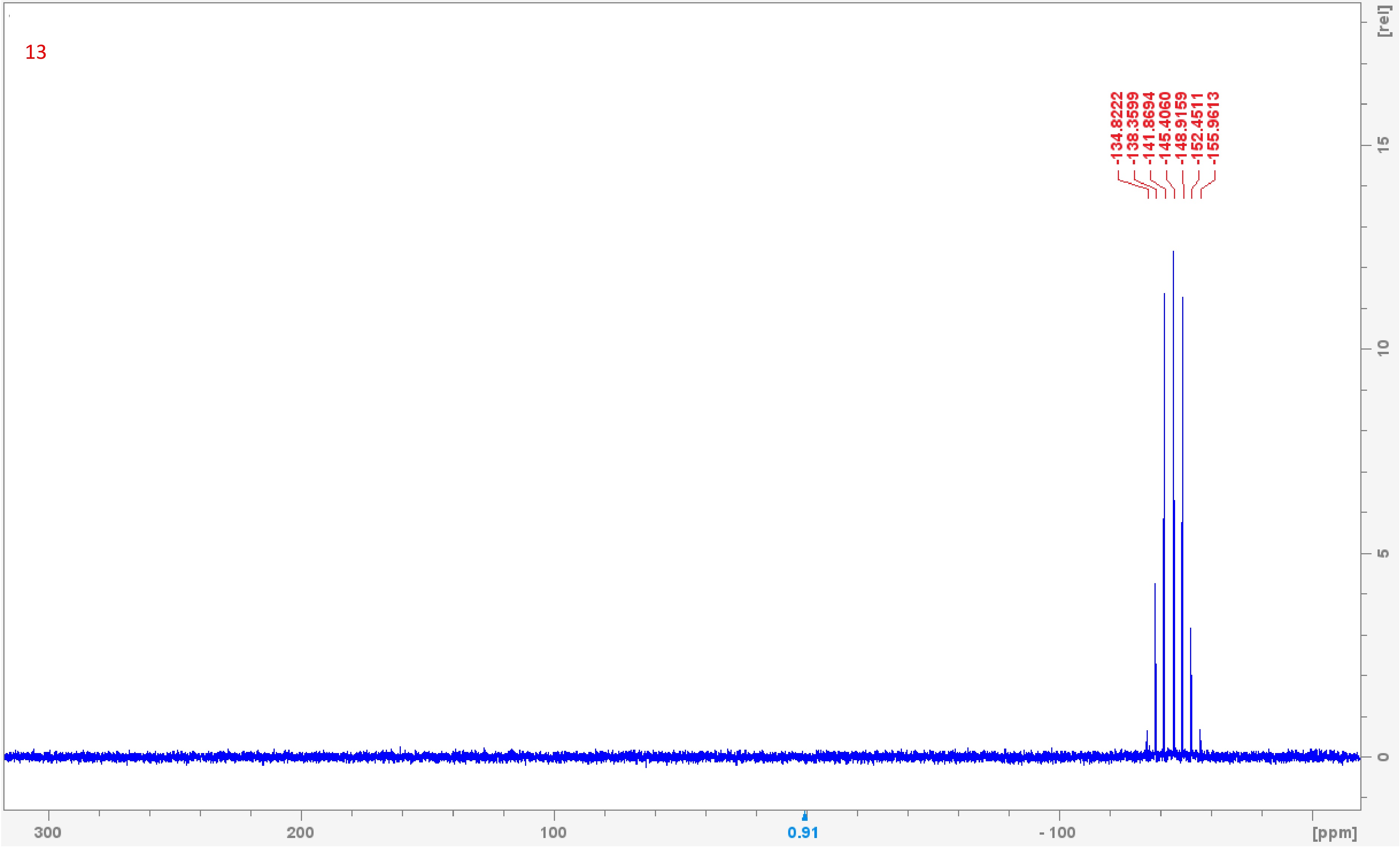

Figure S29. ${}^{31}P\{{}^{1}H\}$ NMR ($CDCl_3$, 202.46 MHz, 300 K) of **13**.

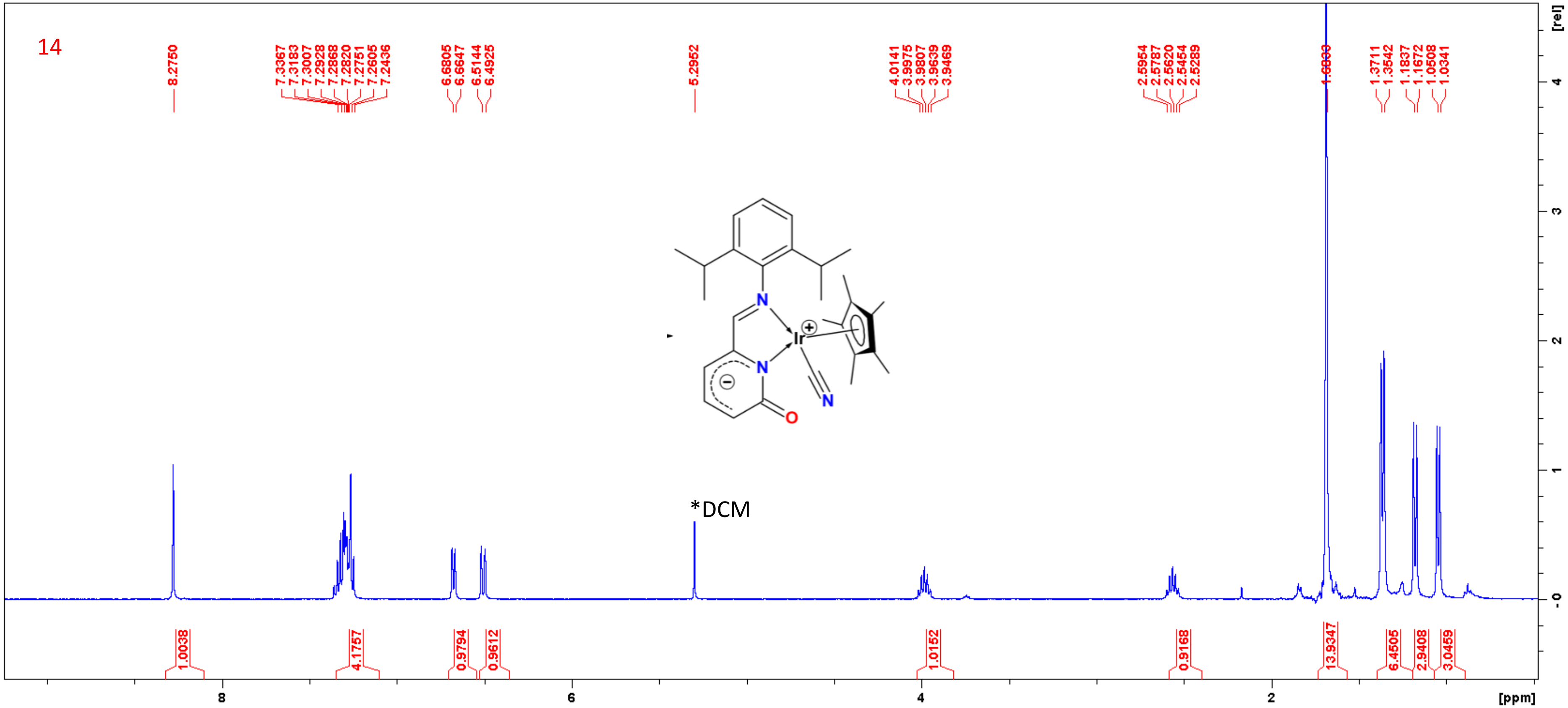

Figure S30. ¹H NMR ($CDCl_3$, 400.13 MHz, 300 K) of **14**.

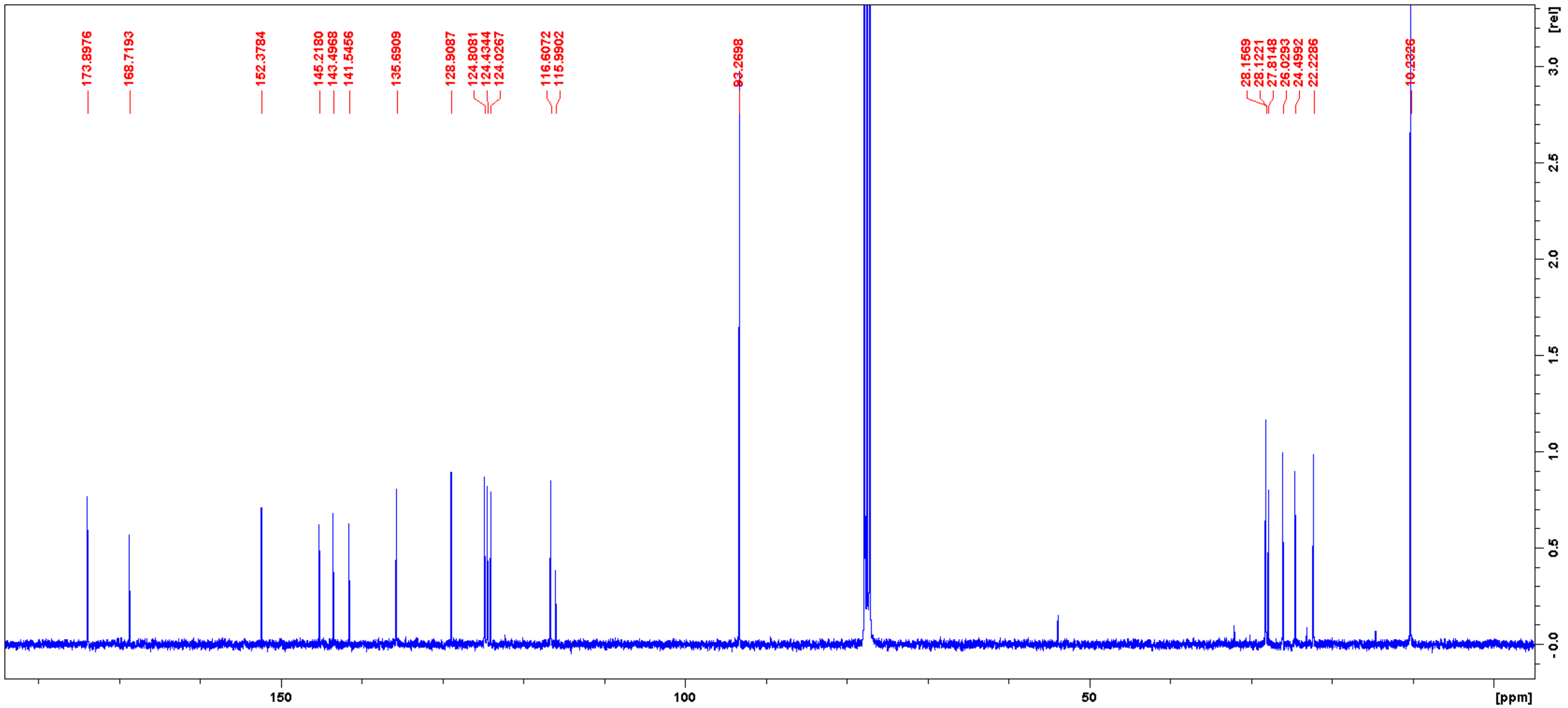

Figure S31. $^{13}C\{^{1}H\}$ NMR ($CDCl_3$, 100.61 MHz, 300 K) of **14**.

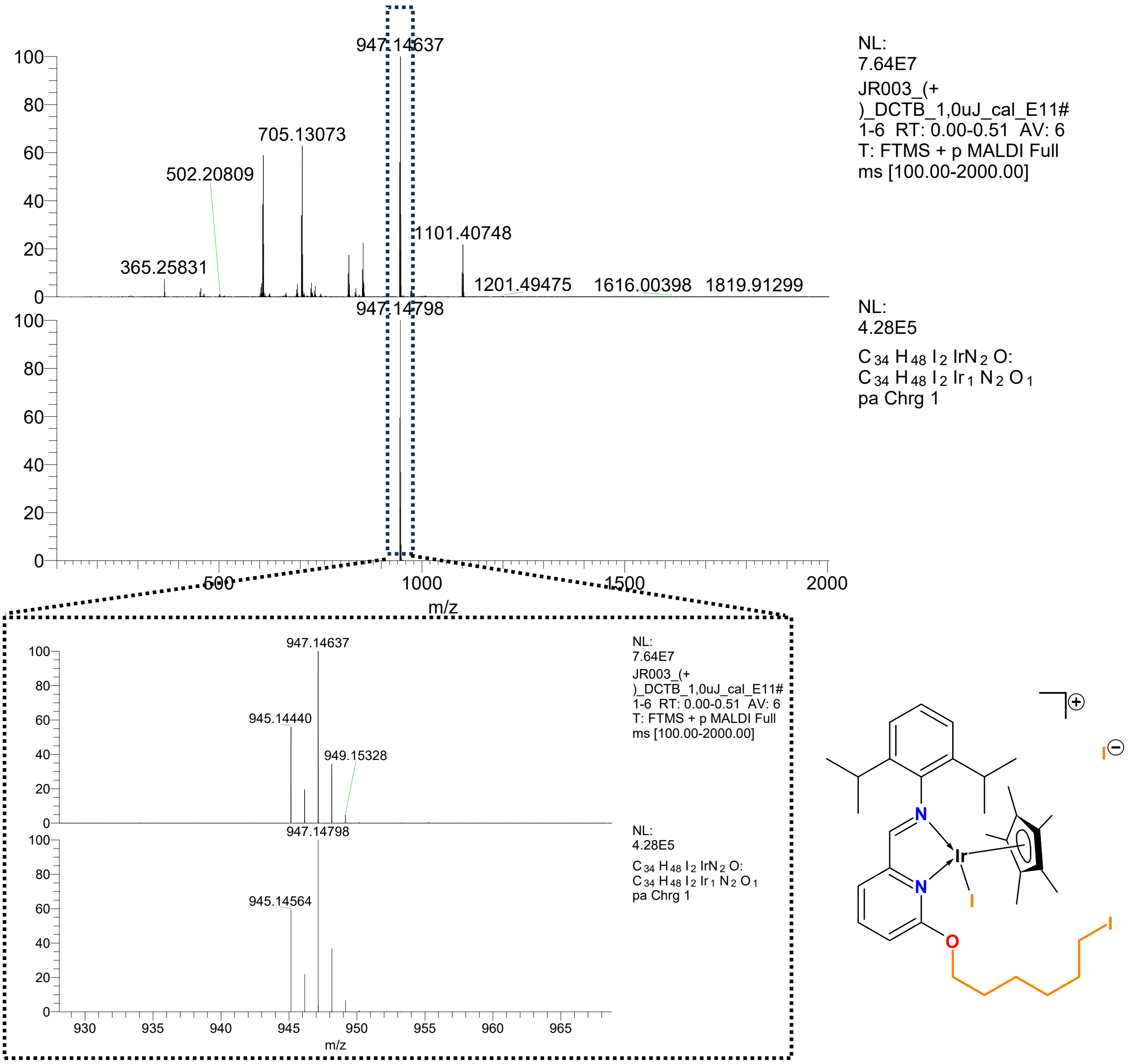

Figure S32. MS spectrum of **7**, $[M]^{+}$, top: experimental, bottom: simulated.

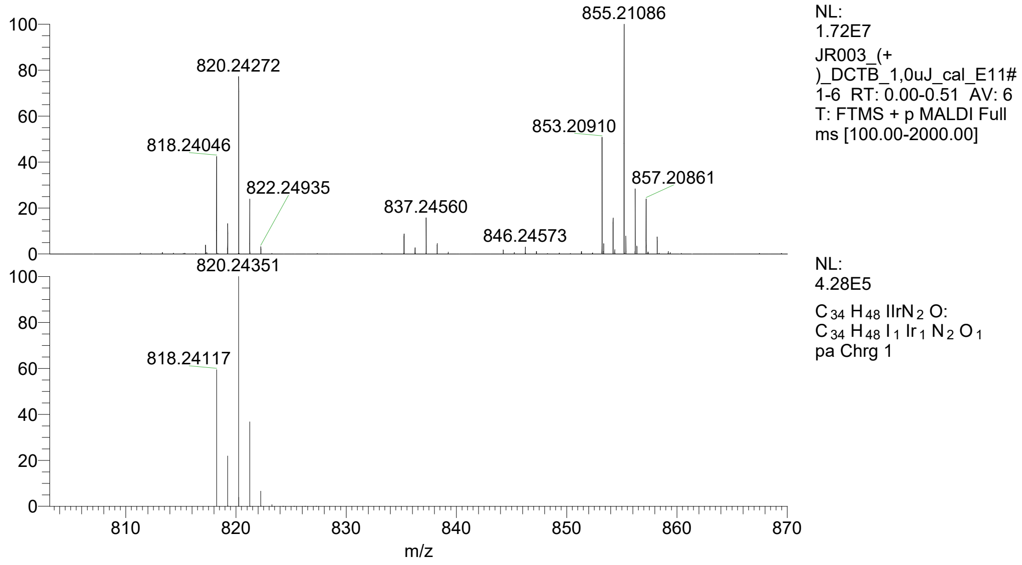

Figure S33. MS spectrum of **7**, $[M-I]^{+}$, top: experimental, bottom: simulated.

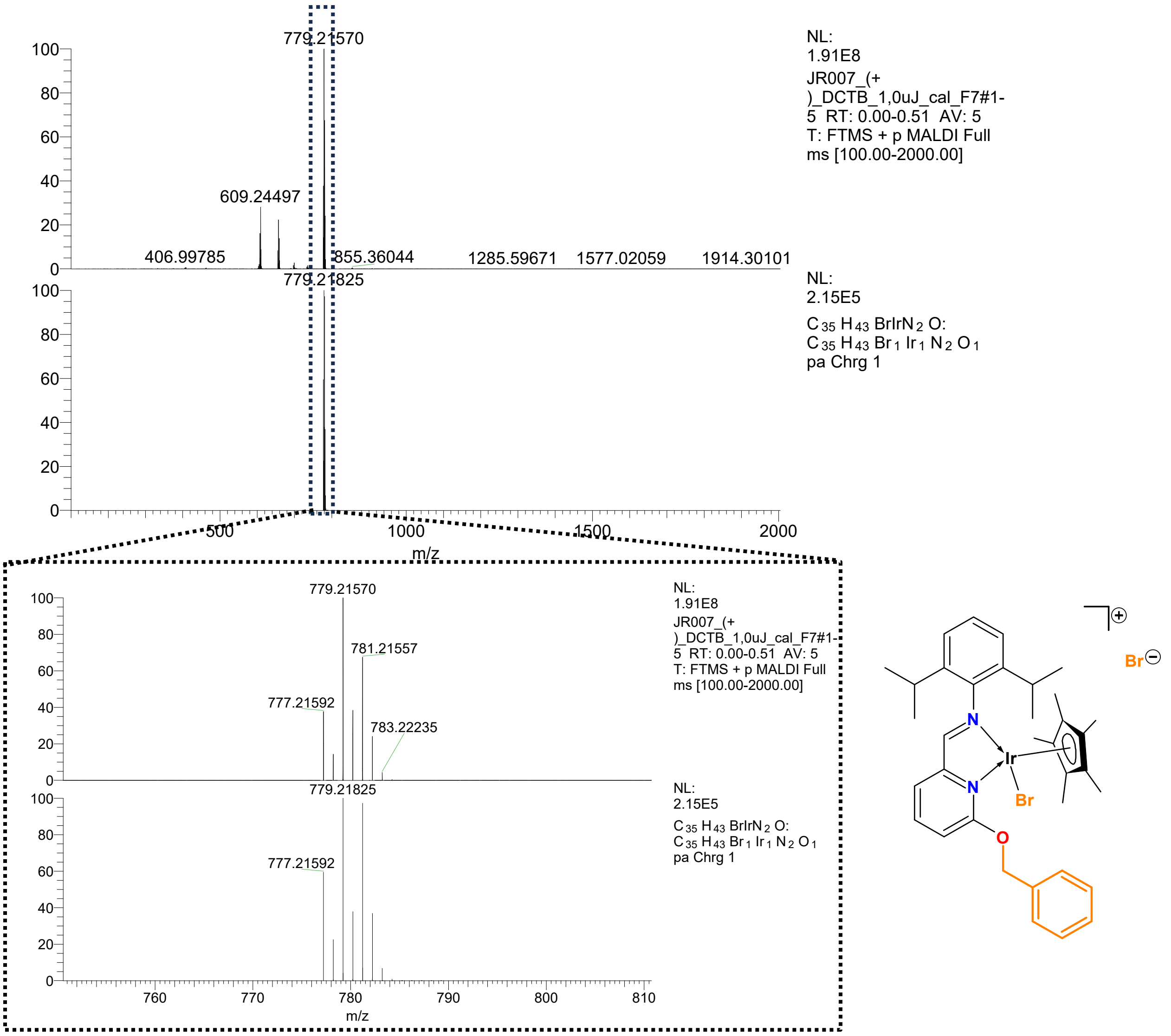

Figure S34. MS spectrum of **8**, [M]$^+$, top: experimental, bottom: simulated.

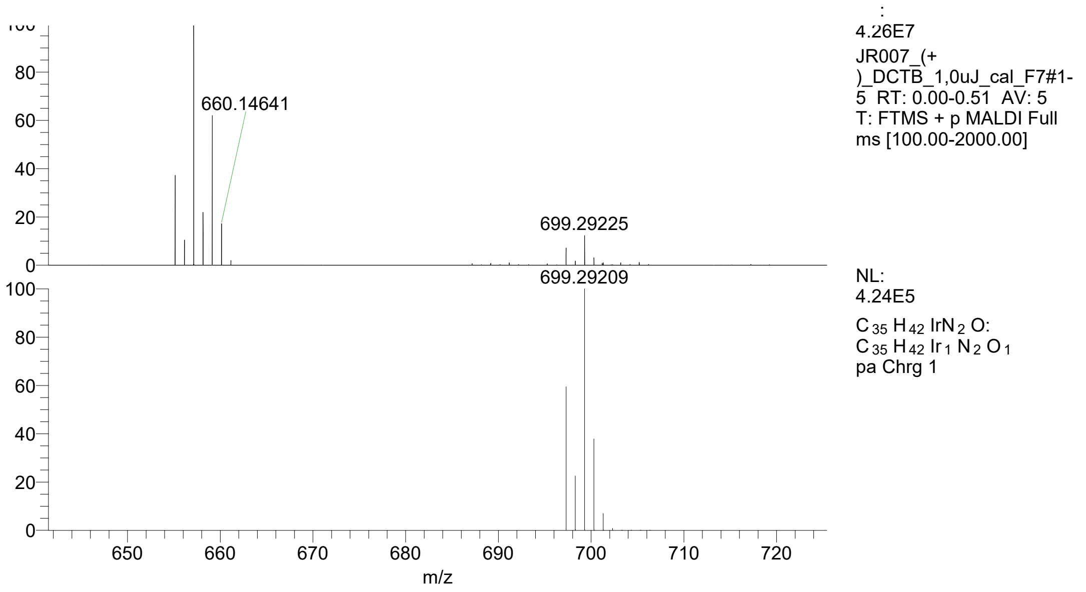

Figure S35. MS spectrum of **8**, [M-HBr]$^+$, top: experimental, bottom: simulated.

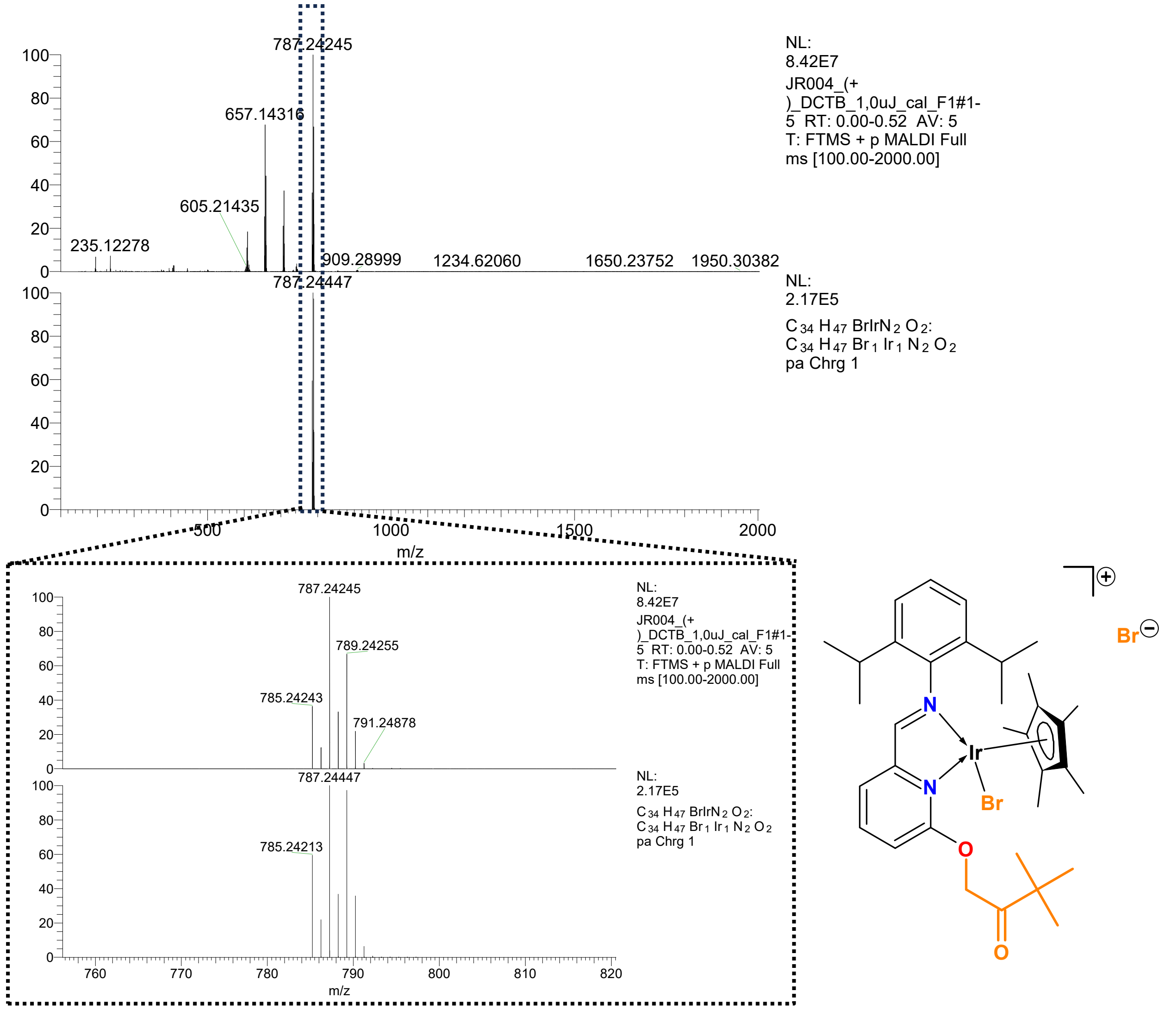

Figure S36. MS spectrum of **9**, $[M]^+$, top: experimental, bottom: simulated.

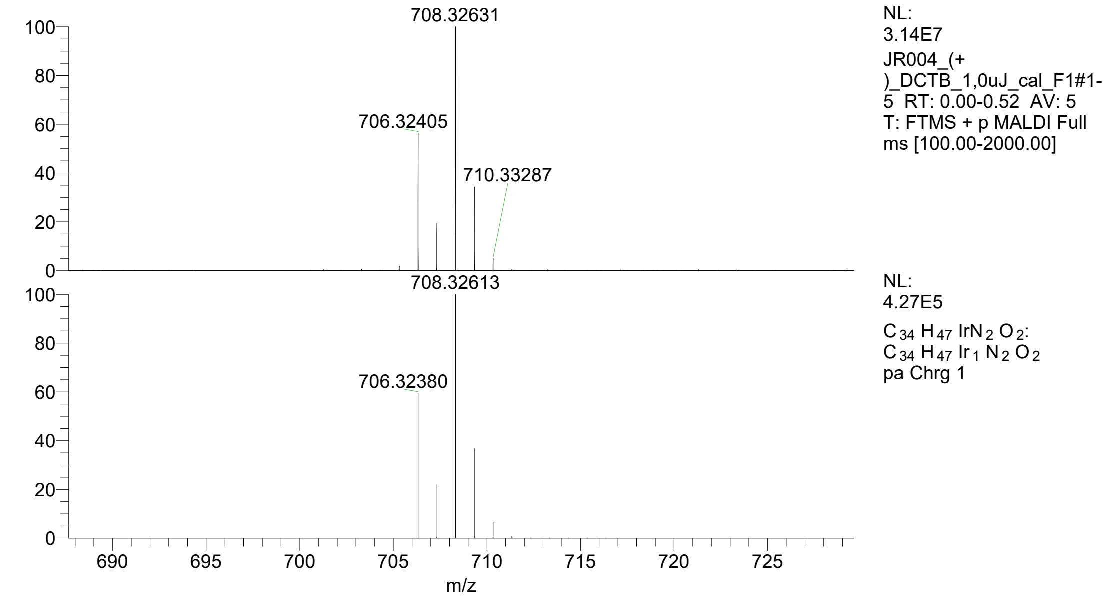

Figure S37. MS spectrum of **9,** $[M\text{-}Br]^+$, top: experimental, bottom: simulated.

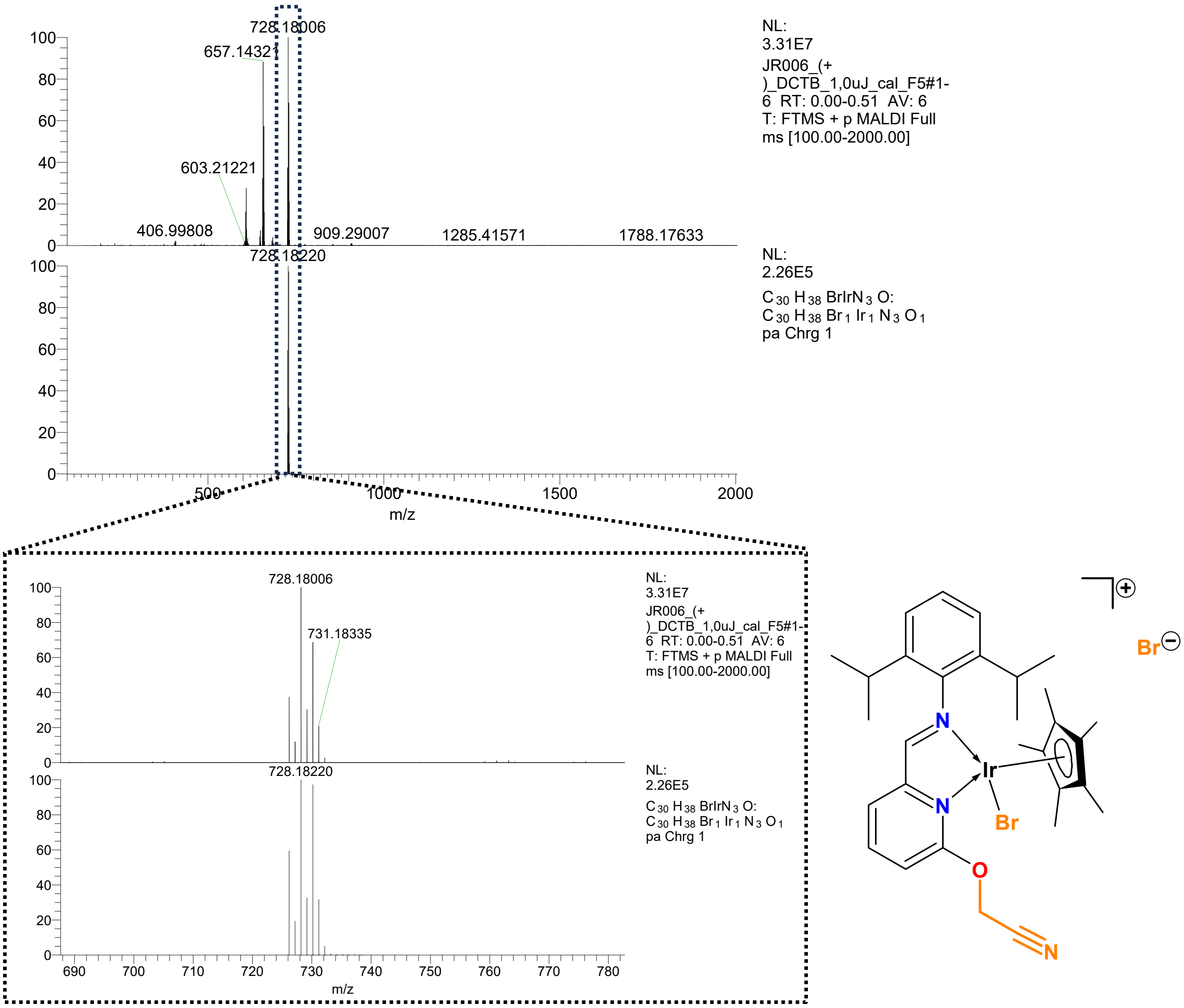

Figure S38. MS spectrum of **10**, $[M]^{+}$, top: experimental, bottom: simulated.

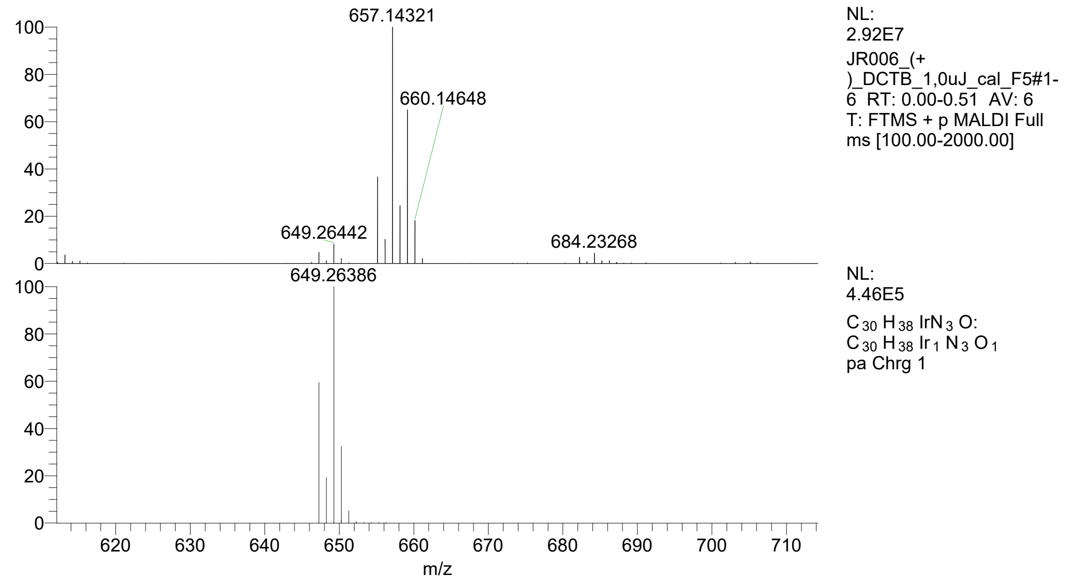

Figure S39. MS spectrum of **10**, $[M\text{-}Br]^{+}$, top: experimental, bottom: simulated.

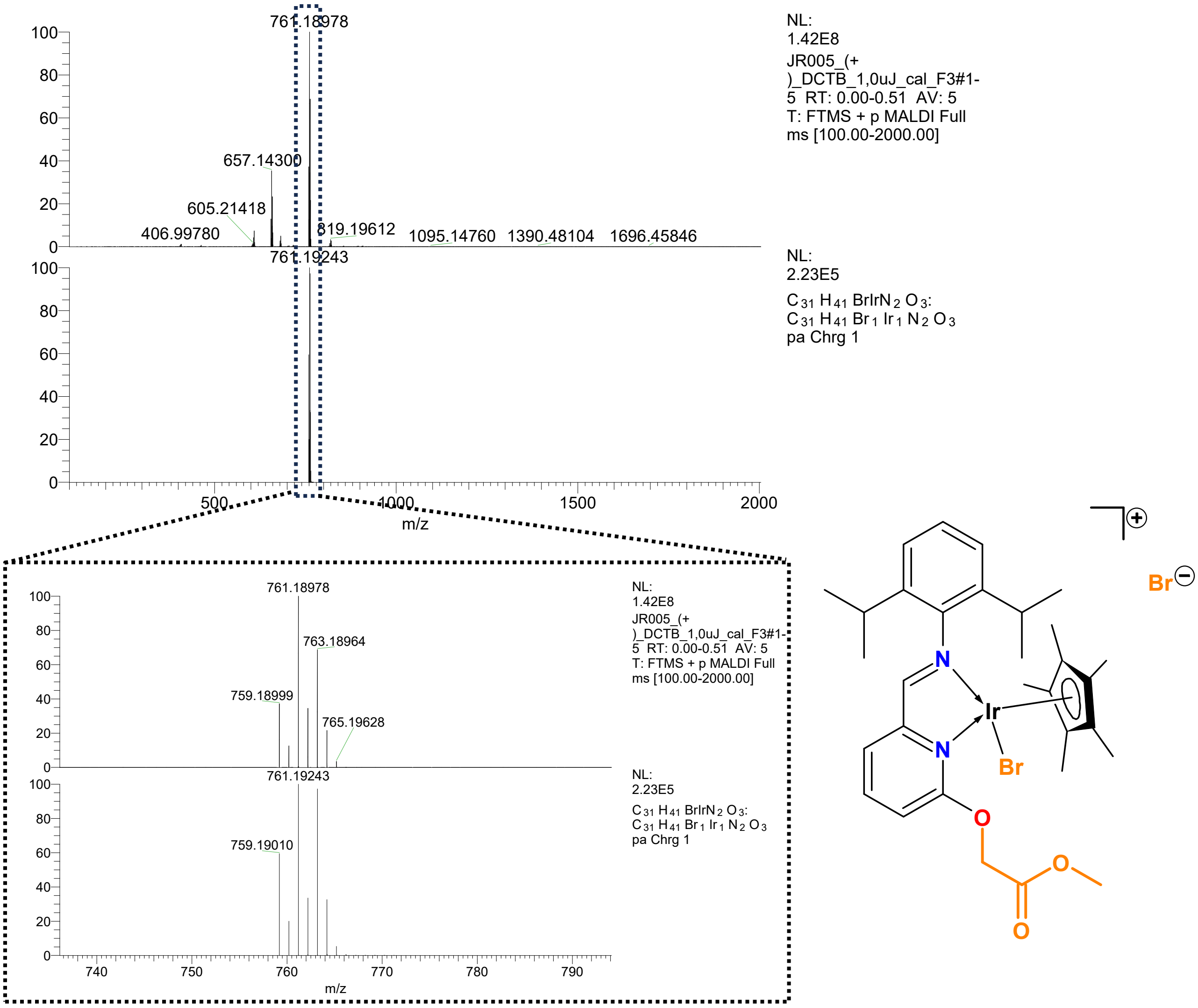

Figure S40. MS spectrum of **11**, $[M]^+$, top: experimental, bottom: simulated.

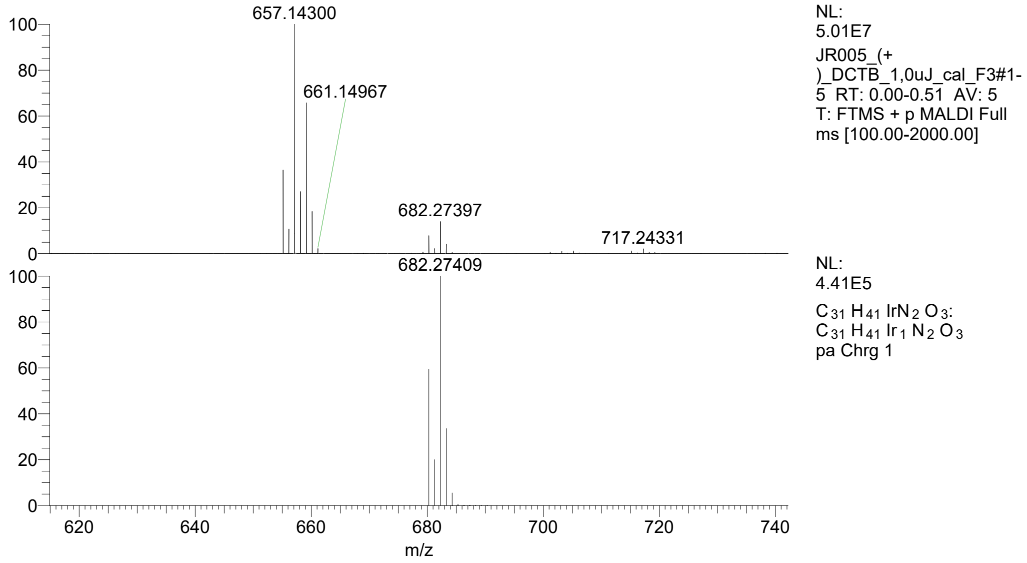

Figure S41. MS spectrum of **11**, $[M\text{-}Br]^+$, top: experimental, bottom: simulated.

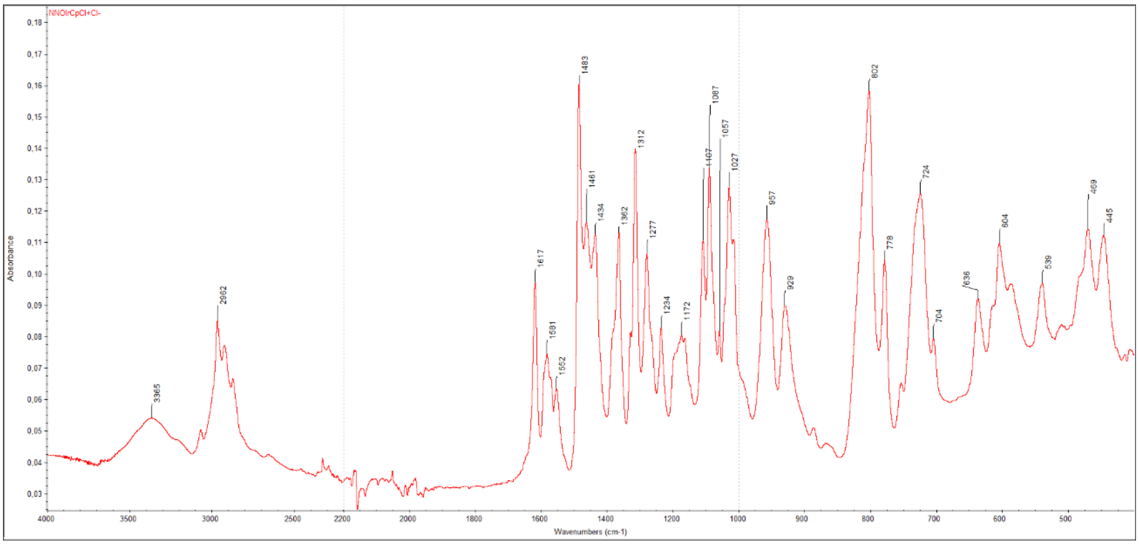

Figure S42. FT-IR (ATR) of **1**. Powder.

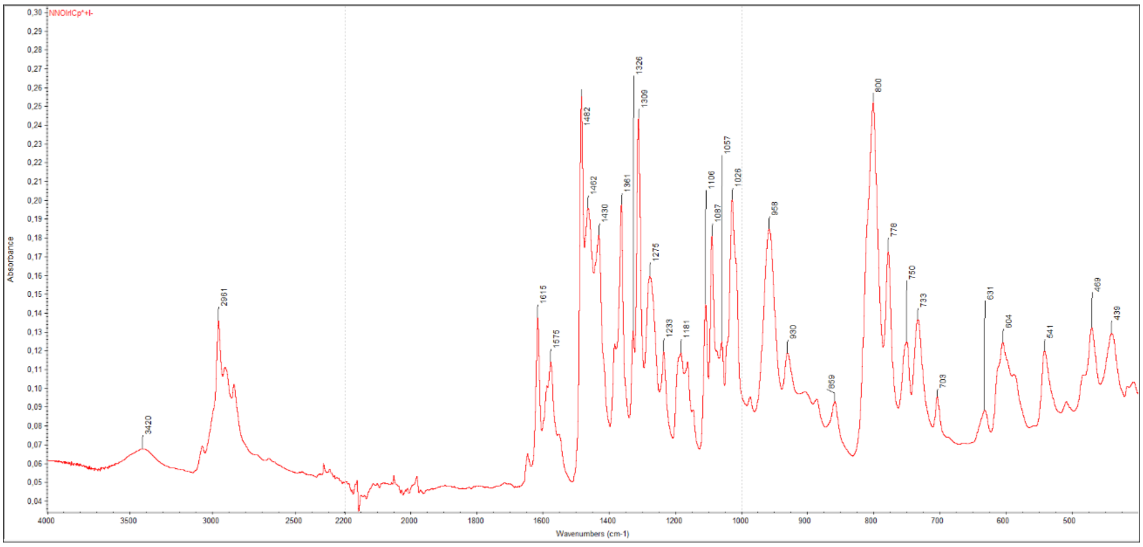

Figure S43. FT-IR (ATR) of **2**. Powder.

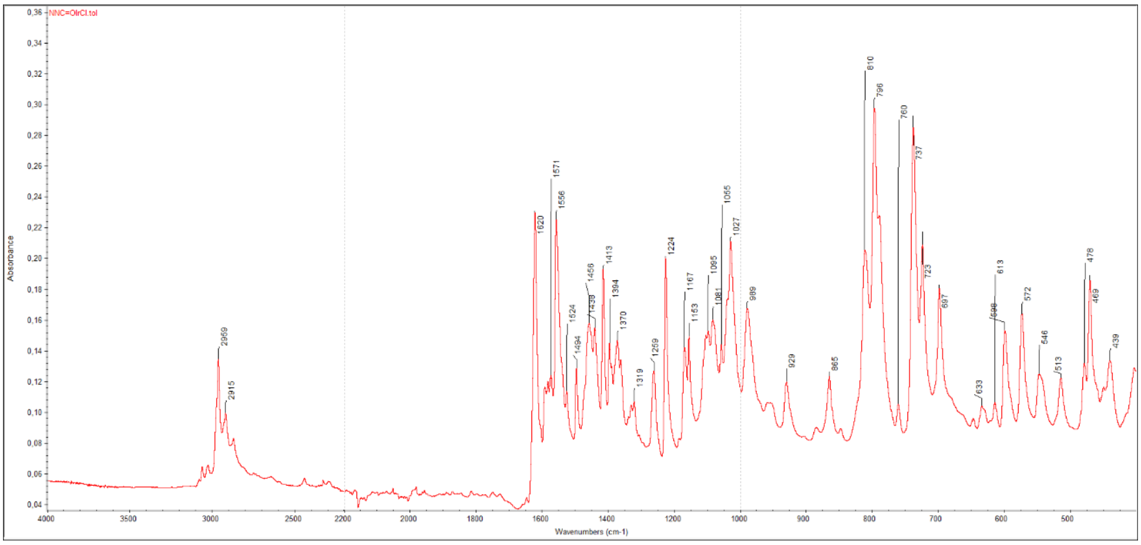

Figure S44. FT-IR (ATR) of **3**. Powder.

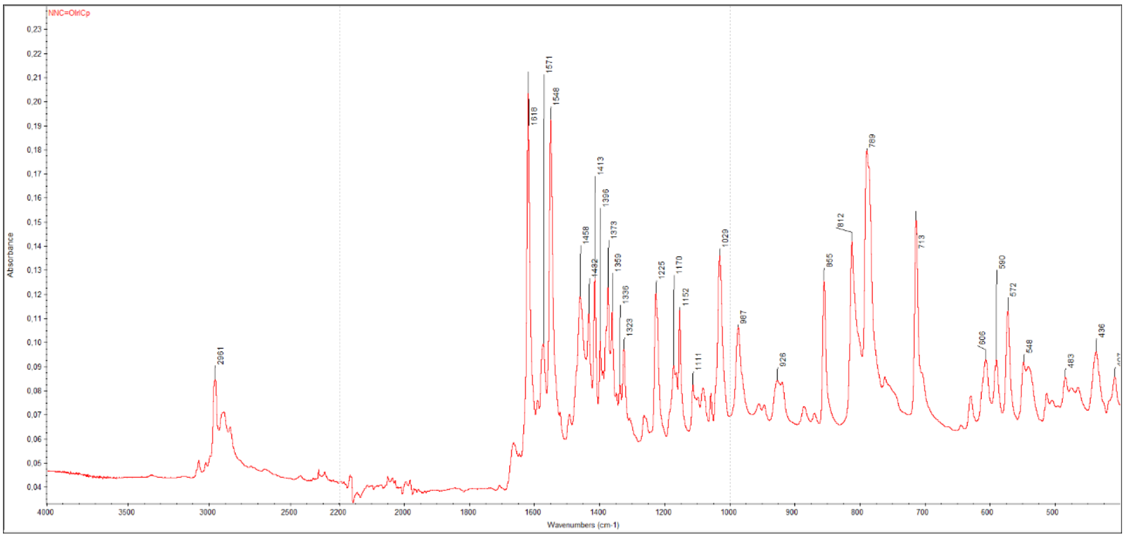

Figure S45. FT-IR (ATR) of **4**. Powder.

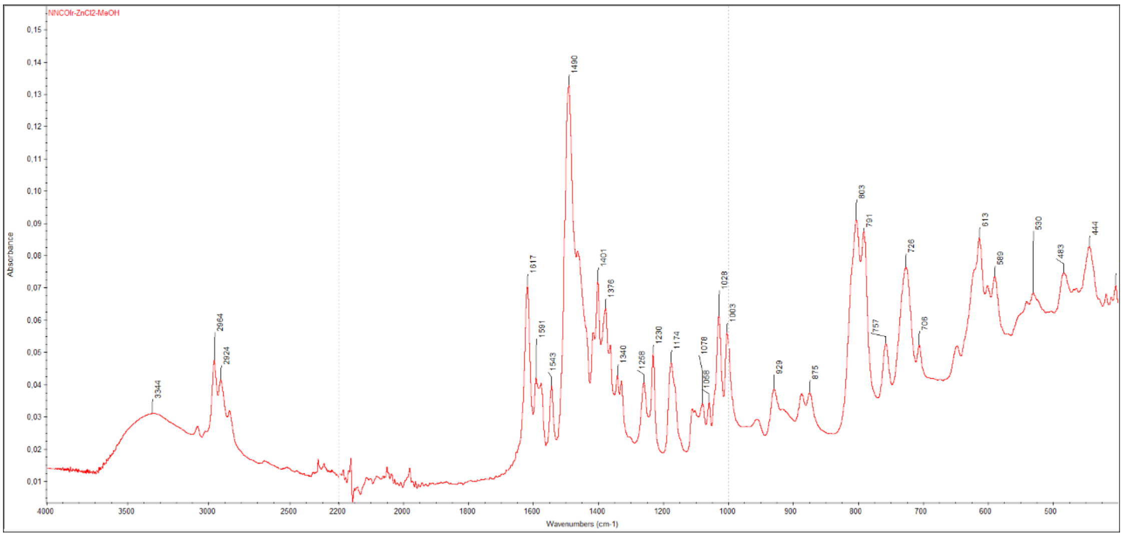

Figure S46. FT-IR (ATR) of **5**. Powder.

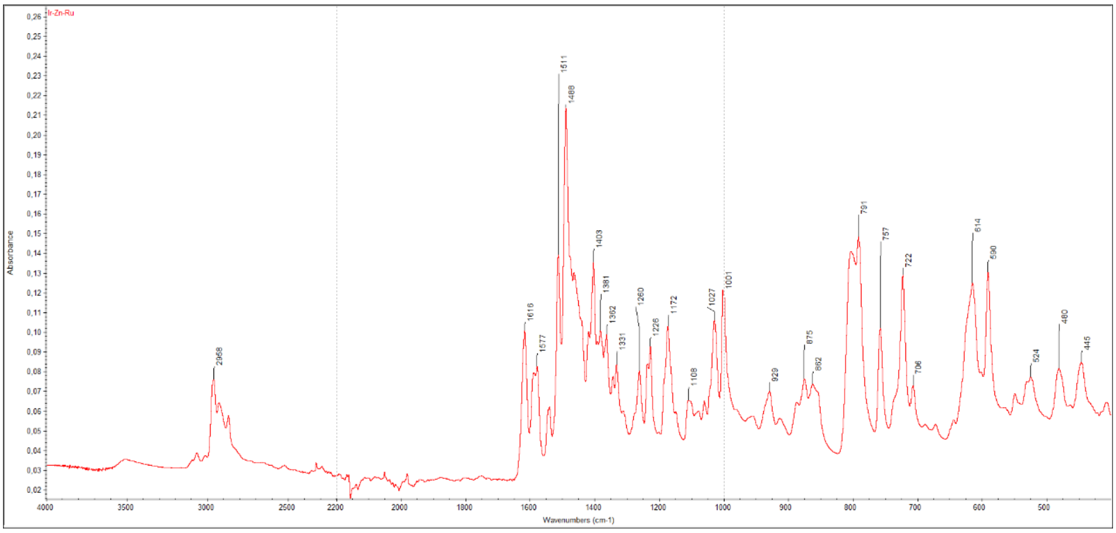

Figure S47. FT-IR (ATR) of **6**. Powder.

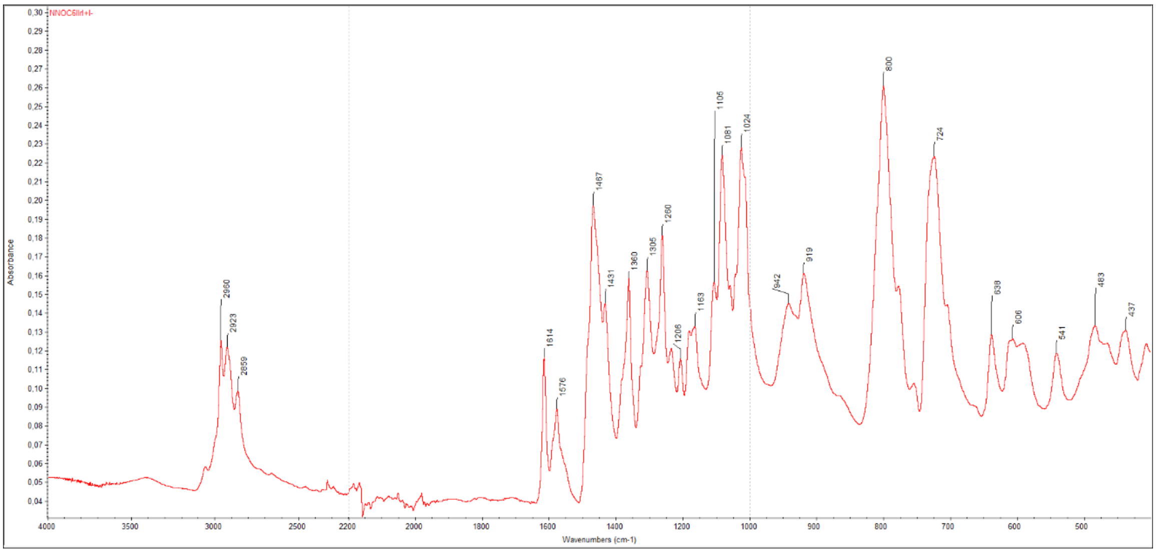

Figure S48. FT-IR (ATR) of **7**. Powder.

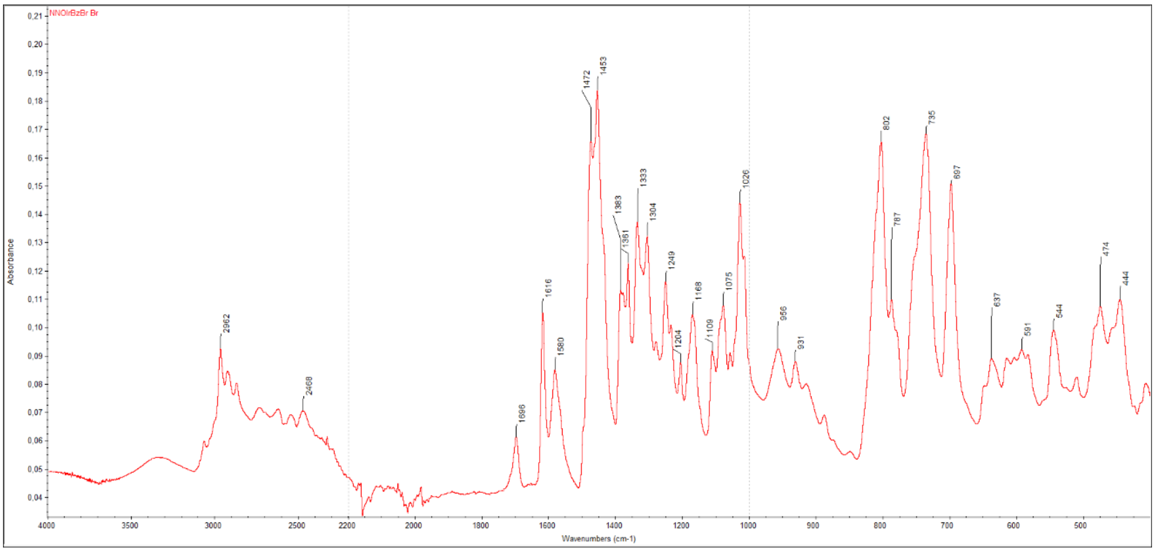

Figure S49. FT-IR (ATR) of **8**. Powder.

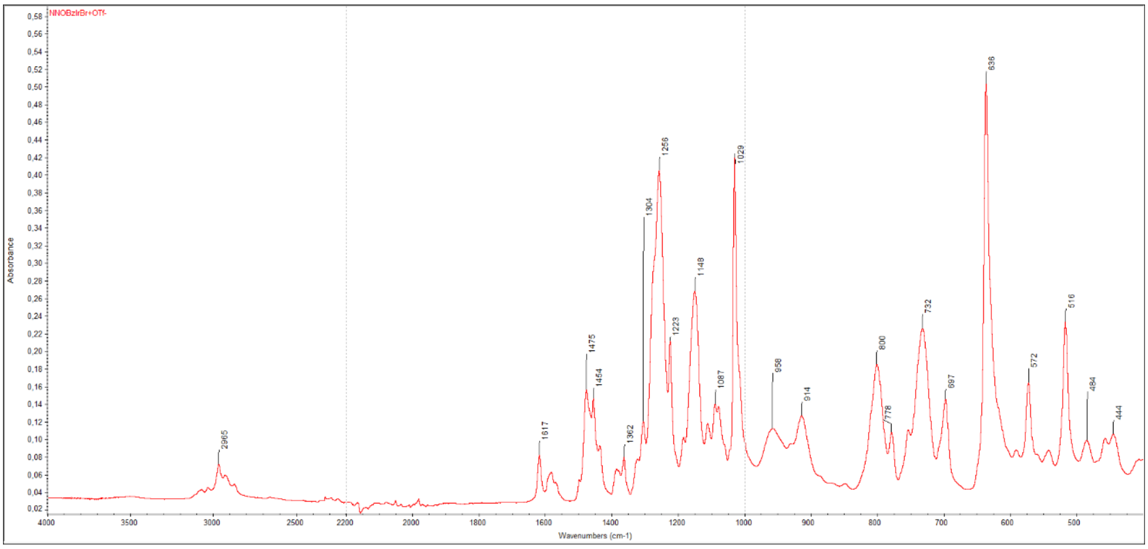

Figure S50. FT-IR (ATR) of **12**. Powder.

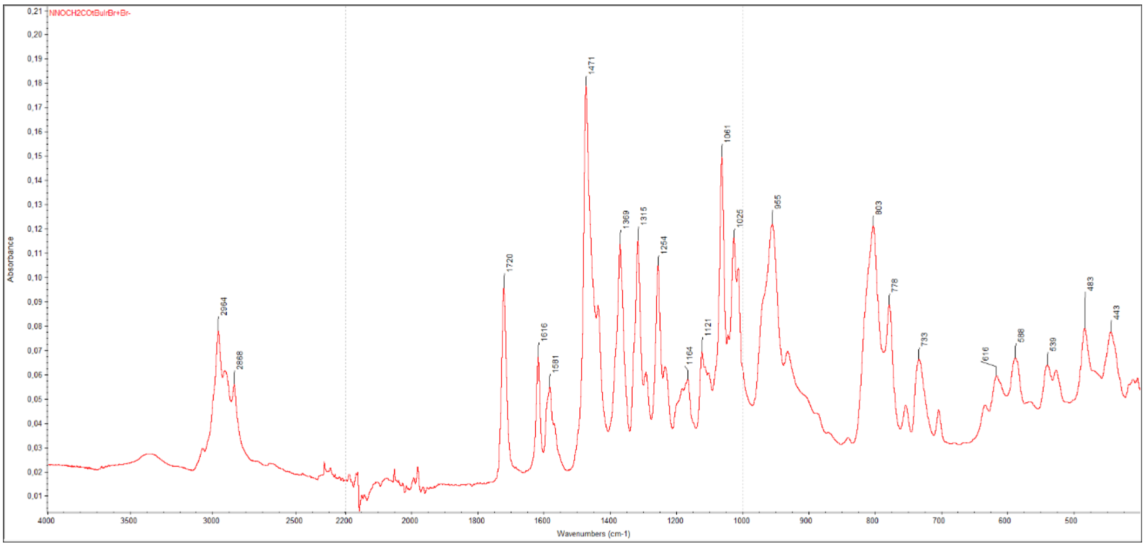

Figure S51. FT-IR (ATR) of **9**. Powder.

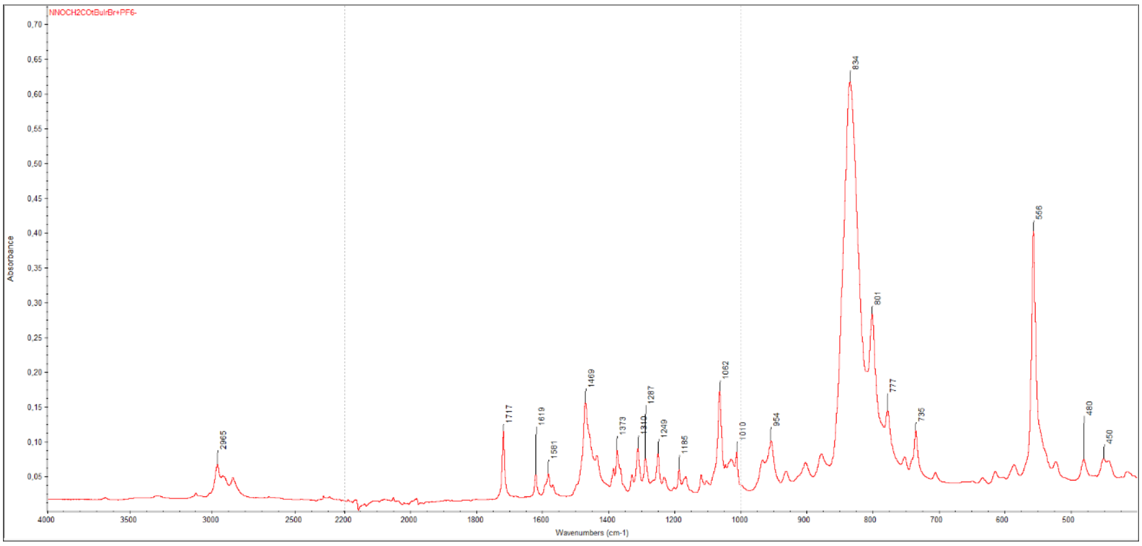

Figure S52. FT-IR (ATR) of **13**. Powder.

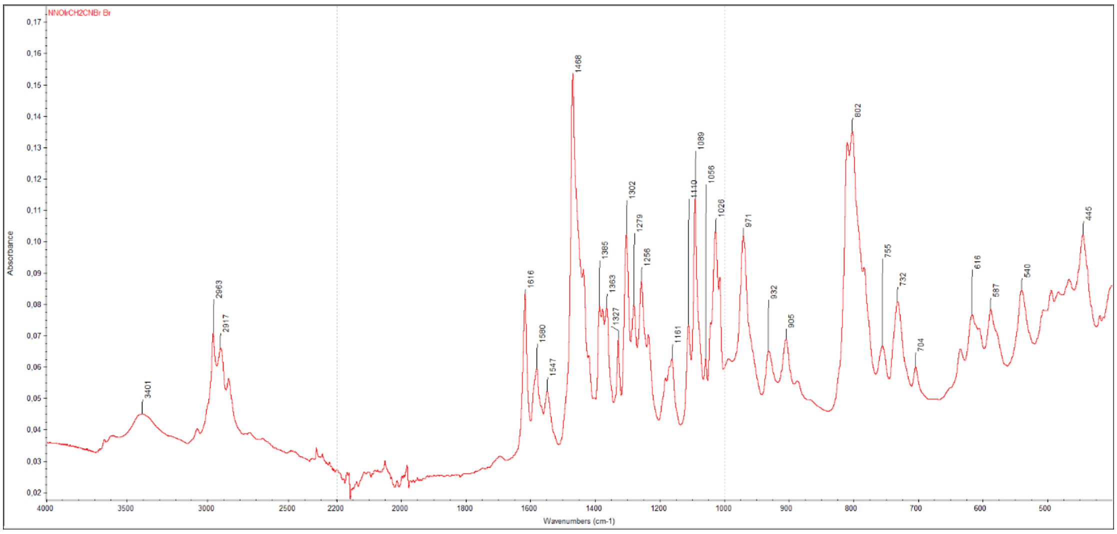

Figure S53. FT-IR (ATR) of **10**. Powder.

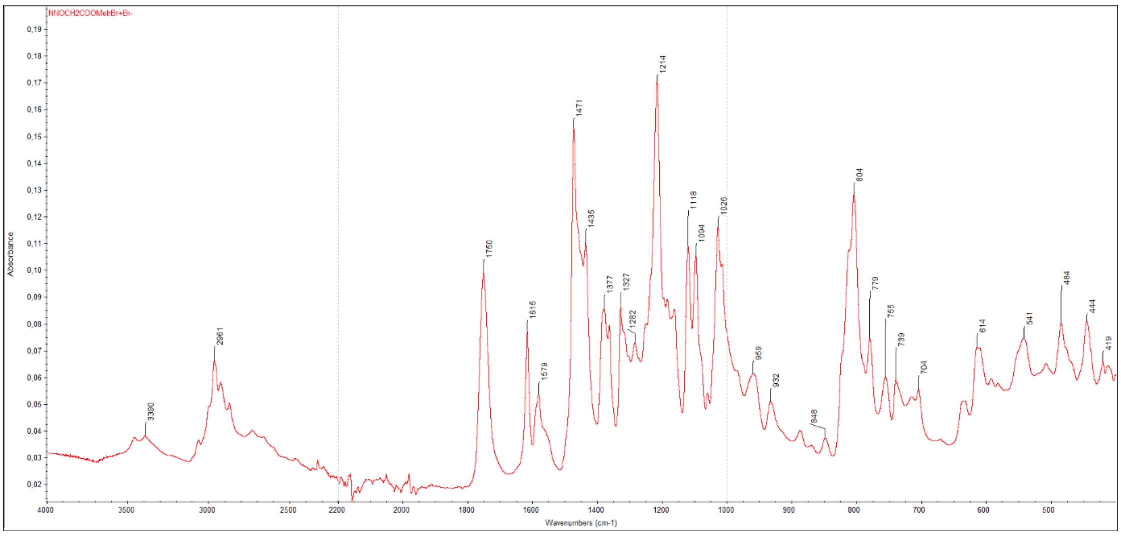

Figure S54. FT-IR (ATR) of **11**. Powder.

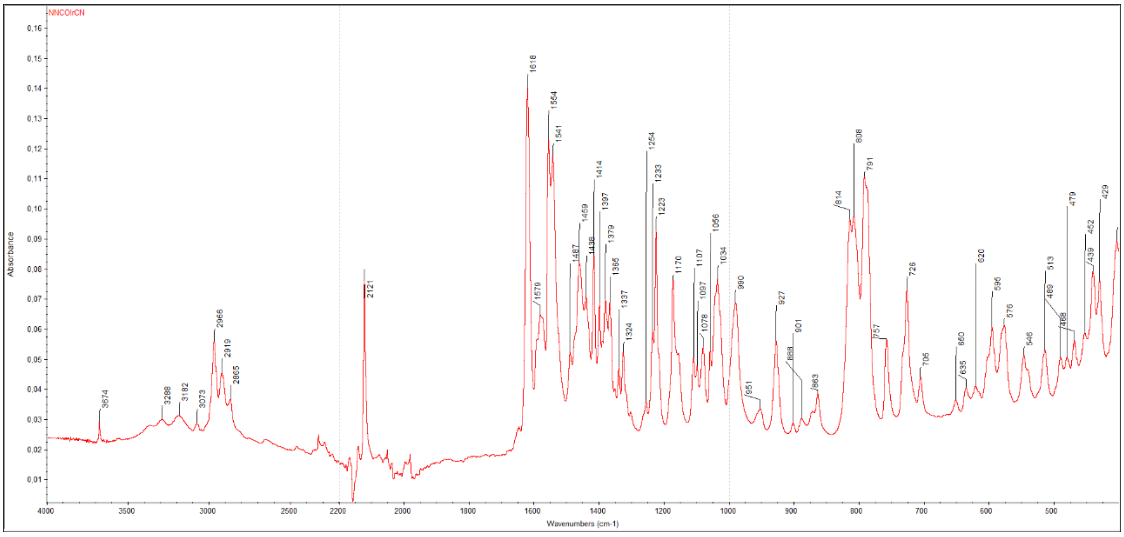

Figure S55. FT-IR (ATR) of **14**. Powder.

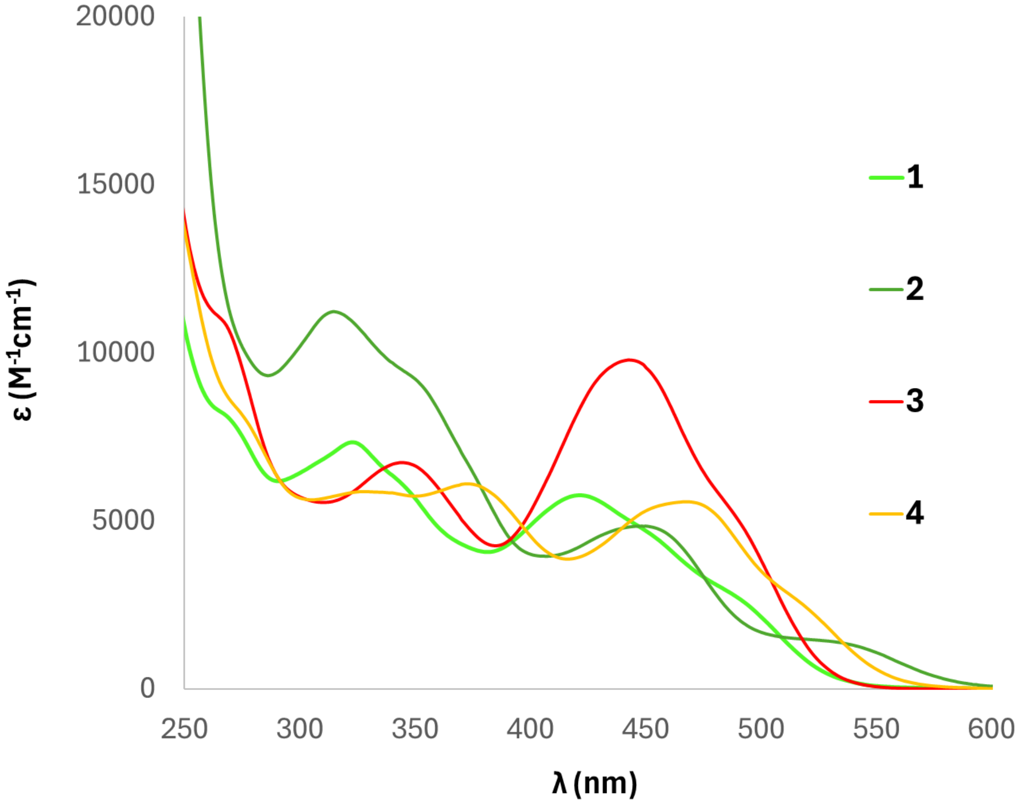

Figure S56. Absorption spectra of ionic **1—2** and neutral **3—4** Ir(III) complexes.

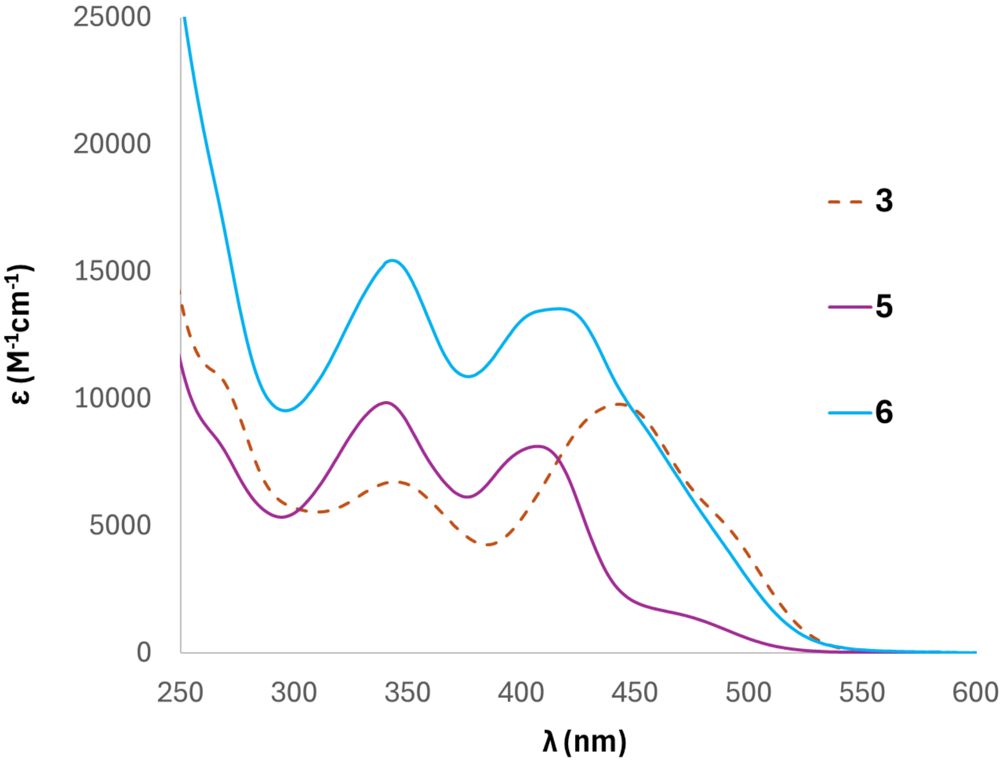

Figure S57. Absorption spectra of bimetallic complexes **5** and **6** compared with the starting Ir(III) complex **3**.

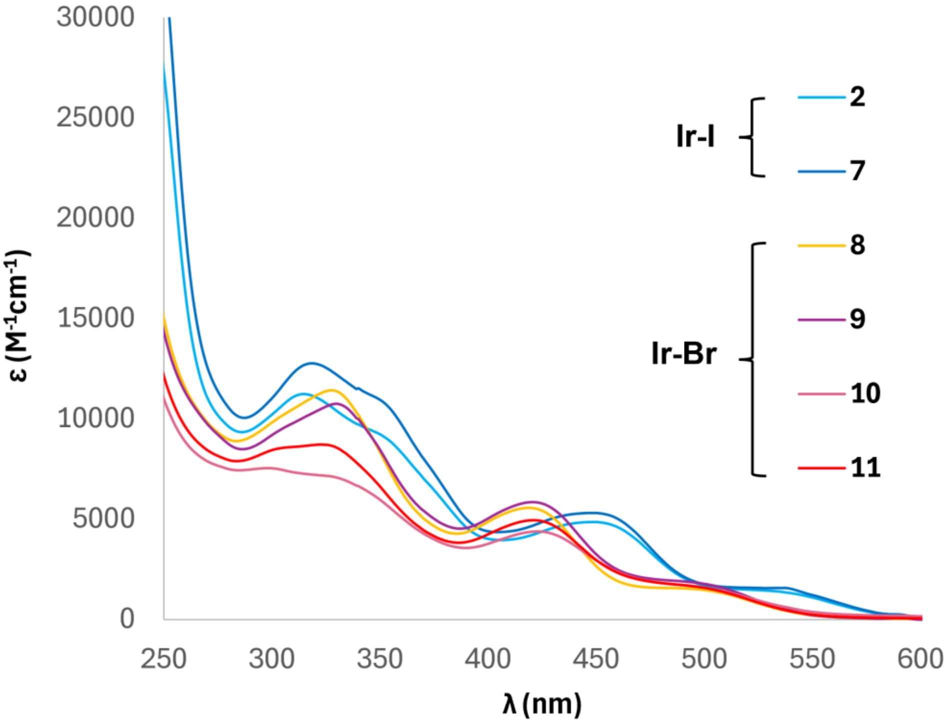

Figure S58. Absorption spectra of ionic complexes **2**, **7**–**11**.

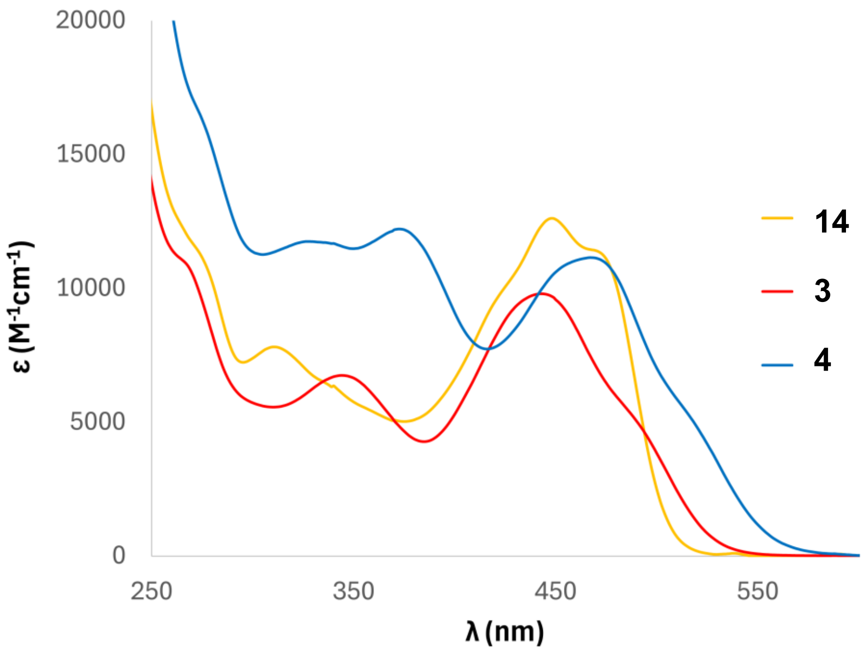

Figure S59. Absorption spectra of neutral complex **14** compared with analogues **3** and **4**.

Characterization of the Complexes: Crystallographic data

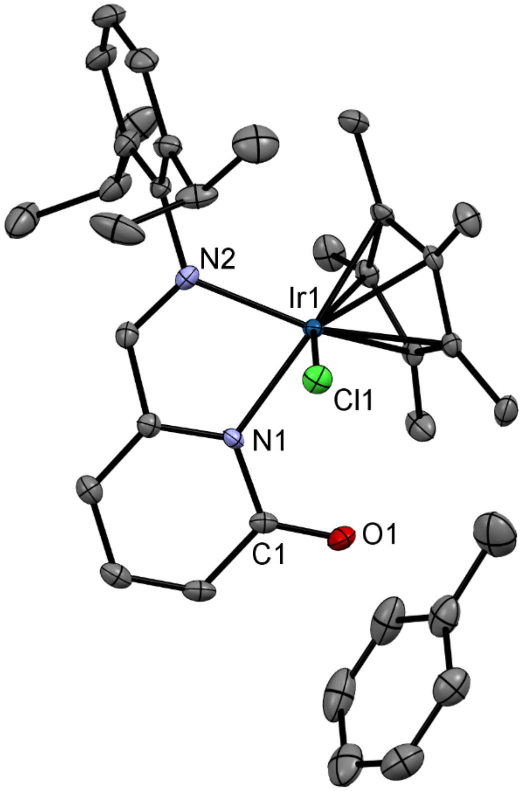

Figure S60. ORTEP of **3·C_7H_8**.

Non-H atoms are represented by 50% probability ellipsoids. H atoms are omitted for clarity. Bond lengths (Å): Ir1-N1 2.106(2); Ir1-N2 2.125(3); Ir1-Cl1 2.4324(8); C1-O1 1.243(4). Bond angles (°): N1-Ir1-N2 76.8(1); N1-Ir1-Cl1 80.53(7); N2-Ir1-Cl1 90.54(7).

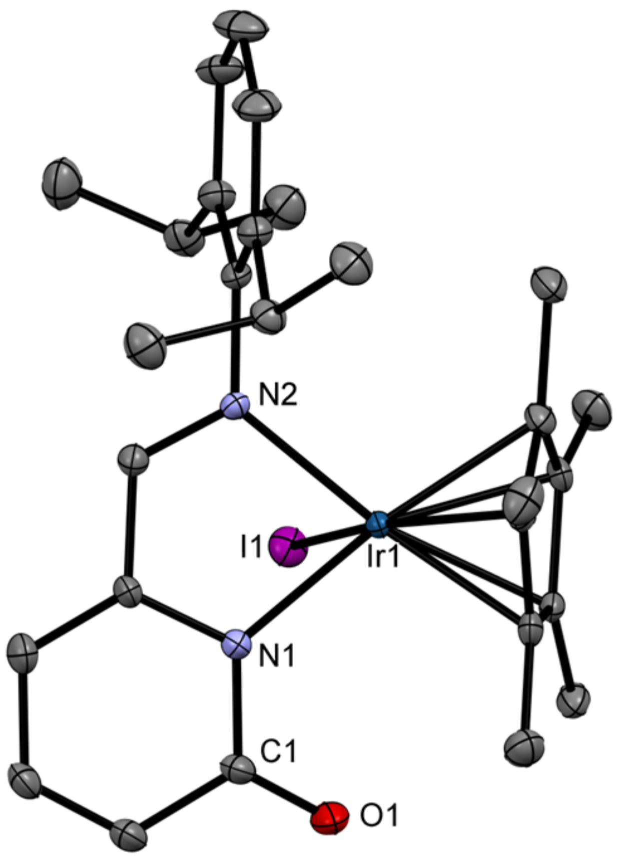

Figure S61. ORTEP of **4**.

Non-H atoms are represented by 50% probability ellipsoids. H atoms are omitted for clarity. Bond lengths (Å): Ir1-N1 2.086(4); Ir1-N2 2.122(4); Ir1-I1 2.7083(4); C1-O1 1.238(6). Bond angles (°): N1-Ir1-N2 77.1(1); N1-Ir1-I1 81.6(1); N2-Ir1-I1 91.5(1).

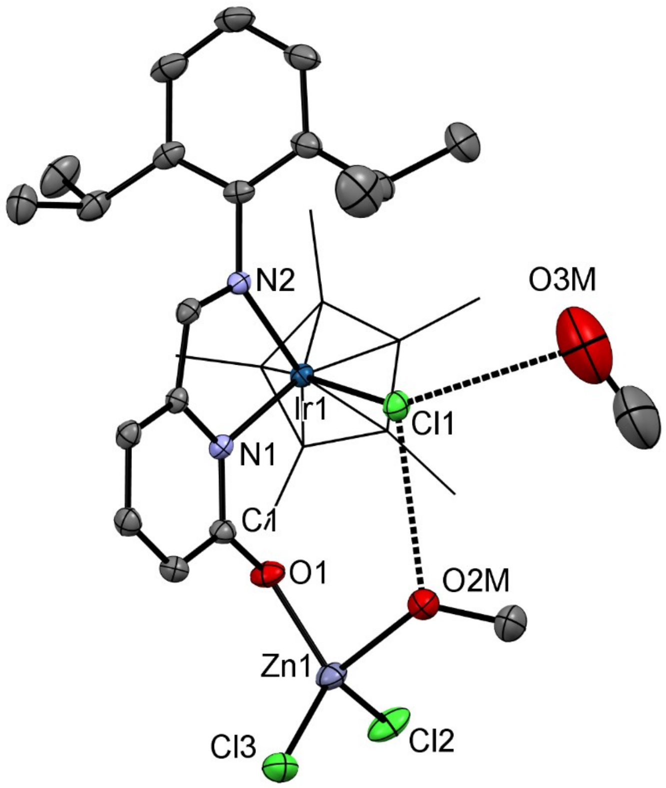

Figure S62. ORTEP of **5·MeOH**.

Non-H atoms are represented by 50% probability ellipsoids. H atoms are omitted for clarity. Bond lengths (Å): Ir1-N1 2.128(3); Ir1-N2 2.121(2); Cl1-Ir1 2.4436(9); C1-O1 1.270(4); Zn1-O1 1.956(2); Zn1-O2M 2.064(3); Zn1-Cl2 2.219(1); Zn1-Cl3 2.200(1); Cl1-H2M 2.23(4). Bond angles (°): N1-Ir1-N2 76.4(1); O1-Zn1-O2M 89.9(1); Cl2-Zn1-Cl3 118.19(4).

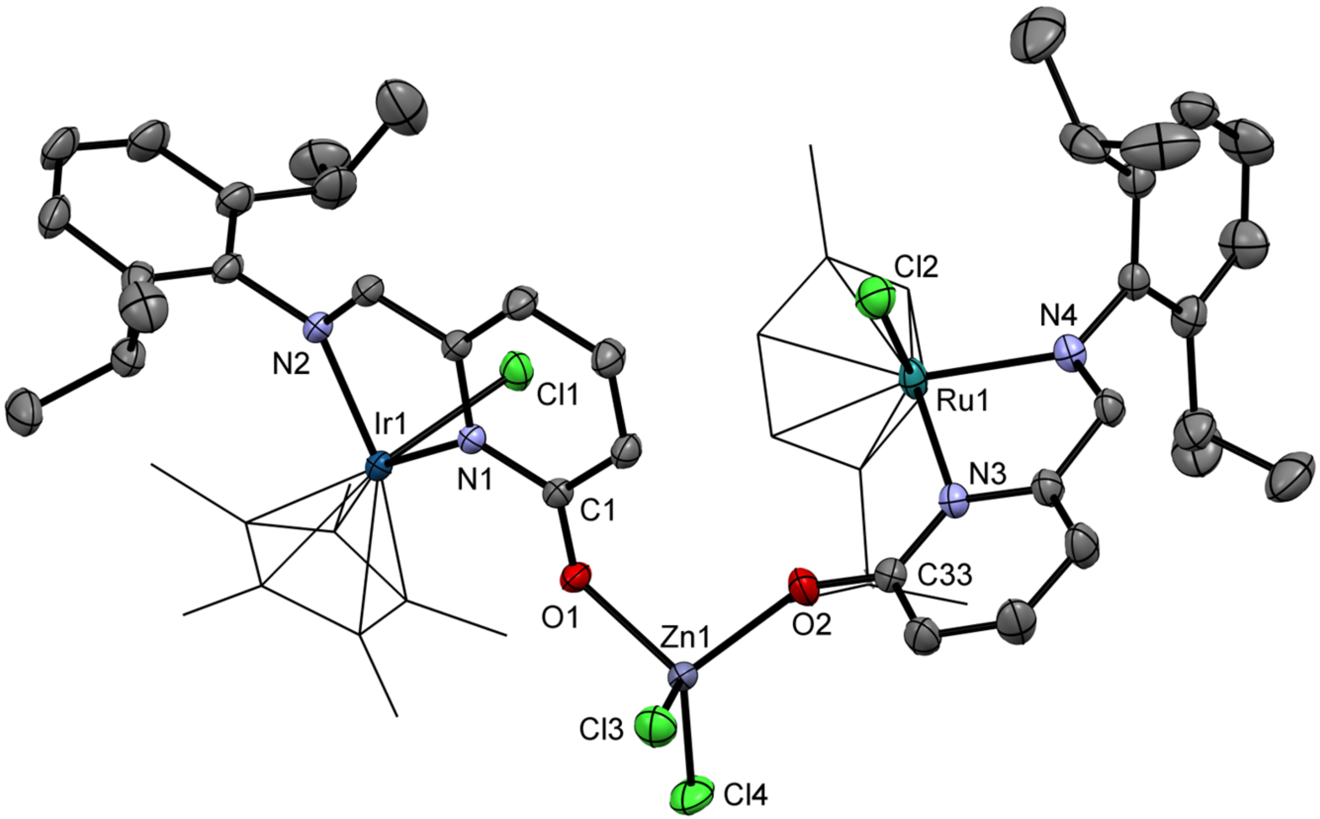

Figure S63. ORTEP of **6**.

Non-H atoms are represented by 50% probability ellipsoids. H atoms are omitted for clarity. Bond lengths (Å): Ir1-N1 2.118(2); Ir1-N2 2.132(2); Ru1-N3 2.117(2); Ru1-N4 2.101(2); Cl1-Ir1 2.4157(6); Cl2-Ru1 2.4039(8); C1-O1 1.273(3); C33-O2 1.273(3); Zn1-O1 1.982(2); Zn1-O2 1.977(2); Zn1-Cl3 2.2325(8); Zn1-Cl4 2.2309(9). Bond angles (°): N1-Ir1-N2 76.62(7); N3-Ru1-N4 76.43(8); O1-Zn1-O2 98.37(8); Cl3-Zn1-Cl4 118.83(3).

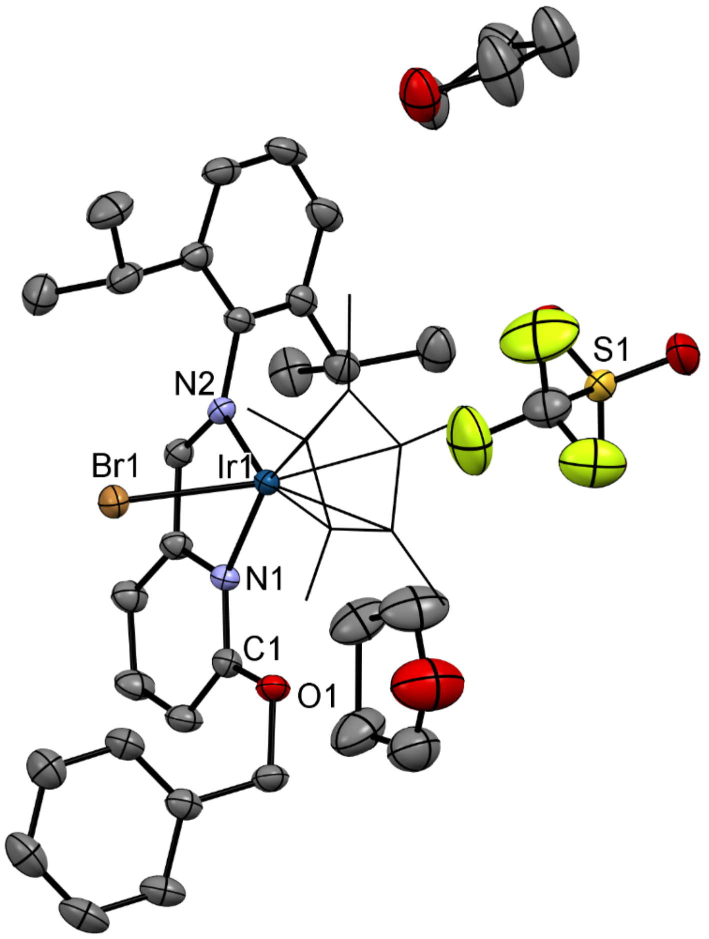

Figure S64. ORTEP of **12·2THF**

Non-H atoms are represented by 30% probability ellipsoids. H atoms are omitted for clarity. Bond lengths (Å): Ir1-N1 2.129(2); Ir1-N2 2.113(2); Ir1-Br1 2.5280(6); C1-O1 1.335(4). Bond angles (°): N1-Ir1-N2 76.06(9); N1-Ir1-Br1 80.09(7); N2-Ir1-Br1 89.42(7); N1-C5-O1 113.7(3).

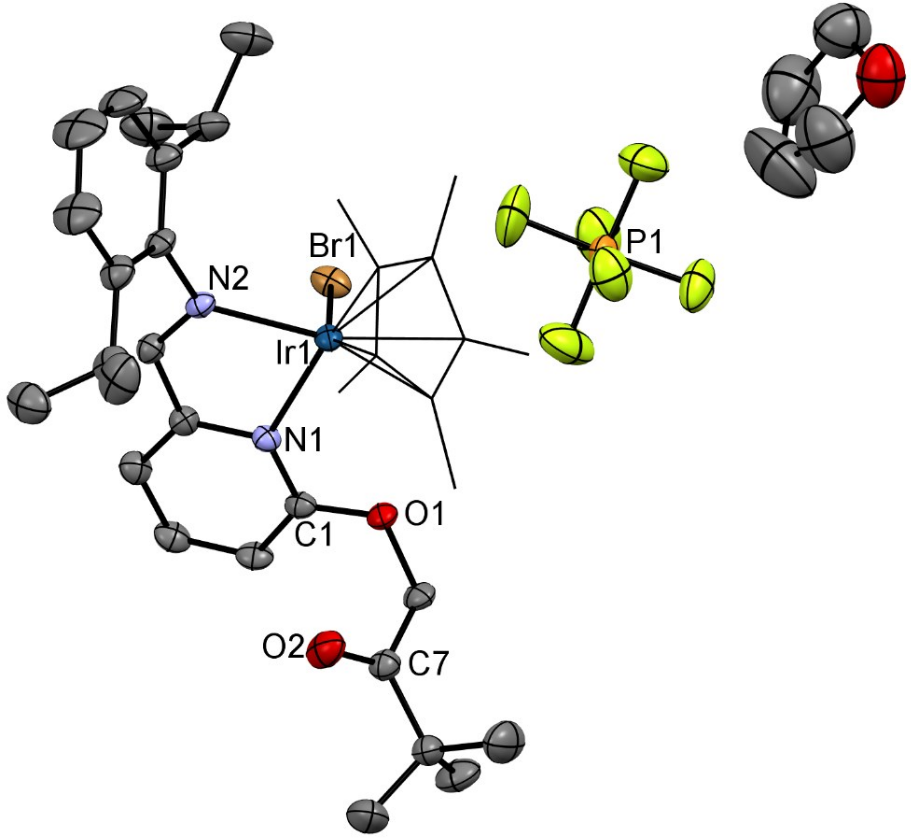

Figure S65. ORTEP of **13·THF**

Non-H atoms are represented by 30% probability ellipsoids. H atoms are omitted for clarity. Bond lengths (Å): Ir1-N1 2.138(1); Ir1-N2 2.112(2); Ir1-Br1 2.5465(5); C1-O1 1.338(2); C7-O2 1.206(3). Bond angles (°): N1-Ir1-N2 76.65(6); N1-Ir1-Br1 79.88(4); N2-Ir1-Br1 89.10(4); N1-C1-O1 114.3(1).

Table S1. Crystallographic data of **3·C₇H₈**, **4**, **5·MeOH**, **6**, **12·2THF**, **13·THF**.

	**3·C_7H_8**	**4**	**5·MeOH**
Empirical formula	$C_{35}H_{44}ClIrN_2O$	$C_{28}H_{36}IIrN_2O$	$C_{30}H_{44}Cl_3IrN_2O_3Zn$
Formula mass [g·mol^{-1}]	736.37	735.69	844.59
λ [Å]	0.71073	0.71073	0.71073
T [K]	150(2)	150(2)	150(2)
Crystal system	Monoclinic	Orthorhombic	Orthorhombic
Space group	*P21/c*	*Pna21*	*Pna21*
Crystal size [mm]	*0.210 x 0.191 x 0.046*	*0.336 x 0.128 x 0.061*	*0.589 × 0.141 × 0.036*
Crystal habit	*Dark orange block*	*Red block*	*Orange needle*
a [Å]	12.3978(7)	17.1221(3)	21.8472(5)
b [Å]	13.4931(6)	11.5611(2)	8.6463(2)
c [Å]	18.5679(10)	12.8601(2)	17.4762(4)
α [°]	90	90	90
β [°]	93.950(2)	90	90
γ [°]	90	90	90
V [Å^3]	3098.7(3)	2545.66(7)	3301.21(13)
Z	4	4	4
$\rho_{calcd.}$[g·cm^{-3}]	1.578	1.920	1.699
μ [mm^{-1}]	4.425	6.481	5.029
F(000)	1480	1424	1680
θ range [°]	2.439 - 25.999	2.369 - 27.109	2.628 - 28.293
Reflections collected	50236	34365	233160
Independent reflections	6090 [R(int) = 0.0440]	5372 [R(int) = 0.0315]	8141 [R(int) = 0.0402]
Absorption correction	multi-scan	multi-scan	multi-scan
T_{max} / T_{min}	0.7463 / 0.4730	0.4306 / 0.7455	0.4591 / 0.7457
Refinement method	Full-matrix least-squares on F^2	Full-matrix least-squares on F^2	Full-matrix least-squares on F^2
Data / restraints / parameters	6090 / 381 / 371	5372 / 313 / 307	8141 / 376 / 353
Final R indices [I > 2σ(I)]	R1 = 0.0239<br>wR2 = 0.0453	R1 = 0.0180<br>wR2 = 0.0424	R1 = 0.0147<br>wR2 = 0.0376
R indices (all data)	R1 = 0.0291<br>wR2 = 0.0464	R1 = 0.0181<br>wR2 = 0.0424	R1 = 0.0152<br>wR2 = 0.0380
Goodness-of-Fit (GoF)	1.160	1.114	1.065
Diff. peak/hole [eÅ^{-3}]	0.716 / -0.993	0.846 / -1.199	1.166 / -0.643

	**6**	**12·2THF**	**13·THF**
Empirical formula	$C_{60}H_{87}Cl_4IrN_4O_6RuZn$	$C_{44}H_{51}BrF_3IrN_2O_6S$	$C_{40}H_{59}BrIrN_2O_{3.5}F_6P$
Formula mass [g·mol^{-1}]	1460.77	1073.10	1040.97
λ [Å]	0.71073	0.71073	0.71073
T [K]	150(2)	150(2)	150(2)
Crystal system	Triclinic	Orthorhombic	Monoclinic
Space group	*P-1*	*Pbca*	*C2/c*
Crystal size [mm]	*0.300 x 0.174 x 0.146*	*0.374 × 0.132 × 0.024*	*0.578 × 0.144 × 0.139*
Crystal habit	*Dark orange block*	*Orange plate*	*Orange block*
a [Å]	10.8483(3)	19.1633(4)	20.3207(4)
b [Å]	16.5999(4)	21.7224(5)	17.8650(4)
c [Å]	17.2152(4)	21.8575(5)	25.9475(6)
α [°]	78.4010(10)	90	90
β [°]	84.8110(10)	90	110.6470(10)
γ [°]	79.2380(10)	90	90
V [Å^3]	2978.89(13)	9098.7(4)	8814.7(3)
Z	2	8	8
$\rho_{calcd.}$[g·cm^{-3}]	1.629	1.567	1.569
μ [mm^{-1}]	3.111	3.918	4.036
F(000)	1484	4320	4176
θ range [°]	2.695 - 26.999	2.145 - 26.385	1.998 - 28.297
Reflections collected	164637	177517	182005
Independent reflections	12889 [R(int) = 0.0407]	9305 [R(int) = 0.0810]	10873 [R(int) = 0.0418]
Absorption correction	multi-scan	multi-scan	multi-scan
T_{max} / T_{min}	0.7457 / 0.5479	0.7454 / 0.5092	0.7457 and 0.4843
Refinement method	Full-matrix least-squares on F^2	Full-matrix least-squares on F^2	Full-matrix least-squares on F^2
Data / restraints / parameters	12889 / 732 / 640	9305 / 509 / 560	10873 / 522 / 545
Final R indices [I > 2σ(I)]	R1 = 0.0215 wR2 = 0.0534	R1 = 0.0231 wR2 = 0.0465	R1 = 0.0182 wR2 = 0.0420
R indices (all data)	R1 = 0.0237 wR2 = 0.0547	R1 = 0.0398 wR2 = 0.0546	R1 = 0.0202 wR2 = 0.0433
Goodness-of-Fit (GoF)	1.066	1.094	1.097
Diff. peak/hole [eÅ^{-3}]	0.855 / -1.026	1.103 / -0.549	0.707 / -0.881

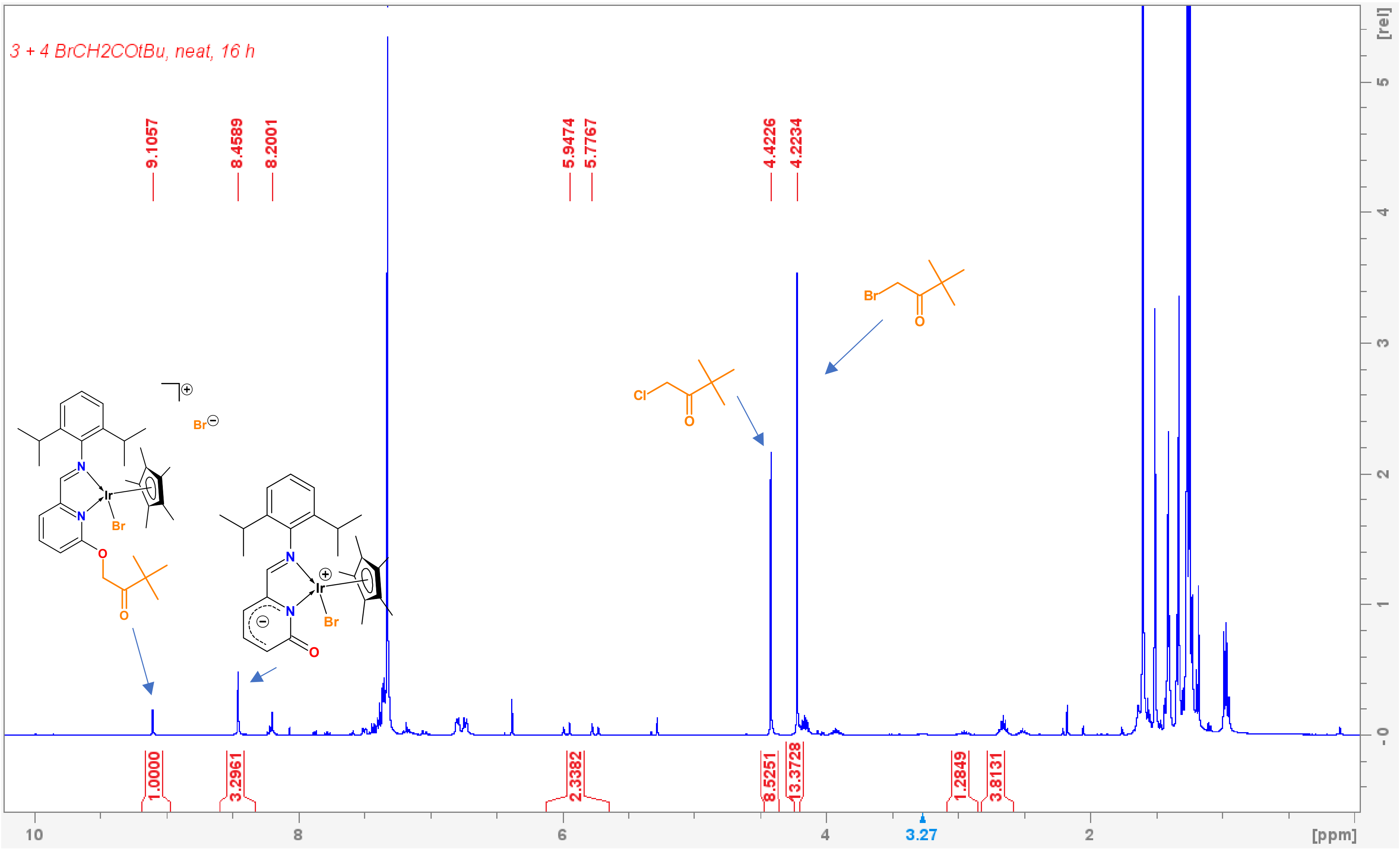

Figure S66. ^{1}H NMR ($CDCl_3$, 500.13 MHz, 300 K) of the reaction mixture of **3** with a minimal amount of $BrCH_2COtBu$ under neat conditions after 16 h showing consumption of Cl ligand by substrate.

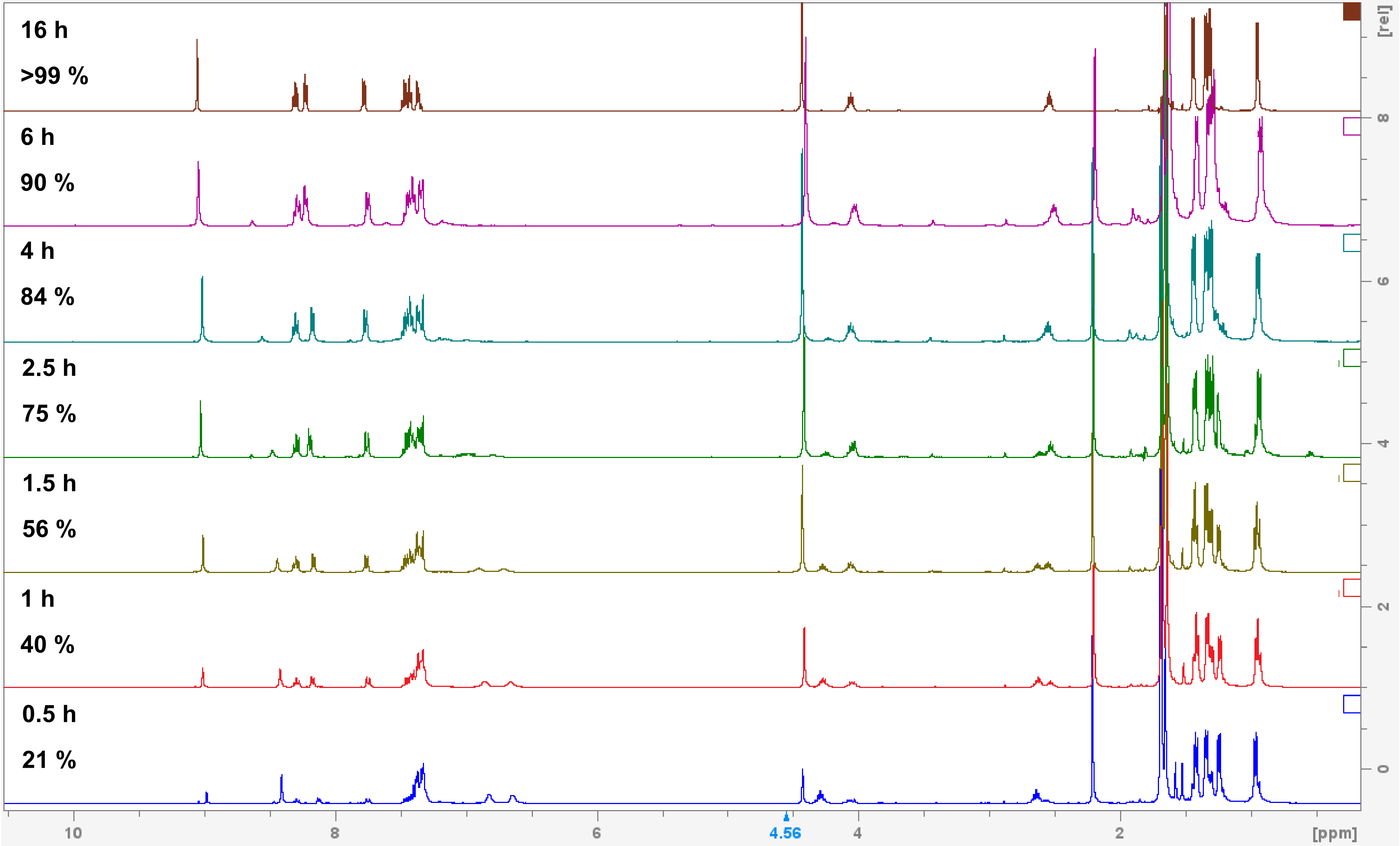

Figure S67. ^{1}H NMR ($CDCl_3$, 500.13 MHz, 300 K) of **3** + MeI kinetic study.

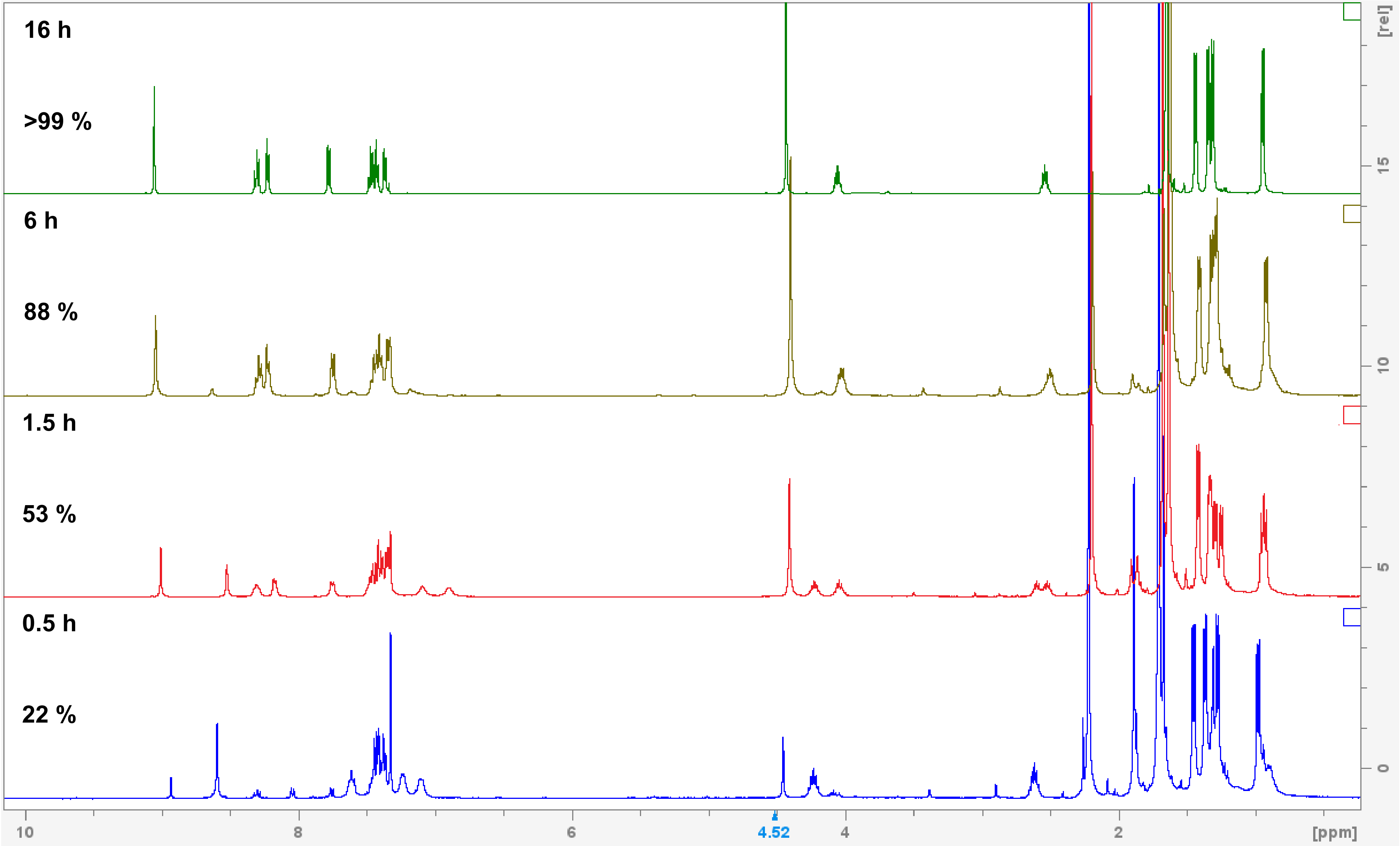

16 h
>99 %
6 h
88 %
1.5 h
53 %
0.5 h
22 %
10
8
6
4.52
4
2
[ppm]
[rel]
15
10
5
0

Figure S68. ^{1}H NMR ($CDCl_3$, 500.13 MHz, 300 K) of **4** + MeI kinetic study.

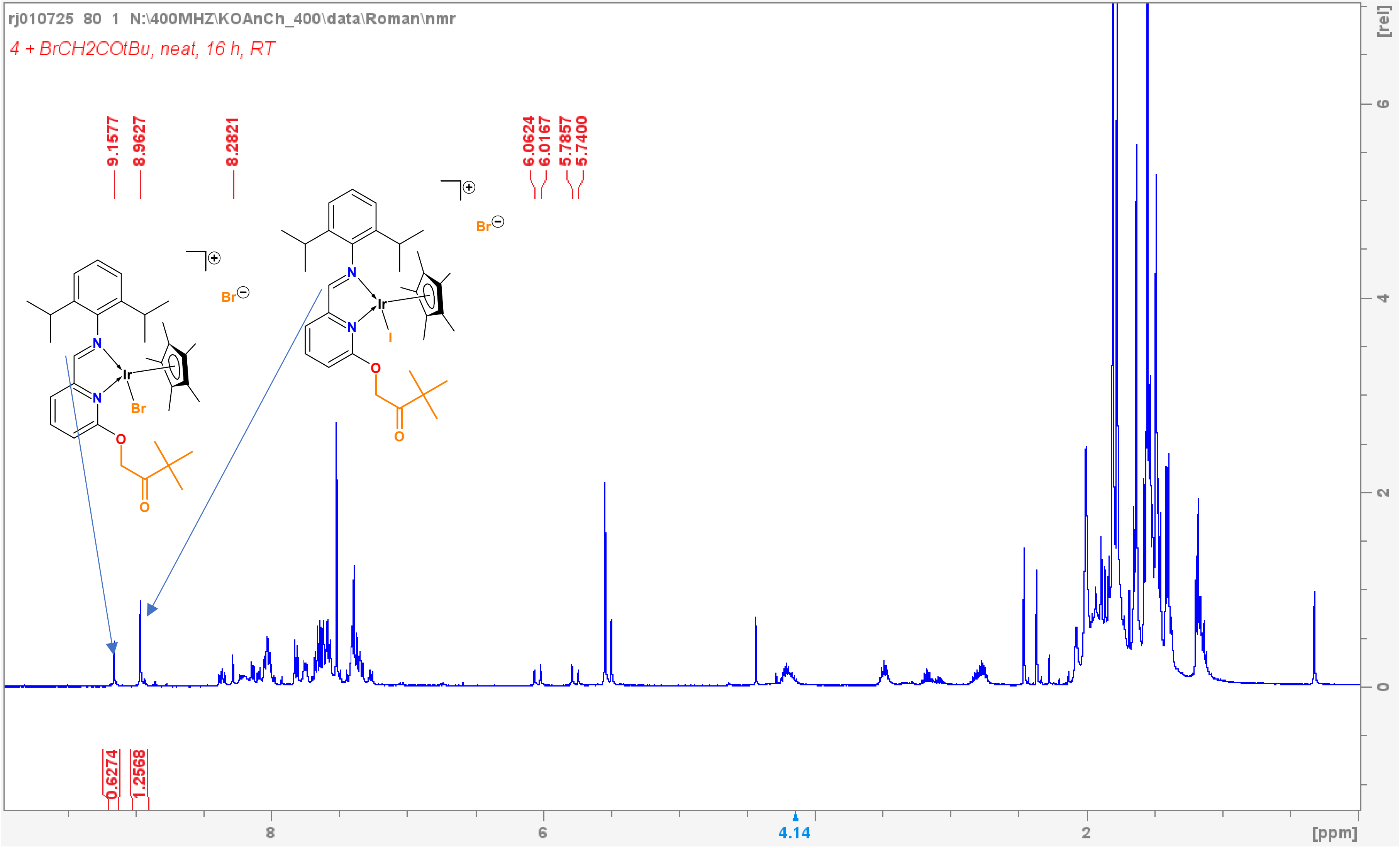

Figure S69. ^{1}H NMR ($CDCl_3$, 400.13 MHz, 300 K) of the reaction mixture of **4** and $BrCH_2COtBu$ under similar conditions as for **3** and $BrCH_2COtBu$. Neat, 16 h, RT.

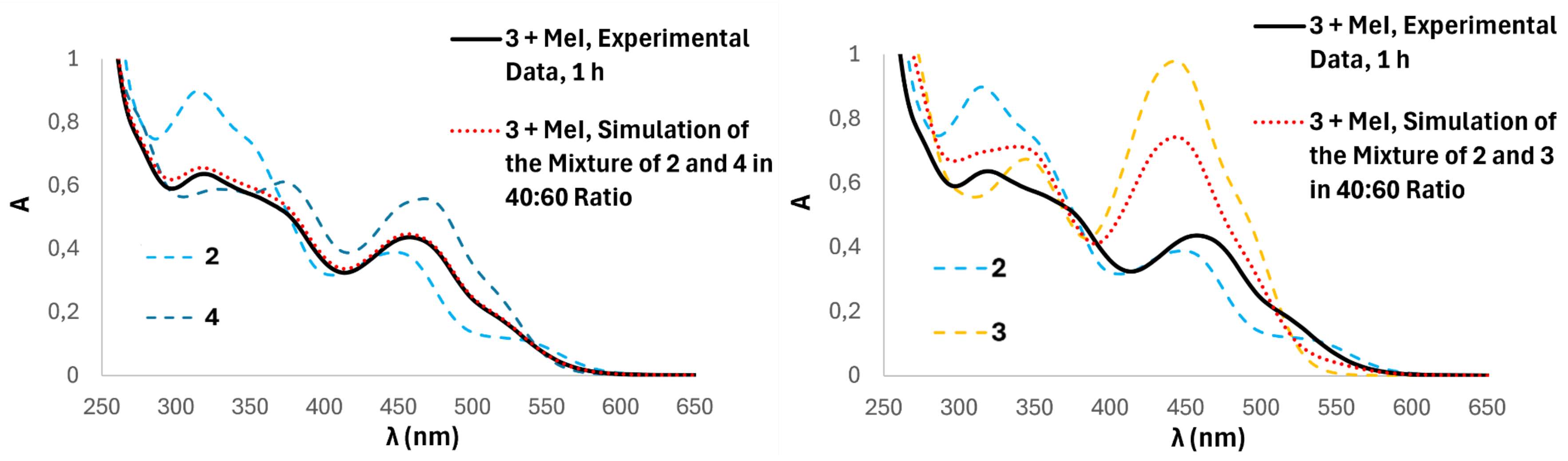

Figure S70. Left: Absorption spectra of the reaction mixture of **3** with MeI (black solid) recorded after 1 h. A simulated absorption spectrum (red dots) of the mixture of **2** and **4** in the 40:60 ratio (detected by ^{1}H NMR) obtained from experimental data of **2** and **4**: $A_{simulated} = 0.4 \cdot A(\mathbf{2}) + 0.6 \cdot A(\mathbf{4})$ closely matches the experimental spectrum of the mixture and confirms the absence of the starting compound **3** after 1 h. **Right**: For comparition, similar model considering mixture of starting **3** and product **2** in 40:60 ratio. Simulation does not match experimental data in this case.

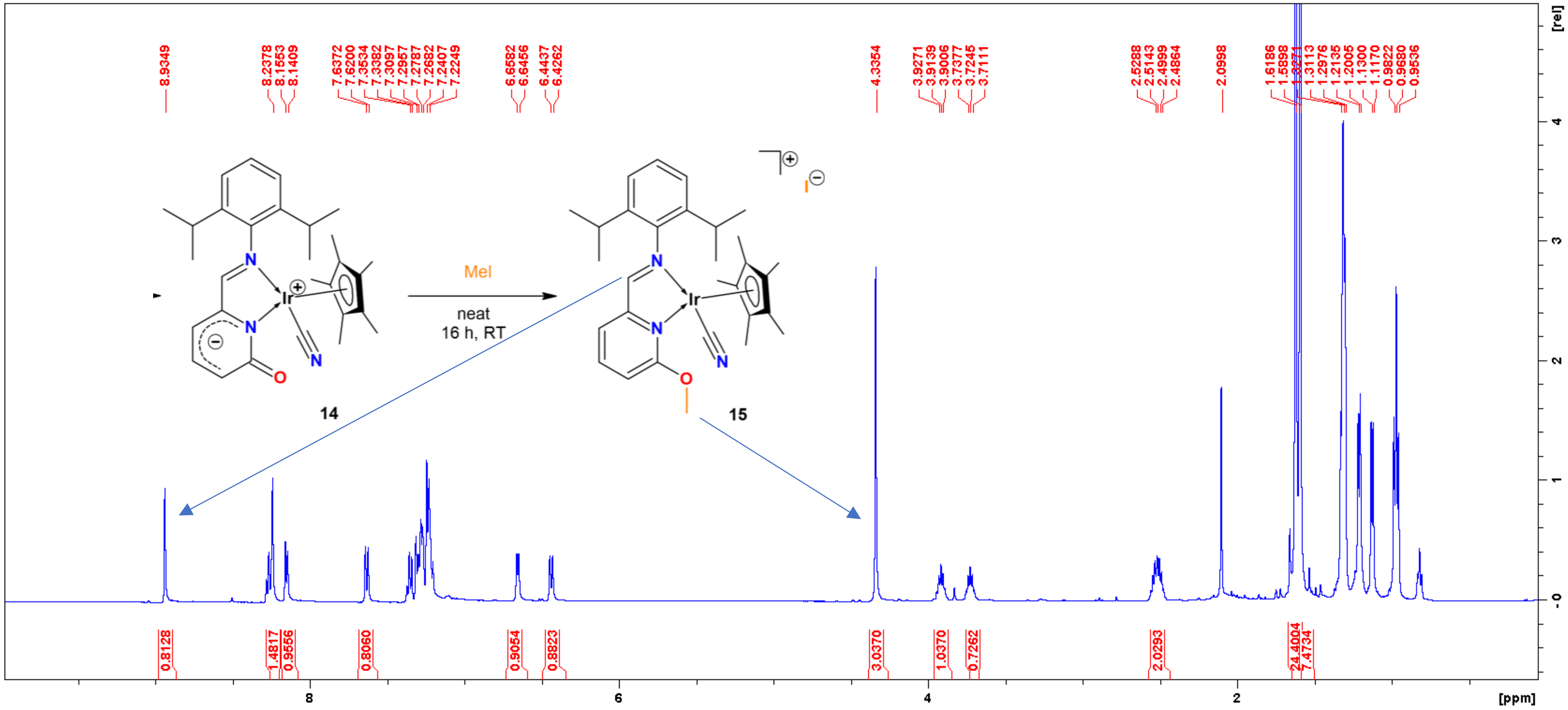

Figure S71. ^{1}H NMR ($CDCl_3$, 500.13 MHz, 300 K) of the reaction mixture of **14** with MeI and **15**.

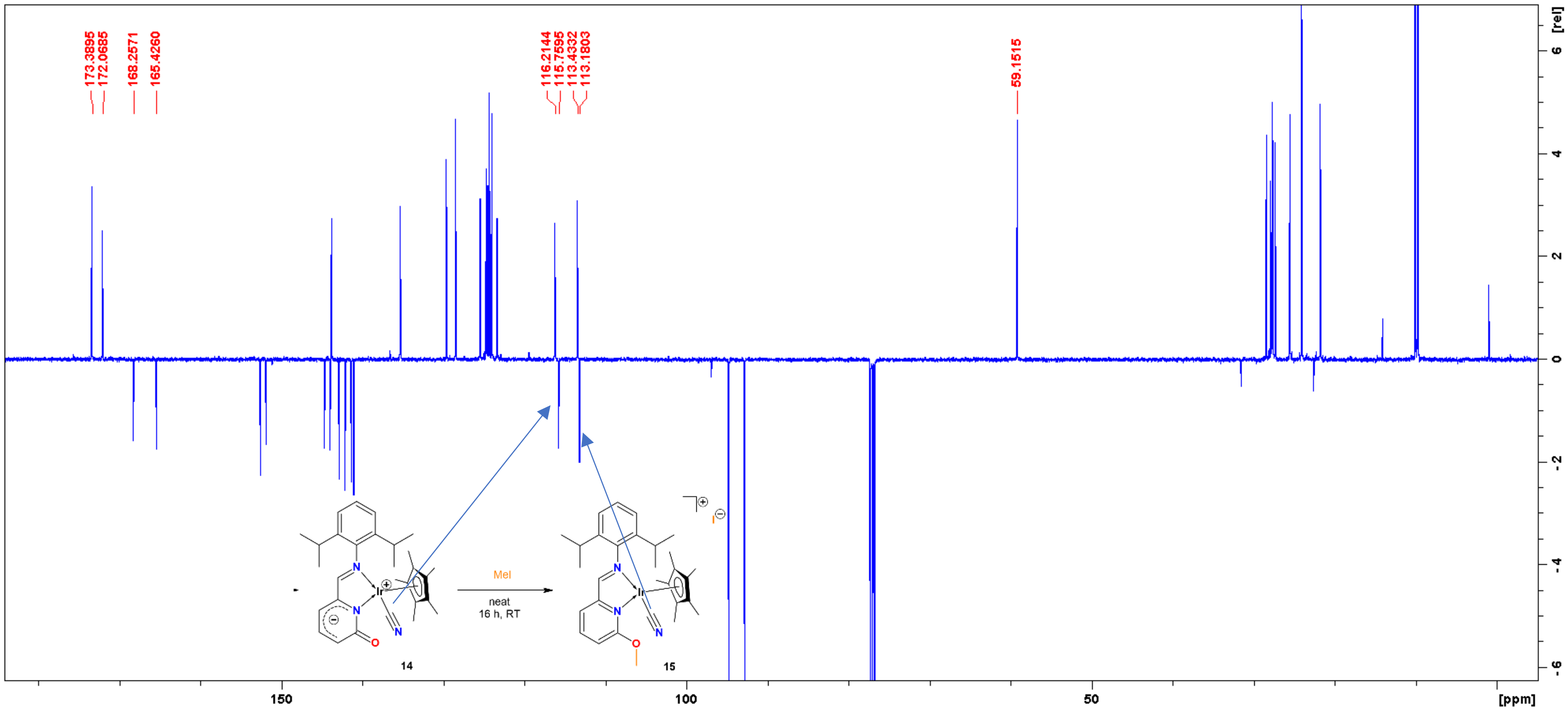

Figure S72. $^{13}C\{^1H\}$ NMR ($CDCl_3$, 125.78 MHz, 300 K) of the reaction mixture of **14** with MeI and **15**.

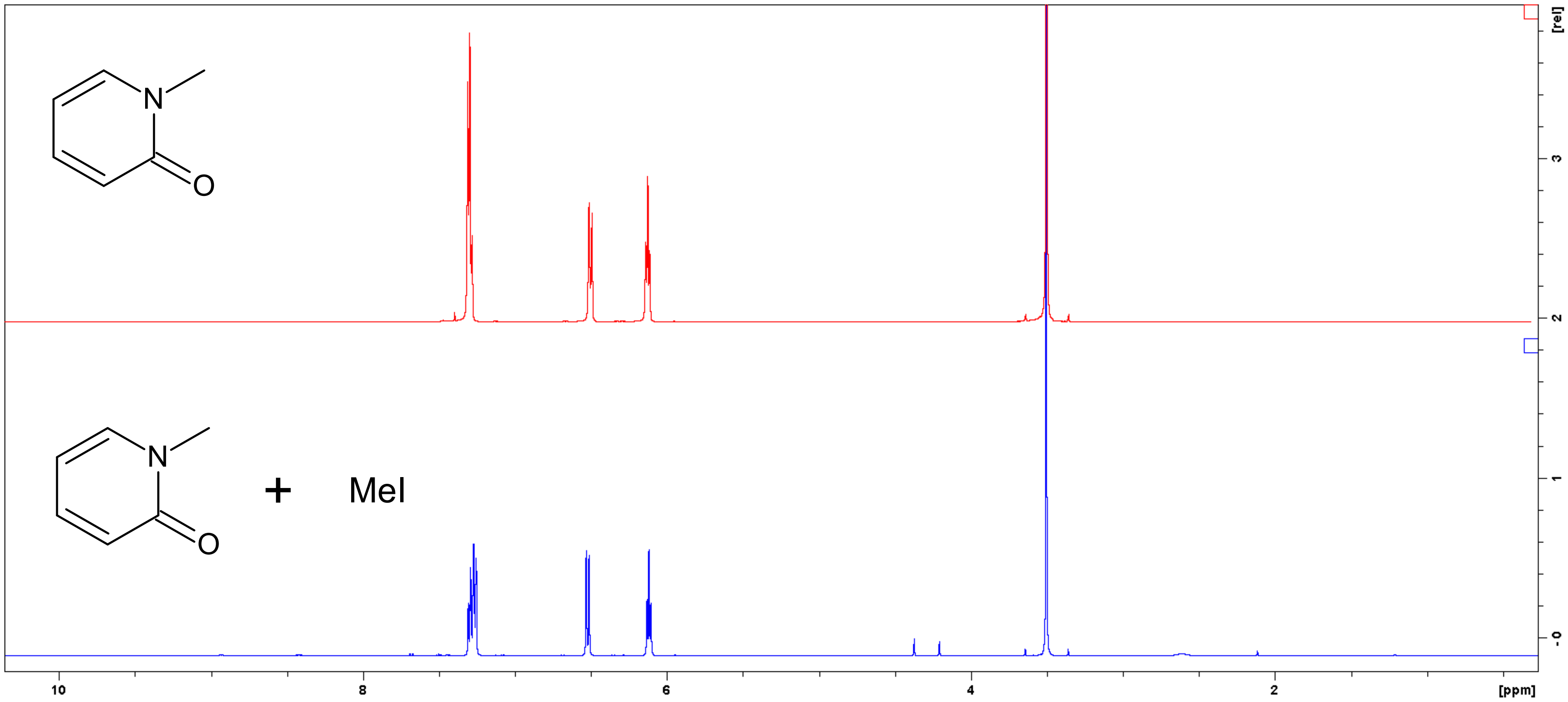

Figure S73. ^{1}H NMR ($CDCl_3$, 500.13 MHz, 300 K) of N-methylpyridone and its reaction with MeI under neat conditions, 16 h, RT.

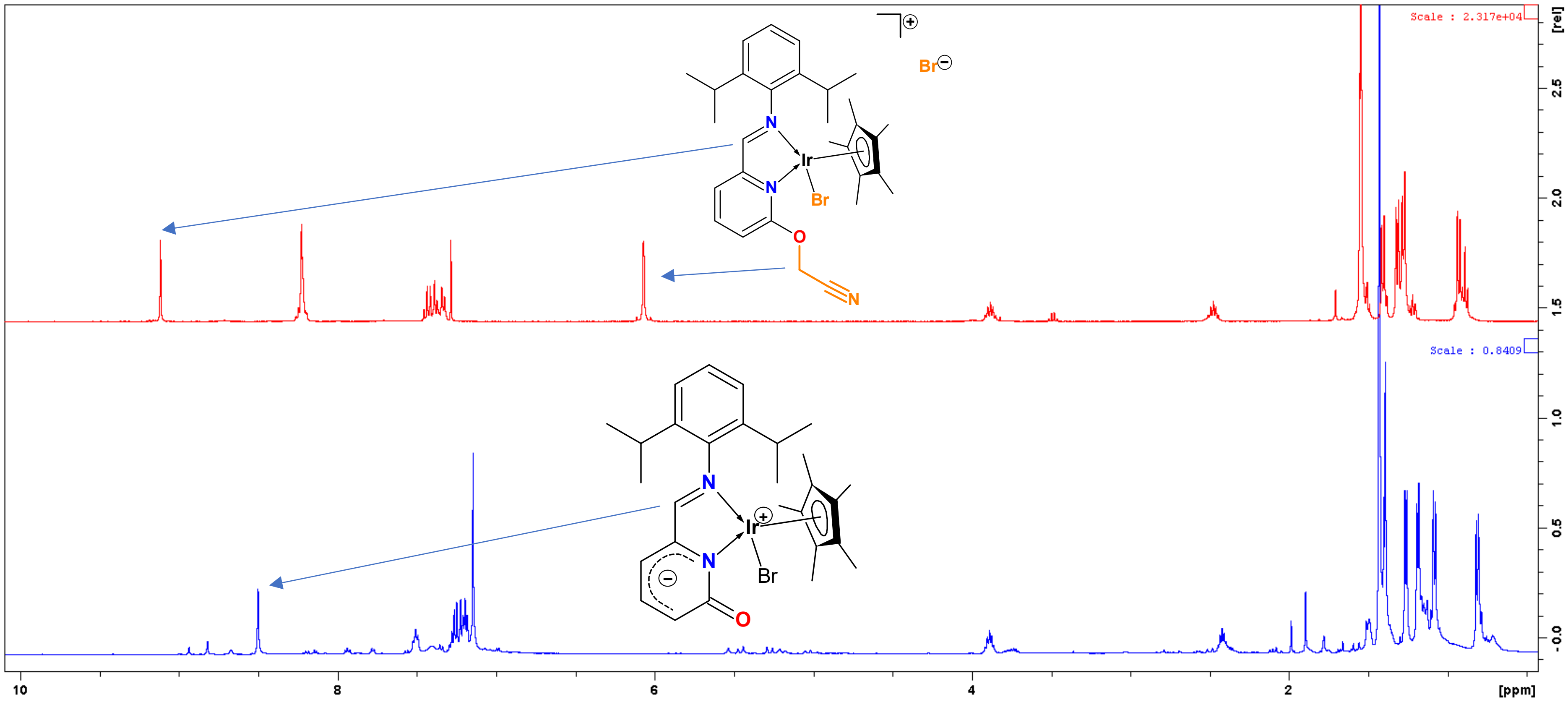

Figure S74. ^{1}H NMR ($CDCl_3$, 400.13 MHz, 300 K) of **10** before heating (red spectrum) and after heating in the solid phase (blue spectrum) with supposed major product.

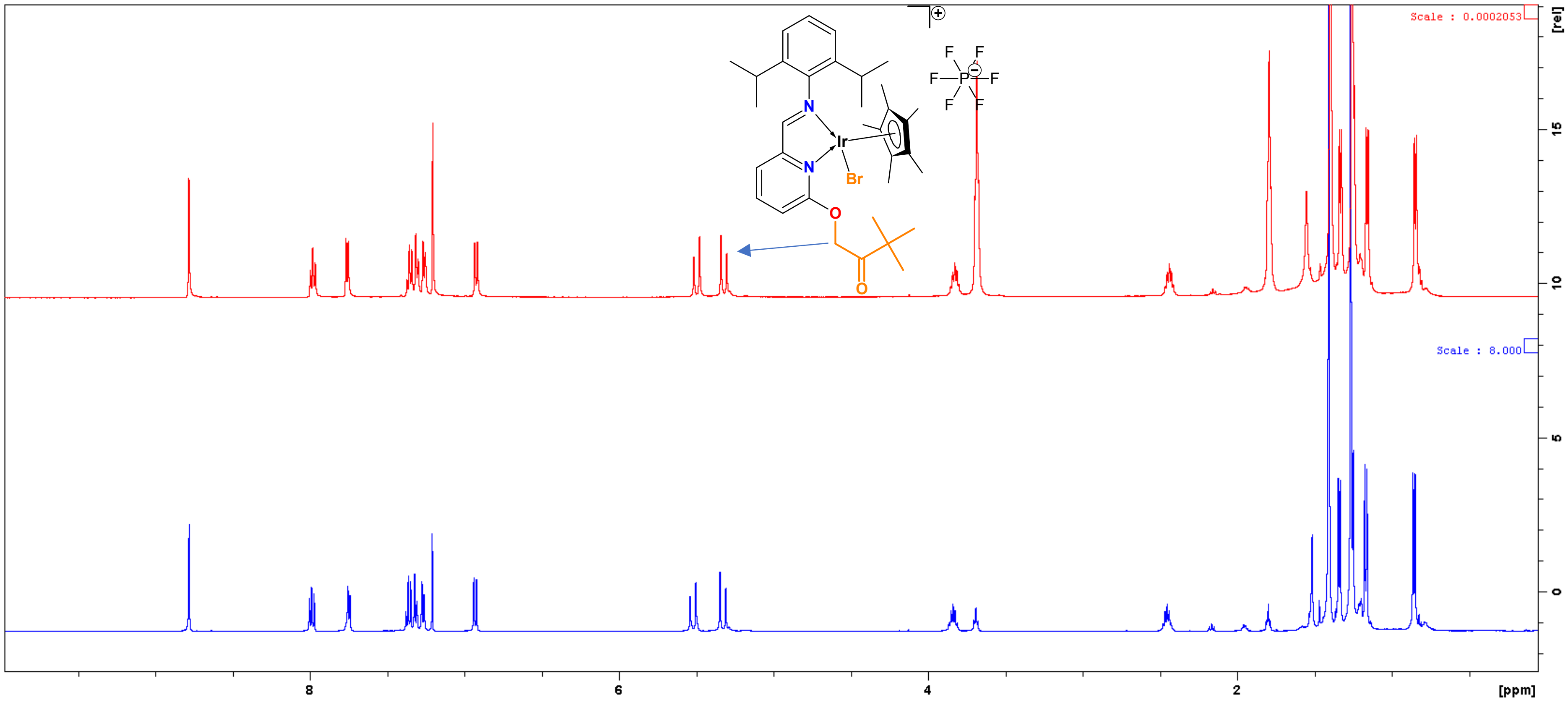

Figure S75. ^{1}H NMR ($CDCl_3$, 500.13 MHz, 300 K) of **13·THF** before heating (red spectrum) and after heating (blue spectrum) in the solid phase.

TD-DFT study of 3

Table S2. TD-DFT calculated vertical electronic transitions from the DFT optimized ground state S_0 of 3 to the first 20 singlet and triplet excited states.

Transition			Energy (cm^{-1})	Wavelength (nm)	Oscillator strength f
S0	->	S1	21476.3	465.6	0.0519
S0	->	S2	22465.5	445.1	0.2359
S0	->	S3	27253.8	366.9	0.0064
S0	->	S4	27580.8	362.6	0.0397
S0	->	S5	28165.3	355.0	0.0146
S0	->	S6	28501.2	350.9	0.0403
S0	->	S7	29495.2	339.0	0.0163
S0	->	S8	30709.2	325.6	0.0251
S0	->	S9	31156.7	321.0	0.0369
S0	->	S10	31412.7	318.3	0.0263
S0	->	S11	32468.3	308.0	0.0039
S0	->	S12	34186.3	292.5	0.0406
S0	->	S13	34888.6	286.6	0.0030
S0	->	S14	35581.8	281.0	0.0188
S0	->	S15	35831.4	279.1	0.0092
S0	->	S16	36263.7	275.8	0.0066
S0	->	S17	36888.7	271.1	0.0086

**S0**	**->**	**S18**	**37391.3**	**267.4**	**0.0412**
**S0**	**->**	**S19**	**37957.1**	**263.5**	**0.0023**
**S0**	**->**	**S20**	**38963.6**	**256.6**	**0.0057**
**S0**	**->**	**T1**	**14014.7**	**713.5**	
**S0**	**->**	**T2**	**20052.5**	**498.7**	
**S0**	**->**	**T3**	**22869.0**	**437.3**	
**S0**	**->**	**T4**	**24674.6**	**405.3**	
**S0**	**->**	**T5**	**24833.0**	**402.7**	
**S0**	**->**	**T6**	**26609.1**	**375.8**	
**S0**	**->**	**T7**	**27190.1**	**367.8**	
**S0**	**->**	**T8**	**27504.9**	**363.6**	
**S0**	**->**	**T9**	**28205.9**	**354.5**	
**S0**	**->**	**T10**	**28595.1**	**349.7**	
**S0**	**->**	**T11**	**30229.3**	**330.8**	
**S0**	**->**	**T12**	**30636.9**	**326.4**	
**S0**	**->**	**T13**	**31308.1**	**319.4**	
**S0**	**->**	**T14**	**31817.3**	**314.3**	
**S0**	**->**	**T15**	**32418.0**	**308.5**	
**S0**	**->**	**T16**	**32664.7**	**306.1**	
**S0**	**->**	**T17**	**33512.4**	**298.4**	
**S0**	**->**	**T18**	**34834.1**	**287.1**	
**S0**	**->**	**T19**	**35173.8**	**284.3**	
**S0**	**->**	**T20**	**36345.5**	**275.1**	

DFT and TD-DFT calculations for complex 3 were performed with the ORCA 5.0.1 program suite with tight SCF convergence criteria.[1] Geometry optimizations (gas-phase) omitting the respective anions were carried out with the BP86 functional[2] as implemented in ORCA, and a frequency analysis ensuring that the optimized structures correspond to energy minima. The def2-SVP[3] basis set was used for all atoms together with the auxiliary basis set SARC/J[4-8] in order to accelerate the computations within the framework of RI approximation. Relativistic effects were accounted for by employing the ZORA method as implemented for the def2-SVP basis set in Orca, and by employing the SARC-ZORA-TZVP[9,10] basis set for the metal atoms. Van der Waals interactions have been considered by an empirical dispersion correction (Grimme-D3BJ).[11,12] TD-DFT calculations for the first 20 singlet and triplet excited states of 3 were performed with the same basis sets, but the PBE0 functional[13,14] was used. Representations of electronic transition differences at isovalues of 0.001 were produced with orca_plot as provided by ORCA 5.0.1 and with Chimera.[15]

Photoluminescence and excitation spectra (Table S3) of complexes **1**, **3** and **5** were recorded both in solution (Figures S76-86) and in the solid state (Figures S88-98). All three complexes exhibited unusual deep red to near-IR emission with λ_{max} = 680-754 nm, of which the large apparent Stokes shifts suggest that the radiative process is $T_1 \rightarrow S_0$ phosphorescence in nature. It should be noted, that known half-sandwich Ir(III) complexes exhibit emissions in the blue to yellow region of the spectrum (λ_{max} = 417-625 nm).[16-18] The excitation spectra of these bands generally match their corresponding absorption spectra, confirming the origin of the emission from the molecular structure of the complexes (see Figure S86). The excited-state relaxation mechanism was investigated by measuring the photoluminescence lifetimes (Figure S87). In all cases, the observed emission lifetimes are found in the nanosecond regime. Albeit the photoluminescence quantum yields (PLQY) were not measurable, i.e. $\Phi < 0.01$, an upper limit for the radiative rate constant k_r can be defined (Table S3). Consequently, we estimate maximum values for k_r of ca. 10^5 s^{-1}, clearly confirming the assignment of spin-forbidden triplet excited states being involved. We note that despite the Ir(d) orbitals contributing to the CT processes, the spin-orbit coupling (SOC) between the S_1 state and the triplet state manifold appears to be moderate as residual fluorescence can be observed in solution between 450-600 nm, hinting at non-quantitative intersystem-crossing $S_1 \rightarrow T_n$, although one would expect Φ_{ISC} = 1.0 as usually observed for Ir^{III} complexes. The high energy emission is not observed in the solid state, where the longer lifetimes hint at more intense phosphorescence due to decreased non-radiative decay in the rigid environment (Table S3 and Figure S98).

Table S3. Photophysical data of **1**, **3** and **5** in DCM solution and in the solid state at 297 K.

	Medium	λ_{ex} [nm]	λ_{em}^{max} [nm]	τ_{av} [ns]	χ^2 of fit	Φ	k_r [10^5 s^{-1}]
**1**	DCM	490	701	29	1.23	< 1%	3.5
	Solid	490	715	95	1.08		
**3**	DCM	480	742	26	1.10	< 1%	3.8
	Solid	480	754	30	1.28		
**5**	DCM	465	680	101	1.06	< 1%	1.0
	Solid	470	703	161	1.09		

Photophysical Properties of 1, 3 and 5 in the DCM solution

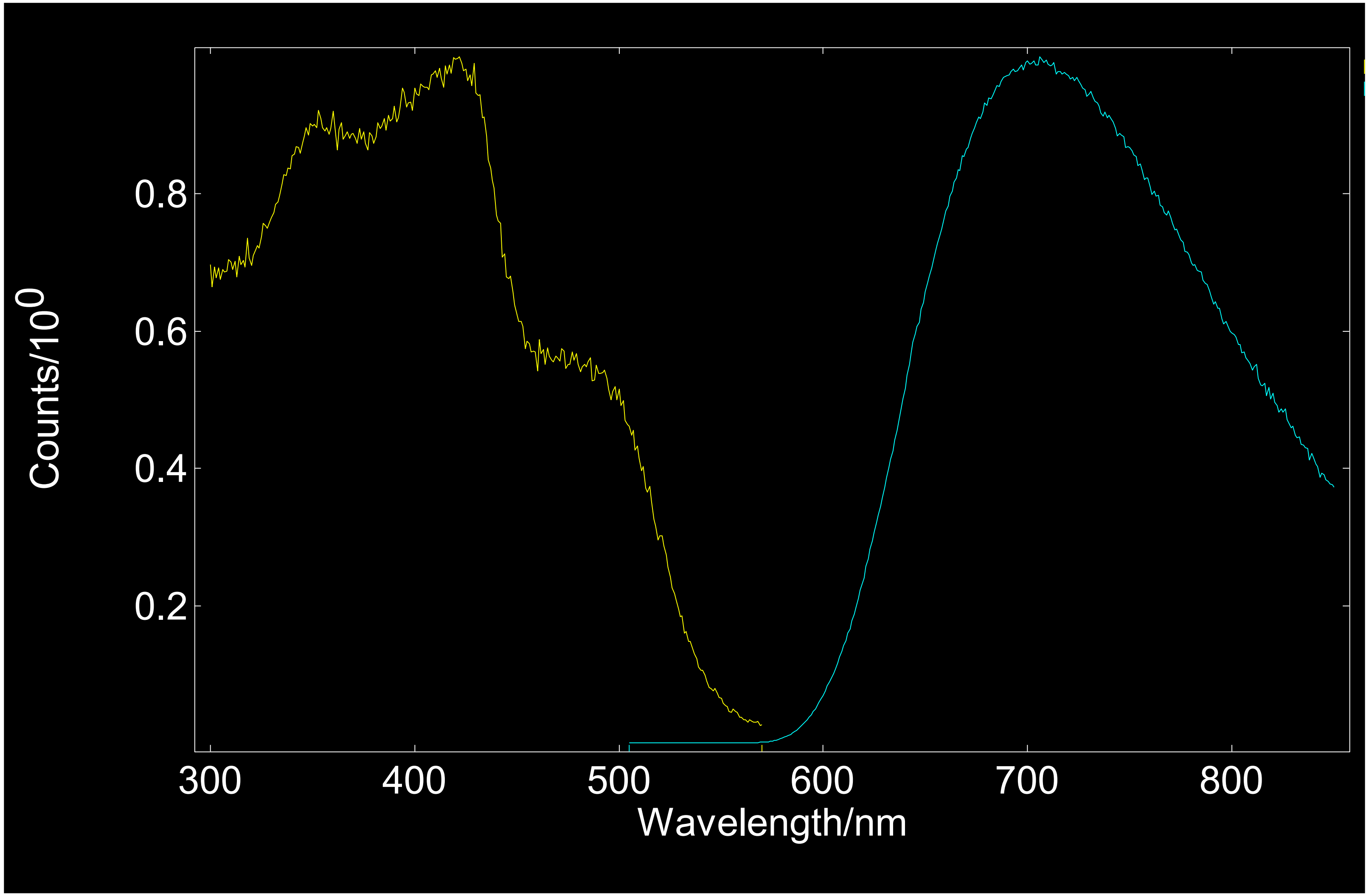

Figure S76. Excitation (yellow) and emission (blue) spectra of **1**, DCM solution, 293 K, λ_{ex} = 490 nm.

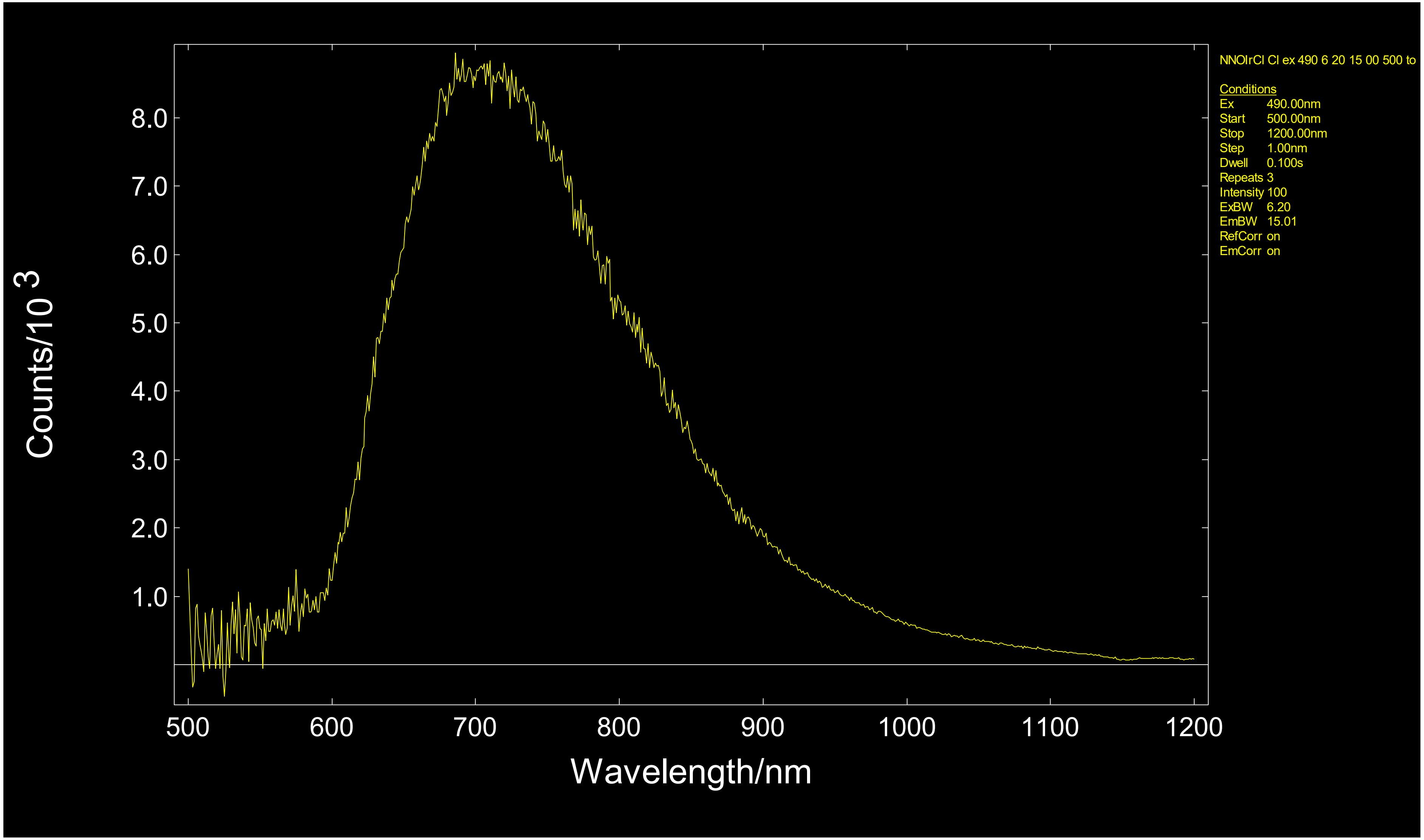

Figure S77. Emission spectrum of **1**, DCM solution, 293 K, λ_{ex} = 490 nm, NIR detector.

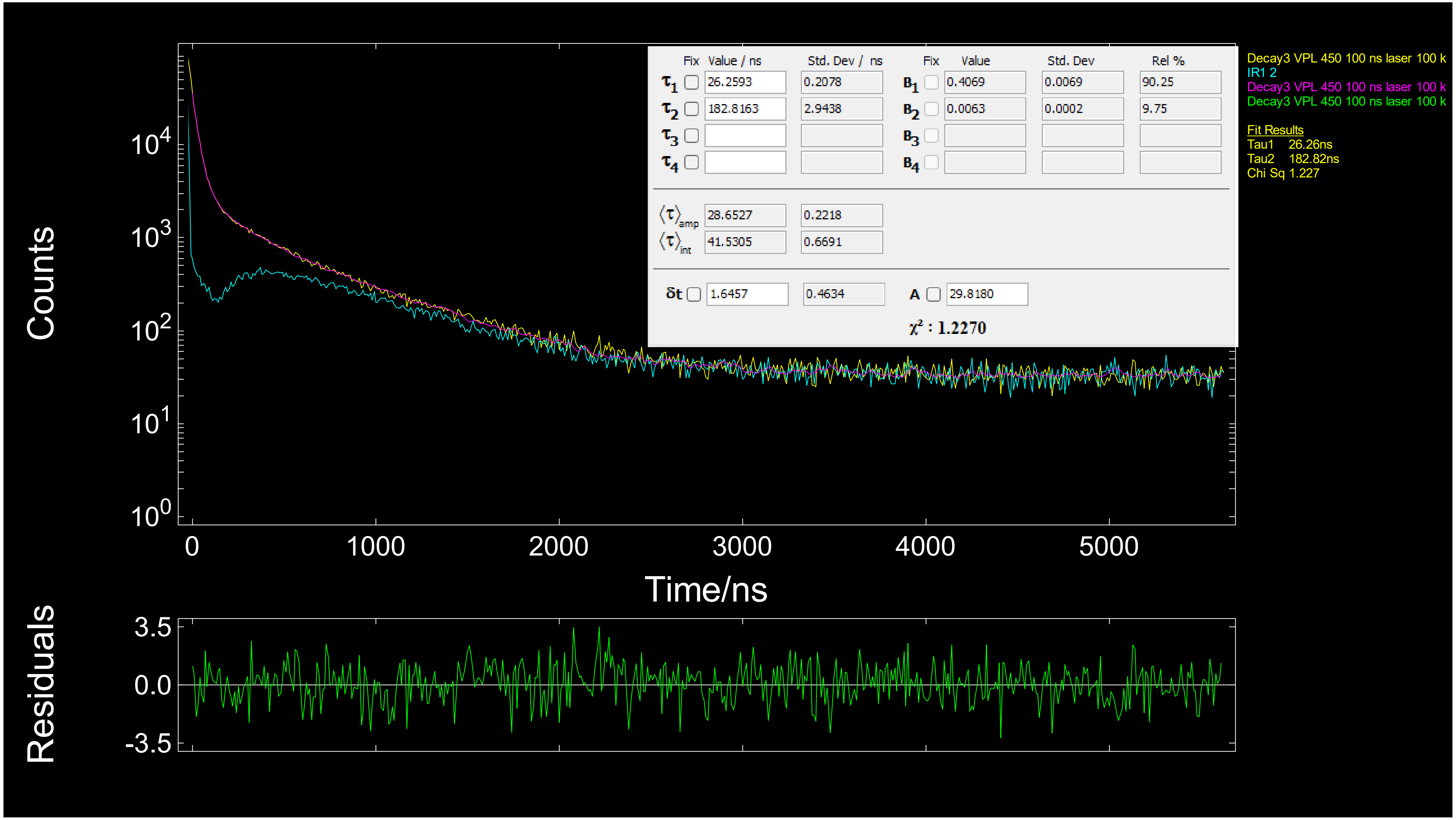

Figure S78. Experimental time-resolved luminescence decay (yellow) with IRF (blue) of **1**, DCM solution, 293 K. Numerical biexponential reconvulation fit (purple).

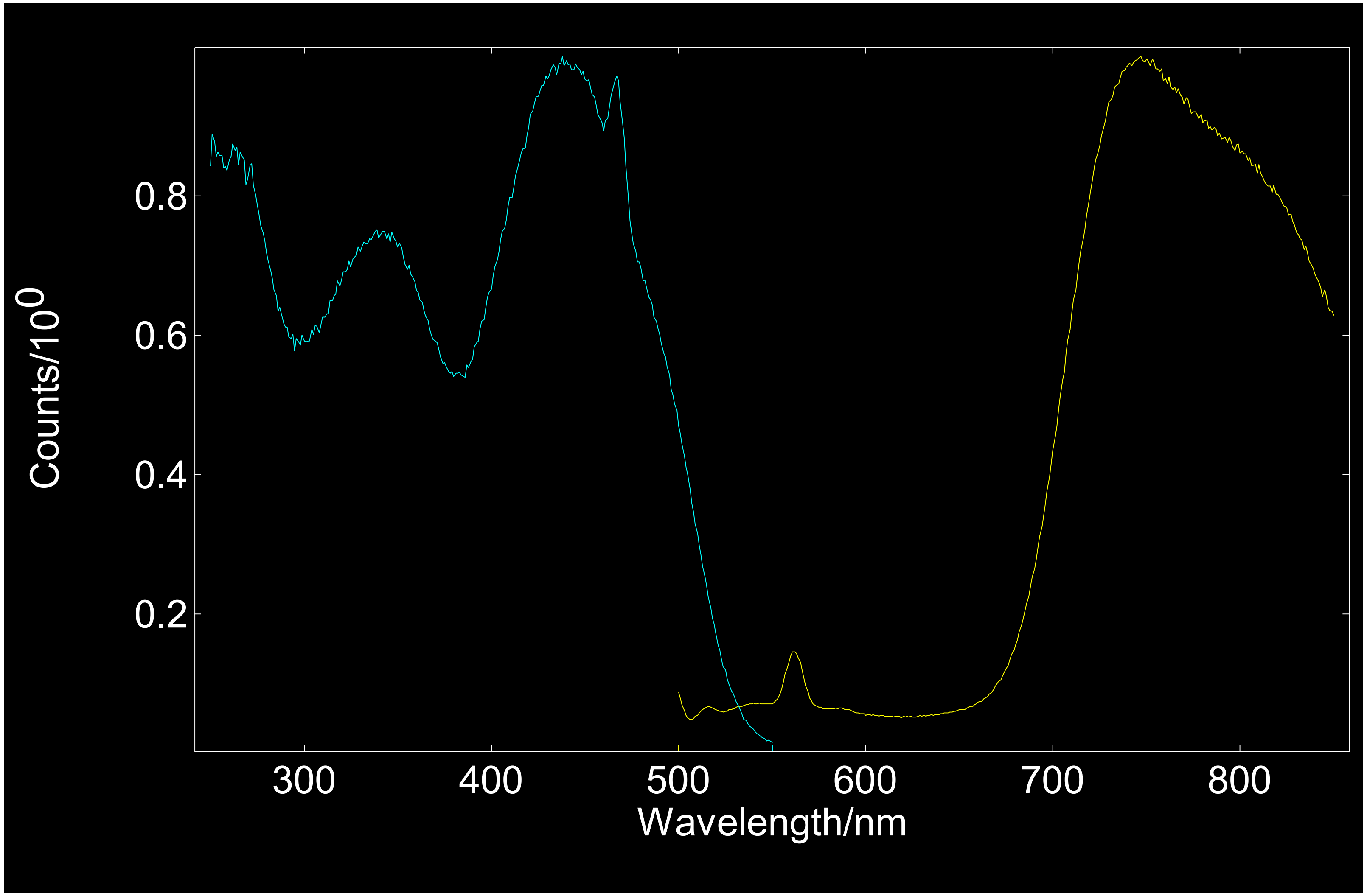

Figure S79. Excitation (blue) and emission (yellow) spectra of **3**, DCM solution, 293 K, λ_{ex} = 480 nm.

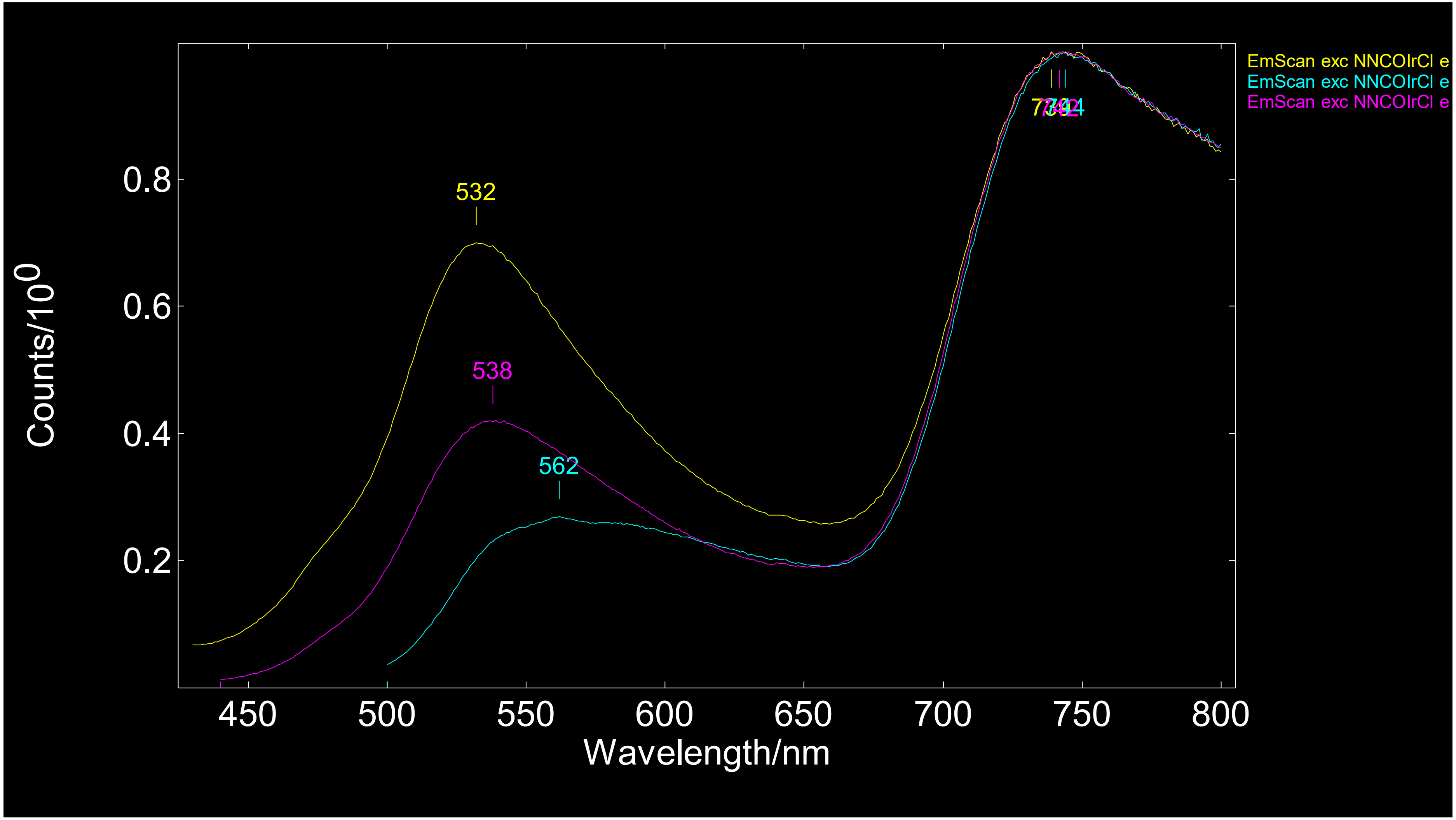

Figure S80. Emission spectra of **3**, DCM solution, 293 K, λ_{ex} = 480 nm (blue), λ_{ex} = 420 nm (purple) and λ_{ex} = 400 nm (yellow).

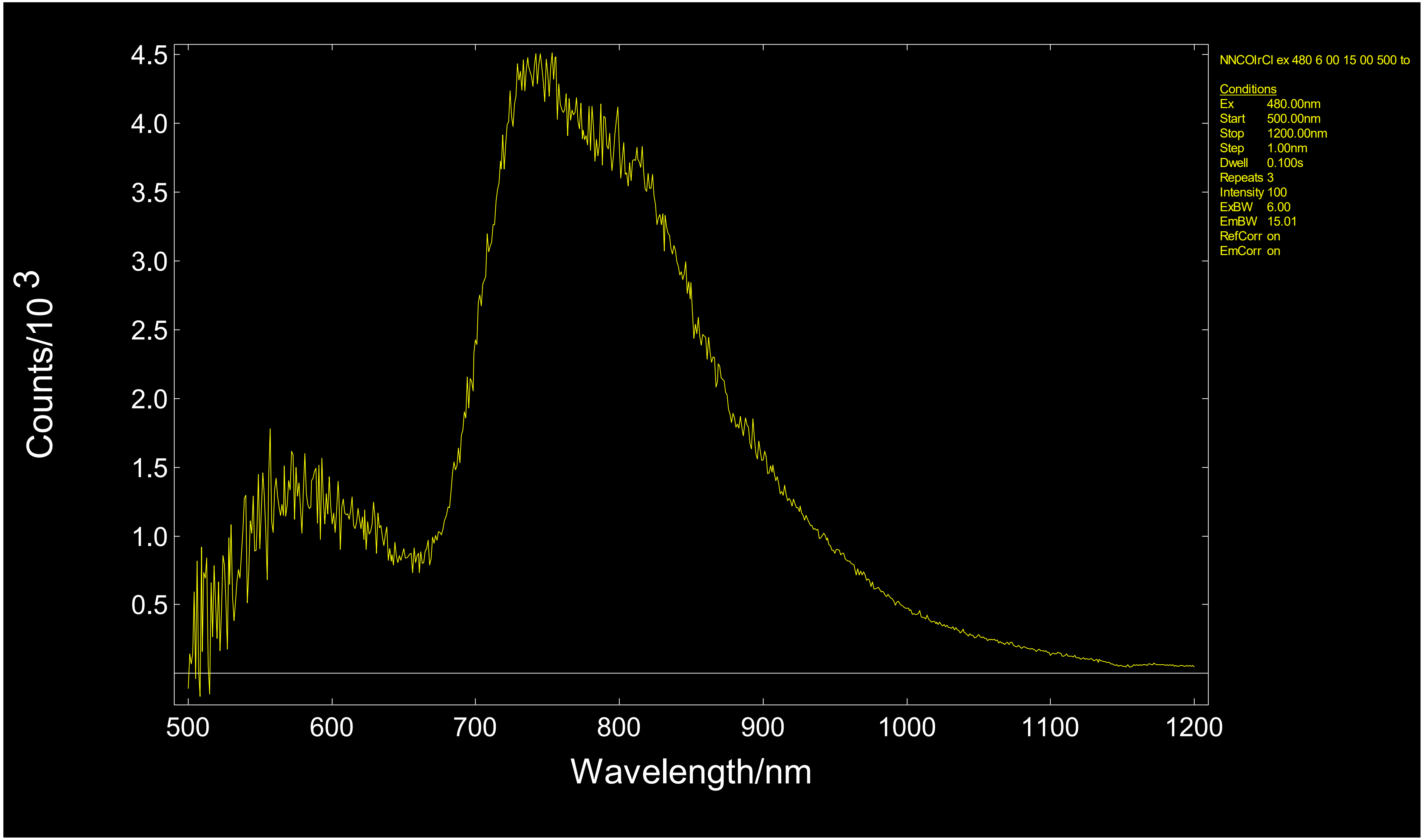

Figure S81. Emission spectrum of **3**, DCM solution, 293 K, λ_{ex} = 480 nm, NIR detector.

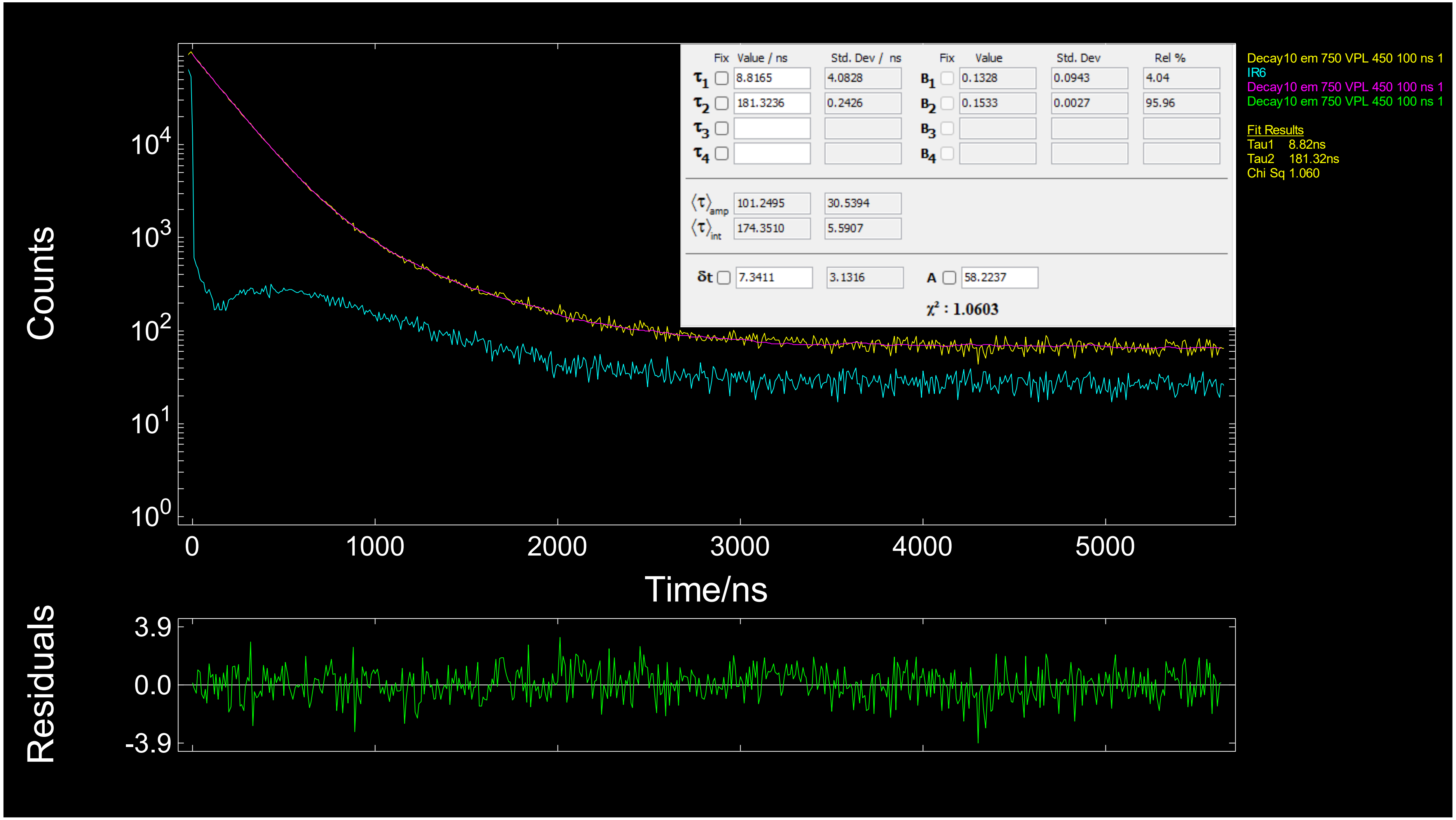

Figure S82. Experimental time-resolved luminescence decay (yellow) with IRF (blue) of **3**, DCM solution, 293 K. Numerical biexponential reconvulation fit (purple).

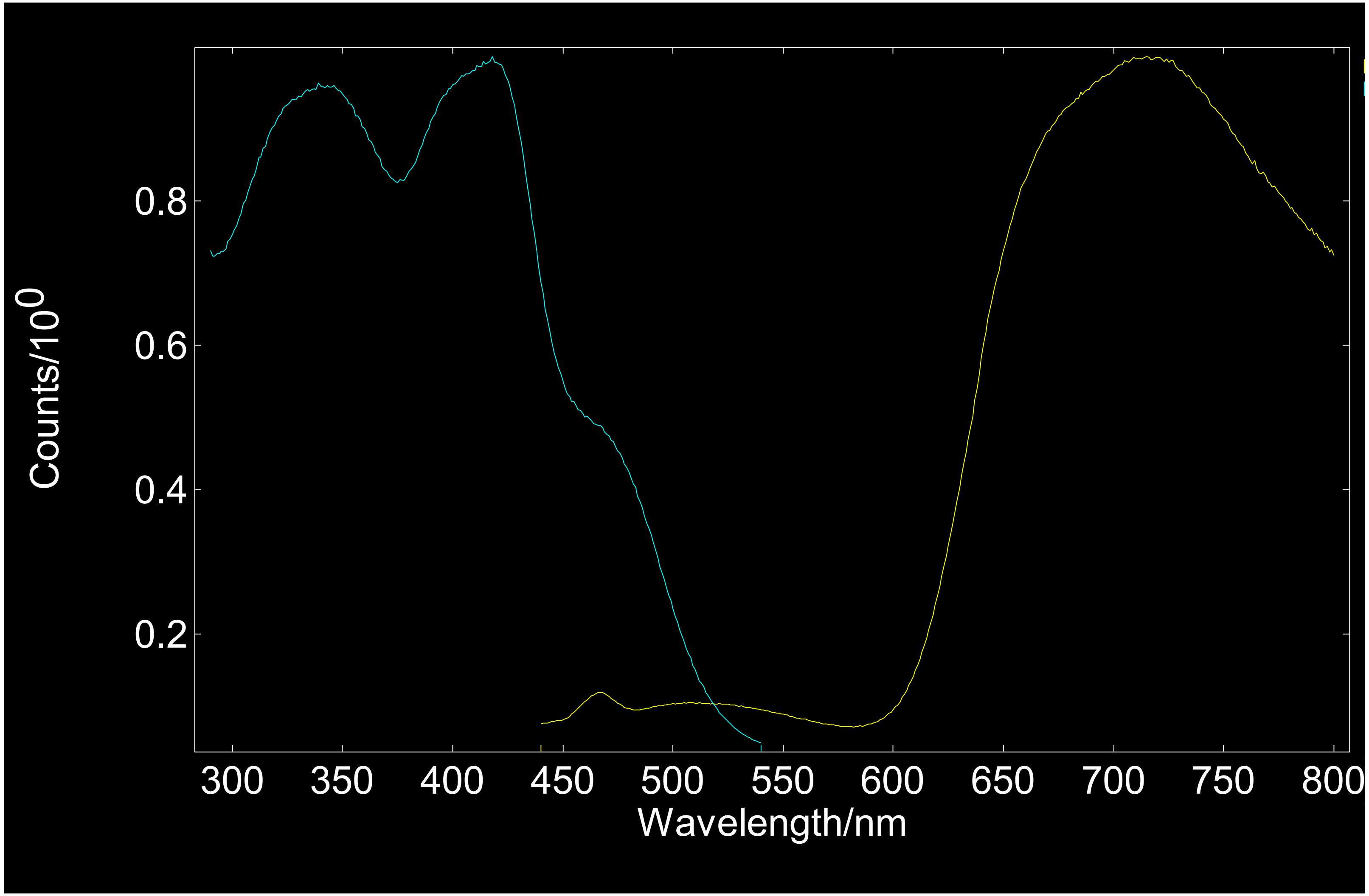

Figure S83. Excitation (blue) and emission (yellow) spectra of **5**, DCM solution, 293 K, λ_{ex} = 465 nm.

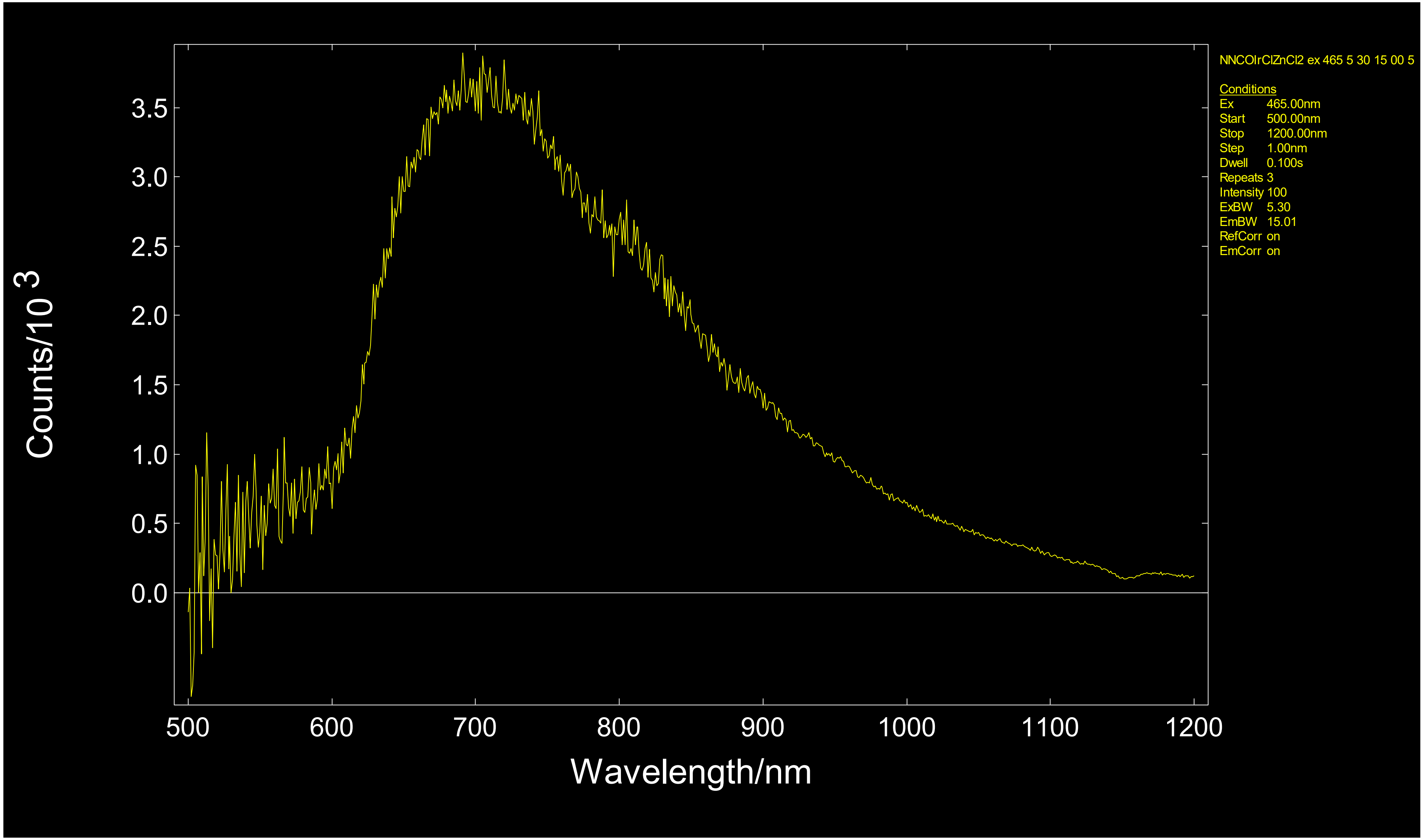

Figure S84. Emission spectrum of **5**, DCM solution, 293 K, λ_{ex} = 465 nm, NIR detector.

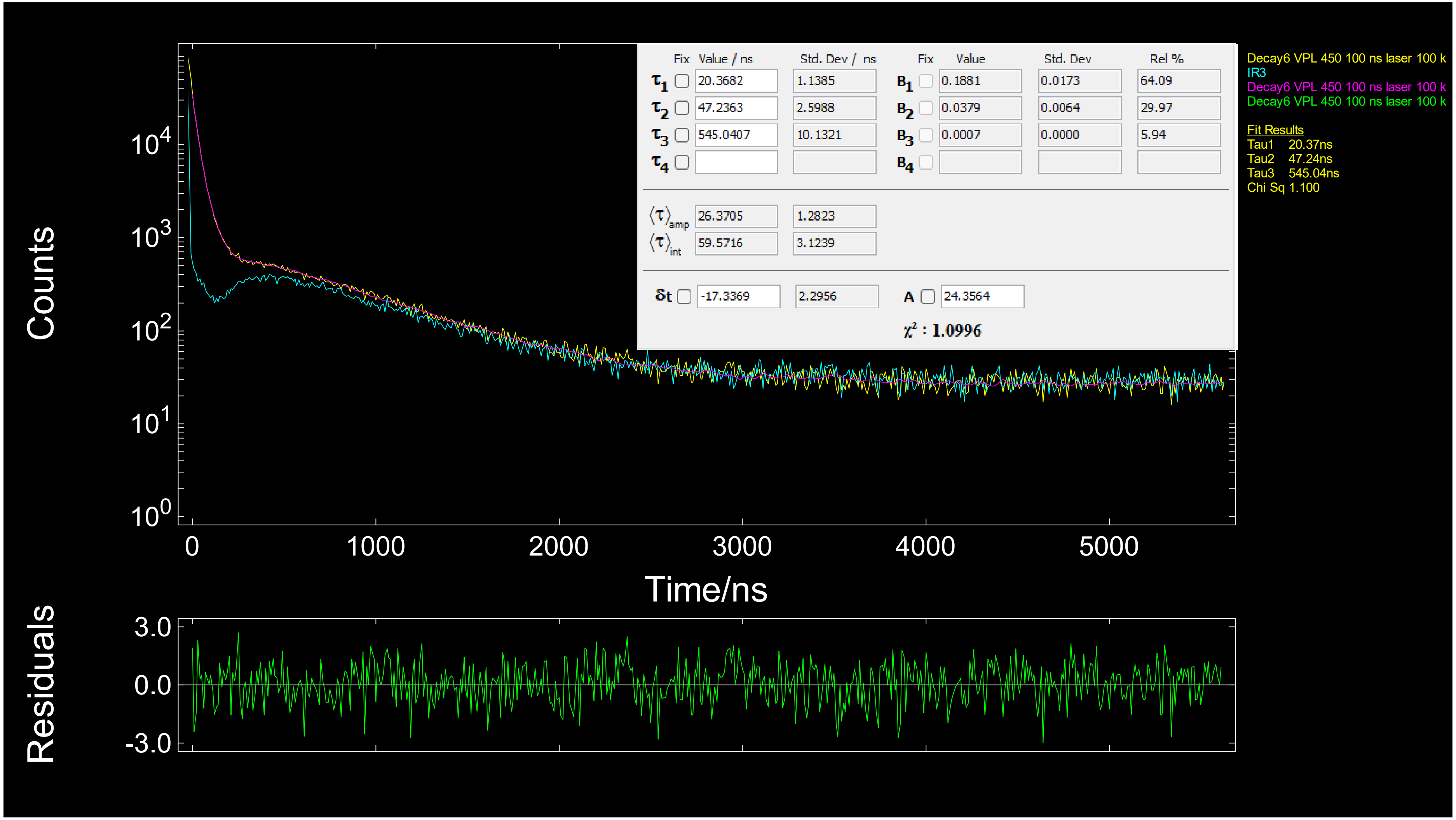

Figure S85. Experimental time-resolved luminescence decay (yellow) with IRF (blue) of **3**, DCM solution, 293 K. Numerical biexponential reconvulation fit (purple).

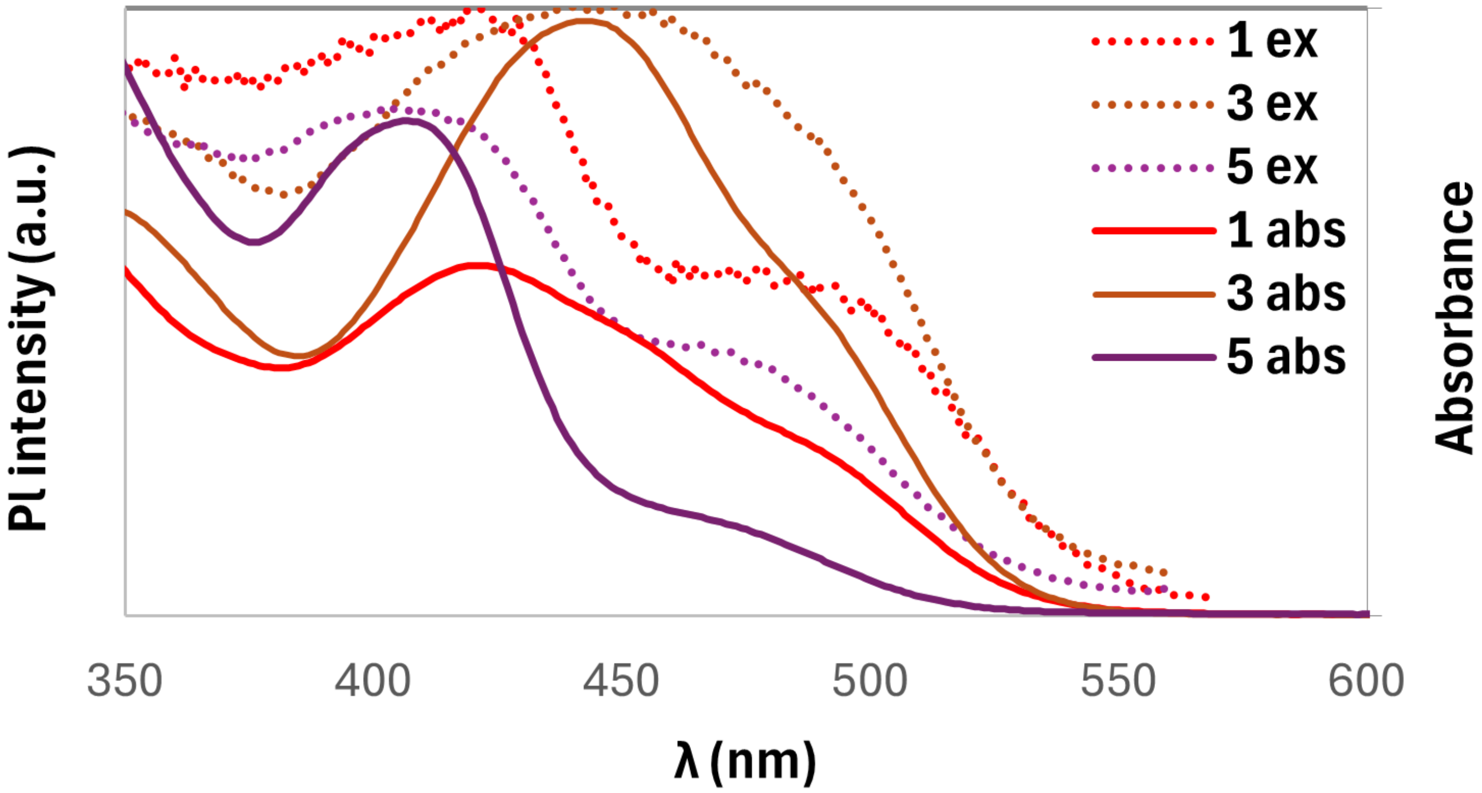

Figure S86. Correlation of absorption (solid lines) and excitation (dotted lines) spectra of **1**, **3** and **5** in DCM.

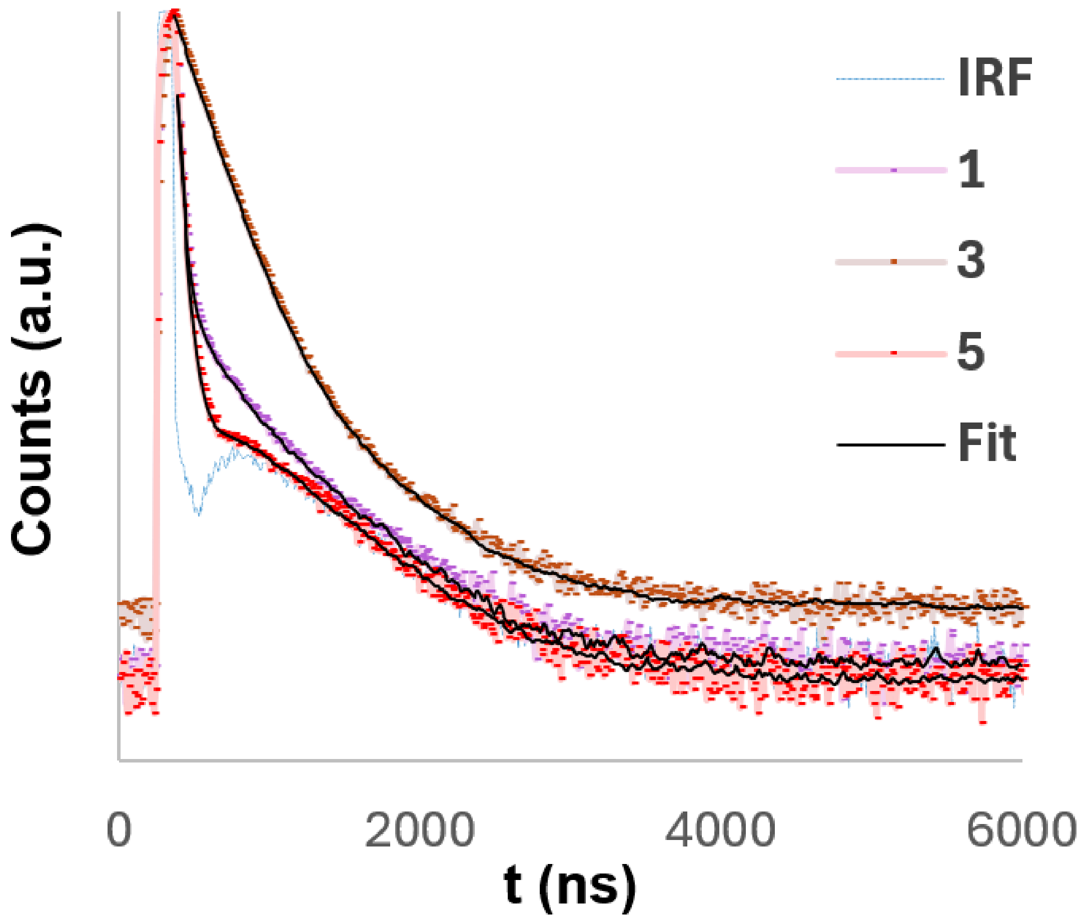

Figure S87. Lifetime comparison of **1**, **3** and **5** in DCM solution together with IRF.

Photophysical Properties of 1, 3 and 5 in the solid state

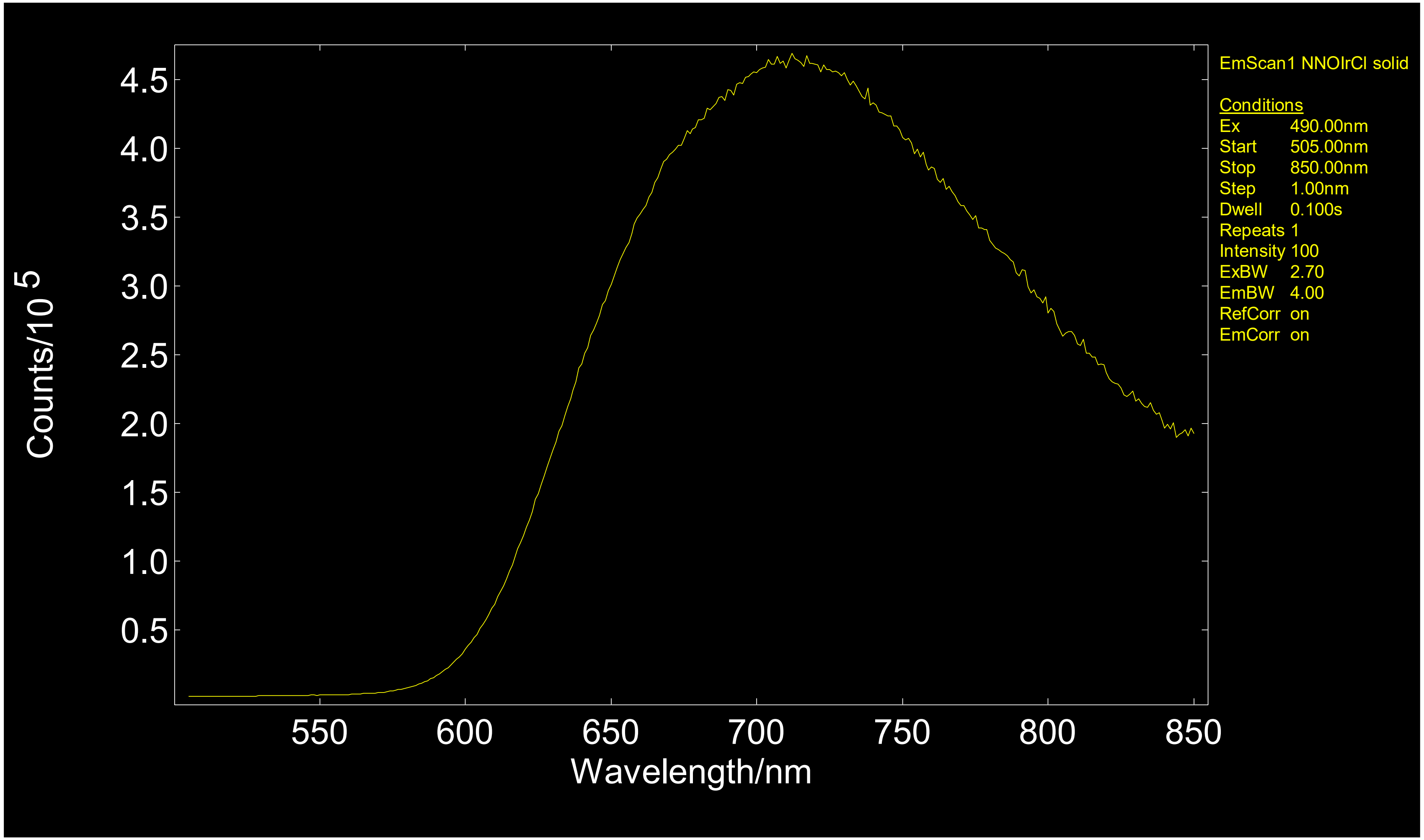

Figure S88. Emission spectrum of **1**, powder, 293 K, λ_{ex} = 490 nm.

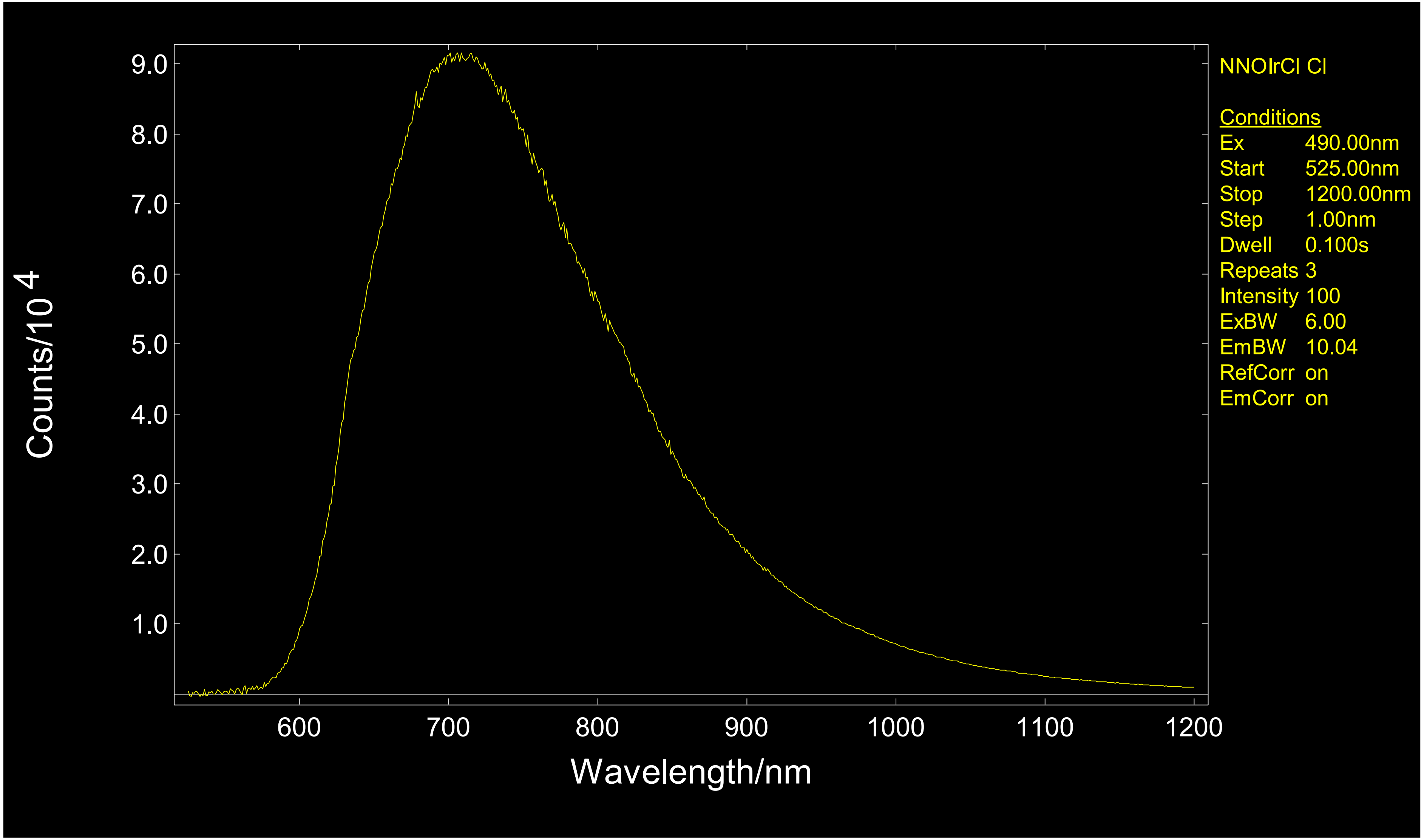

Figure S89. Emission spectrum of **1**, powder, 293 K, λ_{ex} = 490 nm, NIR detector.

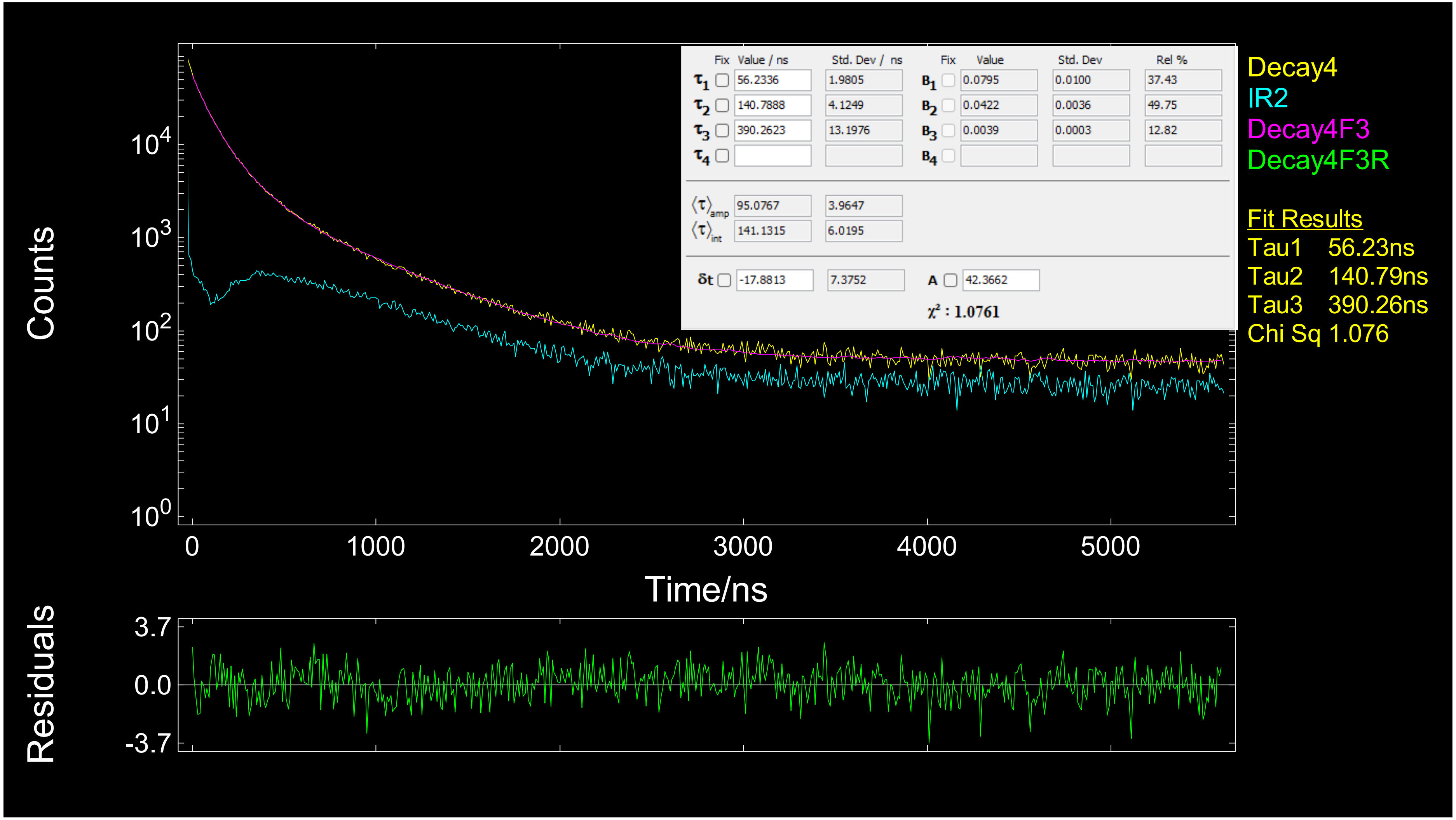

Figure S90. Experimental time-resolved luminescence decay (yellow) with IRF (blue) of **1**, powder, 293 K. Numerical triexponential reconvulation fit (purple).

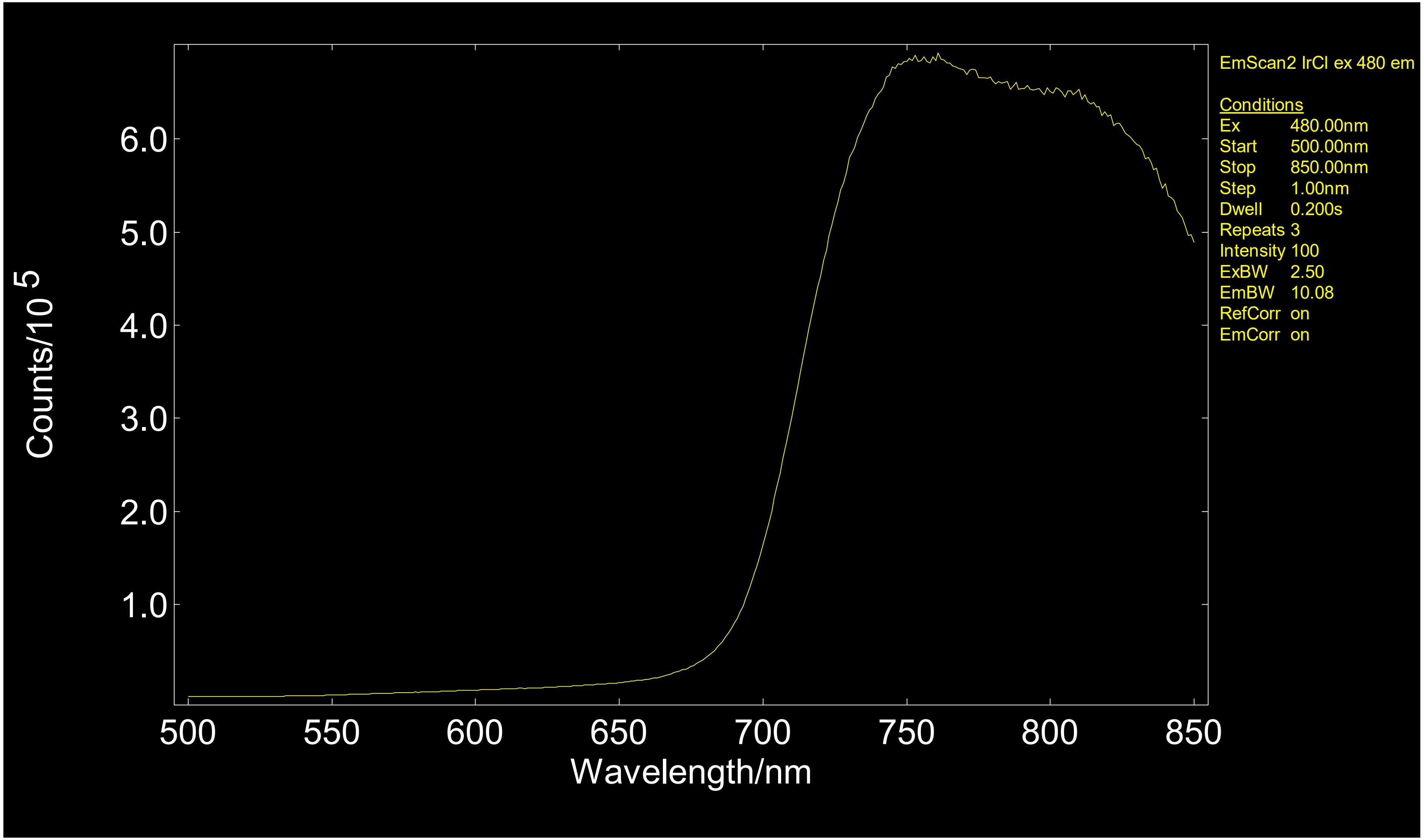

Figure S91. Emission spectrum of **3**, powder, 293 K, λ_{ex} = 480 nm.

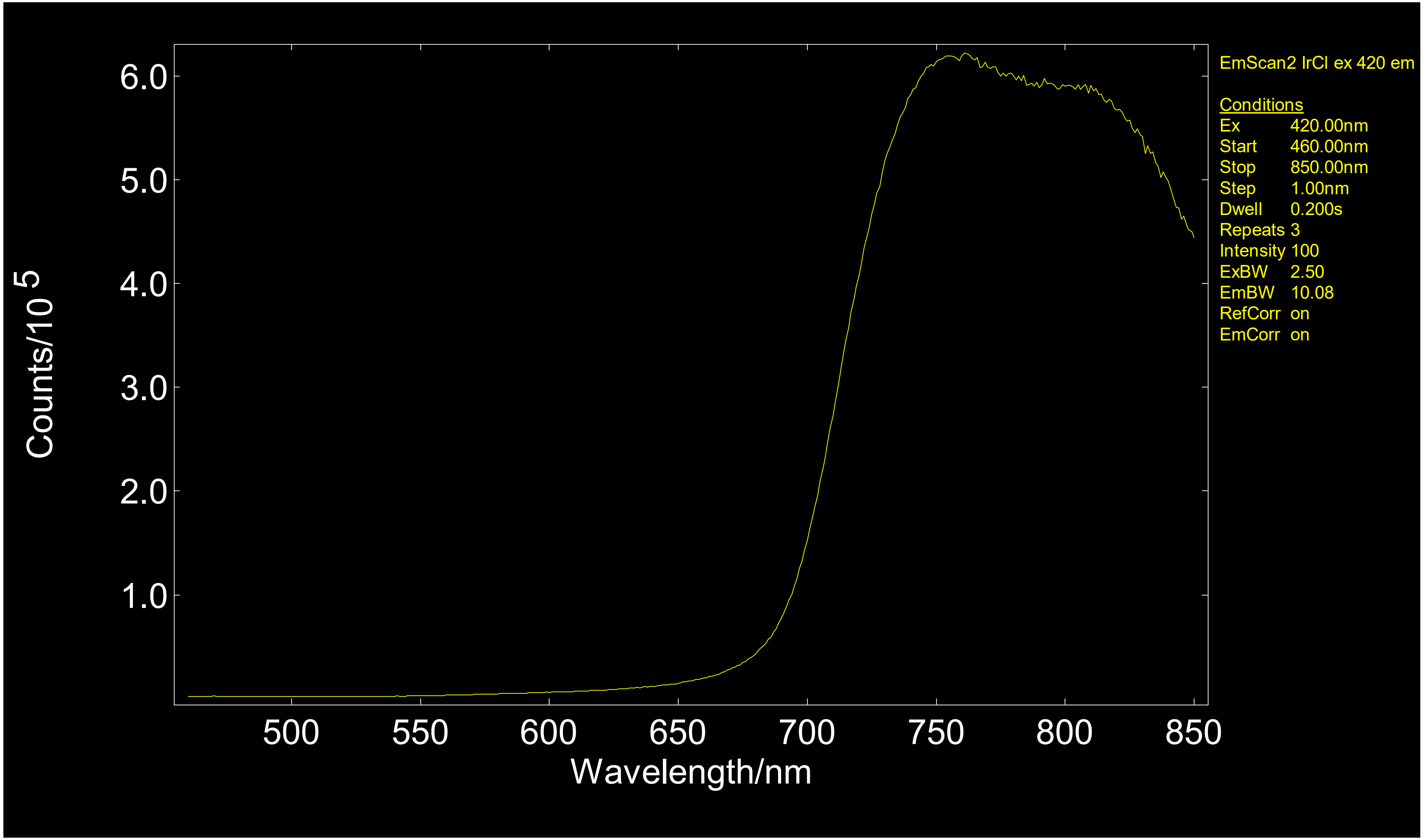

Figure S92. Emission spectrum of **3**, powder, 293 K, λ_{ex} = 420 nm.

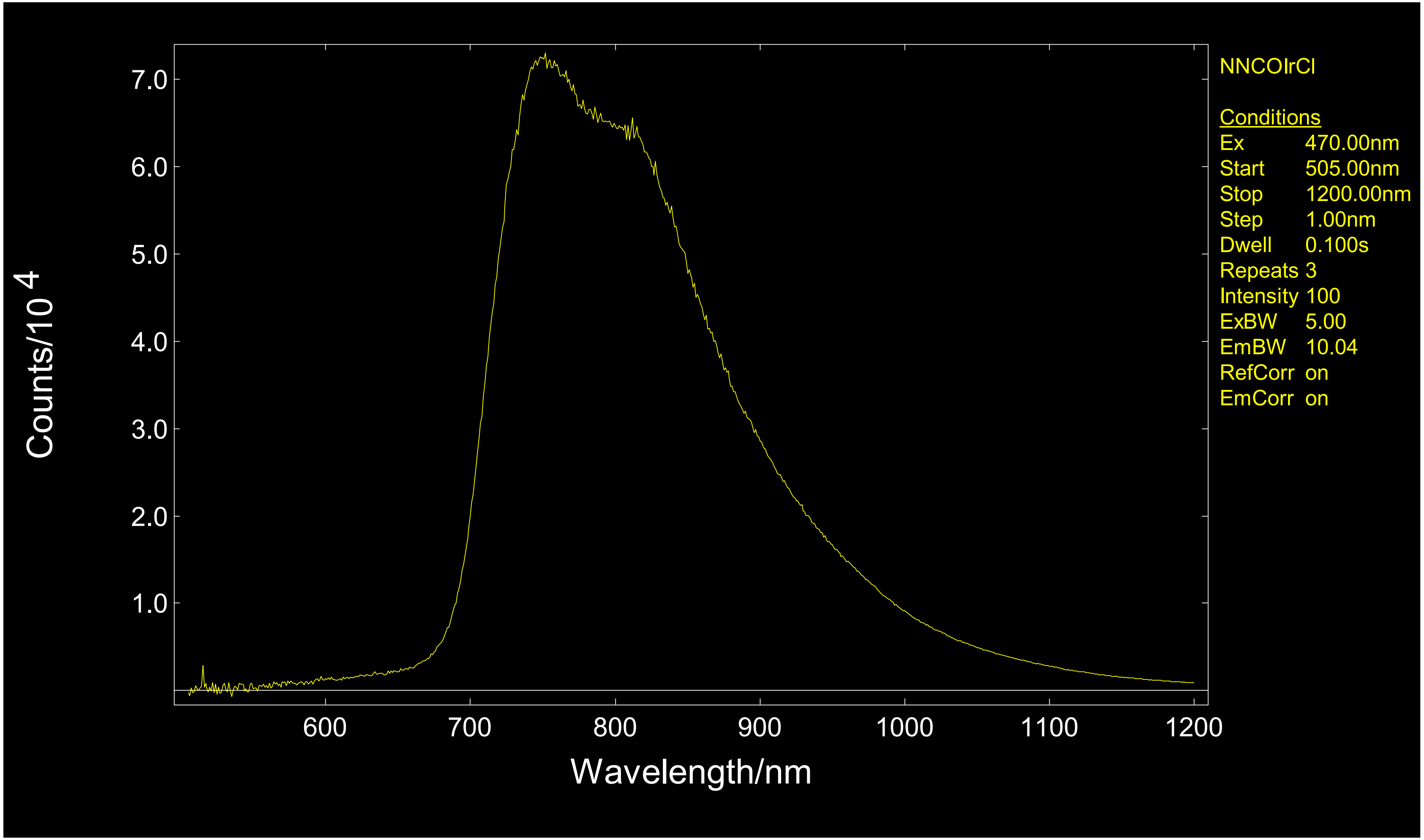

Figure S93. Emission spectrum of **3**, powder, 293 K, λ_{ex} = 470 nm, NIR detector.

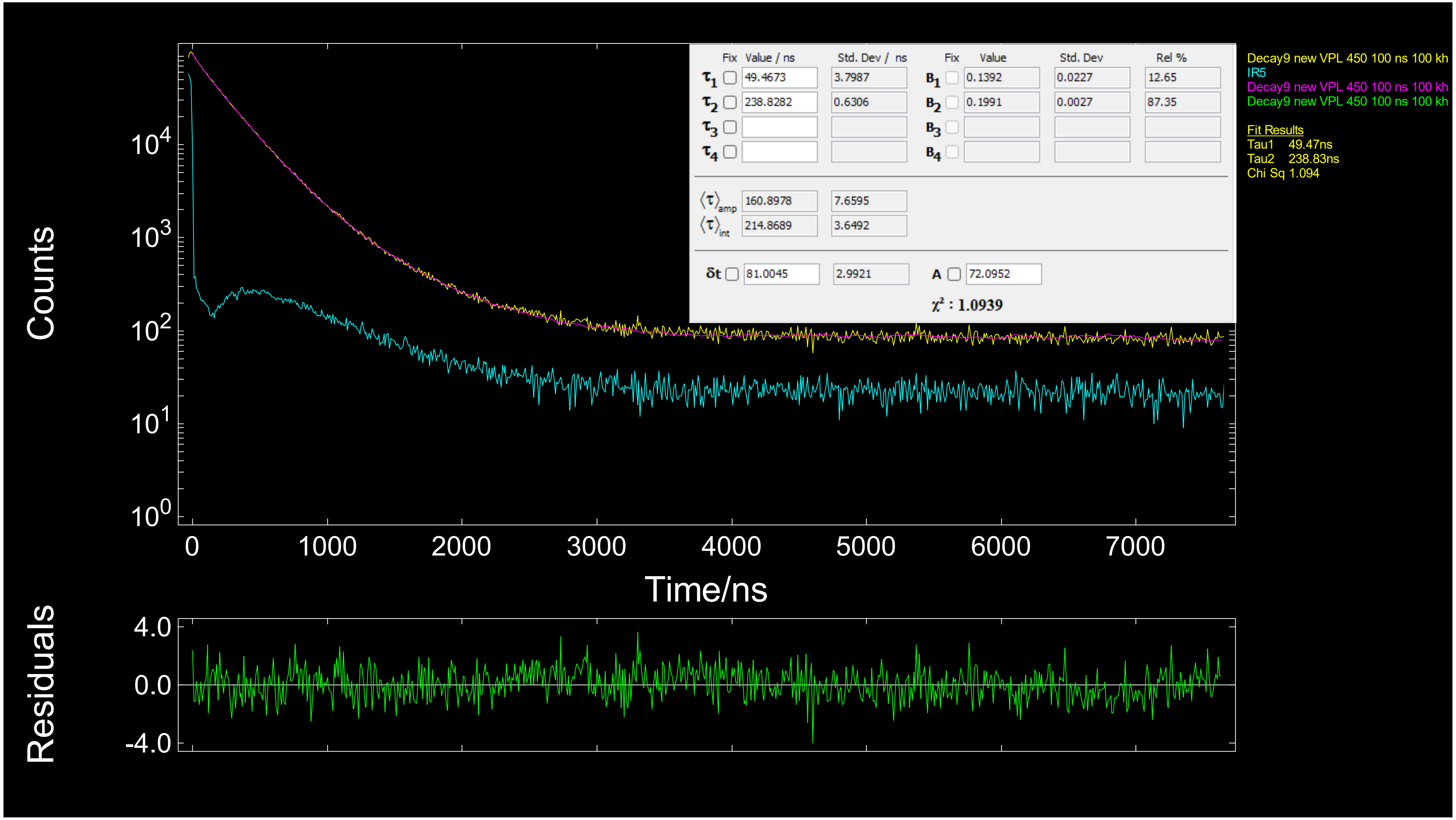

Figure S94. Experimental time-resolved luminescence decay (yellow) with IRF (blue) of **3**, powder, 293 K. Numerical biexponential reconvulation fit (purple).

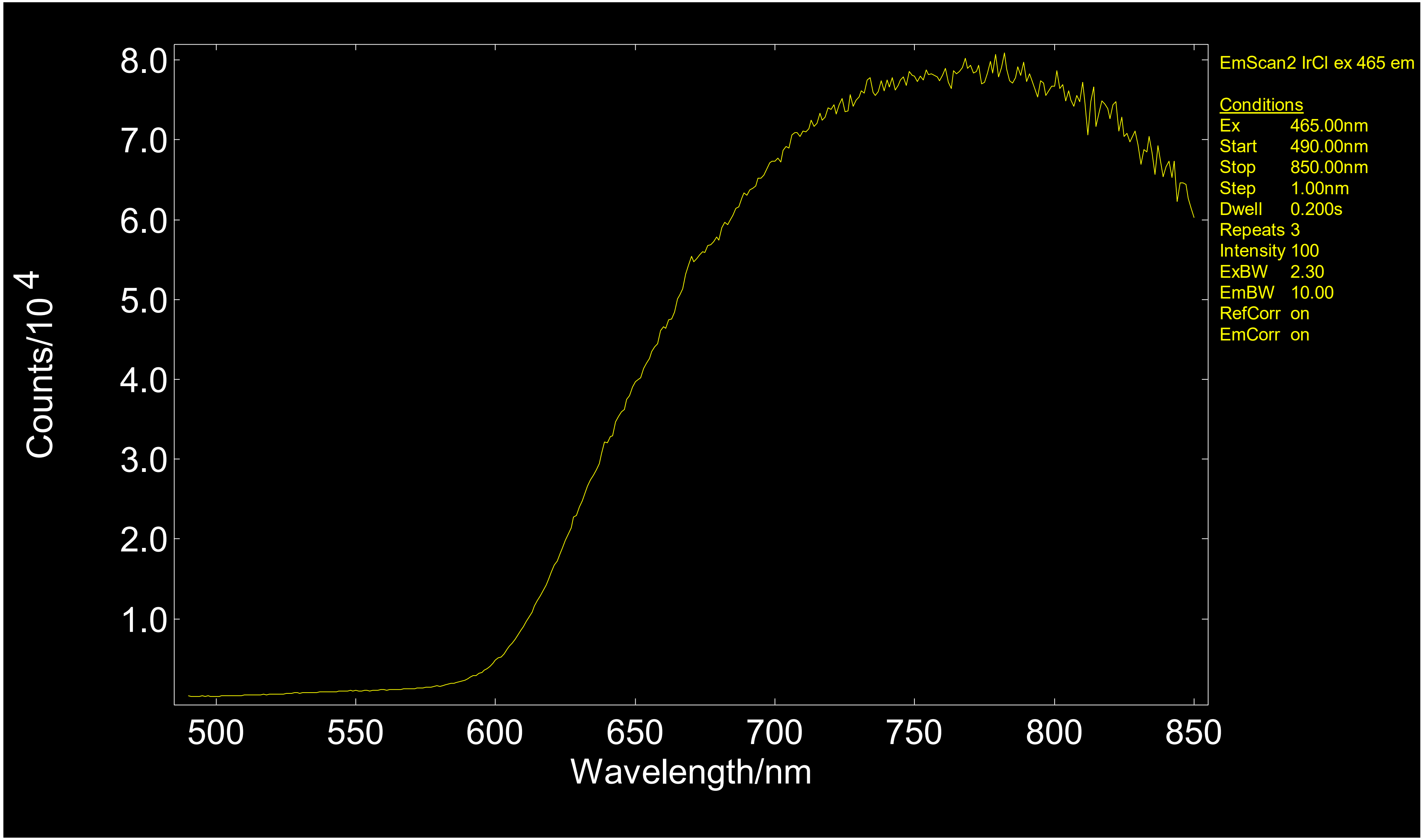

Figure S95. Emission spectrum of **5**, powder, 293 K, λ_{ex} = 465 nm.

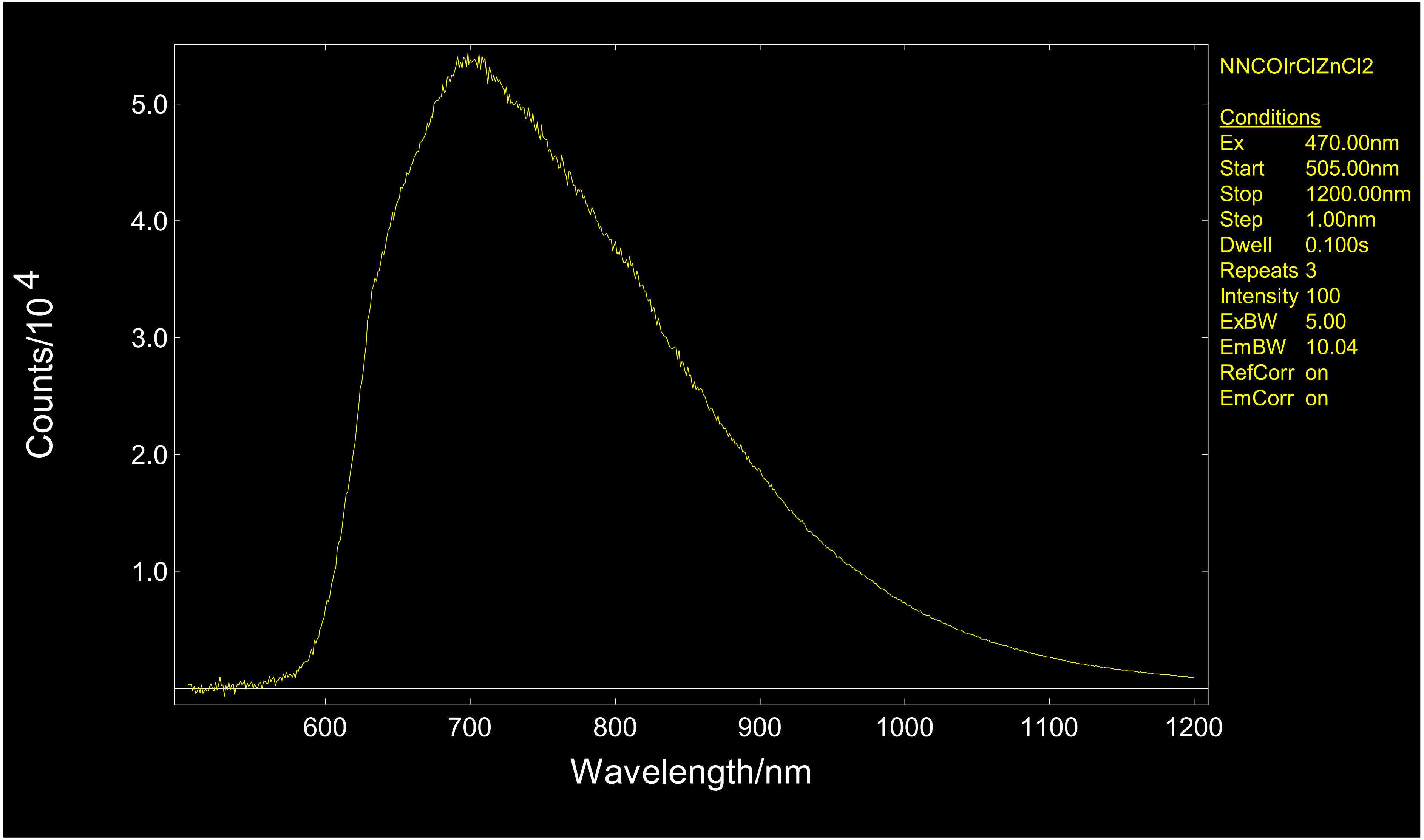

Figure S96. Emission spectrum of **5**, powder, 293 K, λ_{ex} = 470 nm, NIR detector.

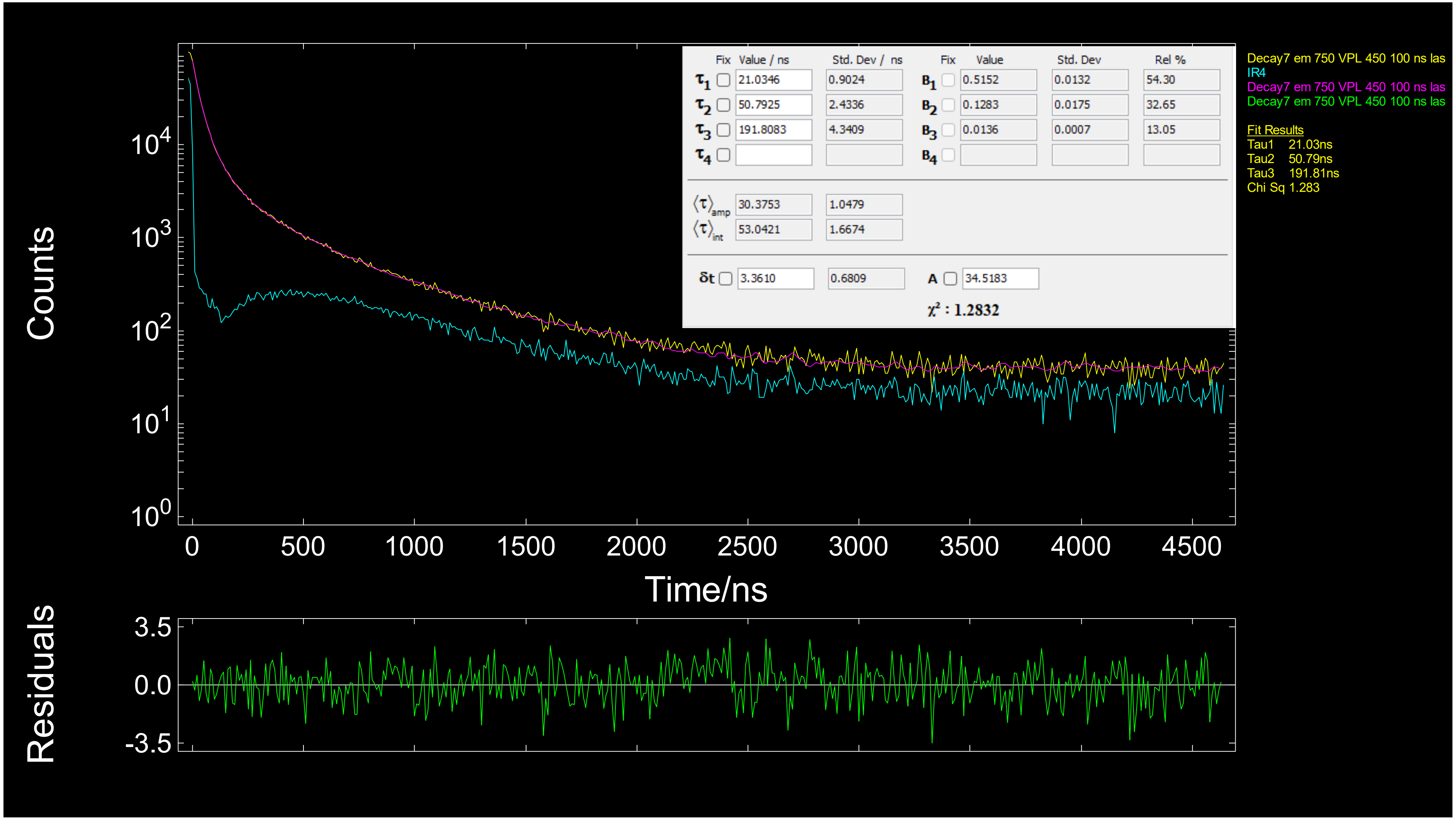

Figure S97. Experimental time-resolved luminescence decay (yellow) with IRF (blue) of **5**, powder, 293 K. Numerical triexponential reconvulation fit (purple).

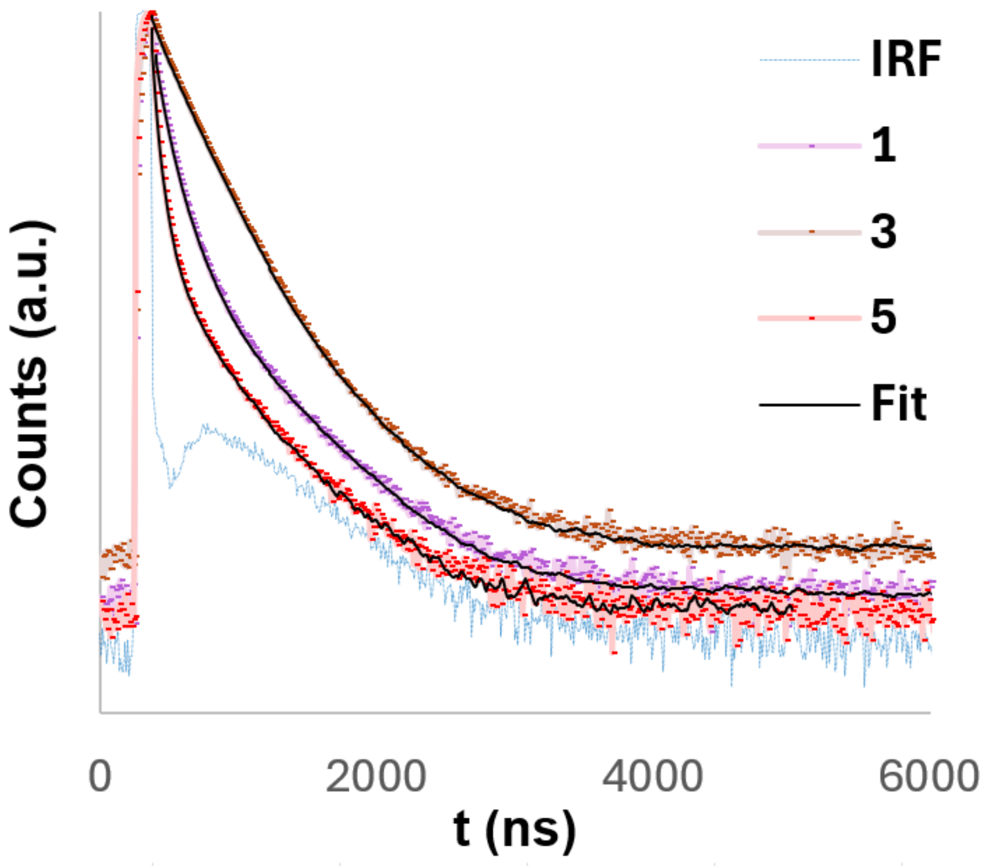

Figure S98. Lifetime comparison of **1**, **3** and **5** in powder together with IRF.

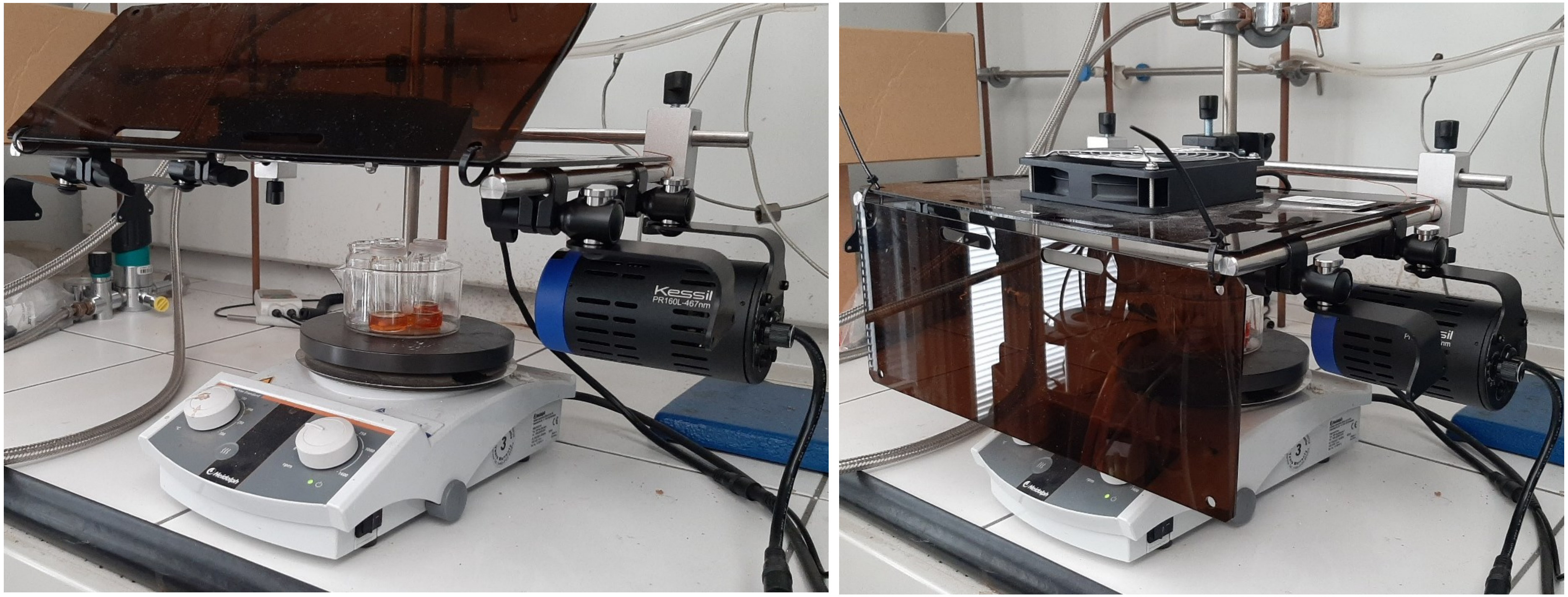

Figure S99. Blue Lamp (467 nm) for irradiation of **1** in DCM or powder. 12 h, RT.

References

[1] F. Neese, WIRES Comput. Molec. Sci., 2022 12(1)e1606.

[2] A. D. Becke, Phys. Rev. A. 38, 3098 (1988).

[3] F. Weigend and R. Ahlrichs, Phys. Chem. Chem. Phys. 7, 3297 (2005).

[4] F. Weigend, Phys. Chem. Chem. Phys. 8, 1057 (2006).

[5] D. A. Pantazis and F. Neese, J. Chem. Theory Comput. 5, 2229 (2009).

[6] D. A. Pantazis, X. Y. Chen, C. R. Landis and F. Neese, J. Chem. Theory Comput. 4, 908 (2008).

[7] D. A. Pantazis and F. Neese, Theor. Chem. Acc. 131, 1292 (2012).

[8] D. A. Pantazis and F. Neese, J. Chem. Theory Comput. 7, 677 (2011).

[9] J. D. Rolfes, F. Neese and D. A. Pantazis, J. Comput. Chem. 41, 1842 (2020).

[10] D. A. Pantazis, X. Y. Chen, C. R. Landis and F. Neese, J. Chem. Theory Comput. 4, 908 (2008).

[11] S. Grimme, J. Antony, S. Ehrlich and H. A. Krieg, J. Chem. Phys. 132, 154104 (2010).

[12] S. Grimme, S. Ehrlich and L. Goerigk, J. Comput. Chem. 32, 1456 (2011).

[13] J. P. Perdew, K. Burke and M. Ernzerhof, Phys. Rev. Lett. 77, 3865 (1996).

[14] C. Adamo and V. Barone, J. Chem. Phys. 110, 6158 (1999).

[15] E. F. Pettersen, T. D. Goddard, C. C. Huang, G. S. Couch, D. M. Greenblatt, E. C. Meng and T. E. Ferrin, J. Comput. Chem. 25, 1605 (2004)

[16] Du, Q.; Yang, Y.; Guo, L.; Tian, M.; Ge, X.; Tian, Z.; Zhao, L.; Xu, Z.; Li, J.; Liu, Z. Dyes Pigments 2019, 162, 821–830.

[17] Gonzalo-Navarro, C.; Zafon, E.; Organero, J. A.; Jalón, F. A.; Lima, J. C.; Espino, G.; Rodríguez, A. M.; Santos, L.; Moro, A. J.; Barrabés, S.; Castro, J.; Camacho-Aguayo, J.; Massaguer, A.; Manzano, B. R.; Durá, G. J. Med. Chem. 2024, 67, 1783–1811.

[18] Yang, Y.; Gao, Y.; Zhao, J.; Gou, S. Inorg. Chem. Front. 2024, 11, 436–450.
